# The Evolutionary History of R2R3-MYB Proteins Across 50 Eukaryotes: New Insights Into Subfamily Classification and Expansion

**DOI:** 10.1038/srep11037

**Published:** 2015-06-05

**Authors:** Hai Du, Zhe Liang, Sen Zhao, Ming-Ge Nan, Lam-Son Phan Tran, Kun Lu, Yu-Bi Huang, Jia-Na Li

**Affiliations:** 1 College of Agronomy and Biotechnology, Southwest University, Chongqing, 400716, China; 2 Key Laboratory of Biology and Genetic Improvement of Maize in Southwest Region, Maize Research Institute of Sichuan Agricultural University, Ministry of Agriculture, Chengdu Sichuan, China; 3 Department of Biological Sciences, National University of Singapore, 117543, Singapore; 4 Department of Cancer Prevention, Institute for Cancer Research, Norwegian Radium Hospital-Oslo University Hospital, Oslo, Norway; 5 Centre for Cancer Biomedicine, Faculty of Medicine, University of Oslo, Oslo, Norway; 6 Signaling Pathway Research Unit, RIKEN Center for Sustainable Resource Science, 1-7-22 Suehiro, Tsurumi, Yokohama, Kanagawa 230-0045 Japan

## Abstract

R2R3-MYB proteins (2R-MYBs) are one of the main transcription factor families in higher plants. Since the evolutionary history of this gene family across the eukaryotic kingdom remains unknown, we performed a comparative analysis of 2R-MYBs from 50 major eukaryotic lineages, with particular emphasis on land plants. A total of 1548 candidates were identified among diverse taxonomic groups, which allowed for an updated classification of 73 highly conserved subfamilies, including many newly identified subfamilies. Our results revealed that the protein architectures, intron patterns, and sequence characteristics were remarkably conserved in each subfamily. At least four subfamilies were derived from early land plants, 10 evolved from spermatophytes, and 19 from angiosperms, demonstrating the diversity and preferential expansion of this gene family in land plants. Moreover, we determined that their remarkable expansion was mainly attributed to whole genome and segmental duplication, where duplicates were preferentially retained within certain subfamilies that shared three homologous intron patterns (a, b, and c) even though up to 12 types of patterns existed. Through our integrated distributions, sequence characteristics, and phylogenetic tree analyses, we confirm that 2R-MYBs are old and postulate that 3R-MYBs may be evolutionarily derived from 2R-MYBs via intragenic domain duplication.

MYB proteins are ubiquitously expressed across eukaryotic organisms and comprise a major family of transcription factors in land plants. MYBs are involved in a myriad of regulatory processes, such as secondary metabolism^1^,^2^, morphogenesis^3^,^4^, and response to environmental stress (e.g., drought^5^, ultraviolet radiation^6^, and gibberellin^7^).

Typically, MYB proteins consist of one to four imperfect repeats (R1, R2, and R3), and these repeats are highly conserved amongst eukaryotic organisms. Each repeat contains about 50-54 amino acids and encodes three α-helices, with the second and third helices forming a helix–turn–helix (HTH) structure^8^,^9^,^10^ Accordingly, MYB proteins can be classified into four major types based on their number of repeats (R): 2R-MYB (R2R3-MYB), 3R-MYB (R1R2R3-MYB), 4R-MYB (R1R2R2R1/2-MYB), and MYB-related proteins (or 1R-MYB)^10^,^11^. Among the subtypes, 2R-MYBs are the most common, with two MYB repeats that are most similar to the R2 and R3 repeats of their vertebrate homologs, c-MYBs; they make up one of the largest transcription factor families in higher plants^9^,^11^,^12^.

Given its large size and critical role in diverse biological processes, genome-wide analyses of this gene family continue to be applied within specific species^13^,^14^,^15^,^16^. However, current knowledge regarding 2R-MYB distribution comes from scattered investigations on relatively small samplings. Moreover, none of the previous studies have shed light on the evolutionary relationship of 2R-MYBs in various land plants, especially in early-diverging groups (e.g., moss and lycophytes). This lack of knowledge has hampered our understanding of the origin and evolutionary history of this gene family. In general, many “orphan” genes and/or “species-specific” subfamilies have been proposed in the above mentioned studies^13^,^14^,^15^,^16^. It is unclear whether these orphan genes result from variations in a specific species, or if they are important genes with conserved numbers (generally one copy). It is also not apparent if species-specific subfamilies imply a lineage-specific distribution or just a loss of orthologs in the corresponding species. Moreover, large-scale analyses of this gene family have revealed massive gene duplication and divergence, which results in new subfamilies and novel gene functions^13^,^14^,^15^,^16^. However, of the subfamilies proposed by recent literature in the field, only the first 25 are consistent with the results of Stracke *et al.*, 2001; the remaining reported subfamilies are generally confused across studies^13^,^14^,^15^,^16^. Taken together, although 2R-MYBs have been identified and characterized in various plant species, systematic classification and a unified nomenclature are still lacking. It is therefore necessary to understand the history of this gene family in a more diverse evolutionary group so that we can elucidate and update our current knowledge of the members of this family.

In the present study, to gain insight into the evolutionary relationship of 2R-MYBs in the Plantae supergroup, we broadened our dataset to cover more representative plant lineages, including green alga, moss, tracheophytes, gymnosperms, basal magnoliophyta, monocots, and eudicots. Moreover, increasing evidence suggests that typical 2R-MYBs are not limited to plants^17^; therefore, we also explored the distribution of 2R-MYB genes in all eukaryotes. In order to accomplish this, we further broadened our dataset to include representatives of both bikonts and unikonts from 50 species. Accordingly, the comprehensive analysis of the relatively full set of 2R-MYBs from diverse species of eukaryotes enabled us to systematically classify this gene family and assess their origin and evolution. We were also able to determine patterns of differentiation and proliferation in various phylogenetic groups.

## Results

### Identification and phylogenetic analysis of 2R-MYB proteins in eukaryotes

**Figure 1:**
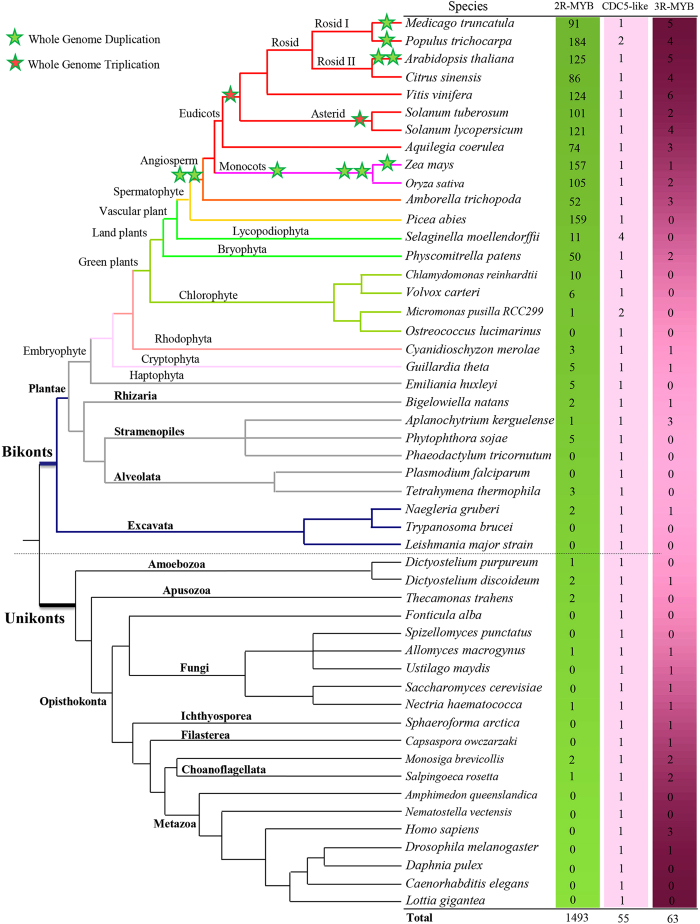
Phylogenetic relationships of 50 species investigated in this study. Phylogenetic relationships (branch lengths are arbitrary) among these species have been described previously (http://www.phytozome.net/). The total number of 2R-MYB proteins identified in each genome is indicated on the right. Green stars indicate whole genome duplication in the corresponding species; the red star denotes whole genome triplication (http://chibba.agtec.uga.edu/duplication/). Colored lines indicate the different lineages of the eukaryotes.

In contrast to earlier diverging groups of land plants that encoded a small number of 2R-MYBs, i.e., only 15 members in *Selaginella moellendorffii* and 51 in *Physcomitrella patens*, our results showed that a large number of 2R-MYBs were present in the genomes of angiosperms. Similarly, fewer 2R-MYBs were identified in the genomes of green and red alga (Fig. 1). This implies a much greater rate of gene expansion within higher plants. Strikingly, our results showed that typical 2R-MYBs, which are characterized by the presence of a highly conserved MYB domain^8^,^10^ (with the exception of CDC5-like proteins), are not limited to plants, but are present in other eukaryotic organisms. In contrast, 3R-MYBs were found to be encoded in most eukaryotes with conserved members (Fig. 1). Since CDC5-like proteins contain two MYB-like repeats^8^,^18^, which play conserved roles in pre-mRNA splicing and cell-cycle control at G2/M, they also function as transcription factors^19^,^20^,^21^,^22^,^23^,^24^. We accordingly included these MYB-related proteins in the present study. In order to distinguish between CDC5-like proteins and other candidates, we defined CDC5-like proteins as atypical 2R-MYBs, and the remainder of the proteins with typical characteristics of c-MYB-like domains^25^,^26^ were defined as typical 2R-MYBs. Furthermore, based on the latest versions of genome annotations, we re-identified 2R-MYBs in rice (*Oryza sativa*, v7.0), grapes (*Vitis vinifera* Genoscope.12X), and *Populus trichocarpa* (v3.0). Here, we observed that the numbers of genes in *Populus* (186), grape (125), and rice (106) were slightly different from those previously reported^14^,^27^,^28^,^29^, but were almost the same as the very recently modified numbers in these three species^30^. We concluded that the differences were mainly due to the updated versions of the genome annotation used in this study. In total, up to 1548 2R-MYB (including MYB-related CDC5-like proteins) and 63 3R-MYB candidate sequences were collected in the major lineages of eukaryotes (Fig. 1 and Supplementary Table S1).

**Figure 2:**
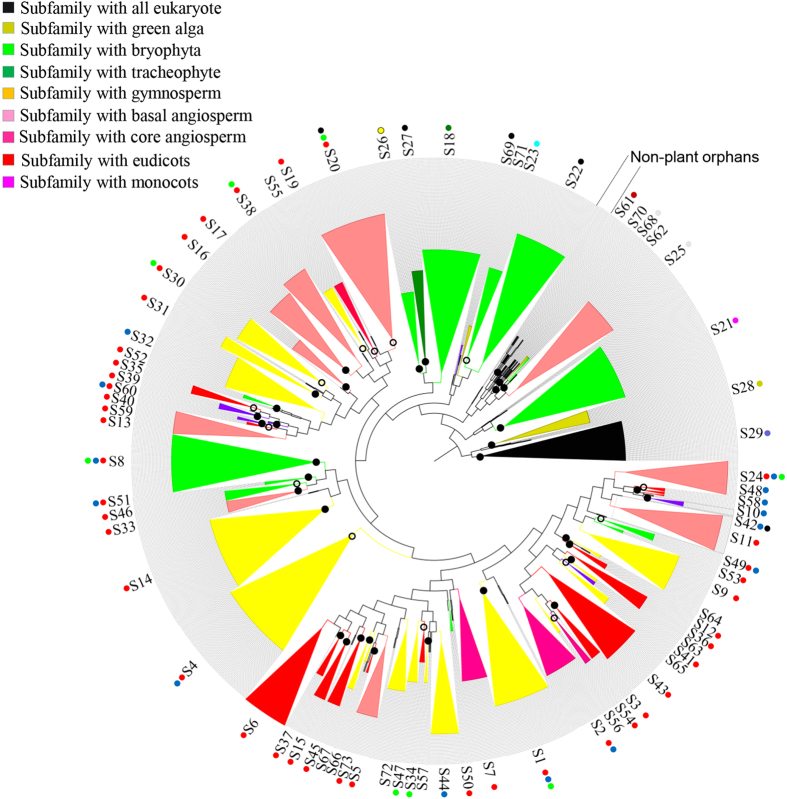
The phylogenetic tree and classification of 1548 eukaryote 2R-MYB genes. The neighbor-joining (NJ) tree includes 1548 2R-MYB proteins from 50 eukaryotes. Proteins are clustered into 73 subfamilies (colored triangles), designated with a subfamily number (e.g., S1). The depth and width of the collapsed triangles is proportionate to the corresponding sequence divergence and size, respectively. The colored block diagrams symbolize the species to which the proteins in each clade belong. Black dots beside the branches represent bootstrap support values from 1000 replications. Bootstrap values >50% are shown as black circles, >75% are shown as black dots, while <50% are not shown in the phylogenetic tree. The colored dots beside the subfamily names indicate the corresponding intron patterns, as shown in Fig. 4.

To understand the evolutionary relationship of the 1548 2R-MYB candidates, we performed an NJ and ML phylogenetic analysis based on multiple alignments of the MYB domains (Supplementary Fig. S1 online). Our results showed that tree topologies from these analyses were highly congruent. Based on the topologies and clade support values, candidates were classified into 73 major subfamilies with robust bootstrap support (Fig. 2). With the exception of the first 25 subgroups (which are well defined in *Arabidopsis*^9^,^11^), most of the newly identified 2R-MYBs, together with the previously defined “orphan” genes, could be clustered into many novel subfamilies with high bootstrap support values (subfamilies S26–S73, Supplementary Figure S1). These results can be attributed to the increased number of datasets used, and suggest that there are many more conserved subfamilies in plants than we knew. Moreover, these findings reveal that these subfamilies were readily neglected in previous studies due to the limited species investigated. The bootstrap values of some subfamilies were somewhat lower because up to 1548 sequences from 50 species were used. As will be discussed in the following sections, the reliability of our classification was well supported by additional features, such as sequence characteristics, intron patterns, conserved non-MYB motifs, etc.

In the phylogenetic tree, members from each species tended to cluster together within a given clade (subfamily), demonstrating that clades could be expanded after divergence from their common ancestor. For example, most dicot members fell into their own subclasses, which were separated from monocot plants, thus, exhibiting a lineage-specific expansion (Fig. 2). In general, unikont and bikont genes were separated from each other, with the exception of the S29 subfamily that consisted of MYB-related CDC5-like proteins. The remaining unikont genes were not clustered into any well-supported lineage- and/or species-specific clade, and were therefore defined as “orphan” genes whose evolutionary history needs to be further verified. In contrast, bikont genes, especially those from plants, likely evolved after the divergence of unikonts and bikonts, because the majority of 2R-MYBs were clustered into several compact clades (Fig. 2).

### The most recent common ancestors of eukaryotic 2R-MYB proteins

The difference in the family size of land plants (Fig. 1) may be a consequence of differential expansion among subfamilies. To further infer the degree of expansion in different subfamilies, we assessed the most recent common ancestor (MRCA) based on the assumption that shared clades composing of ortholog sequences were descendants of the same ancestral gene^31^.

**Figure 3:**
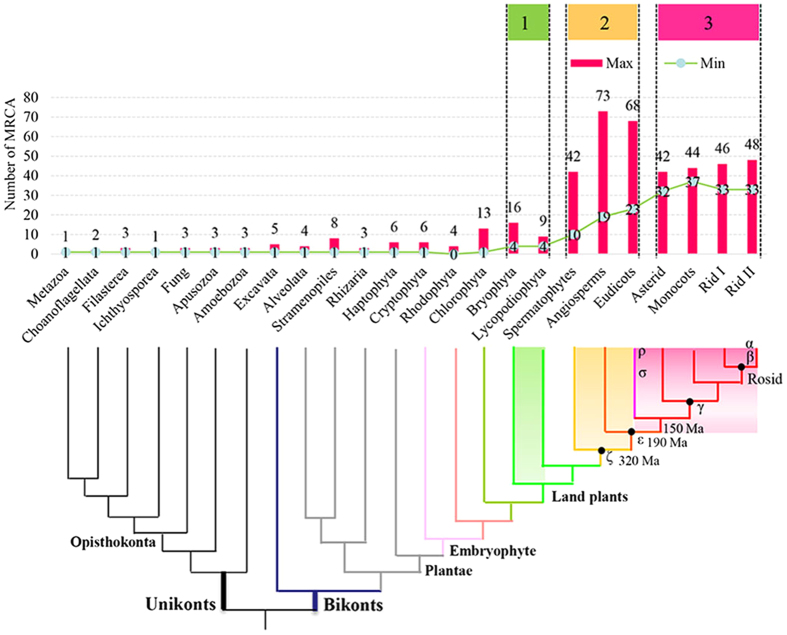
Expansion of the 2R-MYB gene family size in land plants. Estimates of the 2R-MYB gene family size in the most recent common ancestors (MRCA) of examined lineages. Numbers correspond to minimum (green line) and maximum (red block diagram) MRCA in each lineage.

Our results showed that a minimum estimate of one member would have been present in the MRCA of all eukaryotes (MYB-related CDC5-like protein); four in land plants; four in vascular plants; 10 in spermatophyte plants; 19 in angiosperm plants; 37 in monocots or grasses; 23 in dicots; 33 in Eurosids I, and 33 in Eurosids II (Fig. 3). Notably, MRCA members could have been underestimated owing to possible gene loss, pseudogenes, incomplete genes, and orphan genes. Accordingly, we also estimated the maximum number of 2R-MYB members in the MRCA of different evolutionary lineages, and determined that the actual number likely ranged between these two values (Fig. 3).

In other words, our findings revealed the presence of a 2R-MYB-related gene, CDC5-like protein, in the common ancestor of eukaryotes. Moreover, CDC5-like proteins appear to be older than typical 2R-MYBs or 3R-MYBs. An at least four-fold expansion of typical 2R-MYBs was observed in the deep lineages of land plants, and then up to 19-fold in the common ancestors of angiosperms (Fig. 3). Thus, the main diversification of 2R-MYBs occurred before the monocot and dicot split. Interestingly, each lineage continued to expand after this divergence, resulting in a multitude of lineage-specific subfamilies.

### The distribution and conservation of intron patterns in MYB domains

It was reported that the intron patterns of 2R-MYBs are greatly conserved in higher plants^13^,^14^,^15^,^16^. However, all of the current knowledge regarding the intron patterns of 2R-MYBs came from an investigation of angiosperms, with less attention being paid to the more ancestral groups of plants. Therefore, to gain insights into the traceable intron gain or loss, the origin and distribution of intron patterns in land plants, and the structural diversity of plant 2R-MYBs during evolution, we investigated the various exon/intron patterns and intron phases^32^ of each eukaryotic member. Importantly, only introns in the MYB domain were exploited since the remaining regions were extremely variable.

**Figure 4:**
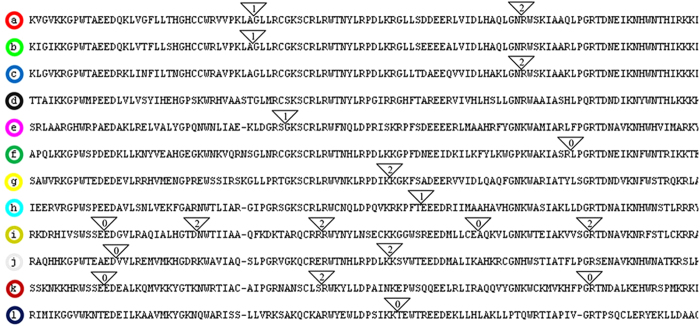
Schematic of intron distribution patterns within the MYB domains of land plant 2R-MYB genes. The alignment of MYB domains is representative of 12 intron patterns, named from a to l. Locations of introns are indicated by white triangles. The number within each triangle indicates the splicing phase of the MYB domain sequence: 0 refers to phase 0, 1 refers to phase 1, and 2 refers to phase 2. The number of 2R-MYB genes within each pattern is given in Supplementary Tables S2 and S3.

Our results showed that intron patterns were highly conserved from moss to angiosperms though the prevalence of 2R-MYBs was quite different, indicating that they were established in the common ancestor of land plants. In contrast, intron patterns in alga and non-land plant eukaryotes were variable and quite different from land plants. These findings suggest that land- and non-land plant lineages use different splicing mechanisms. In general, plant 2R-MYBs contained one to two introns, with the exception of a small number of intronless (Fig. 4, intron pattern d) and multi-intron genes (intron pattern i). In total, a maximum of 12 intron patterns were identified as being highly conserved across land plants (pattern a to l) in terms of absolute conserved intron insertion positions and phases. In other words, we presume that at least 10 independent intron insertion events occurred during the evolution of land plant 2R-MYBs, and 17 unique intron positions were identified in this study (Fig. 4). The striking dissimilarity between the majority of intron patterns may reciprocally lend support to the results of our phylogenetic analysis and classification (Fig. 2, and Supplementary Table S2).

In general, the intron patterns of land plant 2R-MYBs were exclusively conserved within each subfamily (Fig. 2, and Supplementary Table S2), suggesting the common origin of family members. However, these patterns were not absolute, and we found that a very small number of intron patterns could mix among the first three types (patterns a, b, and c) due to their high homology. Unlike the others, these three pattern types only exhibited a certain degree of variation, sharing great similarity in terms of gene structure and sequence characteristics. For example, intron patterns b and c possessed one of the two introns in pattern a, supporting their common origin. The differences in these three patterns might be derived from a single independent intron gain or loss from a parental gene during structural evolution. Given that about 70% of the members were found to share the first three pattern types, this is highly consistent with the observation of the rapid 2R-MYB increase in land plants. Therefore, it was inferred that intron gain and loss might play an important role in the divergence and expansion of this gene family. In contrast, the remaining genes formed up to nine intron types (d to l), most of which had very limited members in each species (Supplementary Table S3). This suggests that the corresponding genes may have distinct predispositions to the gain or loss of introns. Alternatively, it could suggest a difference in exon shuffling in the formation of all gene types. Notably, the last nine patterns tended to be neglected in previous studies due to rare members in each species. However, in our phylogenetic tree, the subfamilies sharing these intron patterns exhibited better topology, higher bootstrap values, and a conserved member in each species. These findings suggest that these patterns are of more ancient and conserved origins.

Taken together, our results revealed that the presence/absence and phase of introns could provide useful information on the evolution of 2R-MYBs.

### Conserved sequence characteristics of 2R-MYB proteins

**Figure 5:**
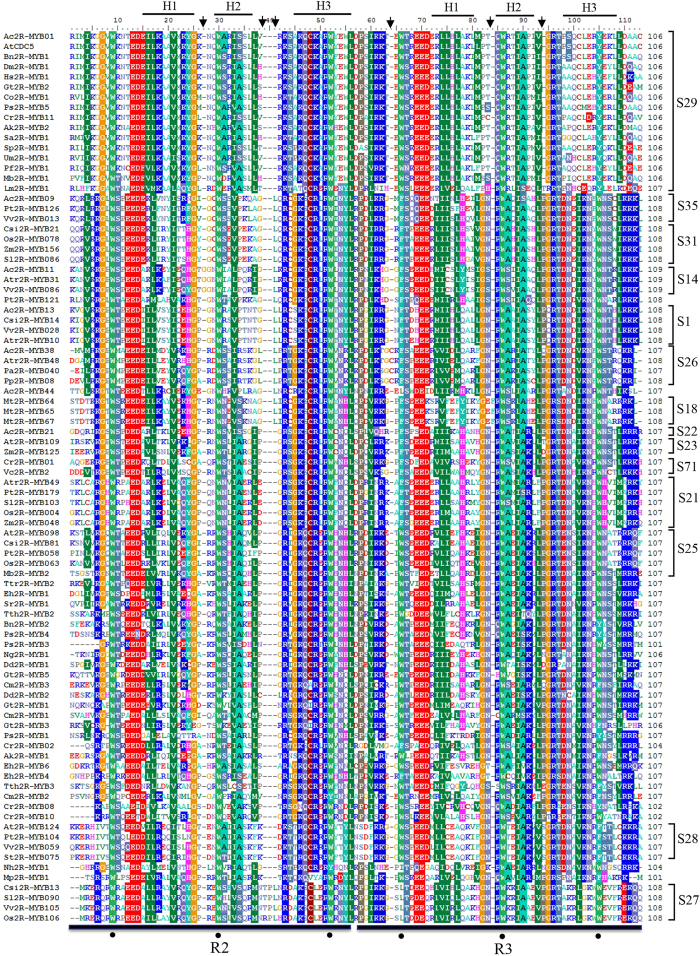
Alignment of the MYB domain of representative plant proteins. A representative of the plant 2R**-**MYBs is shown. MAFFT was used for amino acid sequence alignment of the MYB domains. The shading of the alignment represents different degrees of conservation among sequences. Asterisks indicate the conserved tryptophan residues (W) in the MYB domain. The positions of the three α-helices that form each MYB repeat are marked as Helix 1 to Helix 3. The black arrows indicate the conserved amino acid insertions in the MYB domains.

To investigate 2R-MYB sequence features across eukaryotes, we performed a multiple alignment analysis of the 1548 MYB domains (Fig. 5 and Supplementary Fig. S2 online). Despite the different evolutionary distances, the distribution of amino acids among the MYB domains was very similar, supporting its conservation during evolution.

Interestingly, except for the common conserved residues^9^,^15^, we observed a lineage-specific conservation of amino acid insertion/substitution at specific sites (Fig. 5 and Supplementary Fig. S2 online). This finding puts most of the 2R-MYBs in a clear monophyletic clade, resulting in subfamily and/or lineage-specific features. For example, a conserved G residue was found to be inserted between the first two helices of the R2 repeat in S14 subfamily members, while a conserved C residue was inserted in the R3 repeat of subfamily S26. Specifically, the Cys-41 (C) in the R2 repeat, an essential region for DNA-binding activity, has been found to be highly conserved in typical MYB domains during evolution^33^. However, a few old subfamilies (S21, S22, S23, and S28) and non-plant 2R-MYBs have a specific substitution at this location. In addition, we observed that about 80% of plant 2R-MYBs had a conserved G or Leu (L) residue insertion in the R2 repeat when compared to ancient subfamilies (S21, S22, S23, S25, and S41) and unikont genes. It should be noted that the insertion of the L residue is consistent with the formation of the first intron in pattern a, implying a possible role of the L residue in this pattern formation. Subsequently, this intron was retained throughout evolution, resulting in the large size of land plant 2R-MYBs containing this conserved insertion. These findings suggest that 2R-MYBs may be more variable in structure, and that the insertion of this intron may result in functional diversification. Together, our findings suggest that conserved residue substitutions in different subfamilies can be regarded as a signature of key 2R-MYB variants during evolution. This may provide a valuable clue to the common origins of closely related 2R-MYB members. Finally, the functional properties of these sites need to be further estimated and/or confirmed by mutation analyses; however, this is beyond the scope of the current study.

Although there are some reports about the identification of non-MYB motifs of 2R-MYBs^9^,^13^,^14^,^15^,^16^, there still lacks a systematic study across land plants. In the present study, based on large number of species used, up to 102 highly conserved motifs were identified outside of the MYB domains in different subfamilies (Supplementary Table S4). Our results were consistent with the previous studies^9^,^13^,^14^,^15^,^16^, and expanded the prior work (Supplementary Table S4). With the exception of motif 1, which has been identified as being adjacent to the R3 repeat and conserved within several subfamilies, most of the motifs identified in this study are likely subfamily- and/or branch-specific. This indicates that motif 1 remained alongside the MYB domain during evolution. Moreover, these motifs are most likely selectively distributed amongst specific clades or subfamilies. Nearly all motifs were identified in the orthologous gene of a subfamily, giving further support to the phylogenetic analysis based on MYB domains. Some conserved motifs were already present in the ancestors of land plants (such as moss), suggesting that they originated at the very beginning of land plants. Although the functions of most of the motifs remain unknown, it has been suggested that the functional conservation of 2R-MYBs might be associated with specific and/or similar motifs. For instance, motifs 77 (BOX1), 78 (BOX2), and 82 (BOX3) in the S18 subfamily have been characterized as having additional functional properties for GAMYB-like proteins^34^. Additionally, motif 15 in the S4 subfamily has been characterized as a C2 repressor motif^6^. Together, our findings demonstrated that the domain/motif architecture was remarkably conserved within a specific subfamily, which might indicate a common origin and/or close relationship.

## Discussion

### 2R-MYB proteins exist ubiquitously in eukaryotes

In this study, we investigated the distribution of the 2R-MYB gene family across 50 major lineages of eukaryotes (Fig. 1). It is widely acknowledged that 3R-MYBs are distributed and functionally and structurally conserved across the eukaryotic kingdoms. It is also known that 3R-MYBs existed before the divergence of animal and plant lineages^19^,^25^,^35^,^36^. However, similar to 3R-MYBs, our most striking result was that typical 2R-MYBs (with the exception of CDC5-like proteins), which had been reported to be plant specific^8^, were broadly present in the major eukaryotic supergroups (Fig. 1).

Our findings prove that typical 2R-MYBs are much older than what had previously been thought, likely forming before the massive radiation of eukaryotes. This proves that the occurrence of typical 2R-MYBs outside of plants is not occasional, but widely present in bikonts and lower organisms (e.g., unikonts). Interestingly, only atypical 2R-MYBs (CDC5-like proteins) seem to be present in all eukaryotes, since at least one atypical member could be detected in each organism we investigated. This wide distribution supports the idea that atypical 2R-MYBs had ancient origins in the eukaryotic tree, where their low abundance in each major group suggests that they evolved to be highly conserved throughout eukaryotes.

The large number of 2R-MYBs in land plants contrasted with the relatively small size of 2R-MYBs in other eukaryotes. This implies that a different type of expansion occurred in plant lineage. Accordingly, our results showed that 2R-MYBs underwent a rapid expansion during the evolution of Plantae (i.e., from alga to land plants). For instance, a small number of 2R-MYBs were found in the genome of aquatic chlorophytes (Fig. 1) (e.g., only eight 2R-MYBs were encoded in the single-celled chlorophyte, *Chlamydomonas reinhardtii*). In contrast, we observed a gradual increase in gene number with the differentiation of plants into organisms of higher complexity. For example, one basal lineage of land plants, moss (e.g., *P. patens*), has been determined to encode up to 51 members. Similarly, another early-diverging group of land plants, *Selaginella moellendorffii*, was found to encode 15 2R-MYBs. However, in higher plants this number was much larger, suggesting that a huge expansion occurred after the divergence of angiosperms from vascular plants.

Owing to the recently completed genome sequences of gymnosperms (*Picea abies*) and basal magnoliophyta (*Amborellaceae trichopoda*), we were able to trace the evolutionary history of 2R-MYBs in spermatophytes and basal angiosperms (Fig. 1). This allowed us to investigate the mechanism underlying the unexpected expansion of this gene family in higher plants. Here, we noted that the number of 2R-MYBs in *A. trichopoda* did not show a significant over-representation, which may be due to the incomplete nature of the draft form of the genome sequence that is currently available. However, a very large number of 2R-MYBs identified in *P. abies*, suggest that an obvious expansion occurred after the independent differentiation of gymnosperms and angiosperms. Taken together, our results demonstrate that 2R-MYBs are ubiquitous in eukaryotes, and that a rather drastic expansion occurred independently among land plant lineages. Our results also confirmed that there is a wide distribution of typical 2R-MYB proteins amongst diverse taxonomic groups, indicating the ancient origin of this type of MYB gene.

### The evolutionary history of 2R-MYB genes in land plants

In the present study, we classified the 1548 candidate 2R-MYBs into 73 subfamilies based on topologies and support values of the phylogenetic trees, which represented the main evolutionary relationships of this gene family in eukaryotes (Fig. 2). Based on the substantial number of species sampled, we were able to confirm and/or re-define some of the lineage-specific subfamilies that had previously been neglected. For instance, the subfamilies S35, S43, and S45 proved Eudicot-specific, as most of the eudicots had members included in these subfamilies. Meanwhile, these subfamilies implied a loss of homology in *Arabidopsis*^9^ (Supplementary Fig. S1 online). Similarly, it was previously suggested that there was a specific expansion of 2R-MYBs in grass lineage^13^,^15^,^37^, and our analyses confirmed that more subfamilies such as S39, S60, and S69 were grass- and/or monocot-specific. In fact, our findings suggest that there might be many more species-specific subfamilies than we originally expected, due to the fact that certain species appeared to have no apparent counterpart in the other investigated species. For example, S10 and S12 subfamilies are likely either *Arabidopsis*- or Cruciferae-specific, supporting the fact that the expansion and diversification of 2R-MYBs continued to occur throughout the evolution of angiosperms. The species-specific 2R-MYBs may be genomic relics that have evolved independently. Alternatively, these 2R-MYBs may have unique functions in particular species. For example, S12 members are specific regulators of Cruciferae-specific glucosinolate biosynthesis^38^. Together, our results indicate that there might be many more lineage-specific subfamilies whose evolutionary relationships could be further solved by using more representative species.

Our phylogenetic analysis also allowed us to assess the origin and evolutionary relationships of different subfamilies. For example, the S29 subfamily included at least one representative of each species we investigated, indicating that it might have originated very early in the common ancestor of unikonts and bikonts (Fig. 2). With the exception of the S29 subfamily, the rest of the algae 2R-MYBs tended to cluster at the base of S25 and S23 subfamilies. Moreover, none of the red algae genes could be clearly allocated to any plant 2R-MYB subfamily. This may suggest that plant 2R-MYBs were established after the divergence of red algae from chlorophytes 1.5 billion years ago^39^. Alternatively, the common ancestor of Plantae and red alga did derive 2R-MYBs, thus, it is possible that red alga independently lost these proteins in their later evolution. Similarly, the presence of 2R-MYBs in *Volvox carteri*, *C. reinhardtii*, moss, and angiosperms revealed that the S28 subfamily was probably derived after the divergence of chlorophytes from the ancestors of land plants over 1 billion years ago^40^. In contrast, the S18, S21, S22, S26, and S27 subfamilies might have evolved earlier after the divergence of land plants, because they were all retained in the deep lineage of land plants. In other words, these apparent ancient subfamilies may have been present since the origin of land plant 2R-MYBs. Subsequently, the major land plant-specific subfamilies have been established as monophyletic clades with robust support.

Of the 73 subfamilies, 35 included angiosperm proteins only, 32 included monocot and dicot proteins, 17 included gymnosperm and angiosperm proteins, five included lycophyte and angiosperm proteins, nine included moss and angiosperm proteins, four included moss, lycophyte, and angiosperm proteins, two included chlorophytes and land plants, and only one was shared by all eukaryotic organisms. These subfamilies might represent the descendants of ancestral land plant 2R-MYBs and the initiation of gene expansion in green plants. Since the last common ancestor of angiosperms and lycophytes lived about 415 million years (My) ago^41^, the 35 angiosperm-specific subfamilies were probably formed 415 My ago. The 32 subfamilies present in both monocots and eudicots indicates that the appearance of most 2R-MYB genes in plants predates the divergence of monocots and eudicots, while the nine subfamilies (including vascular plants and/or moss 2R-MYBs) may have originated prior to the emergence of vascular plants, approximately 440 My ago^42^. Given the small number of 2R-MYBs found in chlorophytes, as well as their lack of clear phylogenetic relationship with other eukaryotic genes, we suggest that land plant 2R-MYBs evolved after the primary endosymbiotic event that led to the evolution of plastids (an event that is not represented in other eukaryotic groups).

### Expansion mechanisms of 2R-MYB genes in land plants

Gene duplication, which arises from genome-wide (polyploidization) or region-specific duplication, has proven to be a prominent feature of plant genome evolution, and contributes to the establishment of multi-gene families^43^,^44^.

It has been reported that whole genome duplication (WGD) is a driving force in angiosperm diversification^45^. Here, the tremendous expansion of 2R-MYBs in land plants is consistent with WGD in angiosperms, which could explain the explosive expansion of this gene family (Fig. 3). If this were the case, it would support an important role for the expansion of 2R-MYBs in increasing the complexity of higher plants. The first expansion of plant 2R-MYBs might have been established over 440 My ago, by which time the land plants had diverged from the Chlorophyta^42^. Subsequently, these ancestral units (subfamilies) underwent various expansion rates and retentions in different land plant lineages, resulting in a huge number of 2R-MYBs in diverse species. The second round expansion likely occurred after the divergence of spermatophytes from vascular plants, which would have led to the WGD event of seed plants about 320 My ago. Finally, the third round of expansion would have taken place in the common ancestor of angiosperms, forming the majority of 2R-MYBs subfamilies about 190 My ago. Interestingly, and consistent with previous studies^37^, we found that the rapid expansion of 2R-MYBs is still contiguous, as there have been larger events in core angiosperms before the split of monocots and dicots around 150 My ago (from 19 of MRCA to 22).

In order to investigate the roles of small-scale duplications (e.g., tandem/segmental duplication events) in the expansion of 2R-MYBs, we analyzed the rate of these events across land plants. In most cases, more than 20% of 2R-MYBs in each plant had paralogous counterparts in the syntenic regions of related chromosomes, demonstrating that segmental duplication accounts for a large amount of the expansion of this gene family in land plants (Table 1). Interestingly, it was apparent that the rate of 2R-MYBs involved in segmental duplications in dicots was higher than those of monocots, implying a heterogeneous duplication rate in the two lineages. In addition, a series of tandem duplication genes were identified in each species and were determined to contribute to the diversity of 2R-MYBs. In comparison, tandem duplication genes were generally found to be bipartite and randomly distributed in different subfamilies. However, we found that the genes from five species in subfamily S6 were all tandem duplication genes with three to six repeats. Thus, small-scale duplications were also crucial for the expansion of this gene family. Taken together, these findings imply a possible conserved mechanism that determines whether tandem duplication occurs.

The gene duplicates generated by WGDs or small-scale genome duplications allowed for the possible evolution of novel gene interactions and functions. Moreover, it has been reported that the evolutionary mode of genes occurs in a biased manner after duplication^46^. It could be that gene-balance expanded plant families, including those encoding proteasomal proteins, protein kinases, motors, and transcription factors, explain trends involving complexity^45^. In our study, we observed that although several independent lineage-specific WGD events in angiosperms occurred after lineage divergence, no significant differences of MRCA 2R-MYBs were revealed in main lineages, such as monocots, asterids, and rosacea (Fig. 3). This may suggest that in order to keep their numbers in relative balance, 2R-MYBs exhibit dosage sensitivity after large-scale duplications. Moreover, gene retention seemed to be biased toward specific gene categories with intron patterns a, b, and c, resulting in a huge expansion of 2R-MYBs within the first three types. In contrast, the last nine intron patterns (d-l) seemed to ignore dosage sensitivity. These results indicate that duplicates of 2R-MYBs were preferentially retained in subfamilies with three highly homologous intron patterns (a, b, and c), resulting in a massive expression of this gene family in higher plants. It was demonstrated that the expansion of subfamilies S6 and S7 had affected wine quality^14^; S5, S6, and S44 woody-expanded subfamilies^30^; and the expansion and subsequent sub-functionalization of subfamily S12 in *Brassicaceae* species played a lineage-specific role in the regulation of glucosinolate biosynthesis^14^,^38^. Similarly, as reviewed in a recent study^29^, our results confirmed that the rapid expansion of 2R-MYBs in land plants occurred in response to selection for the functions of specific plant processes (Supplementary Table S3).

Taken together, our observations suggest that 2R-MYBs underwent major radiation after the evolution of vascular plants, probably due to WGD, segmental duplication, and tandem duplication. Thus, despite several rounds of gene duplications and losses that occurred independently amongst the various plant lineages, the majority of subfamilies in the plant kingdom have been relatively conserved thanks to the dosage balance hypothesis.

### Origin and evolution of plant 2R-MYB proteins

Our results raise a further problem regarding the evolutionary relationship of 2R-MYBs and 3R-MYBs, as there is a debate between “loss” and “gain” models^8^,^47^. The “loss” model suggests that ancestral 3R-MYBs were generated by successive intragenic domain duplications in primitive eukaryotes, and that 2R-MYBs were formed by the loss of R1 repeats from 3R-MYBs in the plant lineage^8^. In contrast, the “gain” model argues that ancestral 2R-MYBs were derived from an intragenic domain duplication, and that 3R-MYBs were produced by a subsequent intragenic domain duplication from 2R-MYBs^47^. Owing to the progress in the phylogeny of eukaryotes^48^,^49^,^50^,^51^ and the identification of typical 2R-MYBs in non-plant species^17^,^52^,^53^,^54^,^55^, evidence for the loss model is no longer compelling. For instance, it was reported that 3R-MYBs are older than 2R-MYBs because of the observation that 3R-MYBs are widely distributed in eukaryotes, whereas typical 2R-MYBs are regarded as plant-specific^25^,^36^. However, based on the substantial genome sequences available, we investigated the distribution of the 2R-MYB and 3R-MYB gene family across 50 major lineages of eukaryotes. Our results showed that both MYBs co-existed in many eukaryotes, but that typical 2R-MYBs were encoded in a broader range of eukaryotes and in some primitive eukaryotes (Fig. 1).

**Figure 6:**
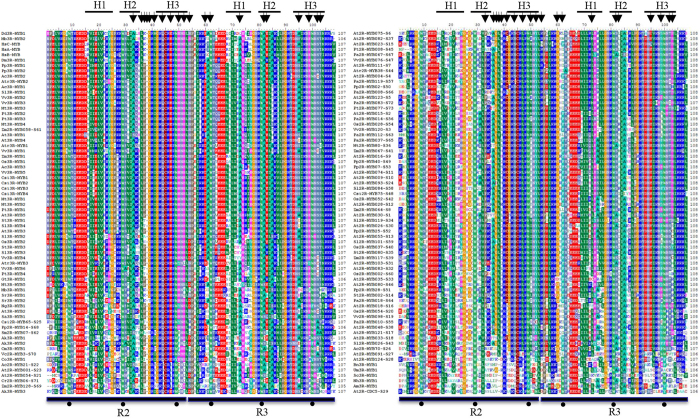
Multiple alignments of R2R3 MYB domains of the representatives of 73 2R-MYB subfamilies and 63 eukaryotes 3R-MYBs. The three α-helices in each repeat are indicated by a black bold line and marked as H1 to H3. The black arrows indicate the conserved residue changes between the typical plant 2R-MYBs and the early-deriving plant 2R-MYBs and eukaryote 3R-MYBs. The asterisks indicate the three highly conserved tryptophan residues (W) in each MYB repeat.

In the current study, we performed an alignment analysis of the R2R3 domains of the representatives of the 73 2R-MYB subfamilies and on all eukaryote 3R-MYBs. Our findings showed that R2R3 MYB domains of 3R-MYBs shared a high degree of sequence similarity with old 2R-MYB subfamilies, which included early diverged land plant members, such as S22, S23, and S25 (Fig. 6). Similar to previous studies, our results showed that 2R-MYBs and 3R-MYBs had up to 20 sites with highly conserved residues, most of which were distributed in the third helix region of the R2 repeat (Fig. 6). Interestingly, we observed that the residues in the corresponding sites of the early-diverged 2R-MYB subfamilies were generally consistent with those of 3R-MYBs. For example, it has been reported that Cys-41 (C) in the R2 repeat is highly conserved in typical 2R-MYBs during evolution, and that it is essential for the DNA-binding activity of the 2R-MYB gene^56^. We found that the residue was C in typical 2R-MYBs, but was Ile (I) or Ser (S) in 3R-MYBs and older subfamilies of 2R-MYBs. Similarly, the first conserved Trp (W) residue in the R3 repeat is generally Phe (F) or I in typical 2R-MYBs; however, we found that it was highly conserved as W in the 3R-MYBs and members of older 2R-MYB subfamilies. Taken together, the similarity in 3R-MYB and 2R-MYB sequences suggests that their origins are strongly correlated.

**Figure 7:**
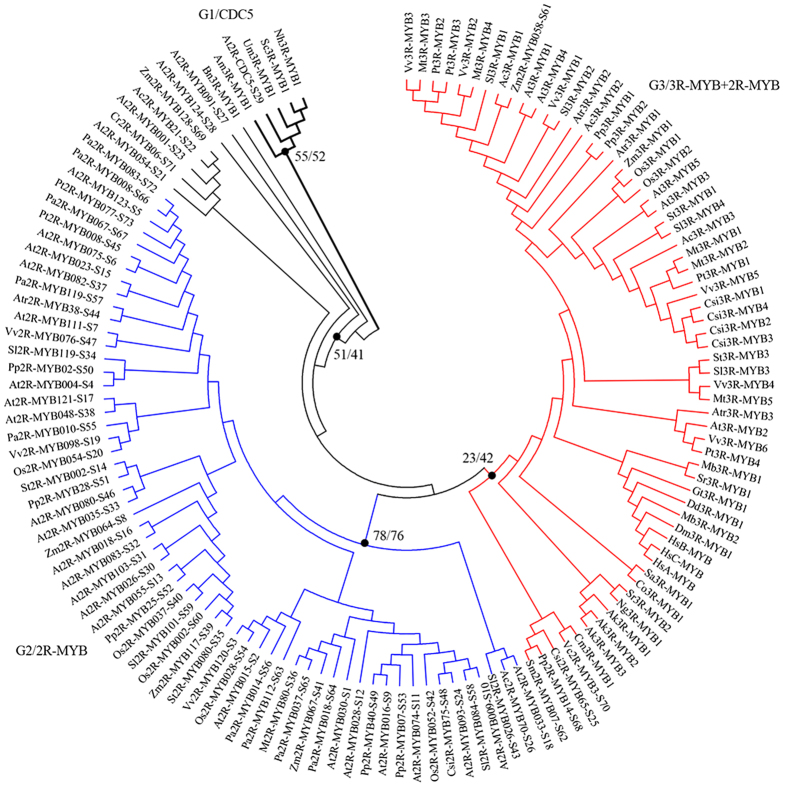
A ML tree of the R2 and R3 MYB repeats of the representatives of 2R-MYB and 3R-MYB proteins. The maximum likelihood (ML) tree represents relationships among 73 representatives of 2R-MYBs and 63 eukaryotes 3R-MYBs. Numbers beside the branches represent bootstrap support values (>50%) from 100 replications in the ML analysis and 1000 replications in the Neighbor-joining (NJ) analysis.

To further explore the origin of 2R-MYBs, we constructed phylogenetic trees using the R2R3 domains of representatives of the 73 2R-MYB subfamilies and all of the eukaryote 3R-MYBs. Given its similar sequence to typical 2R-MYBs, as well as the evolutionary conservation among eukaryotic organisms, atypical 2R-MYBs were utilized as an out-group for the following analysis. Our phylogenetic trees were highly consistent with the results of the gain model^47^, where fungus 3R-MYBs, together with CDC5-like proteins, comprised an out-group. The remaining proteins (including 2R-MYBs and 3R-MYBs) clustered in another large clade (Fig. 7). There was no doubt that the majority of the 2R-MYB or 3R-MYB representatives were generally clustered in a given clade. However, our results showed that the two representatives of the S27 and S28 subfamilies were more homologous to CDC5-like proteins (Fig. 6), which were not clustered in the 2R-MYB clade but were closer to CDC5-like proteins in the phylogenetic trees (Fig. 7). This may indicate that 2R-MYBs, rather than 3R-MYBs, are more homologous to CDC5-like proteins. More interestingly, the older 2R-MYB subfamilies with early-derived plant members (S21, S22, S23, S25, S62, S68, S69, and S71) were not clustered in the major 2R-MYB clade, but were instead clustered in the 3R-MYB clade. However, bootstrap values were somewhat lower because of sequence divergences after the split. This finding indicates a relatively higher evolutionary relationship between these 2R-MYB subfamilies and 3R-MYBs. It is worth noting that the representative of subfamily S61 was more homologous to 3R-MYBs and clustered in the 3R-MYB clade with high a support value (Figs 6,7). Moreover, we observed that the intron pattern in the R2R3 repeats of these 3R-MYBs was consistent with those of members of the S61 subfamily. This finding highly supports the evolutionary relationship of these two types of MYBs.

Given that 2R-MYBs were widely distributed in eukaryotes (even broader than 3R-MYBs), and that they exhibited similar sequence characteristics and/or a closer evolutionary relationship to early-derived land plant 2R-MYB subfamilies and 3R-MYBs, we suggest that 2R-MYBs are older than 3R-MYBs. Further, we propose that 3R-MYBs may be evolutionarily derived from 2R-MYBs via intragenic domain duplication. Overall, our results strongly indicate that the gain model provides a more parsimonious and reasonable explanation for the phylogenetic distribution of these two types of MYBs^47^. In other words, we believe that ancient 2R-MYBs and 3R-MYBs coexisted in primitive eukaryotes, and gave rise to the currently extant plant 2R-MYB and 3R-MYB genes, respectively.

As shown in Fig. 2, the current phylogenies allow for the delimitation of major evolutionary lineages of eukaryotic 2R-MYBs. However, this tree could not completely resolve phylogenetic relationships of all subfamilies, as the basal nodes often received low support values due to the large gene number used. Therefore, to address the evolutionary relationships of each subfamily, we constructed a Bayesian phylogenetic tree using representatives of the 73 subfamilies and of the orphan genes (Supplementary Fig. S3 online).

As discussed above, the MYB-related CDC5-like proteins are older, and were accordingly appropriate as an out-group for our phylogenetic analyses. In the Bayesian (BA) tree, the topology was essentially the same as it was with the NJ and ML trees. All representative sequences from both plant and non-plant eukaryotic organisms formed a monophyletic clade directly ancestral to the CDC5-like gene (Supplementary Fig. S3 online). As expected, the BA tree improved the deep nodes of the 2R-MYB tree with high support values, which elucidated the deep evolutionary relationships of the 2R-MYBs. Together with the results of the sequence characteristic analyses (discussed above), the findings of this analysis further supported the common origin of plant 2R-MYBs. Moreover, our phylogenetic analysis also revealed evolutionary relationships of the orphan genes, and demonstrated the high conservation of this gene family across the eukaryotic kingdom. Interestingly, subfamily S21, S22, S23, S25, and S28 representatives tended to cluster at the base of the tree, sister to the ancestral subfamily, S29, with high support values. The rest of the representative sequences were clustered into a more highly supported clade, which is highly consistent with the results throughout this study regarding the origin and expansion of plant 2R-MYBs. Altogether, our results support the hypothesis that the plant 2R-MYBs are monophyletic^13^. Furthermore, these findings confirm that the diversity of 2R-MYBs was mainly established in the common ancestor of land plants, from which all modern plant 2R-MYBs descended and radiated by the mechanisms discussed above.

## Methods

### Sequence retrieval of 2R-MYB-encoding genes in eukaryotes

To identify potential 2R-MYB homologs in the genomes of major eukaryotes, we first conducted BLASTP searches against the plant genome sequences of the chlorophyte, lycophyte, gymnosperm, basal magnoliophyta, monocot, and asterid, as well as eudicots, moss, eurosids I and II in Phytozome v9.1 and/or v10 (http://www.phytozome.net/), NCBI, JGI, UniProt databases, and BROAD (http://www.broadinstitute.org/annotation/genome/multicellularity_project/MultiHome.html), respectively. To ensure that no 2R-MYB proteins were eliminated by lack of correspondence to the consensus, we used the representative sequences of At2R-MYB proteins as queries (Supplementary Table S5), with a low-stringency criterion (cut-off p-value <1E-1). Each matching sequence was then used to search the respective genome database, until no new sequences were found. Subsequently, all candidate proteins were manually checked to eliminate partial and redundant sequences. To further ensure that the candidate proteins contained two or three MYB repeats, putative sequences were examined for the MYB domain using the SMART (http://smart.embl-heidelberg.de/) and ExPASy (The PROSITE profile PS50090 was used, http://expasy.org/prosite/) tools. The candidates were considered as positive hits based on the following three additional criteria: MYB repeats were adjacent to each other; the MYB domain had significant sequence similarity to c-MYB-like proteins; and each MYB repeat contained at least two of the three highly conserved Trp (W) residues. In the present study, we applied the same procedure and criteria to identify 2R-MYBs in rice (v7.0), grapes (Genoscope.12X), and *Populus* (v3.0). Finally, all newly identified sequences were named according to protein identifiers from the corresponding genome browsers (Supplementary Table S1).

Similarly, to further address the distribution of 2R-MYBs in other eukaryotes, we extended our dataset to include a full set of genes encoded in the genomes of non-plant bikonts and unikonts by searching NCBI, JGI, and UniProt protein databases. Representative genomes included red alga, Cryptophyta, Haptophyta, Rhizaria, Stramenopiles, Alveolata, excavate, Amoebozoa, Apusozoa, fungi, Ichthyosporea, Filasterea, Choanoflagellata, and metazoans (Fig. 1).

The newly identified 2R-MYB sequences, together with the previously identified sequences of *Arabidopsis thaliana*^9^ and *Zea mays* (maize)^15^, generated a preliminary dataset that was used in this study (Fig. 1, Supplementary Table S1).

### Sequence alignment and phylogenetic analysis

Multiple alignments of the 1548 candidate 2R-MYB domains were performed by MAFFT version 7 (http://mafft.cbrc.jp/alignment/server/) with default options, which were then extensively checked by BioEdit (Pittsburgh Supercomputing Center; http://www.psc.edu/biomed/genedoc/).

The MEGA version 6.0^57^ was used to perform a neighbor joining (NJ) tree analysis. For statistical reliability, the following parameters were applied: 1000 replicas, p-distance model, and pairwise deletion. The program ProtTest was used to estimate the best model for each alignment by default^58^. ProtTest indicated the JJT amino substitution model with estimation of the gamma distribution shape parameter (JJT + G) as the best fit of the 112 examined evolutionary models, according to Akaike information criterion (AIC) statistics. A maximum likelihood (ML) tree was constructed using MEGA v6.0, with 100 replicas and the JJT + G model. Bayesian analysis was conducted with MrBayes version 3.2^59^, with the mixed model, two independent runs of four simultaneous chains for 30,000,000 generations, and sampling every 10,000 generations. Convergence was assessed by comparing the standard deviation (SD) of split frequencies between runs. Trees were visualized using the program, Figtree (http://tree.bio.ed.ac.uk/software/figtree/).

### Identification of conserved motifs

The MEME program was used to predict conserved motifs outside of MYB domains (http://meme.sdsc.edu). The maximum number of motifs was set to 100, the optimum motif width was constrained to between 6 and 300 residues to make sure MYB domains and/or other long non-MYB motifs were included, and other factors were of default selection. All putative motifs with expected values of >1E-30 were discarded.

### Genome synteny and gene duplication

Syntenic blocks among the corresponding species of land plants were downloaded from the Plant Genome Duplication Database^60^. All 2R-MYBs were then mapped to the syntenic blocks for intra- and intergenomic comparison. Tandem duplication genes were identified based on their physical locations within individual chromosomes, with no more than one intervening gene.

## Additional Information

**How to cite this article**: Du, H. *et al.* The Evolutionary History of R2R3-MYB Proteins Across 50 Eukaryotes: New Insights into Subfamily Classification and Expansion. *Sci. Rep.* **5**, 11037; doi: 10.1038/srep11037 (2015).

## Supplementary Material

Supplementary Information

## Figures and Tables

**Table 1:**
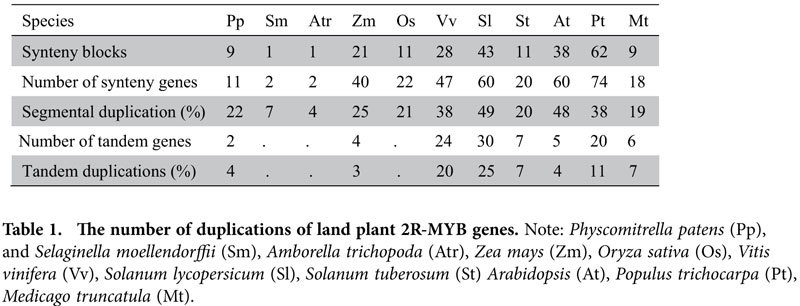
The number of duplications of land plant 2R-MYB genes.
