# Supplementary material for: The Evolutionary History of R2R3-MYB Proteins Across 50 Eukaryotes: New Insights Into Subfamily Classification and Expansion
Source: Sci Rep. 2015 Jun 5;5:11037. doi: 10.1038/srep11037 (PMC4603784; doi:10.1038/srep11037)
Supplement: Supplementary Information [file srep11037-s1.pdf]

Supplementary information for

The Evolutionary History of R2R3-MYB Proteins Across 50 Eukaryotes: New Insights into Subfamily Classification and Expansion

Hai Du[1], Zhe Liang[3], Sen Zhao[4,5], Ming-Ge Nan[1], Lam-Son Phan Tran[6], Kun Lu[1],Yu-Bi Huang[2*], and Jia-Na Li[1*]

[1]College of Agronomy and Biotechnology, Southwest University, Chongqing, 400716, China; [2]Key Laboratory of Biology and Genetic Improvement of Maize in Southwest Region, Maize Research Institute of Sichuan Agricultural University, Ministry of Agriculture, Chengdu Sichuan, China; [3]Department of Biological Sciences, National University of Singapore, 117543, Singapore; [4]Department of Cancer Prevention, Institute for Cancer Research, Norwegian Radium Hospital-Oslo University Hospital, Oslo, Norway; [5]Centre for Cancer Biomedicine, Faculty of Medicine, University of Oslo, Oslo, Norway; [6] Signaling Pathway Research Unit, RIKEN Center for Sustainable Resource Science, #E610 6F East Research Building, 1-7-22 Suehiro, Tsurumi, Yokohama, Kanagawa 230-0045 Japan.

*To whom correspondence should be addressed. Email: yubihuang@sohu.com; ljn1950@swu.edu.cn

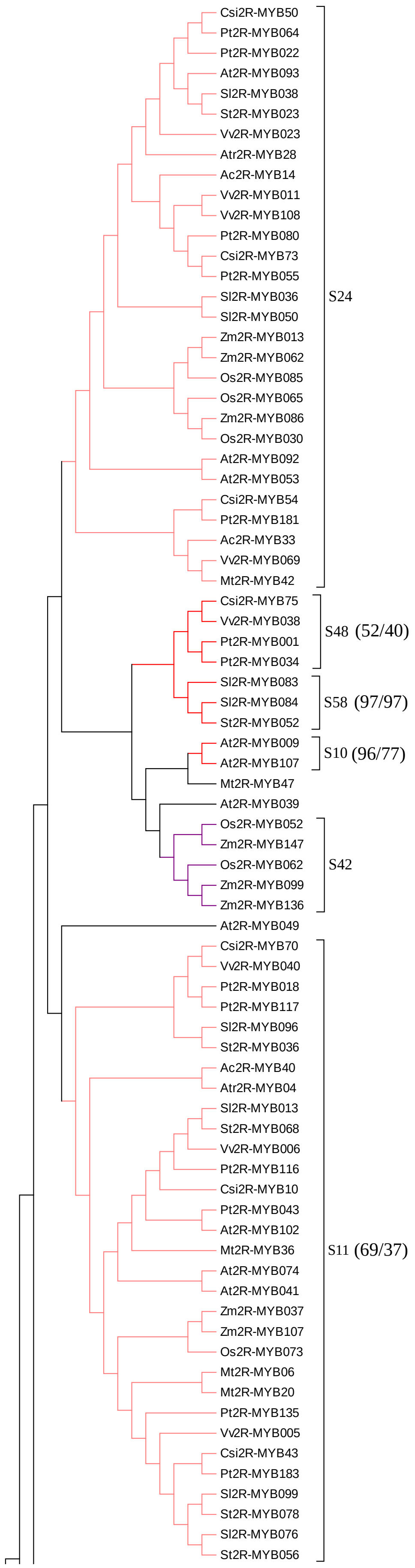
Csi2R-MYB50
Pt2R-MYB064
Pt2R-MYB022
At2R-MYB093
Sl2R-MYB038
St2R-MYB023
Vv2R-MYB023
Atr2R-MYB28
Ac2R-MYB14
Vv2R-MYB011
Vv2R-MYB108
Pt2R-MYB080
Csi2R-MYB73
Pt2R-MYB055
Sl2R-MYB036
Sl2R-MYB050
Zm2R-MYB013
Zm2R-MYB062
Os2R-MYB085
Os2R-MYB065
Zm2R-MYB086
Os2R-MYB030
At2R-MYB092
At2R-MYB053
Csi2R-MYB54
Pt2R-MYB181
Ac2R-MYB33
Vv2R-MYB069
Mt2R-MYB42
S24
Csi2R-MYB75
Vv2R-MYB038
Pt2R-MYB001
Pt2R-MYB034
S48 (52/40)
Sl2R-MYB083
Sl2R-MYB084
St2R-MYB052
S58 (97/97)
At2R-MYB009
At2R-MYB107
S10 (96/77)
Mt2R-MYB47
At2R-MYB039
Os2R-MYB052
Zm2R-MYB147
Os2R-MYB062
Zm2R-MYB099
Zm2R-MYB136
S42
At2R-MYB049
Csi2R-MYB70
Vv2R-MYB040
Pt2R-MYB018
Pt2R-MYB117
Sl2R-MYB096
St2R-MYB036
Ac2R-MYB40
Atr2R-MYB04
Sl2R-MYB013
St2R-MYB068
Vv2R-MYB006
Pt2R-MYB116
Csi2R-MYB10
Pt2R-MYB043
At2R-MYB102
Mt2R-MYB36
At2R-MYB074
At2R-MYB041
Zm2R-MYB037
Zm2R-MYB107
Os2R-MYB073
Mt2R-MYB06
Mt2R-MYB20
Pt2R-MYB135
Vv2R-MYB005
Csi2R-MYB43
Pt2R-MYB183
Sl2R-MYB099
St2R-MYB078
Sl2R-MYB076
St2R-MYB056
S11 (69/37)

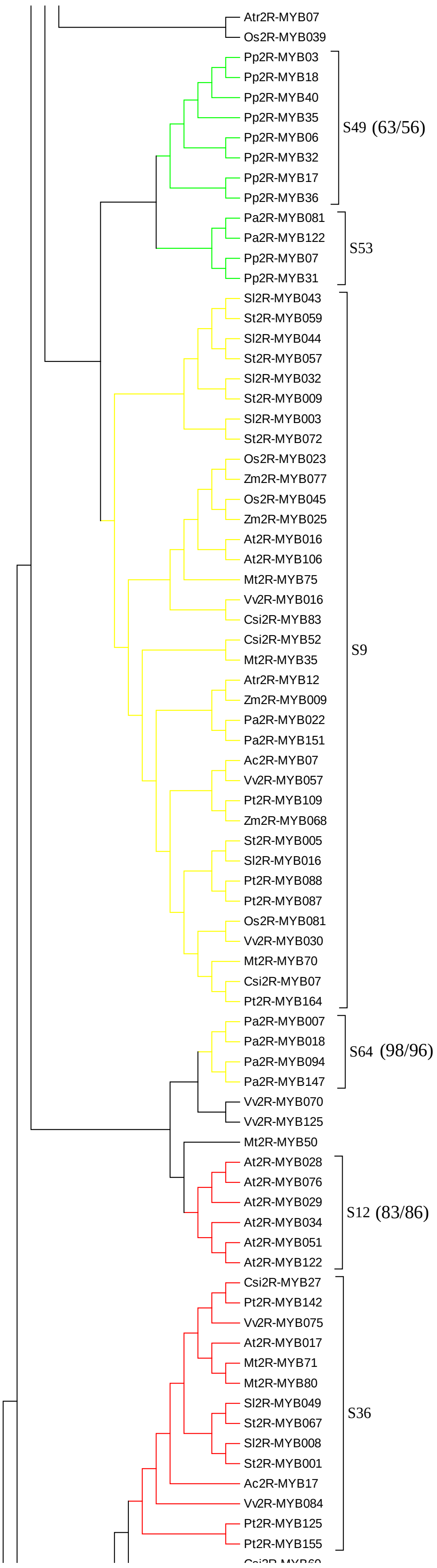
Atr2R-MYB07
Os2R-MYB039
Pp2R-MYB03
Pp2R-MYB18
Pp2R-MYB40
Pp2R-MYB35
Pp2R-MYB06
Pp2R-MYB32
Pp2R-MYB17
Pp2R-MYB36
S49 (63/56)
Pa2R-MYB081
Pa2R-MYB122
Pp2R-MYB07
Pp2R-MYB31
S53
Sl2R-MYB043
St2R-MYB059
Sl2R-MYB044
St2R-MYB057
Sl2R-MYB032
St2R-MYB009
Sl2R-MYB003
St2R-MYB072
Os2R-MYB023
Zm2R-MYB077
Os2R-MYB045
Zm2R-MYB025
At2R-MYB016
At2R-MYB106
Mt2R-MYB75
Vv2R-MYB016
Csi2R-MYB83
Csi2R-MYB52
Mt2R-MYB35
Atr2R-MYB12
Zm2R-MYB009
Pa2R-MYB022
Pa2R-MYB151
Ac2R-MYB07
Vv2R-MYB057
Pt2R-MYB109
Zm2R-MYB068
St2R-MYB005
Sl2R-MYB016
Pt2R-MYB088
Pt2R-MYB087
Os2R-MYB081
Vv2R-MYB030
Mt2R-MYB70
Csi2R-MYB07
Pt2R-MYB164
S9
Pa2R-MYB007
Pa2R-MYB018
Pa2R-MYB094
Pa2R-MYB147
S64 (98/96)
Vv2R-MYB070
Vv2R-MYB125
Mt2R-MYB50
At2R-MYB028
At2R-MYB076
At2R-MYB029
At2R-MYB034
At2R-MYB051
At2R-MYB122
S12 (83/86)
Csi2R-MYB27
Pt2R-MYB142
Vv2R-MYB075
At2R-MYB017
Mt2R-MYB71
Mt2R-MYB80
Sl2R-MYB049
St2R-MYB067
Sl2R-MYB008
St2R-MYB001
Ac2R-MYB17
Vv2R-MYB084
Pt2R-MYB125
Pt2R-MYB155
S36

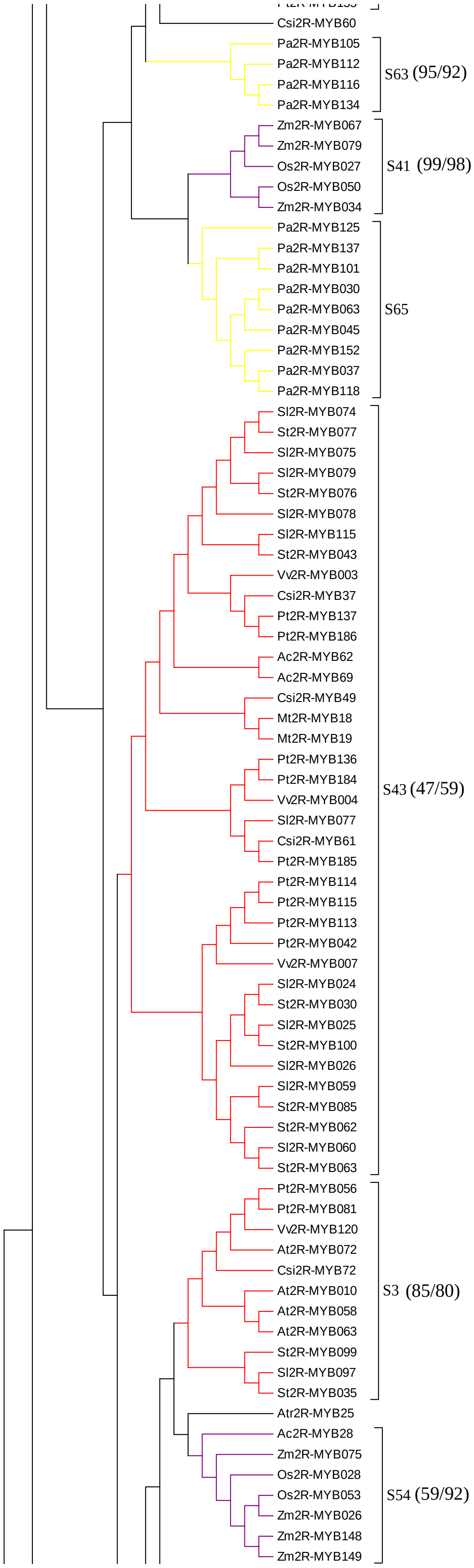
Csi2R-MYB60
Pa2R-MYB105
Pa2R-MYB112
Pa2R-MYB116
Pa2R-MYB134
S63 (95/92)
Zm2R-MYB067
Zm2R-MYB079
Os2R-MYB027
Os2R-MYB050
Zm2R-MYB034
S41 (99/98)
Pa2R-MYB125
Pa2R-MYB137
Pa2R-MYB101
Pa2R-MYB030
Pa2R-MYB063
Pa2R-MYB045
Pa2R-MYB152
Pa2R-MYB037
Pa2R-MYB118
S65
Sl2R-MYB074
St2R-MYB077
Sl2R-MYB075
Sl2R-MYB079
St2R-MYB076
Sl2R-MYB078
Sl2R-MYB115
St2R-MYB043
Vv2R-MYB003
Csi2R-MYB37
Pt2R-MYB137
Pt2R-MYB186
Ac2R-MYB62
Ac2R-MYB69
Csi2R-MYB49
Mt2R-MYB18
Mt2R-MYB19
Pt2R-MYB136
Pt2R-MYB184
Vv2R-MYB004
Sl2R-MYB077
Csi2R-MYB61
Pt2R-MYB185
Pt2R-MYB114
Pt2R-MYB115
Pt2R-MYB113
Pt2R-MYB042
Vv2R-MYB007
Sl2R-MYB024
St2R-MYB030
Sl2R-MYB025
St2R-MYB100
Sl2R-MYB026
Sl2R-MYB059
St2R-MYB085
St2R-MYB062
Sl2R-MYB060
St2R-MYB063
S43 (47/59)
Pt2R-MYB056
Pt2R-MYB081
Vv2R-MYB120
At2R-MYB072
Csi2R-MYB72
At2R-MYB010
At2R-MYB058
At2R-MYB063
St2R-MYB099
Sl2R-MYB097
St2R-MYB035
S3 (85/80)
Atr2R-MYB25
Ac2R-MYB28
Zm2R-MYB075
Os2R-MYB028
Os2R-MYB053
Zm2R-MYB026
Zm2R-MYB148
Zm2R-MYB149
S54 (59/92)

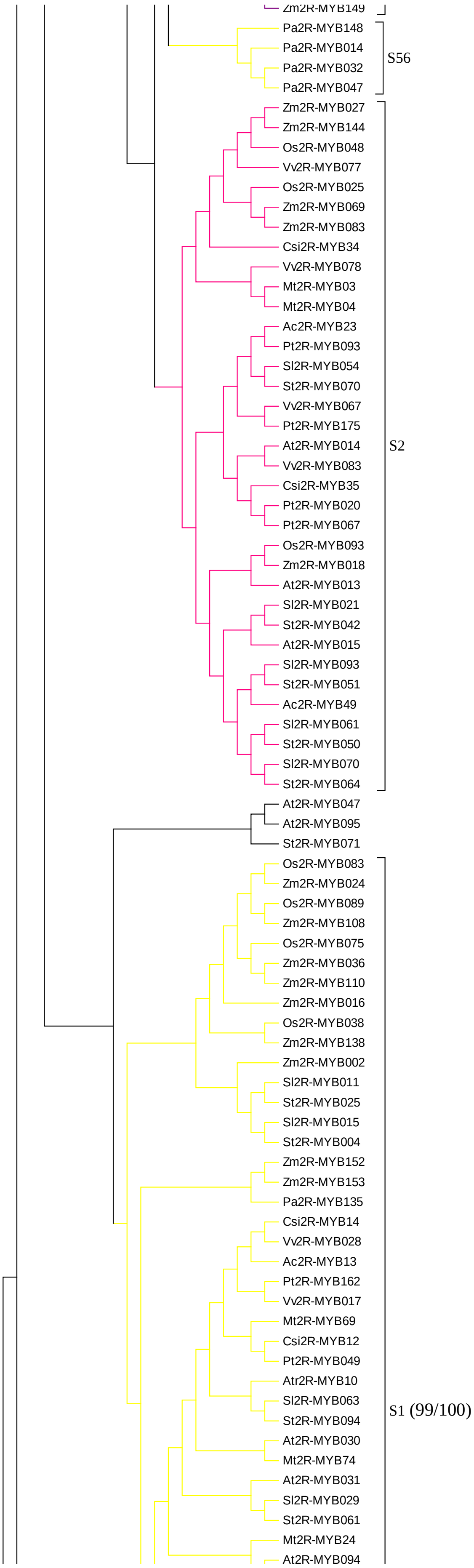
Zm2R-MYB149
Pa2R-MYB148
Pa2R-MYB014
Pa2R-MYB032
Pa2R-MYB047
S56
Zm2R-MYB027
Zm2R-MYB144
Os2R-MYB048
Vv2R-MYB077
Os2R-MYB025
Zm2R-MYB069
Zm2R-MYB083
Csi2R-MYB34
Vv2R-MYB078
Mt2R-MYB03
Mt2R-MYB04
Ac2R-MYB23
Pt2R-MYB093
Sl2R-MYB054
St2R-MYB070
Vv2R-MYB067
Pt2R-MYB175
At2R-MYB014
Vv2R-MYB083
Csi2R-MYB35
Pt2R-MYB020
Pt2R-MYB067
Os2R-MYB093
Zm2R-MYB018
At2R-MYB013
Sl2R-MYB021
St2R-MYB042
At2R-MYB015
Sl2R-MYB093
St2R-MYB051
Ac2R-MYB49
Sl2R-MYB061
St2R-MYB050
Sl2R-MYB070
St2R-MYB064
S2
At2R-MYB047
At2R-MYB095
St2R-MYB071
Os2R-MYB083
Zm2R-MYB024
Os2R-MYB089
Zm2R-MYB108
Os2R-MYB075
Zm2R-MYB036
Zm2R-MYB110
Zm2R-MYB016
Os2R-MYB038
Zm2R-MYB138
Zm2R-MYB002
Sl2R-MYB011
St2R-MYB025
Sl2R-MYB015
St2R-MYB004
Zm2R-MYB152
Zm2R-MYB153
Pa2R-MYB135
Csi2R-MYB14
Vv2R-MYB028
Ac2R-MYB13
Pt2R-MYB162
Vv2R-MYB017
Mt2R-MYB69
Csi2R-MYB12
Pt2R-MYB049
Atr2R-MYB10
Sl2R-MYB063
St2R-MYB094
At2R-MYB030
Mt2R-MYB74
At2R-MYB031
Sl2R-MYB029
St2R-MYB061
Mt2R-MYB24
At2R-MYB094
S1 (99/100)

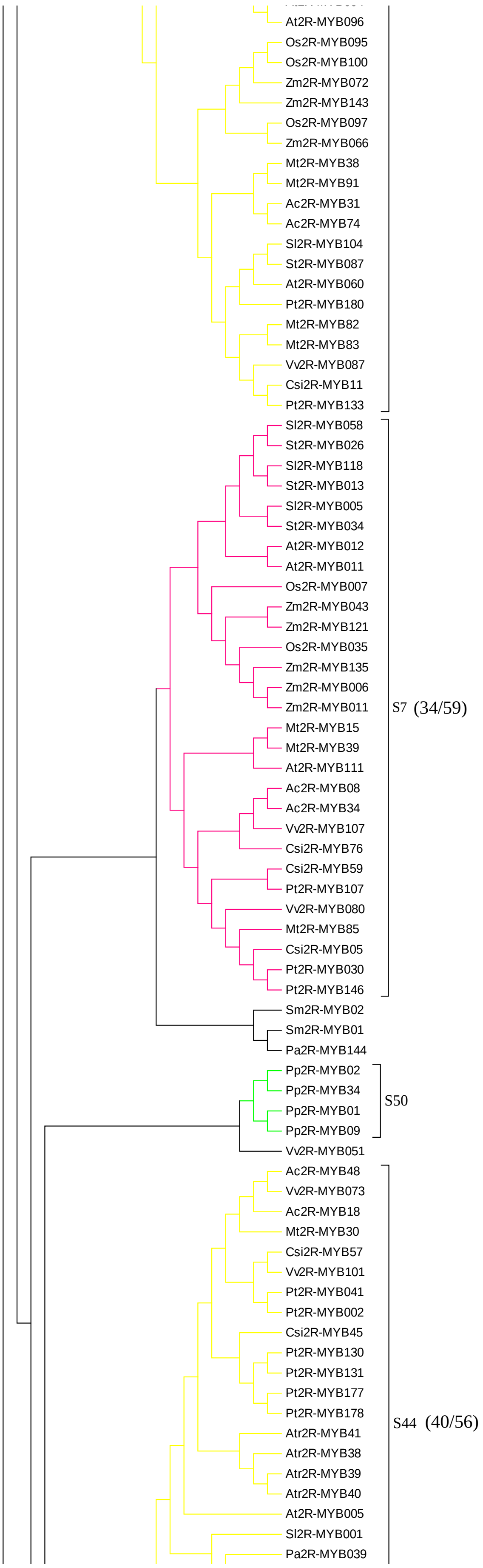
At2R-MYB096
Os2R-MYB095
Os2R-MYB100
Zm2R-MYB072
Zm2R-MYB143
Os2R-MYB097
Zm2R-MYB066
Mt2R-MYB38
Mt2R-MYB91
Ac2R-MYB31
Ac2R-MYB74
Sl2R-MYB104
St2R-MYB087
At2R-MYB060
Pt2R-MYB180
Mt2R-MYB82
Mt2R-MYB83
Vv2R-MYB087
Csi2R-MYB11
Pt2R-MYB133
Sl2R-MYB058
St2R-MYB026
Sl2R-MYB118
St2R-MYB013
Sl2R-MYB005
St2R-MYB034
At2R-MYB012
At2R-MYB011
Os2R-MYB007
Zm2R-MYB043
Zm2R-MYB121
Os2R-MYB035
Zm2R-MYB135
Zm2R-MYB006
Zm2R-MYB011
S7 (34/59)
Mt2R-MYB15
Mt2R-MYB39
At2R-MYB111
Ac2R-MYB08
Ac2R-MYB34
Vv2R-MYB107
Csi2R-MYB76
Csi2R-MYB59
Pt2R-MYB107
Vv2R-MYB080
Mt2R-MYB85
Csi2R-MYB05
Pt2R-MYB030
Pt2R-MYB146
Sm2R-MYB02
Sm2R-MYB01
Pa2R-MYB144
Pp2R-MYB02
Pp2R-MYB34
S50
Pp2R-MYB01
Pp2R-MYB09
Vv2R-MYB051
Ac2R-MYB48
Vv2R-MYB073
Ac2R-MYB18
Mt2R-MYB30
Csi2R-MYB57
Vv2R-MYB101
Pt2R-MYB041
Pt2R-MYB002
Csi2R-MYB45
Pt2R-MYB130
Pt2R-MYB131
Pt2R-MYB177
Pt2R-MYB178
S44 (40/56)
Atr2R-MYB41
Atr2R-MYB38
Atr2R-MYB39
Atr2R-MYB40
At2R-MYB005
Sl2R-MYB001
Pa2R-MYB039

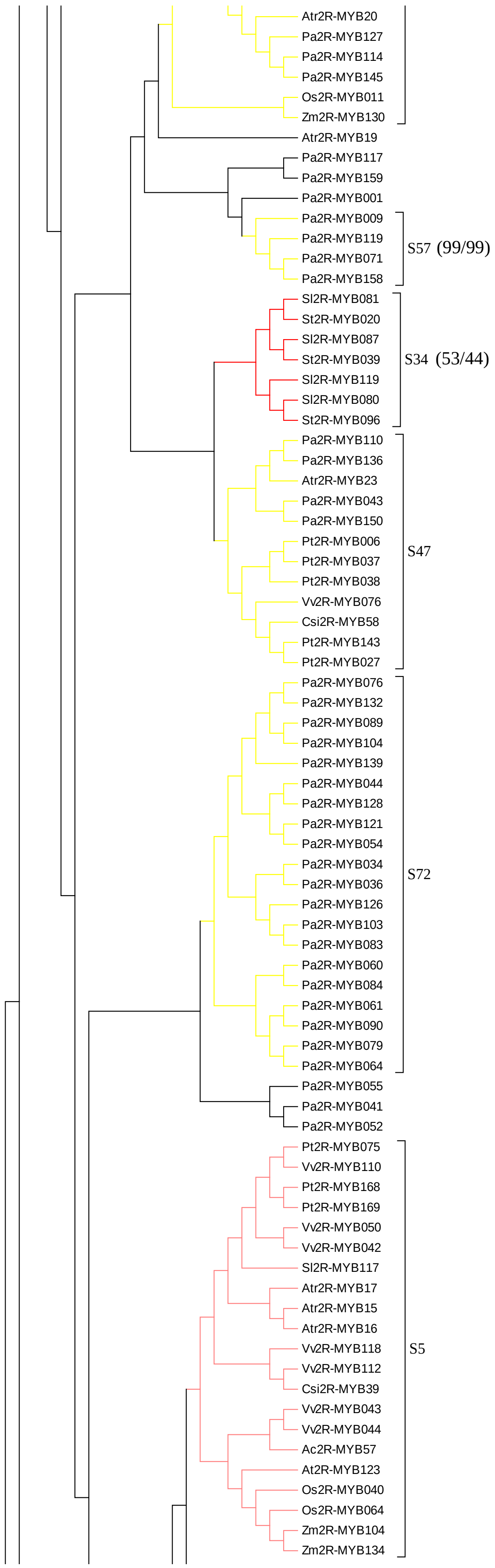

Atr2R-MYB20
Pa2R-MYB127
Pa2R-MYB114
Pa2R-MYB145
Os2R-MYB011
Zm2R-MYB130
Atr2R-MYB19
Pa2R-MYB117
Pa2R-MYB159
Pa2R-MYB001
Pa2R-MYB009
Pa2R-MYB119
Pa2R-MYB071
Pa2R-MYB158
S57 (99/99)
Sl2R-MYB081
St2R-MYB020
Sl2R-MYB087
St2R-MYB039
Sl2R-MYB119
Sl2R-MYB080
St2R-MYB096
S34 (53/44)
Pa2R-MYB110
Pa2R-MYB136
Atr2R-MYB23
Pa2R-MYB043
Pa2R-MYB150
Pt2R-MYB006
Pt2R-MYB037
Pt2R-MYB038
Vv2R-MYB076
Csi2R-MYB58
Pt2R-MYB143
Pt2R-MYB027
S47
Pa2R-MYB076
Pa2R-MYB132
Pa2R-MYB089
Pa2R-MYB104
Pa2R-MYB139
Pa2R-MYB044
Pa2R-MYB128
Pa2R-MYB121
Pa2R-MYB054
Pa2R-MYB034
Pa2R-MYB036
Pa2R-MYB126
Pa2R-MYB103
Pa2R-MYB083
Pa2R-MYB060
Pa2R-MYB084
Pa2R-MYB061
Pa2R-MYB090
Pa2R-MYB079
Pa2R-MYB064
S72
Pa2R-MYB055
Pa2R-MYB041
Pa2R-MYB052
Pt2R-MYB075
Vv2R-MYB110
Pt2R-MYB168
Pt2R-MYB169
Vv2R-MYB050
Vv2R-MYB042
Sl2R-MYB117
Atr2R-MYB17
Atr2R-MYB15
Atr2R-MYB16
Vv2R-MYB118
Vv2R-MYB112
Csi2R-MYB39
Vv2R-MYB043
Vv2R-MYB044
Ac2R-MYB57
At2R-MYB123
Os2R-MYB040
Os2R-MYB064
Zm2R-MYB104
Zm2R-MYB134
S5

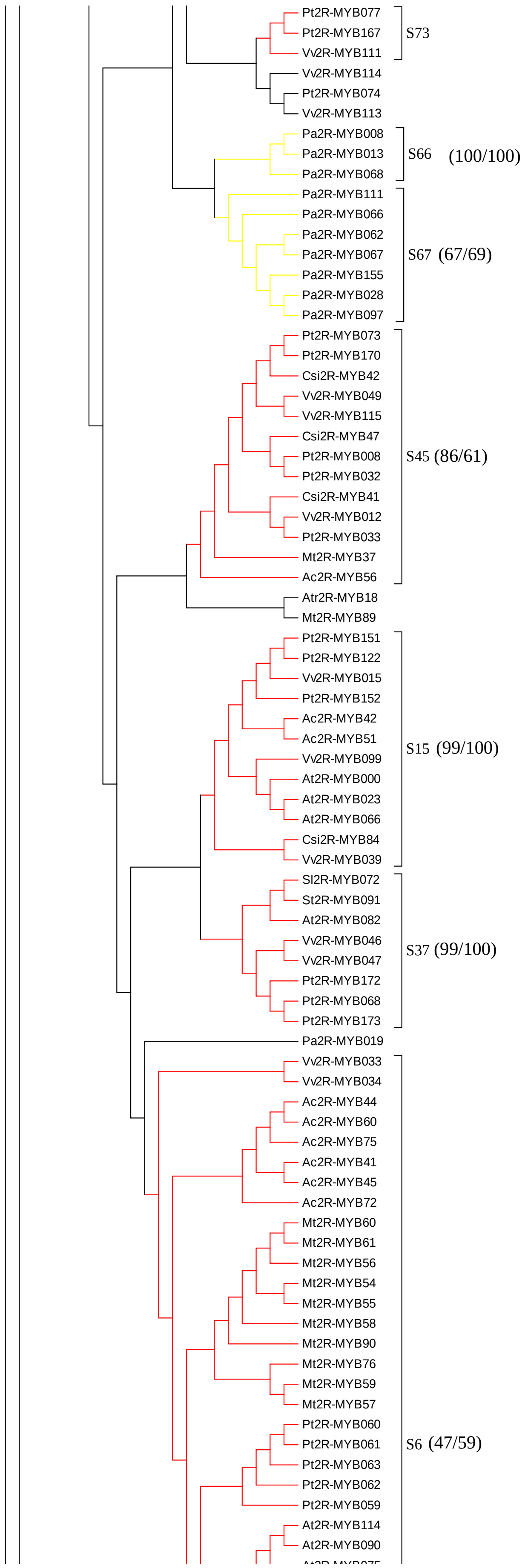
Pt2R-MYB077
Pt2R-MYB167
Vv2R-MYB111
Vv2R-MYB114
Pt2R-MYB074
Vv2R-MYB113
S73
Pa2R-MYB008
Pa2R-MYB013
Pa2R-MYB068
S66 (100/100)
Pa2R-MYB111
Pa2R-MYB066
Pa2R-MYB062
Pa2R-MYB067
Pa2R-MYB155
Pa2R-MYB028
Pa2R-MYB097
S67 (67/69)
Pt2R-MYB073
Pt2R-MYB170
Csi2R-MYB42
Vv2R-MYB049
Vv2R-MYB115
Csi2R-MYB47
Pt2R-MYB008
Pt2R-MYB032
Csi2R-MYB41
Vv2R-MYB012
Pt2R-MYB033
Mt2R-MYB37
Ac2R-MYB56
S45 (86/61)
Atr2R-MYB18
Mt2R-MYB89
Pt2R-MYB151
Pt2R-MYB122
Vv2R-MYB015
Pt2R-MYB152
Ac2R-MYB42
Ac2R-MYB51
Vv2R-MYB099
At2R-MYB000
At2R-MYB023
At2R-MYB066
Csi2R-MYB84
Vv2R-MYB039
S15 (99/100)
Sl2R-MYB072
St2R-MYB091
At2R-MYB082
Vv2R-MYB046
Vv2R-MYB047
Pt2R-MYB172
Pt2R-MYB068
Pt2R-MYB173
S37 (99/100)
Pa2R-MYB019
Vv2R-MYB033
Vv2R-MYB034
Ac2R-MYB44
Ac2R-MYB60
Ac2R-MYB75
Ac2R-MYB41
Ac2R-MYB45
Ac2R-MYB72
Mt2R-MYB60
Mt2R-MYB61
Mt2R-MYB56
Mt2R-MYB54
Mt2R-MYB55
Mt2R-MYB58
Mt2R-MYB90
Mt2R-MYB76
Mt2R-MYB59
Mt2R-MYB57
Pt2R-MYB060
Pt2R-MYB061
Pt2R-MYB063
Pt2R-MYB062
Pt2R-MYB059
At2R-MYB114
At2R-MYB090
S6 (47/59)

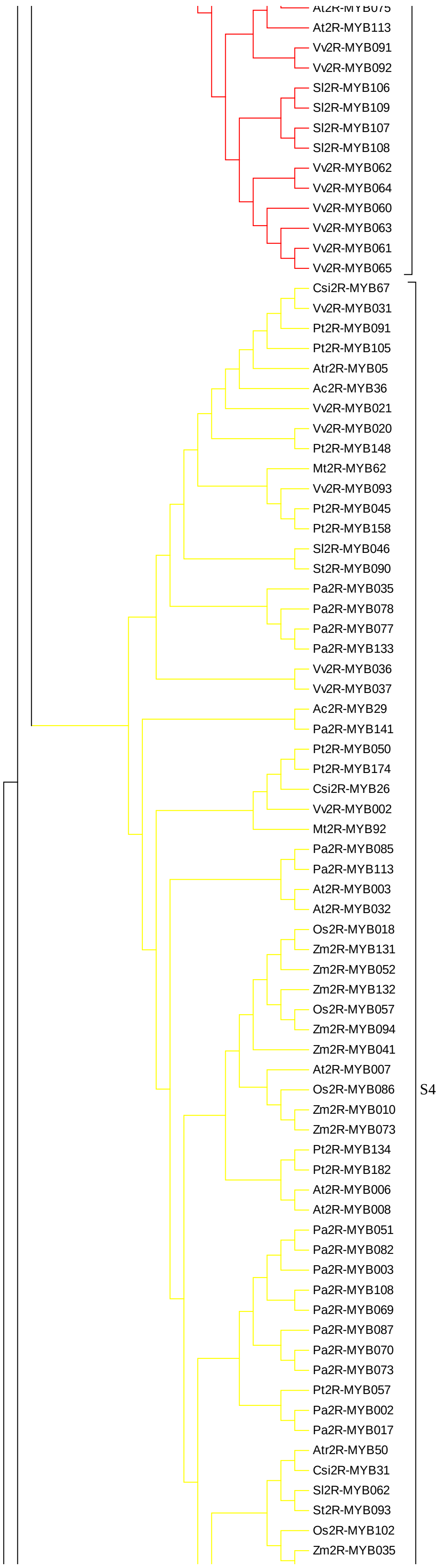
At2R-MYB113
Vv2R-MYB091
Vv2R-MYB092
Sl2R-MYB106
Sl2R-MYB109
Sl2R-MYB107
Sl2R-MYB108
Vv2R-MYB062
Vv2R-MYB064
Vv2R-MYB060
Vv2R-MYB063
Vv2R-MYB061
Vv2R-MYB065
Csi2R-MYB67
Vv2R-MYB031
Pt2R-MYB091
Pt2R-MYB105
Atr2R-MYB05
Ac2R-MYB36
Vv2R-MYB021
Vv2R-MYB020
Pt2R-MYB148
Mt2R-MYB62
Vv2R-MYB093
Pt2R-MYB045
Pt2R-MYB158
Sl2R-MYB046
St2R-MYB090
Pa2R-MYB035
Pa2R-MYB078
Pa2R-MYB077
Pa2R-MYB133
Vv2R-MYB036
Vv2R-MYB037
Ac2R-MYB29
Pa2R-MYB141
Pt2R-MYB050
Pt2R-MYB174
Csi2R-MYB26
Vv2R-MYB002
Mt2R-MYB92
Pa2R-MYB085
Pa2R-MYB113
At2R-MYB003
At2R-MYB032
Os2R-MYB018
Zm2R-MYB131
Zm2R-MYB052
Zm2R-MYB132
Os2R-MYB057
Zm2R-MYB094
Zm2R-MYB041
At2R-MYB007
Os2R-MYB086
Zm2R-MYB010
Zm2R-MYB073
Pt2R-MYB134
Pt2R-MYB182
At2R-MYB006
At2R-MYB008
Pa2R-MYB051
Pa2R-MYB082
Pa2R-MYB003
Pa2R-MYB108
Pa2R-MYB069
Pa2R-MYB087
Pa2R-MYB070
Pa2R-MYB073
Pt2R-MYB057
Pa2R-MYB002
Pa2R-MYB017
Atr2R-MYB50
Csi2R-MYB31
Sl2R-MYB062
St2R-MYB093
Os2R-MYB102
Zm2R-MYB035
S4

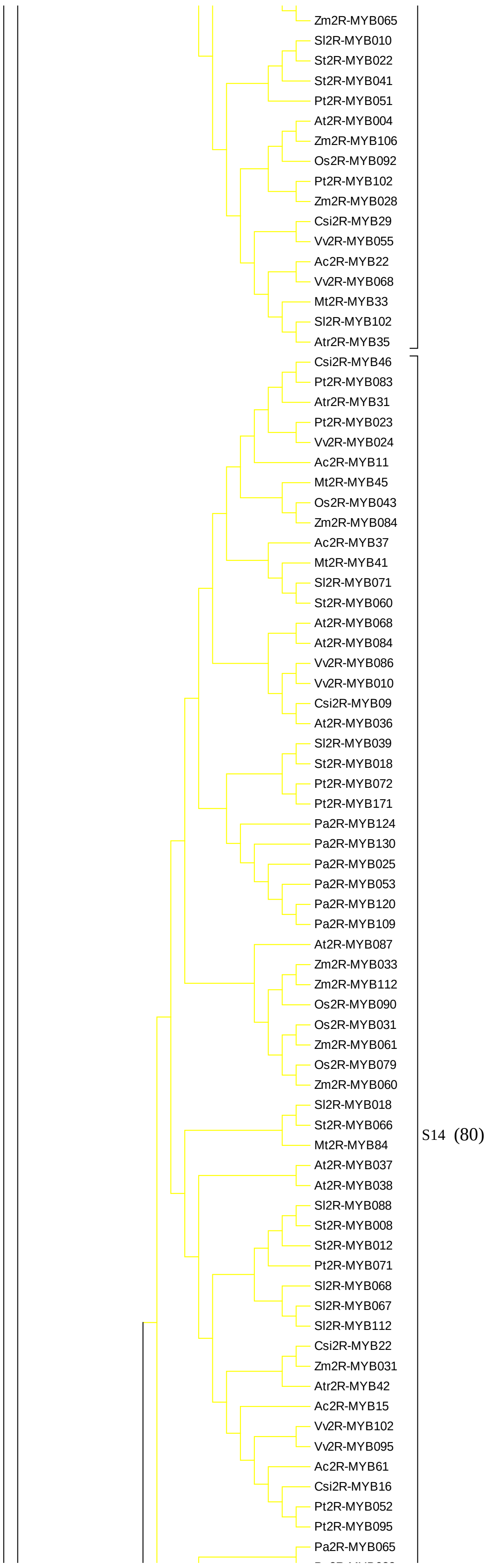
Zm2R-MYB065
Sl2R-MYB010
St2R-MYB022
St2R-MYB041
Pt2R-MYB051
At2R-MYB004
Zm2R-MYB106
Os2R-MYB092
Pt2R-MYB102
Zm2R-MYB028
Csi2R-MYB29
Vv2R-MYB055
Ac2R-MYB22
Vv2R-MYB068
Mt2R-MYB33
Sl2R-MYB102
Atr2R-MYB35
Csi2R-MYB46
Pt2R-MYB083
Atr2R-MYB31
Pt2R-MYB023
Vv2R-MYB024
Ac2R-MYB11
Mt2R-MYB45
Os2R-MYB043
Zm2R-MYB084
Ac2R-MYB37
Mt2R-MYB41
Sl2R-MYB071
St2R-MYB060
At2R-MYB068
At2R-MYB084
Vv2R-MYB086
Vv2R-MYB010
Csi2R-MYB09
At2R-MYB036
Sl2R-MYB039
St2R-MYB018
Pt2R-MYB072
Pt2R-MYB171
Pa2R-MYB124
Pa2R-MYB130
Pa2R-MYB025
Pa2R-MYB053
Pa2R-MYB120
Pa2R-MYB109
At2R-MYB087
Zm2R-MYB033
Zm2R-MYB112
Os2R-MYB090
Os2R-MYB031
Zm2R-MYB061
Os2R-MYB079
Zm2R-MYB060
Sl2R-MYB018
St2R-MYB066
Mt2R-MYB84
At2R-MYB037
At2R-MYB038
Sl2R-MYB088
St2R-MYB008
St2R-MYB012
Pt2R-MYB071
Sl2R-MYB068
Sl2R-MYB067
Sl2R-MYB112
Csi2R-MYB22
Zm2R-MYB031
Atr2R-MYB42
Ac2R-MYB15
Vv2R-MYB102
Vv2R-MYB095
Ac2R-MYB61
Csi2R-MYB16
Pt2R-MYB052
Pt2R-MYB095
Pa2R-MYB065
S14 (80)

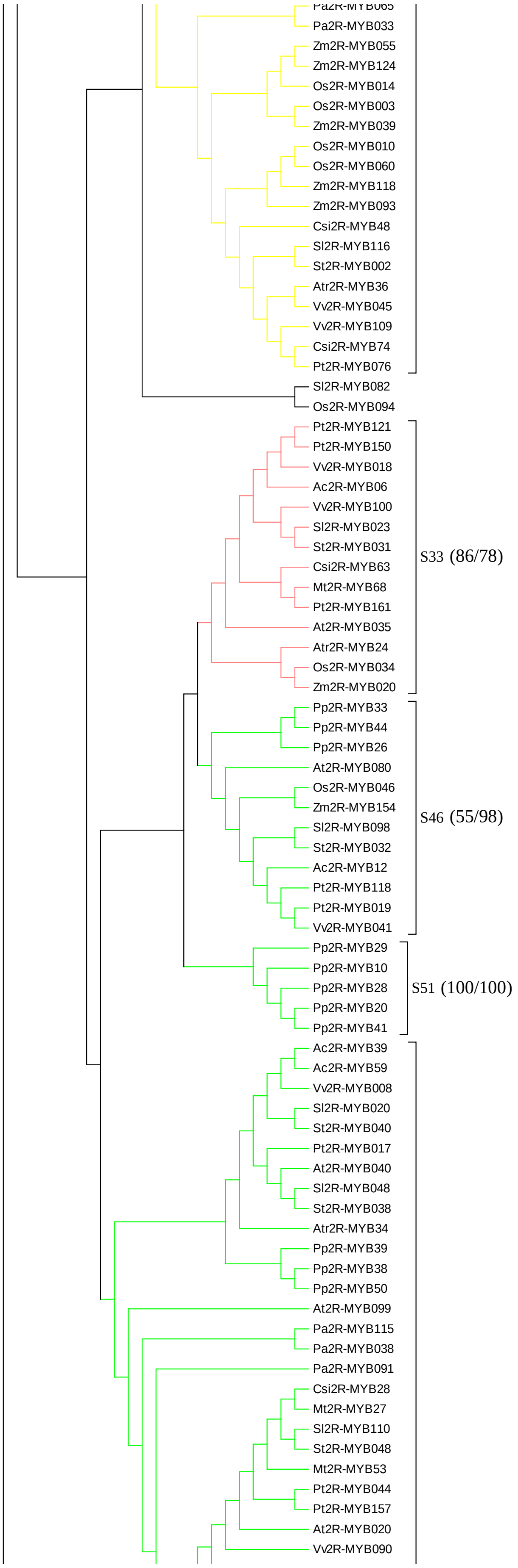

Pa2R-MYB033
Zm2R-MYB055
Zm2R-MYB124
Os2R-MYB014
Os2R-MYB003
Zm2R-MYB039
Os2R-MYB010
Os2R-MYB060
Zm2R-MYB118
Zm2R-MYB093
Csi2R-MYB48
Sl2R-MYB116
St2R-MYB002
Atr2R-MYB36
Vv2R-MYB045
Vv2R-MYB109
Csi2R-MYB74
Pt2R-MYB076
Sl2R-MYB082
Os2R-MYB094
Pt2R-MYB121
Pt2R-MYB150
Vv2R-MYB018
Ac2R-MYB06
Vv2R-MYB100
Sl2R-MYB023
St2R-MYB031
Csi2R-MYB63
Mt2R-MYB68
Pt2R-MYB161
At2R-MYB035
Atr2R-MYB24
Os2R-MYB034
Zm2R-MYB020
S33 (86/78)
Pp2R-MYB33
Pp2R-MYB44
Pp2R-MYB26
At2R-MYB080
Os2R-MYB046
Zm2R-MYB154
Sl2R-MYB098
St2R-MYB032
Ac2R-MYB12
Pt2R-MYB118
Pt2R-MYB019
Vv2R-MYB041
S46 (55/98)
Pp2R-MYB29
Pp2R-MYB10
Pp2R-MYB28
Pp2R-MYB20
Pp2R-MYB41
S51 (100/100)
Ac2R-MYB39
Ac2R-MYB59
Vv2R-MYB008
Sl2R-MYB020
St2R-MYB040
Pt2R-MYB017
At2R-MYB040
Sl2R-MYB048
St2R-MYB038
Atr2R-MYB34
Pp2R-MYB39
Pp2R-MYB38
Pp2R-MYB50
At2R-MYB099
Pa2R-MYB115
Pa2R-MYB038
Pa2R-MYB091
Csi2R-MYB28
Mt2R-MYB27
Sl2R-MYB110
St2R-MYB048
Mt2R-MYB53
Pt2R-MYB044
Pt2R-MYB157
At2R-MYB020
Vv2R-MYB090

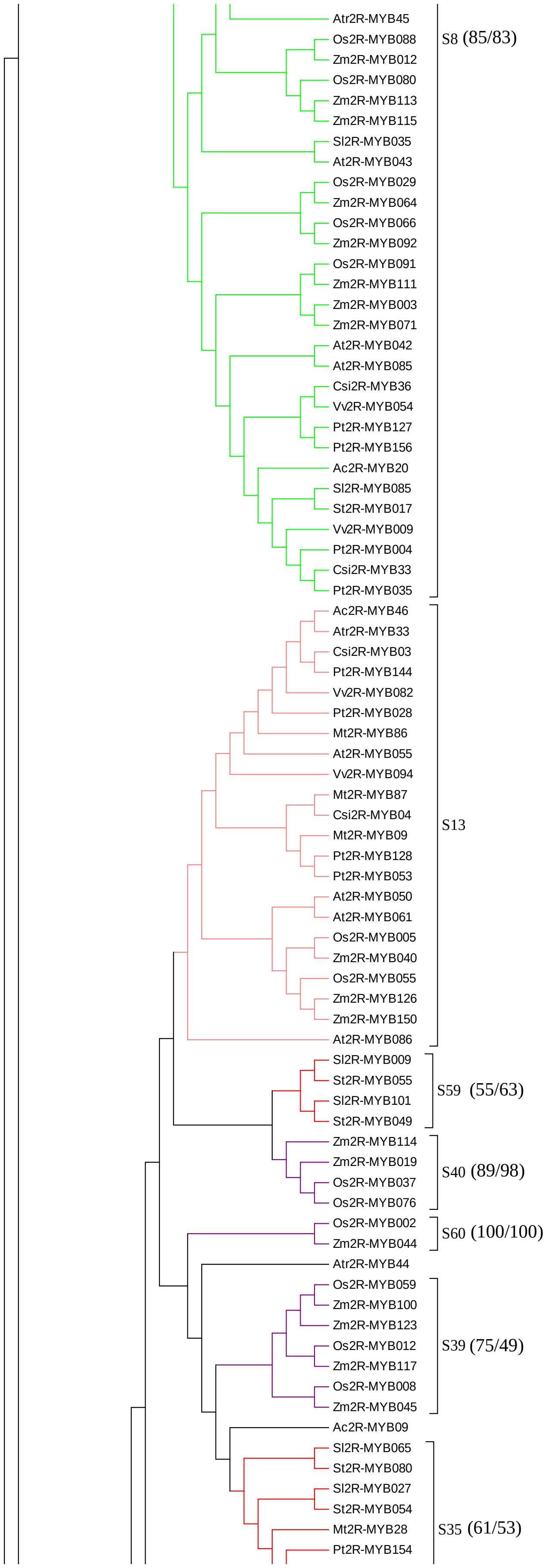
Atr2R-MYB45
Os2R-MYB088
Zm2R-MYB012
Os2R-MYB080
Zm2R-MYB113
Zm2R-MYB115
Sl2R-MYB035
At2R-MYB043
Os2R-MYB029
Zm2R-MYB064
Os2R-MYB066
Zm2R-MYB092
Os2R-MYB091
Zm2R-MYB111
Zm2R-MYB003
Zm2R-MYB071
At2R-MYB042
At2R-MYB085
Csi2R-MYB36
Vv2R-MYB054
Pt2R-MYB127
Pt2R-MYB156
Ac2R-MYB20
Sl2R-MYB085
St2R-MYB017
Vv2R-MYB009
Pt2R-MYB004
Csi2R-MYB33
Pt2R-MYB035
S8 (85/83)
Ac2R-MYB46
Atr2R-MYB33
Csi2R-MYB03
Pt2R-MYB144
Vv2R-MYB082
Pt2R-MYB028
Mt2R-MYB86
At2R-MYB055
Vv2R-MYB094
Mt2R-MYB87
Csi2R-MYB04
Mt2R-MYB09
Pt2R-MYB128
Pt2R-MYB053
At2R-MYB050
At2R-MYB061
Os2R-MYB005
Zm2R-MYB040
Os2R-MYB055
Zm2R-MYB126
Zm2R-MYB150
At2R-MYB086
S13
Sl2R-MYB009
St2R-MYB055
Sl2R-MYB101
St2R-MYB049
S59 (55/63)
Zm2R-MYB114
Zm2R-MYB019
Os2R-MYB037
Os2R-MYB076
S40 (89/98)
Os2R-MYB002
Zm2R-MYB044
S60 (100/100)
Atr2R-MYB44
Os2R-MYB059
Zm2R-MYB100
Zm2R-MYB123
Os2R-MYB012
Zm2R-MYB117
Os2R-MYB008
Zm2R-MYB045
S39 (75/49)
Ac2R-MYB09
Sl2R-MYB065
St2R-MYB080
Sl2R-MYB027
St2R-MYB054
Mt2R-MYB28
Pt2R-MYB154
S35 (61/53)

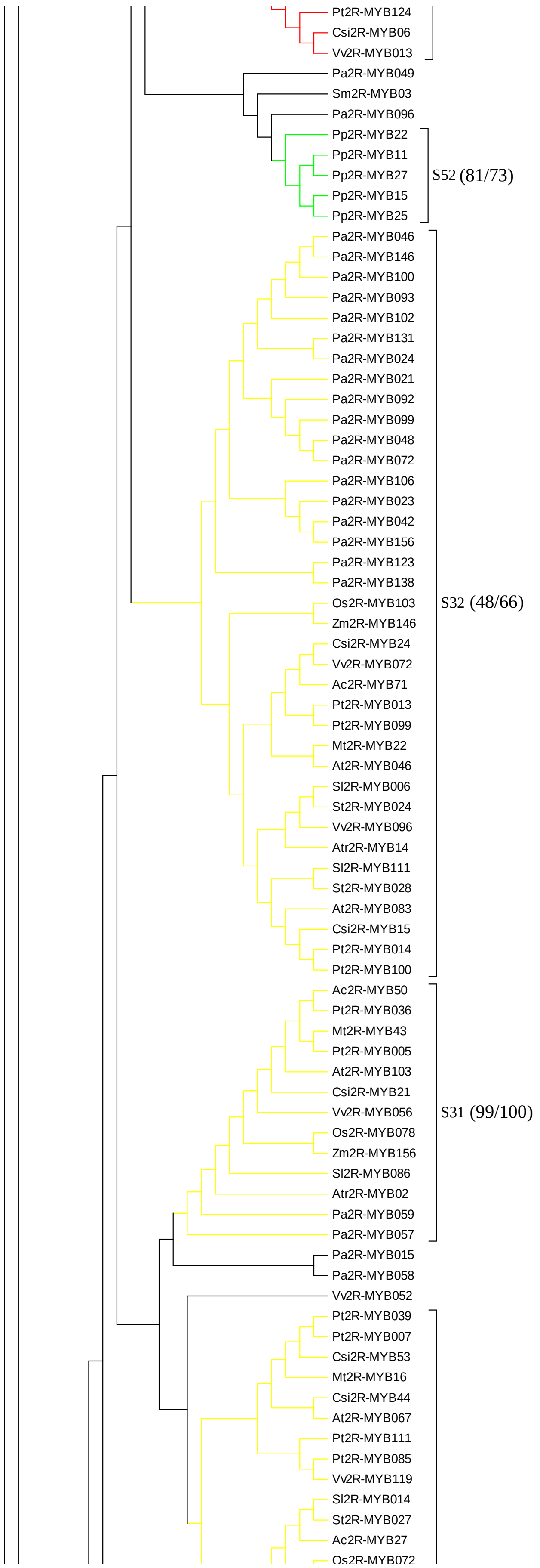
Pt2R-MYB124
Csi2R-MYB06
Vv2R-MYB013
Pa2R-MYB049
Sm2R-MYB03
Pa2R-MYB096
Pp2R-MYB22
Pp2R-MYB11
Pp2R-MYB27
Pp2R-MYB15
Pp2R-MYB25
S52 (81/73)
Pa2R-MYB046
Pa2R-MYB146
Pa2R-MYB100
Pa2R-MYB093
Pa2R-MYB102
Pa2R-MYB131
Pa2R-MYB024
Pa2R-MYB021
Pa2R-MYB092
Pa2R-MYB099
Pa2R-MYB048
Pa2R-MYB072
Pa2R-MYB106
Pa2R-MYB023
Pa2R-MYB042
Pa2R-MYB156
Pa2R-MYB123
Pa2R-MYB138
Os2R-MYB103
Zm2R-MYB146
Csi2R-MYB24
Vv2R-MYB072
Ac2R-MYB71
Pt2R-MYB013
Pt2R-MYB099
Mt2R-MYB22
At2R-MYB046
Sl2R-MYB006
St2R-MYB024
Vv2R-MYB096
Atr2R-MYB14
Sl2R-MYB111
St2R-MYB028
At2R-MYB083
Csi2R-MYB15
Pt2R-MYB014
Pt2R-MYB100
S32 (48/66)
Ac2R-MYB50
Pt2R-MYB036
Mt2R-MYB43
Pt2R-MYB005
At2R-MYB103
Csi2R-MYB21
Vv2R-MYB056
Os2R-MYB078
Zm2R-MYB156
Sl2R-MYB086
Atr2R-MYB02
Pa2R-MYB059
Pa2R-MYB057
S31 (99/100)
Pa2R-MYB015
Pa2R-MYB058
Vv2R-MYB052
Pt2R-MYB039
Pt2R-MYB007
Csi2R-MYB53
Mt2R-MYB16
Csi2R-MYB44
At2R-MYB067
Pt2R-MYB111
Pt2R-MYB085
Vv2R-MYB119
Sl2R-MYB014
St2R-MYB027
Ac2R-MYB27
Os2R-MYB072

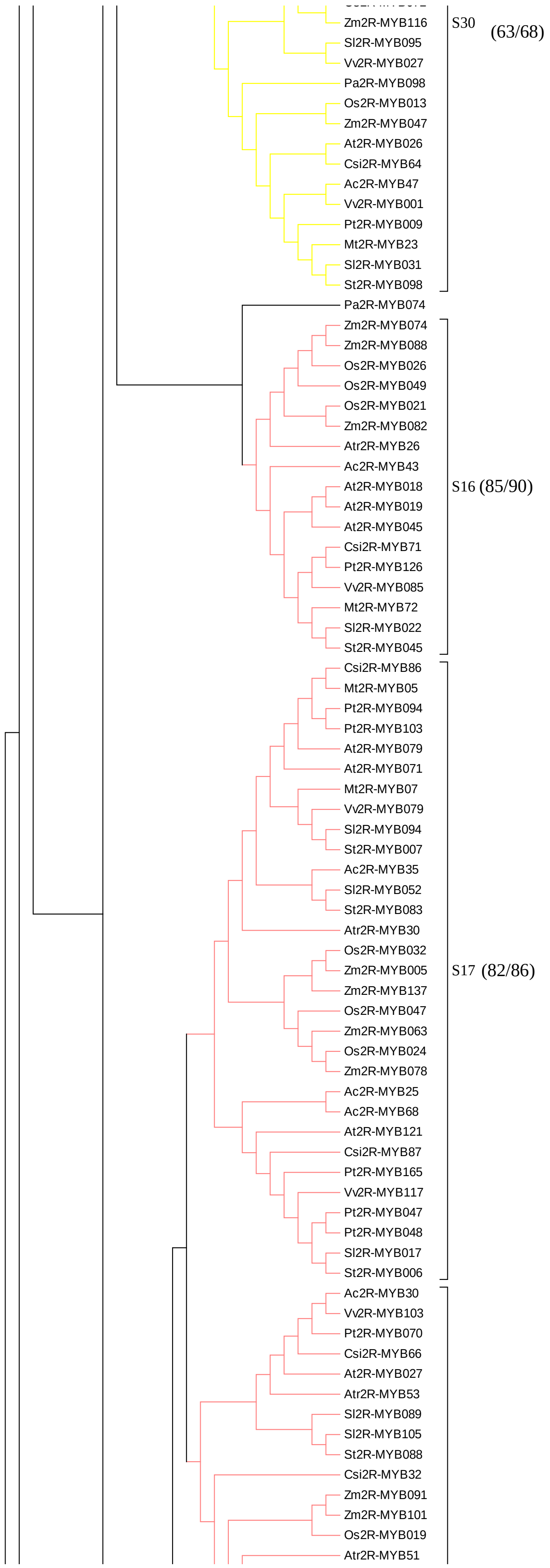
Zm2R-MYB116
Sl2R-MYB095
Vv2R-MYB027
Pa2R-MYB098
Os2R-MYB013
Zm2R-MYB047
At2R-MYB026
Csi2R-MYB64
Ac2R-MYB47
Vv2R-MYB001
Pt2R-MYB009
Mt2R-MYB23
Sl2R-MYB031
St2R-MYB098
S30 (63/68)
Pa2R-MYB074
Zm2R-MYB074
Zm2R-MYB088
Os2R-MYB026
Os2R-MYB049
Os2R-MYB021
Zm2R-MYB082
Atr2R-MYB26
Ac2R-MYB43
At2R-MYB018
At2R-MYB019
At2R-MYB045
Csi2R-MYB71
Pt2R-MYB126
Vv2R-MYB085
Mt2R-MYB72
Sl2R-MYB022
St2R-MYB045
S16 (85/90)
Csi2R-MYB86
Mt2R-MYB05
Pt2R-MYB094
Pt2R-MYB103
At2R-MYB079
At2R-MYB071
Mt2R-MYB07
Vv2R-MYB079
Sl2R-MYB094
St2R-MYB007
Ac2R-MYB35
Sl2R-MYB052
St2R-MYB083
Atr2R-MYB30
Os2R-MYB032
Zm2R-MYB005
Zm2R-MYB137
Os2R-MYB047
Zm2R-MYB063
Os2R-MYB024
Zm2R-MYB078
Ac2R-MYB25
Ac2R-MYB68
At2R-MYB121
Csi2R-MYB87
Pt2R-MYB165
Vv2R-MYB117
Pt2R-MYB047
Pt2R-MYB048
Sl2R-MYB017
St2R-MYB006
S17 (82/86)
Ac2R-MYB30
Vv2R-MYB103
Pt2R-MYB070
Csi2R-MYB66
At2R-MYB027
Atr2R-MYB53
Sl2R-MYB089
Sl2R-MYB105
St2R-MYB088
Csi2R-MYB32
Zm2R-MYB091
Zm2R-MYB101
Os2R-MYB019
Atr2R-MYB51

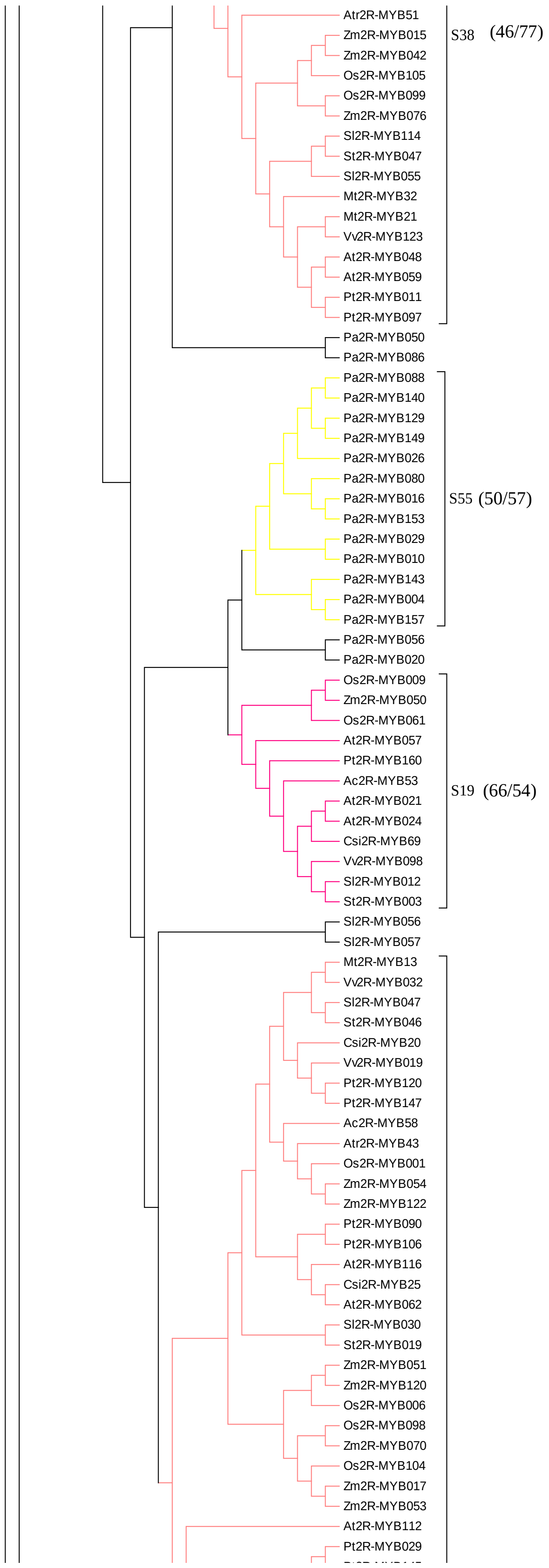

Atr2R-MYB51
Zm2R-MYB015
Zm2R-MYB042
Os2R-MYB105
Os2R-MYB099
Zm2R-MYB076
Sl2R-MYB114
St2R-MYB047
Sl2R-MYB055
Mt2R-MYB32
Mt2R-MYB21
Vv2R-MYB123
At2R-MYB048
At2R-MYB059
Pt2R-MYB011
Pt2R-MYB097
S38 (46/77)
Pa2R-MYB050
Pa2R-MYB086
Pa2R-MYB088
Pa2R-MYB140
Pa2R-MYB129
Pa2R-MYB149
Pa2R-MYB026
Pa2R-MYB080
Pa2R-MYB016
Pa2R-MYB153
Pa2R-MYB029
Pa2R-MYB010
Pa2R-MYB143
Pa2R-MYB004
Pa2R-MYB157
S55 (50/57)
Pa2R-MYB056
Pa2R-MYB020
Os2R-MYB009
Zm2R-MYB050
Os2R-MYB061
At2R-MYB057
Pt2R-MYB160
Ac2R-MYB53
At2R-MYB021
At2R-MYB024
Csi2R-MYB69
Vv2R-MYB098
Sl2R-MYB012
St2R-MYB003
S19 (66/54)
Sl2R-MYB056
Sl2R-MYB057
Mt2R-MYB13
Vv2R-MYB032
Sl2R-MYB047
St2R-MYB046
Csi2R-MYB20
Vv2R-MYB019
Pt2R-MYB120
Pt2R-MYB147
Ac2R-MYB58
Atr2R-MYB43
Os2R-MYB001
Zm2R-MYB054
Zm2R-MYB122
Pt2R-MYB090
Pt2R-MYB106
At2R-MYB116
Csi2R-MYB25
At2R-MYB062
Sl2R-MYB030
St2R-MYB019
Zm2R-MYB051
Zm2R-MYB120
Os2R-MYB006
Os2R-MYB098
Zm2R-MYB070
Os2R-MYB104
Zm2R-MYB017
Zm2R-MYB053
At2R-MYB112
Pt2R-MYB029

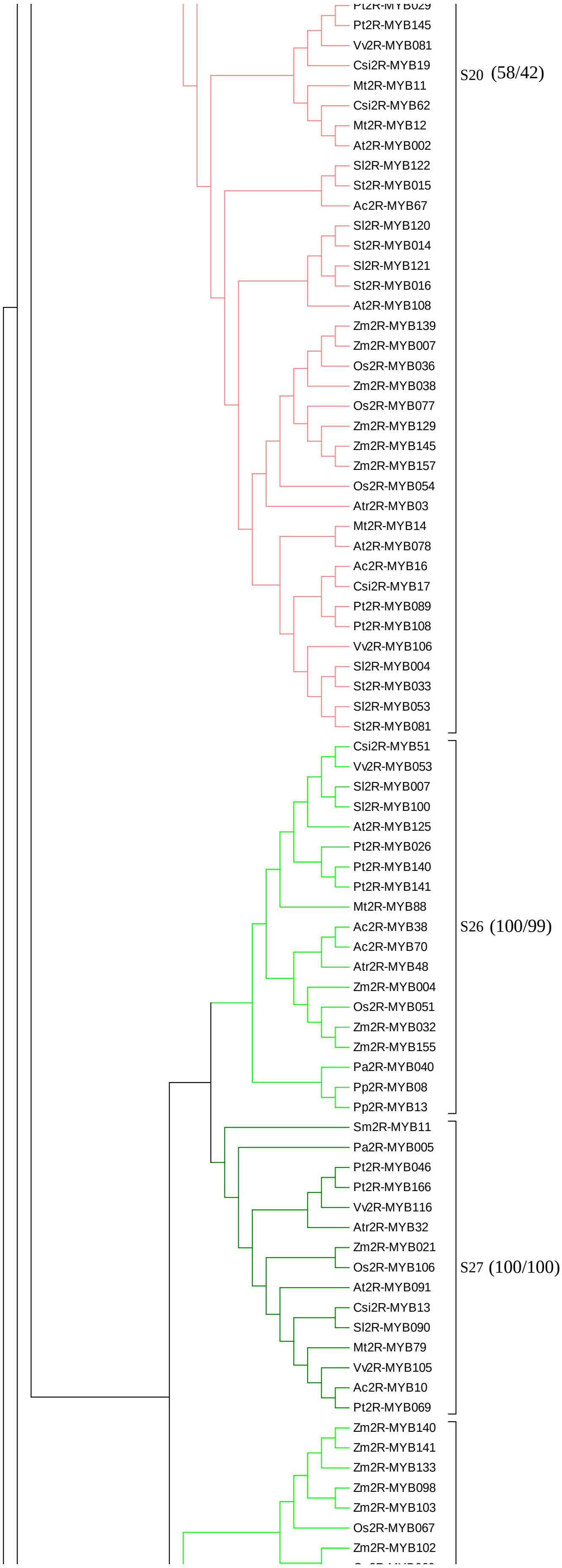
Pt2R-MYB145
Vv2R-MYB081
Csi2R-MYB19
Mt2R-MYB11
Csi2R-MYB62
Mt2R-MYB12
At2R-MYB002
Sl2R-MYB122
St2R-MYB015
Ac2R-MYB67
Sl2R-MYB120
St2R-MYB014
Sl2R-MYB121
St2R-MYB016
At2R-MYB108
Zm2R-MYB139
Zm2R-MYB007
Os2R-MYB036
Zm2R-MYB038
Os2R-MYB077
Zm2R-MYB129
Zm2R-MYB145
Zm2R-MYB157
Os2R-MYB054
Atr2R-MYB03
Mt2R-MYB14
At2R-MYB078
Ac2R-MYB16
Csi2R-MYB17
Pt2R-MYB089
Pt2R-MYB108
Vv2R-MYB106
Sl2R-MYB004
St2R-MYB033
Sl2R-MYB053
St2R-MYB081
S20 (58/42)
Csi2R-MYB51
Vv2R-MYB053
Sl2R-MYB007
Sl2R-MYB100
At2R-MYB125
Pt2R-MYB026
Pt2R-MYB140
Pt2R-MYB141
Mt2R-MYB88
Ac2R-MYB38
Ac2R-MYB70
Atr2R-MYB48
Zm2R-MYB004
Os2R-MYB051
Zm2R-MYB032
Zm2R-MYB155
Pa2R-MYB040
Pp2R-MYB08
Pp2R-MYB13
S26 (100/99)
Sm2R-MYB11
Pa2R-MYB005
Pt2R-MYB046
Pt2R-MYB166
Vv2R-MYB116
Atr2R-MYB32
Zm2R-MYB021
Os2R-MYB106
At2R-MYB091
Csi2R-MYB13
Sl2R-MYB090
Mt2R-MYB79
Vv2R-MYB105
Ac2R-MYB10
Pt2R-MYB069
S27 (100/100)
Zm2R-MYB140
Zm2R-MYB141
Zm2R-MYB133
Zm2R-MYB098
Zm2R-MYB103
Os2R-MYB067
Zm2R-MYB102

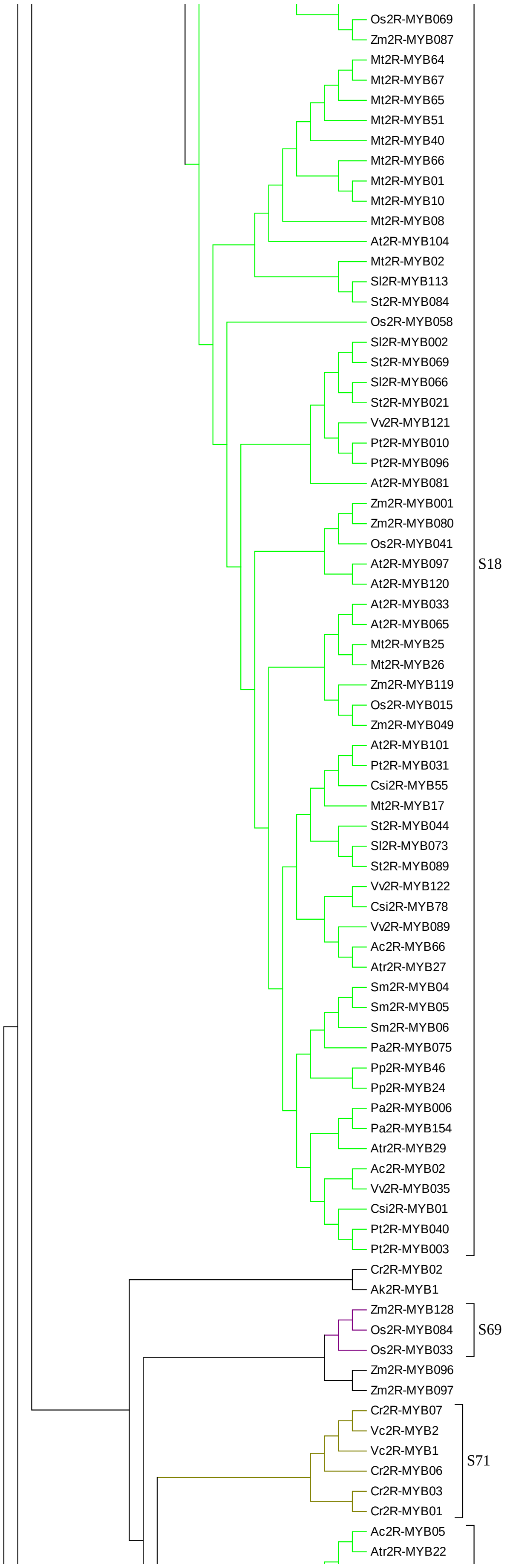
Os2R-MYB069
Zm2R-MYB087
Mt2R-MYB64
Mt2R-MYB67
Mt2R-MYB65
Mt2R-MYB51
Mt2R-MYB40
Mt2R-MYB66
Mt2R-MYB01
Mt2R-MYB10
Mt2R-MYB08
At2R-MYB104
Mt2R-MYB02
Sl2R-MYB113
St2R-MYB084
Os2R-MYB058
Sl2R-MYB002
St2R-MYB069
Sl2R-MYB066
St2R-MYB021
Vv2R-MYB121
Pt2R-MYB010
Pt2R-MYB096
At2R-MYB081
Zm2R-MYB001
Zm2R-MYB080
Os2R-MYB041
At2R-MYB097
At2R-MYB120
At2R-MYB033
At2R-MYB065
Mt2R-MYB25
Mt2R-MYB26
Zm2R-MYB119
Os2R-MYB015
Zm2R-MYB049
At2R-MYB101
Pt2R-MYB031
Csi2R-MYB55
Mt2R-MYB17
St2R-MYB044
Sl2R-MYB073
St2R-MYB089
Vv2R-MYB122
Csi2R-MYB78
Vv2R-MYB089
Ac2R-MYB66
Atr2R-MYB27
Sm2R-MYB04
Sm2R-MYB05
Sm2R-MYB06
Pa2R-MYB075
Pp2R-MYB46
Pp2R-MYB24
Pa2R-MYB006
Pa2R-MYB154
Atr2R-MYB29
Ac2R-MYB02
Vv2R-MYB035
Csi2R-MYB01
Pt2R-MYB040
Pt2R-MYB003
S18
Cr2R-MYB02
Ak2R-MYB1
Zm2R-MYB128
Os2R-MYB084
Os2R-MYB033
S69
Zm2R-MYB096
Zm2R-MYB097
Cr2R-MYB07
Vc2R-MYB2
Vc2R-MYB1
Cr2R-MYB06
Cr2R-MYB03
Cr2R-MYB01
S71
Ac2R-MYB05
Atr2R-MYB22

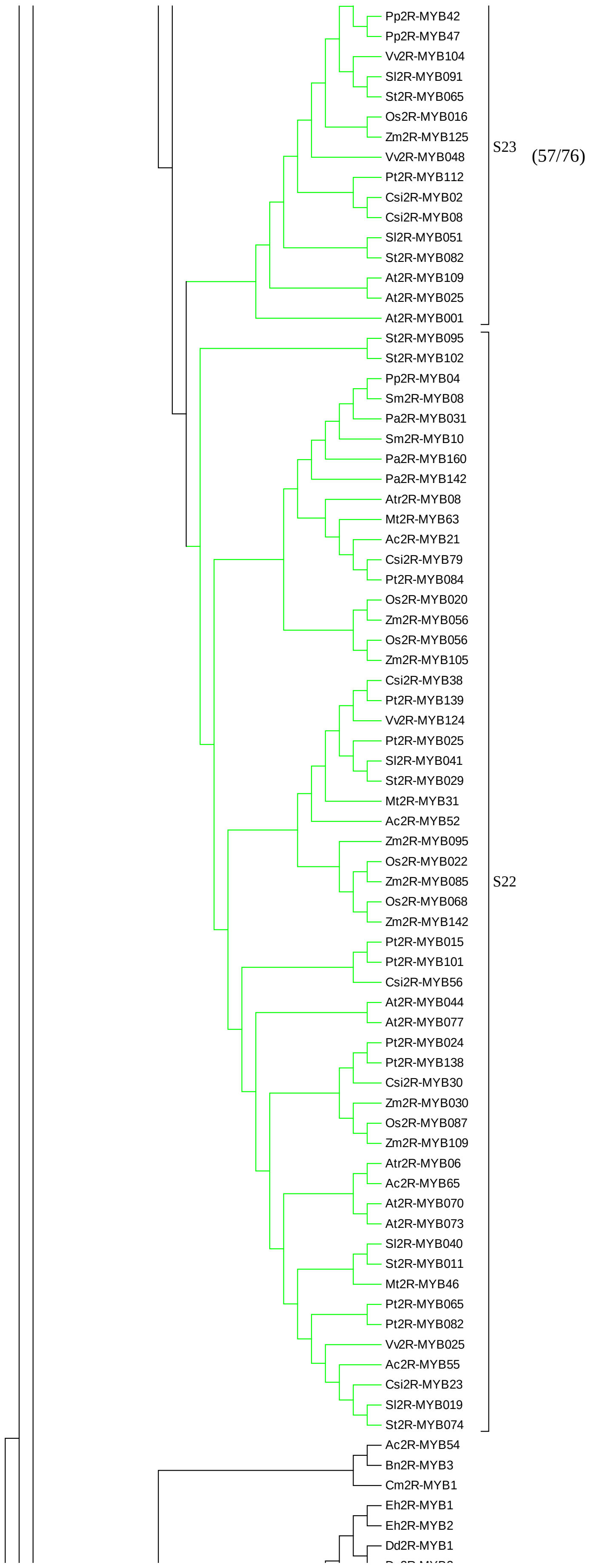
Pp2R-MYB42
Pp2R-MYB47
Vv2R-MYB104
Sl2R-MYB091
St2R-MYB065
Os2R-MYB016
Zm2R-MYB125
Vv2R-MYB048
Pt2R-MYB112
Csi2R-MYB02
Csi2R-MYB08
Sl2R-MYB051
St2R-MYB082
At2R-MYB109
At2R-MYB025
At2R-MYB001
S23
(57/76)
St2R-MYB095
St2R-MYB102
Pp2R-MYB04
Sm2R-MYB08
Pa2R-MYB031
Sm2R-MYB10
Pa2R-MYB160
Pa2R-MYB142
Atr2R-MYB08
Mt2R-MYB63
Ac2R-MYB21
Csi2R-MYB79
Pt2R-MYB084
Os2R-MYB020
Zm2R-MYB056
Os2R-MYB056
Zm2R-MYB105
Csi2R-MYB38
Pt2R-MYB139
Vv2R-MYB124
Pt2R-MYB025
Sl2R-MYB041
St2R-MYB029
Mt2R-MYB31
Ac2R-MYB52
Zm2R-MYB095
Os2R-MYB022
Zm2R-MYB085
Os2R-MYB068
Zm2R-MYB142
Pt2R-MYB015
Pt2R-MYB101
Csi2R-MYB56
At2R-MYB044
At2R-MYB077
Pt2R-MYB024
Pt2R-MYB138
Csi2R-MYB30
Zm2R-MYB030
Os2R-MYB087
Zm2R-MYB109
Atr2R-MYB06
Ac2R-MYB65
At2R-MYB070
At2R-MYB073
Sl2R-MYB040
St2R-MYB011
Mt2R-MYB46
Pt2R-MYB065
Pt2R-MYB082
Vv2R-MYB025
Ac2R-MYB55
Csi2R-MYB23
Sl2R-MYB019
St2R-MYB074
S22
Ac2R-MYB54
Bn2R-MYB3
Cm2R-MYB1
Eh2R-MYB1
Eh2R-MYB2
Dd2R-MYB1

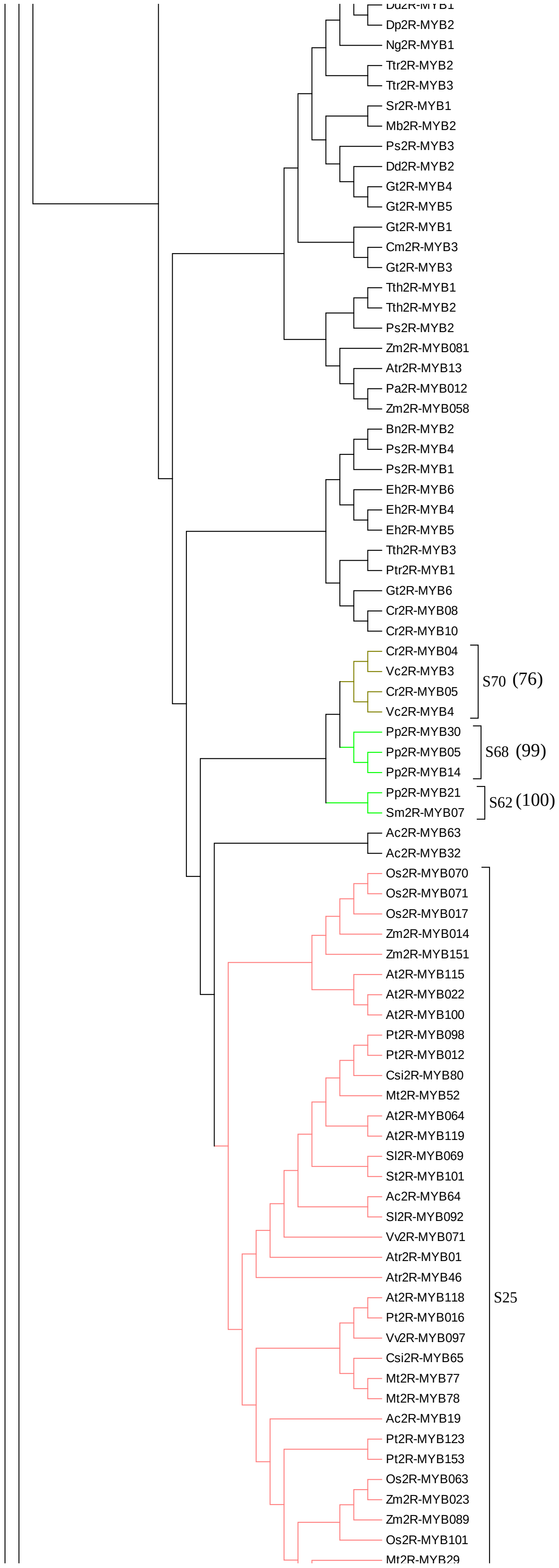
Dp2R-MYB2
Ng2R-MYB1
Ttr2R-MYB2
Ttr2R-MYB3
Sr2R-MYB1
Mb2R-MYB2
Ps2R-MYB3
Dd2R-MYB2
Gt2R-MYB4
Gt2R-MYB5
Gt2R-MYB1
Cm2R-MYB3
Gt2R-MYB3
Tth2R-MYB1
Tth2R-MYB2
Ps2R-MYB2
Zm2R-MYB081
Atr2R-MYB13
Pa2R-MYB012
Zm2R-MYB058
Bn2R-MYB2
Ps2R-MYB4
Ps2R-MYB1
Eh2R-MYB6
Eh2R-MYB4
Eh2R-MYB5
Tth2R-MYB3
Ptr2R-MYB1
Gt2R-MYB6
Cr2R-MYB08
Cr2R-MYB10
Cr2R-MYB04
Vc2R-MYB3
Cr2R-MYB05
Vc2R-MYB4
S70 (76)
Pp2R-MYB30
Pp2R-MYB05
Pp2R-MYB14
S68 (99)
Pp2R-MYB21
Sm2R-MYB07
S62 (100)
Ac2R-MYB63
Ac2R-MYB32
Os2R-MYB070
Os2R-MYB071
Os2R-MYB017
Zm2R-MYB014
Zm2R-MYB151
At2R-MYB115
At2R-MYB022
At2R-MYB100
Pt2R-MYB098
Pt2R-MYB012
Csi2R-MYB80
Mt2R-MYB52
At2R-MYB064
At2R-MYB119
Sl2R-MYB069
St2R-MYB101
Ac2R-MYB64
Sl2R-MYB092
Vv2R-MYB071
Atr2R-MYB01
Atr2R-MYB46
At2R-MYB118
Pt2R-MYB016
Vv2R-MYB097
Csi2R-MYB65
Mt2R-MYB77
Mt2R-MYB78
Ac2R-MYB19
Pt2R-MYB123
Pt2R-MYB153
Os2R-MYB063
Zm2R-MYB023
Zm2R-MYB089
Os2R-MYB101
Mt2R-MYB29
S25

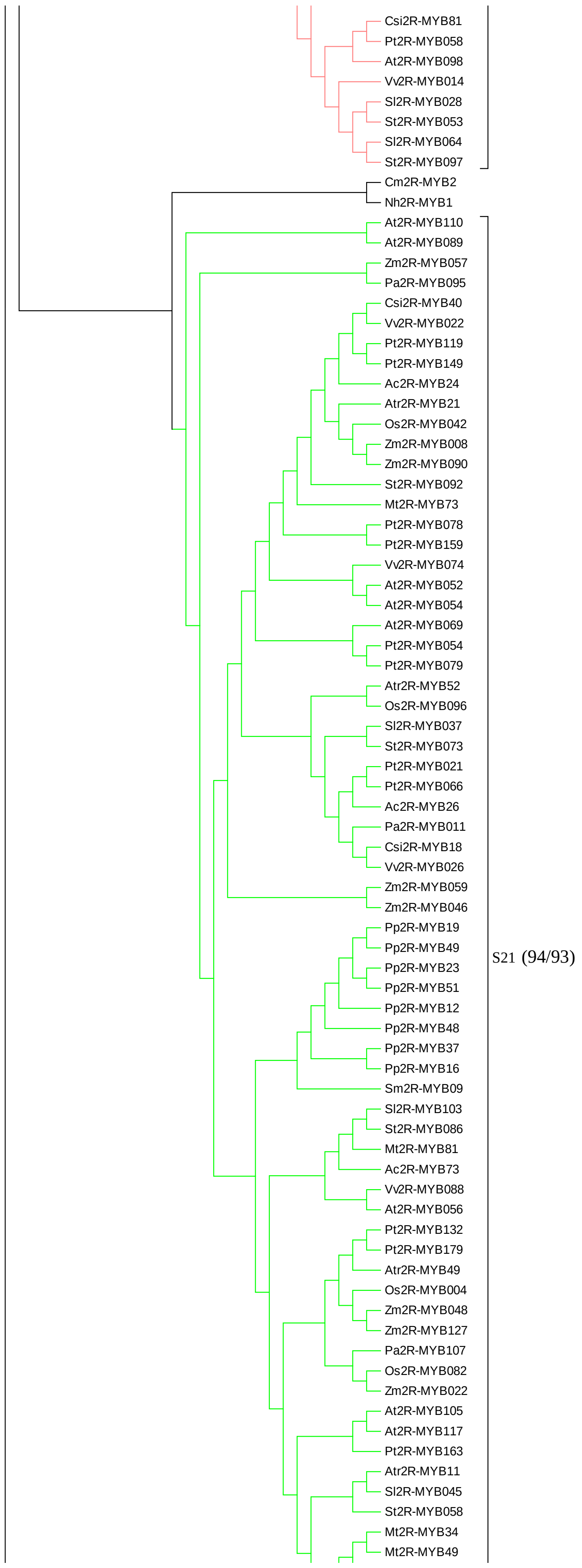
Csi2R-MYB81
Pt2R-MYB058
At2R-MYB098
Vv2R-MYB014
Sl2R-MYB028
St2R-MYB053
Sl2R-MYB064
St2R-MYB097
Cm2R-MYB2
Nh2R-MYB1
At2R-MYB110
At2R-MYB089
Zm2R-MYB057
Pa2R-MYB095
Csi2R-MYB40
Vv2R-MYB022
Pt2R-MYB119
Pt2R-MYB149
Ac2R-MYB24
Atr2R-MYB21
Os2R-MYB042
Zm2R-MYB008
Zm2R-MYB090
St2R-MYB092
Mt2R-MYB73
Pt2R-MYB078
Pt2R-MYB159
Vv2R-MYB074
At2R-MYB052
At2R-MYB054
At2R-MYB069
Pt2R-MYB054
Pt2R-MYB079
Atr2R-MYB52
Os2R-MYB096
Sl2R-MYB037
St2R-MYB073
Pt2R-MYB021
Pt2R-MYB066
Ac2R-MYB26
Pa2R-MYB011
Csi2R-MYB18
Vv2R-MYB026
Zm2R-MYB059
Zm2R-MYB046
Pp2R-MYB19
Pp2R-MYB49
Pp2R-MYB23
Pp2R-MYB51
Pp2R-MYB12
Pp2R-MYB48
Pp2R-MYB37
Pp2R-MYB16
Sm2R-MYB09
Sl2R-MYB103
St2R-MYB086
Mt2R-MYB81
Ac2R-MYB73
Vv2R-MYB088
At2R-MYB056
Pt2R-MYB132
Pt2R-MYB179
Atr2R-MYB49
Os2R-MYB004
Zm2R-MYB048
Zm2R-MYB127
Pa2R-MYB107
Os2R-MYB082
Zm2R-MYB022
At2R-MYB105
At2R-MYB117
Pt2R-MYB163
Atr2R-MYB11
Sl2R-MYB045
St2R-MYB058
Mt2R-MYB34
Mt2R-MYB49
S21 (94/93)

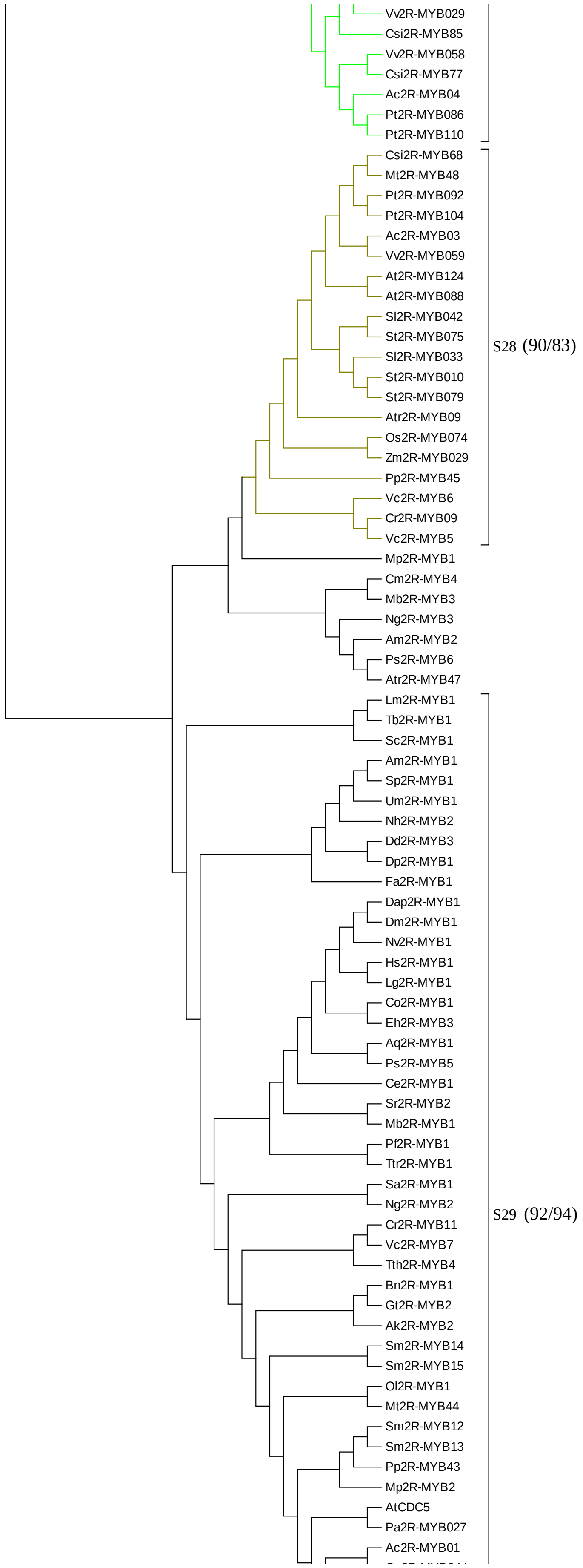
Vv2R-MYB029
Csi2R-MYB85
Vv2R-MYB058
Csi2R-MYB77
Ac2R-MYB04
Pt2R-MYB086
Pt2R-MYB110
Csi2R-MYB68
Mt2R-MYB48
Pt2R-MYB092
Pt2R-MYB104
Ac2R-MYB03
Vv2R-MYB059
At2R-MYB124
At2R-MYB088
Sl2R-MYB042
St2R-MYB075
Sl2R-MYB033
St2R-MYB010
St2R-MYB079
Atr2R-MYB09
Os2R-MYB074
Zm2R-MYB029
Pp2R-MYB45
Vc2R-MYB6
Cr2R-MYB09
Vc2R-MYB5
S28 (90/83)
Mp2R-MYB1
Cm2R-MYB4
Mb2R-MYB3
Ng2R-MYB3
Am2R-MYB2
Ps2R-MYB6
Atr2R-MYB47
Lm2R-MYB1
Tb2R-MYB1
Sc2R-MYB1
Am2R-MYB1
Sp2R-MYB1
Um2R-MYB1
Nh2R-MYB2
Dd2R-MYB3
Dp2R-MYB1
Fa2R-MYB1
Dap2R-MYB1
Dm2R-MYB1
Nv2R-MYB1
Hs2R-MYB1
Lg2R-MYB1
Co2R-MYB1
Eh2R-MYB3
Aq2R-MYB1
Ps2R-MYB5
Ce2R-MYB1
Sr2R-MYB2
Mb2R-MYB1
Pf2R-MYB1
Ttr2R-MYB1
Sa2R-MYB1
Ng2R-MYB2
S29 (92/94)
Cr2R-MYB11
Vc2R-MYB7
Tth2R-MYB4
Bn2R-MYB1
Gt2R-MYB2
Ak2R-MYB2
Sm2R-MYB14
Sm2R-MYB15
Ol2R-MYB1
Mt2R-MYB44
Sm2R-MYB12
Sm2R-MYB13
Pp2R-MYB43
Mp2R-MYB2
AtCDC5
Pa2R-MYB027
Ac2R-MYB01

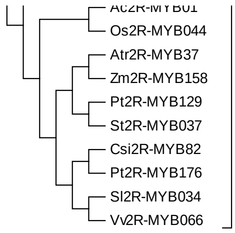

Supplementary Figure S1. Phylogenetic tree of 1548 eukaryote 2R-MYB proteins. The unrooted tree was constructed by the neighbor-joining (NJ) method based on the alignment of the corresponding MYB domains of the candidates listed in Supplementary Table S1. The 2R-MYB proteins are clustered into 73 subgroups (designated as S1 to S73). The first 25 subgroups were designated as previously reports of AtMYBs by Stracke et al. (2001). Bootstrap values of the corresponding subfamilies in NJ (Fig. 2) and maximum likelihood (ML) analysis are listed in round bracket (>50%), for reference, respectively.

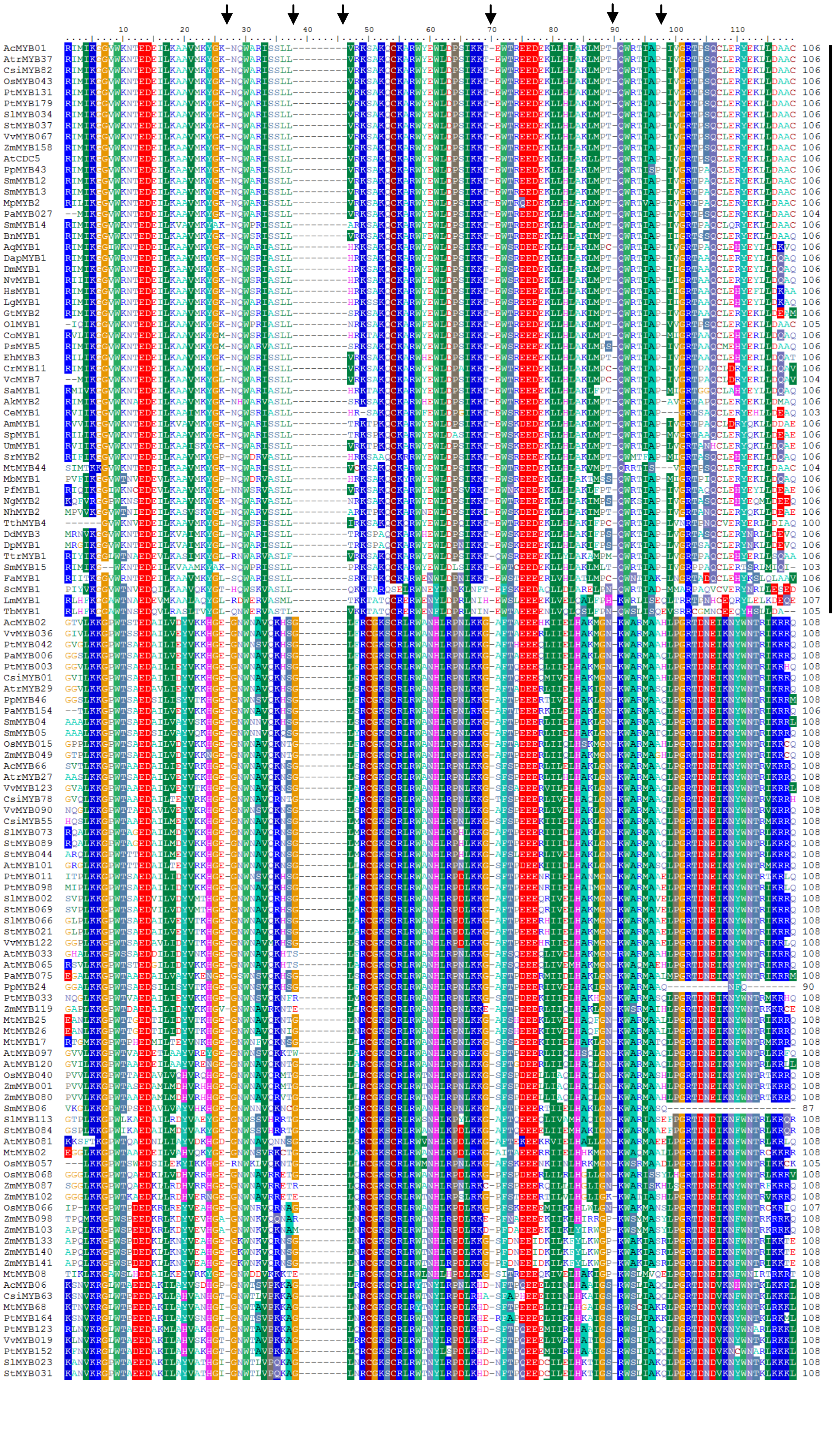

S29

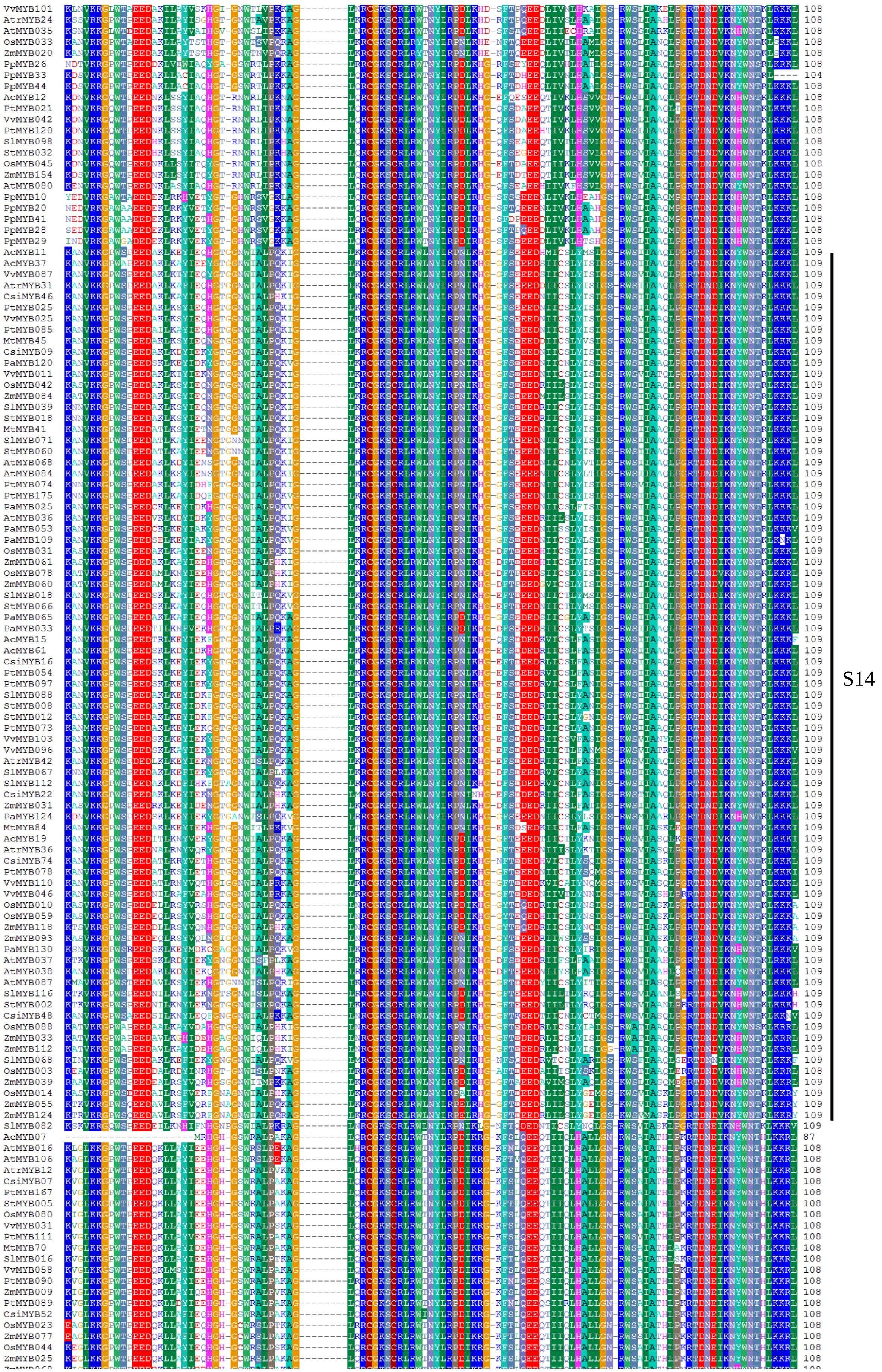
VvMYB101
AtrMYB24
AtMYB035
OsMYB033
ZmMYB020
PpMYB26
PpMYB33
PpMYB44
AcMYB12
PtMYB021
VvMYB042
PtMYB120
SlMYB098
StMYB032
OsMYB045
ZmMYB154
AtMYB080
PpMYB10
PpMYB20
PpMYB41
PpMYB28
PpMYB29
AcMYB11
AcMYB37
VvMYB087
AtrMYB31
CsiMYB46
PtMYB025
VvMYB025
PtMYB085
MtMYB45
CsiMYB09
PaMYB120
VvMYB011
OsMYB042
ZmMYB084
SlMYB039
StMYB018
MtMYB41
SlMYB071
StMYB060
AtMYB068
AtMYB084
PtMYB074
PtMYB175
PaMYB025
AtMYB036
PaMYB053
PaMYB109
OsMYB031
ZmMYB061
OsMYB078
ZmMYB060
SlMYB018
StMYB066
PaMYB065
PaMYB033
AcMYB15
AcMYB61
CsiMYB16
PtMYB054
PtMYB097
SlMYB088
StMYB008
StMYB012
PtMYB073
VvMYB103
VvMYB096
AtrMYB42
SlMYB067
SlMYB112
CsiMYB22
ZmMYB031
PaMYB124
MtMYB84
AcMYB19
AtrMYB36
CsiMYB74
PtMYB078
VvMYB110
VvMYB046
OsMYB010
OsMYB059
ZmMYB118
ZmMYB093
PaMYB130
AtMYB037
AtMYB038
AtMYB087
SlMYB116
StMYB002
CsiMYB48
OsMYB088
ZmMYB033
ZmMYB112
SlMYB068
OsMYB003
ZmMYB039
OsMYB014
ZmMYB055
ZmMYB124
SlMYB082
AcMYB07
AtMYB016
AtMYB106
AtrMYB12
CsiMYB07
PtMYB167
StMYB005
OsMYB080
VvMYB031
PtMYB111
MtMYB70
SlMYB016
VvMYB058
PtMYB090
ZmMYB009
PtMYB089
CsiMYB52
OsMYB023
ZmMYB077
OsMYB044
ZmMYB025
S14

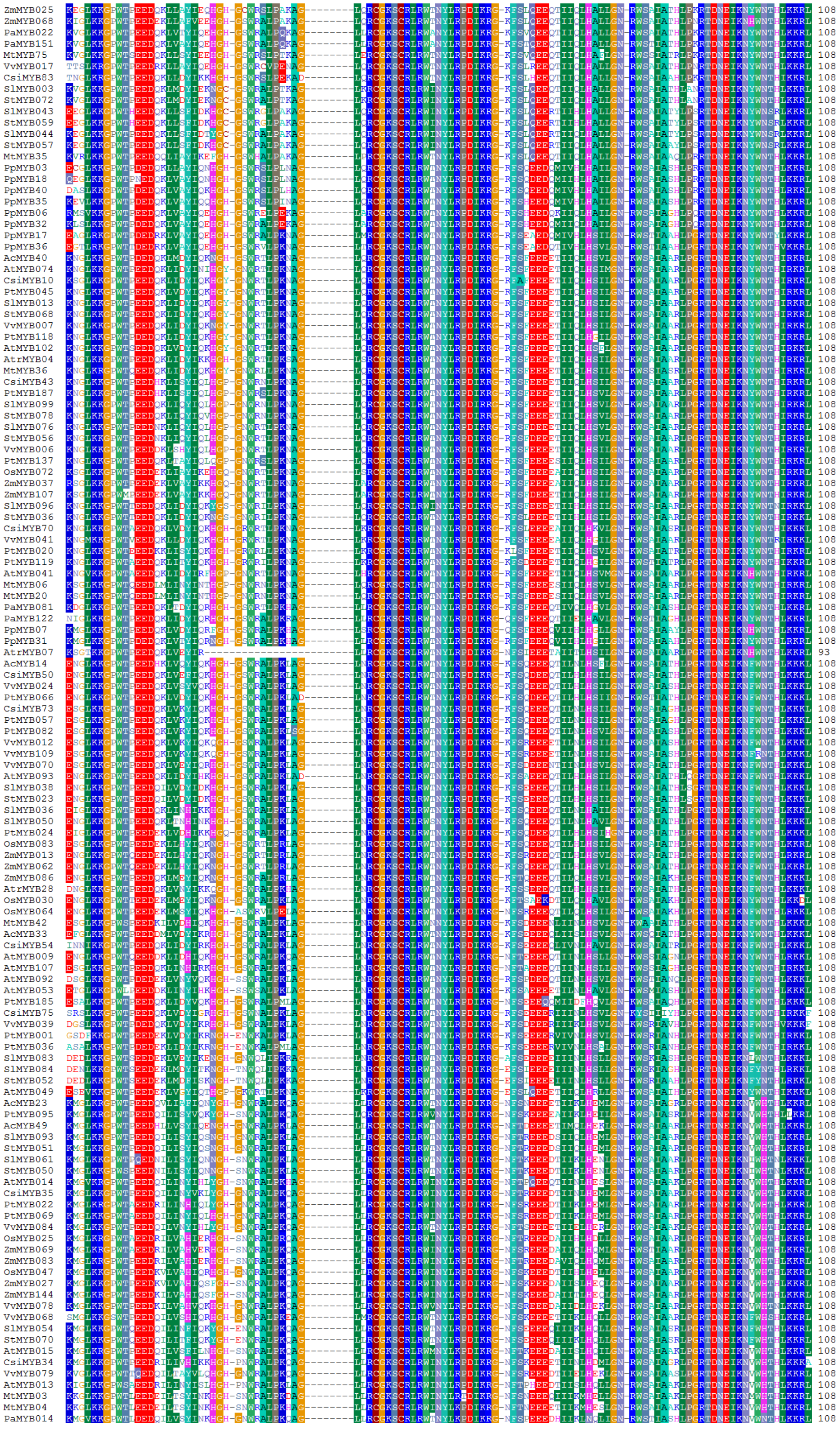

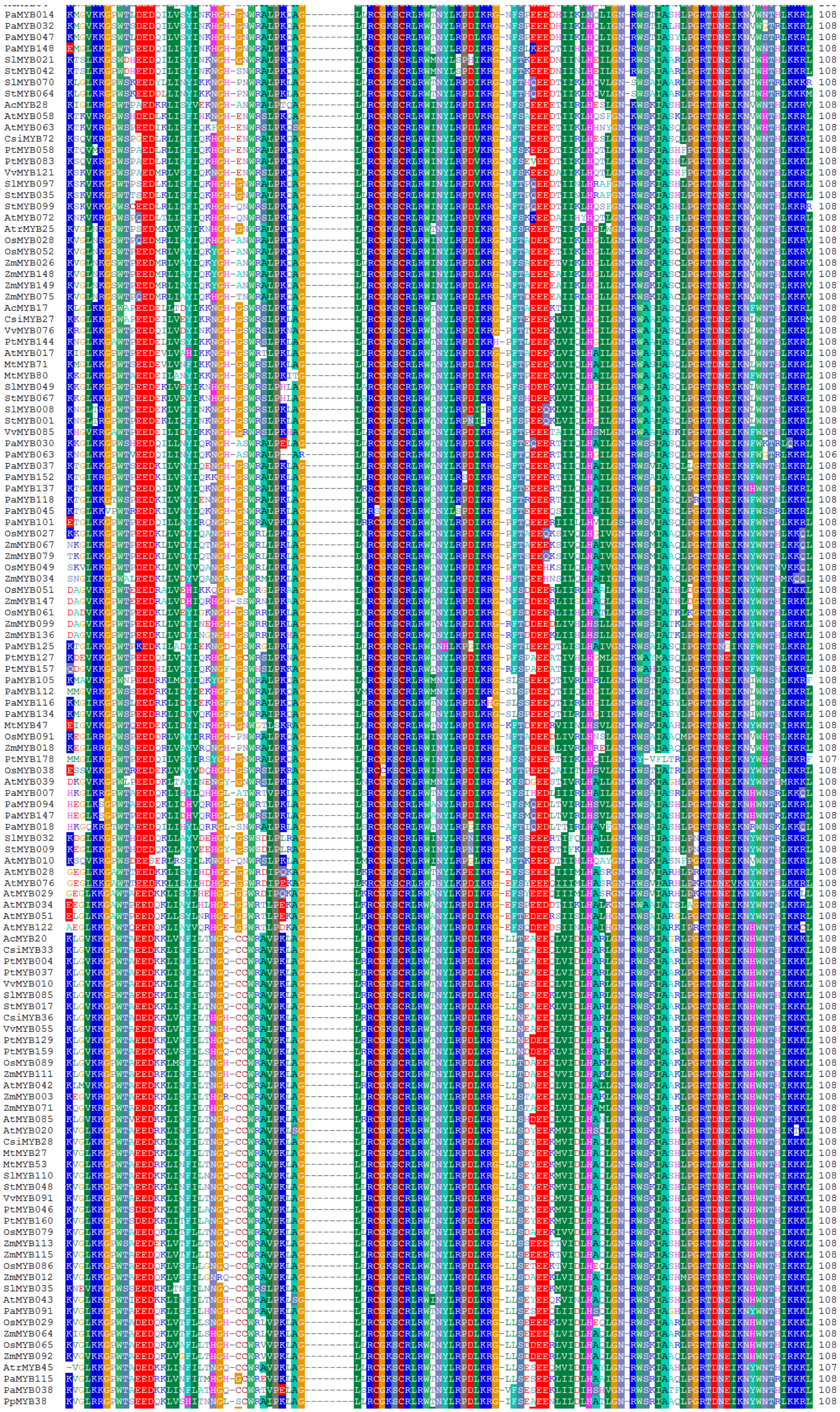
PaMYB014
PaMYB032
PaMYB047
PaMYB148
SlMYB021
StMYB042
SlMYB070
StMYB064
AcMYB28
AtMYB058
AtMYB063
CsiMYB72
PtMYB058
PtMYB083
VvMYB121
SlMYB097
StMYB035
StMYB099
AtMYB072
AtrMYB25
OsMYB028
OsMYB052
ZmMYB026
ZmMYB148
ZmMYB149
ZmMYB075
AcMYB17
CsiMYB27
VvMYB076
PtMYB144
AtMYB017
MtMYB71
MtMYB80
SlMYB049
StMYB067
SlMYB008
StMYB001
VvMYB085
PaMYB030
PaMYB063
PaMYB037
PaMYB152
PaMYB137
PaMYB118
PaMYB045
PaMYB101
OsMYB027
ZmMYB067
ZmMYB079
OsMYB049
ZmMYB034
OsMYB051
ZmMYB147
OsMYB061
ZmMYB099
ZmMYB136
PaMYB125
PtMYB127
PtMYB157
PaMYB105
PaMYB112
PaMYB116
PaMYB134
MtMYB47
OsMYB091
ZmMYB018
PtMYB178
OsMYB038
AtMYB039
PaMYB007
PaMYB094
PaMYB147
PaMYB018
SlMYB032
StMYB009
AtMYB010
AtMYB028
AtMYB076
AtMYB029
AtMYB034
AtMYB051
AtMYB122
AcMYB20
CsiMYB33
PtMYB004
PtMYB037
VvMYB010
SlMYB085
StMYB017
CsiMYB36
VvMYB055
PtMYB129
PtMYB159
OsMYB089
ZmMYB111
AtMYB042
ZmMYB003
ZmMYB071
AtMYB085
AtMYB020
CsiMYB28
MtMYB27
MtMYB53
SlMYB110
StMYB048
VvMYB091
PtMYB046
PtMYB160
OsMYB079
ZmMYB113
ZmMYB115
OsMYB086
ZmMYB012
SlMYB035
AtMYB043
PaMYB091
OsMYB029
ZmMYB064
OsMYB065
ZmMYB092
AtrMYB45
PaMYB115
PaMYB038
PpMYB38

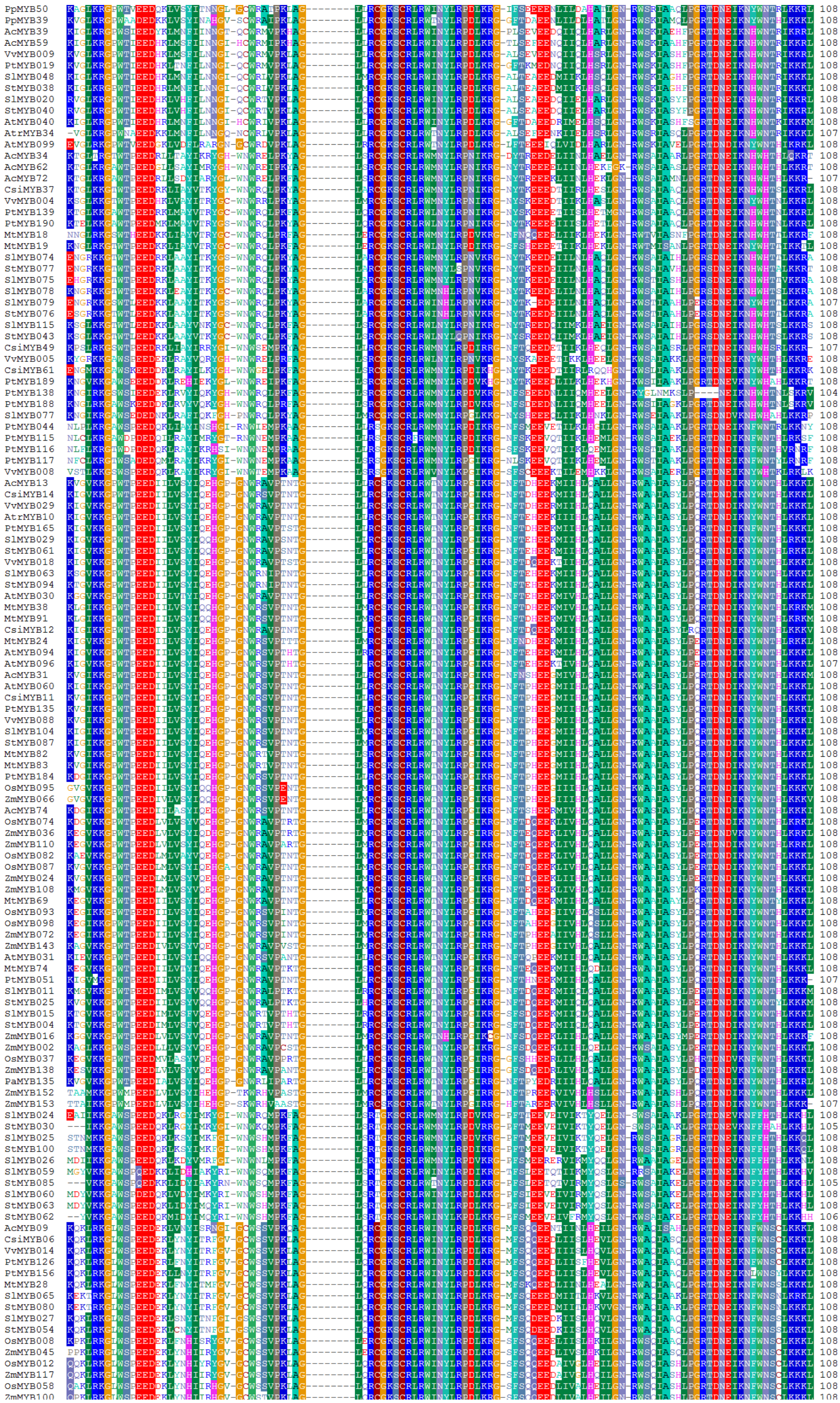

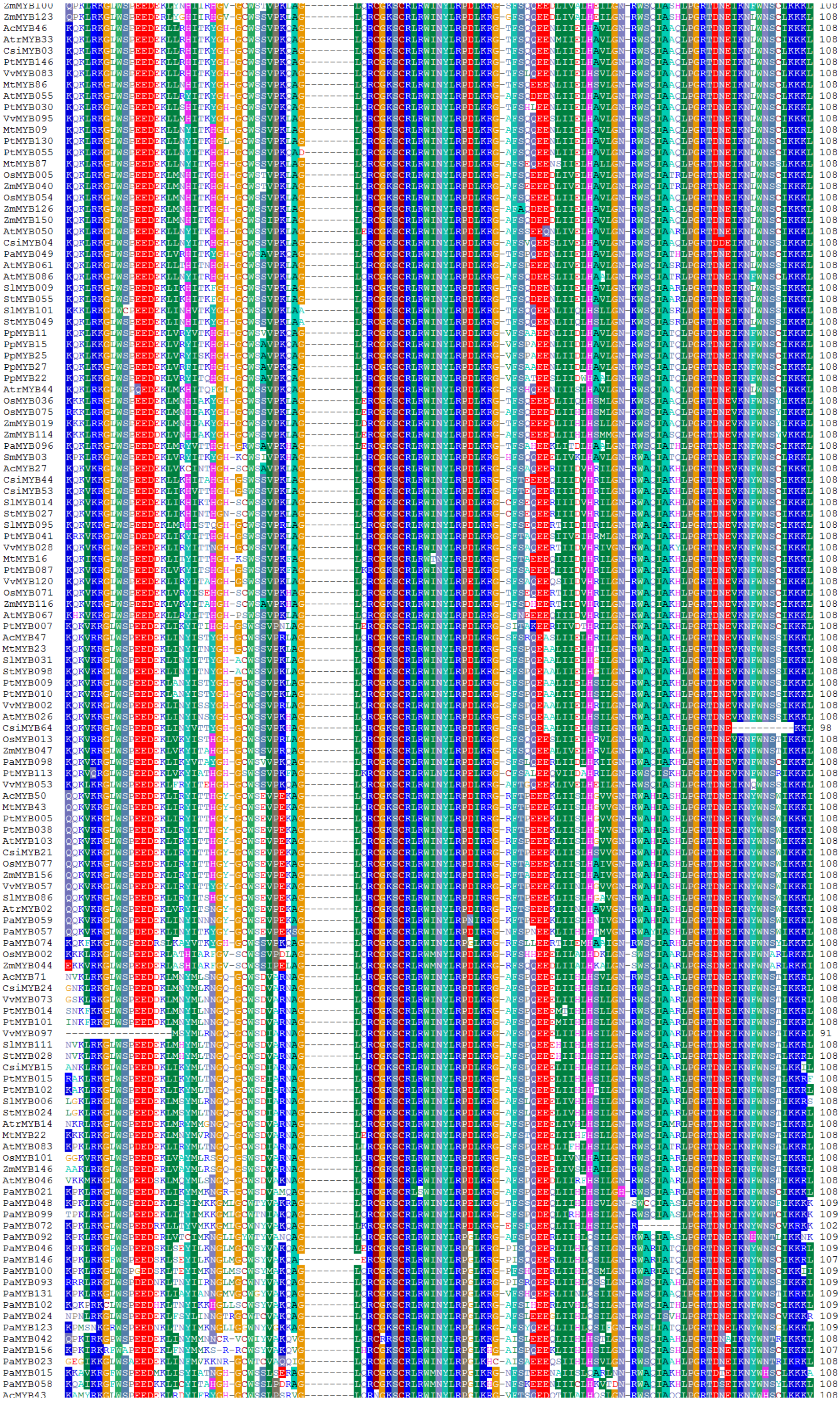
ZmMYB100 108
ZmMYB123 108
AcMYB46 108
AtrMYB33 108
CsiMYB03 108
PtMYB146 108
VvMYB083 108
MtMYB86 108
AtMYB055 108
PtMYB030 108
VvMYB095 108
MtMYB09 108
PtMYB130 108
PtMYB055 108
MtMYB87 108
OsMYB005 108
ZmMYB040 108
OsMYB054 108
ZmMYB126 108
ZmMYB150 108
AtMYB050 108
CsiMYB04 108
PaMYB049 108
AtMYB061 108
AtMYB086 108
SlMYB009 108
StMYB055 108
SlMYB101 108
StMYB049 108
PpMYB11 108
PpMYB15 108
PpMYB25 108
PpMYB27 108
PpMYB22 108
AtrMYB44 108
OsMYB036 108
OsMYB075 108
ZmMYB019 108
ZmMYB114 108
PaMYB096 108
SmMYB03 108
AcMYB27 108
CsiMYB44 108
CsiMYB53 108
SlMYB014 108
StMYB027 108
SlMYB095 108
PtMYB041 108
VvMYB028 108
MtMYB16 108
PtMYB087 108
VvMYB120 108
OsMYB071 108
ZmMYB116 108
AtMYB067 108
PtMYB007 108
AcMYB47 108
MtMYB23 108
SlMYB031 108
StMYB098 108
PtMYB009 108
PtMYB010 108
VvMYB002 108
AtMYB026 108
CsiMYB64 98
OsMYB013 108
ZmMYB047 108
PaMYB098 108
PtMYB113 108
VvMYB053 108
AcMYB50 108
MtMYB43 108
PtMYB005 108
PtMYB038 108
AtMYB103 108
CsiMYB21 108
OsMYB077 108
ZmMYB156 108
VvMYB057 108
SlMYB086 108
AtrMYB02 108
PaMYB059 108
PaMYB057 108
PaMYB074 108
OsMYB002 108
ZmMYB044 108
AcMYB71 108
CsiMYB24 108
VvMYB073 108
PtMYB014 108
PtMYB101 108
VvMYB097 91
SlMYB111 108
StMYB028 108
CsiMYB15 108
PtMYB015 108
PtMYB102 108
SlMYB006 108
StMYB024 108
AtrMYB14 108
MtMYB22 108
AtMYB083 108
OsMYB101 108
ZmMYB146 108
AtMYB046 108
PaMYB021 108
PaMYB048 109
PaMYB099 109
PaMYB072 102
PaMYB092 109
PaMYB046 109
PaMYB146 107
PaMYB100 109
PaMYB093 109
PaMYB131 109
PaMYB102 109
PaMYB024 109
PaMYB123 109
PaMYB042 108
PaMYB156 107
PaMYB023 108
PaMYB015 108
PaMYB058 108
AcMYB43 108

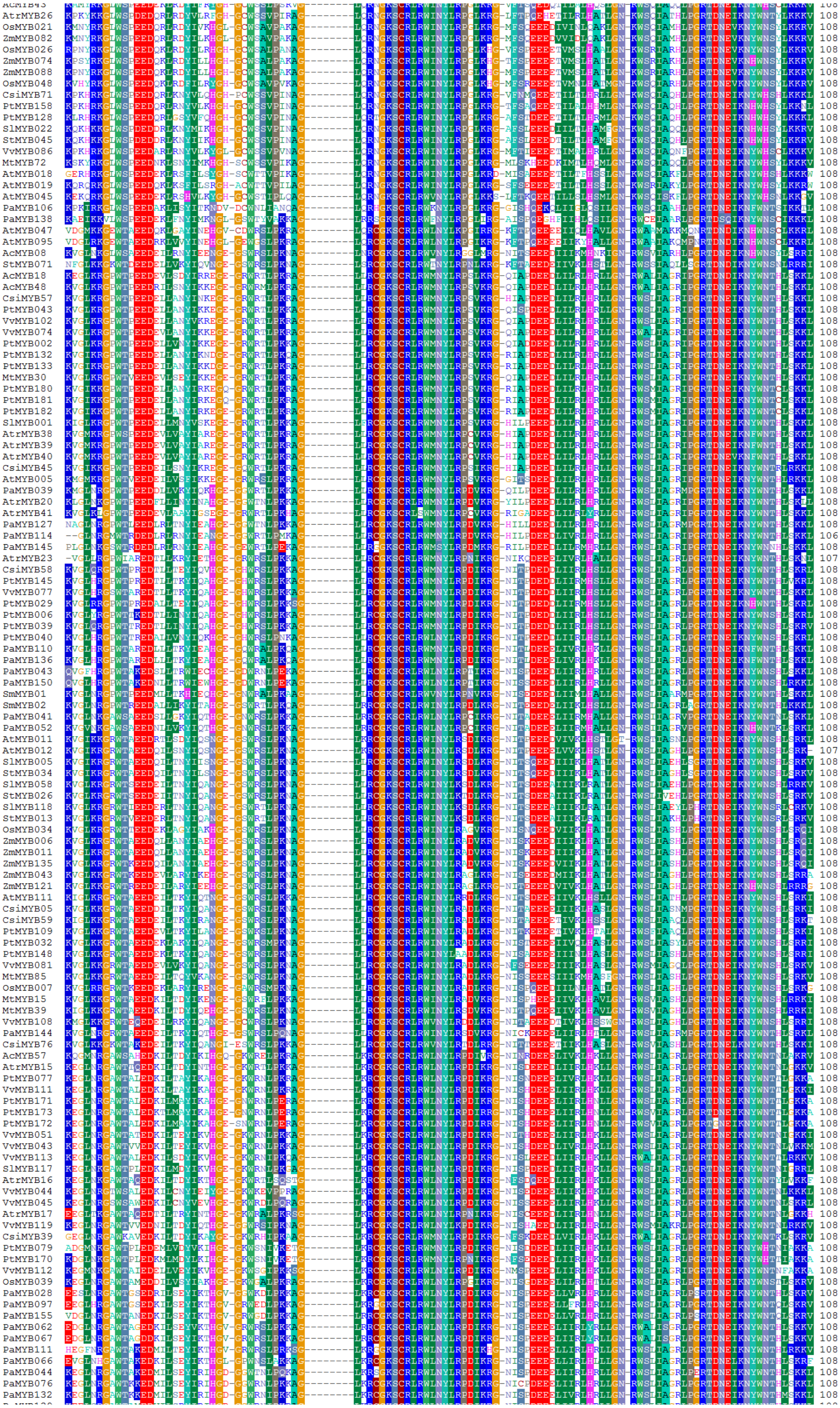
AtrMYB26
OsMYB021
ZmMYB082
OsMYB026
ZmMYB074
ZmMYB088
OsMYB048
CsiMYB71
PtMYB158
PtMYB128
SlMYB022
StMYB045
VvMYB086
MtMYB72
AtMYB018
AtMYB019
AtMYB045
PaMYB106
PaMYB138
AtMYB047
AtMYB095
AcMYB08
StMYB071
AcMYB18
AcMYB48
CsiMYB57
PtMYB043
VvMYB102
VvMYB074
PtMYB002
PtMYB132
PtMYB133
MtMYB30
PtMYB180
PtMYB181
PtMYB182
SlMYB001
AtrMYB38
AtrMYB39
AtrMYB40
CsiMYB45
AtMYB005
PaMYB039
AtrMYB20
AtrMYB41
PaMYB127
PaMYB114
PaMYB145
AtrMYB23
CsiMYB58
PtMYB145
VvMYB077
PtMYB029
PtMYB006
PtMYB039
PtMYB040
PaMYB110
PaMYB136
PaMYB043
PaMYB150
SmMYB01
SmMYB02
PaMYB041
PaMYB052
AtMYB011
AtMYB012
SlMYB005
StMYB034
SlMYB058
StMYB026
SlMYB118
StMYB013
OsMYB034
ZmMYB006
ZmMYB011
ZmMYB135
ZmMYB043
ZmMYB121
AtMYB111
CsiMYB05
CsiMYB59
PtMYB109
PtMYB032
PtMYB148
VvMYB081
MtMYB85
OsMYB007
MtMYB15
MtMYB39
VvMYB108
PaMYB144
CsiMYB76
AcMYB57
AtrMYB15
PtMYB077
VvMYB111
PtMYB171
PtMYB173
PtMYB172
VvMYB051
VvMYB043
VvMYB113
SlMYB117
AtrMYB16
VvMYB044
VvMYB045
AtrMYB17
VvMYB119
CsiMYB39
PtMYB079
PtMYB170
VvMYB112
OsMYB039
PaMYB028
PaMYB097
PaMYB155
PaMYB062
PaMYB067
PaMYB111
PaMYB066
PaMYB044
PaMYB076
PaMYB132

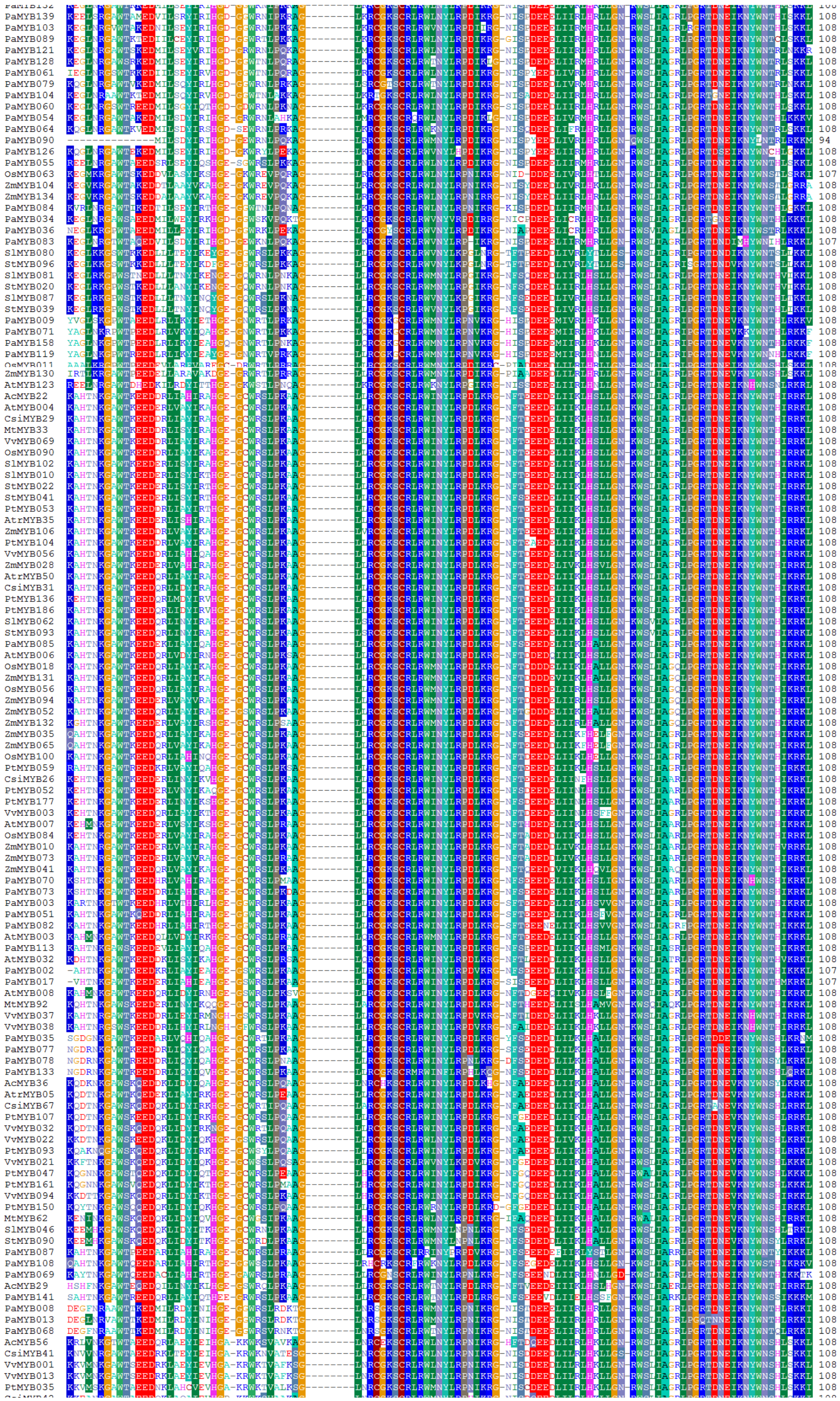

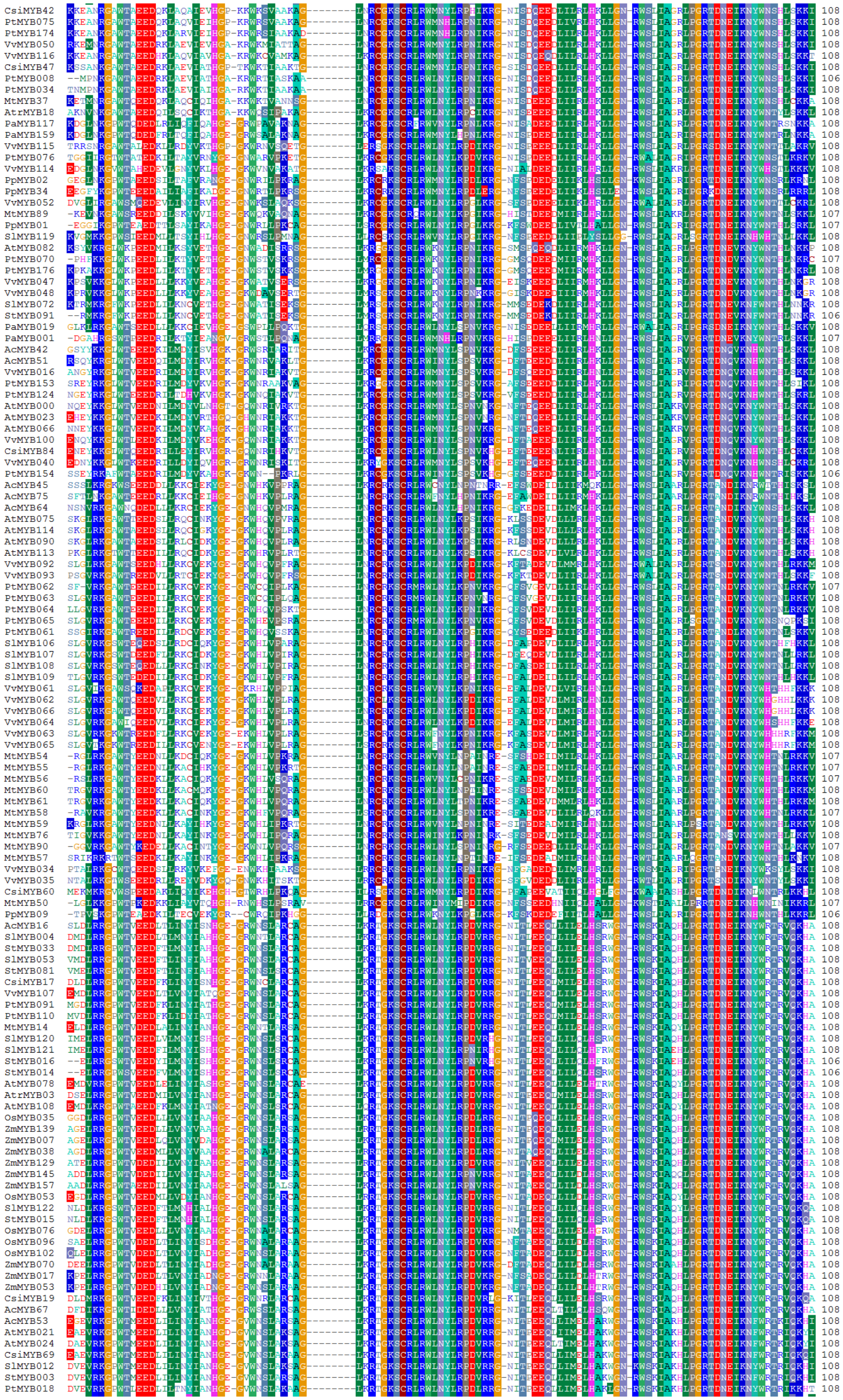
CsiMYB42 108
PtMYB075 108
PtMYB174 108
VvMYB050 108
VvMYB116 108
CsiMYB47 108
PtMYB008 106
PtMYB034 108
MtMYB37 108
AtrMYB18 108
PaMYB117 108
PaMYB159 108
VvMYB115 108
PtMYB076 108
VvMYB114 108
PpMYB02 108
PpMYB34 108
VvMYB052 108
MtMYB89 107
PpMYB01 107
SlMYB119 108
AtMYB082 108
PtMYB070 107
PtMYB176 108
VvMYB047 108
VvMYB048 108
SlMYB072 108
StMYB091 106
PaMYB019 108
PaMYB001 107
AcMYB42 108
AcMYB51 108
VvMYB016 108
PtMYB153 108
PtMYB124 108
AtMYB000 108
AtMYB023 108
AtMYB066 108
VvMYB100 108
CsiMYB84 108
VvMYB040 108
PtMYB154 106
AcMYB45 108
AcMYB75 108
AcMYB64 108
AtMYB075 108
AtMYB114 108
AtMYB090 108
AtMYB113 108
VvMYB092 108
VvMYB093 108
PtMYB062 107
PtMYB063 108
PtMYB064 108
PtMYB065 108
PtMYB061 108
SlMYB106 108
SlMYB107 108
SlMYB108 108
SlMYB109 108
VvMYB061 108
VvMYB062 108
VvMYB066 108
VvMYB064 108
VvMYB063 108
VvMYB065 108
MtMYB54 107
MtMYB55 107
MtMYB56 107
MtMYB60 108
MtMYB61 108
MtMYB58 107
MtMYB59 108
MtMYB76 108
MtMYB90 107
MtMYB57 108
VvMYB034 108
VvMYB035 108
CsiMYB60 108
MtMYB50 107
PpMYB09 106
AcMYB16 108
SlMYB004 108
StMYB033 108
SlMYB053 108
StMYB081 108
CsiMYB17 108
VvMYB107 108
PtMYB091 108
PtMYB110 108
MtMYB14 108
SlMYB120 108
SlMYB121 108
StMYB016 106
StMYB014 106
AtMYB078 108
AtrMYB03 108
AtMYB108 108
OsMYB035 108
ZmMYB139 108
ZmMYB007 108
ZmMYB038 108
ZmMYB129 108
ZmMYB145 108
ZmMYB157 108
OsMYB053 108
SlMYB122 108
StMYB015 108
OsMYB076 108
OsMYB096 108
OsMYB102 108
ZmMYB070 108
ZmMYB017 108
ZmMYB053 108
CsiMYB19 108
AcMYB67 108
AcMYB53 108
AtMYB021 108
AtMYB024 108
CsiMYB69 108
SlMYB012 108
StMYB003 108
PtMYB018 108

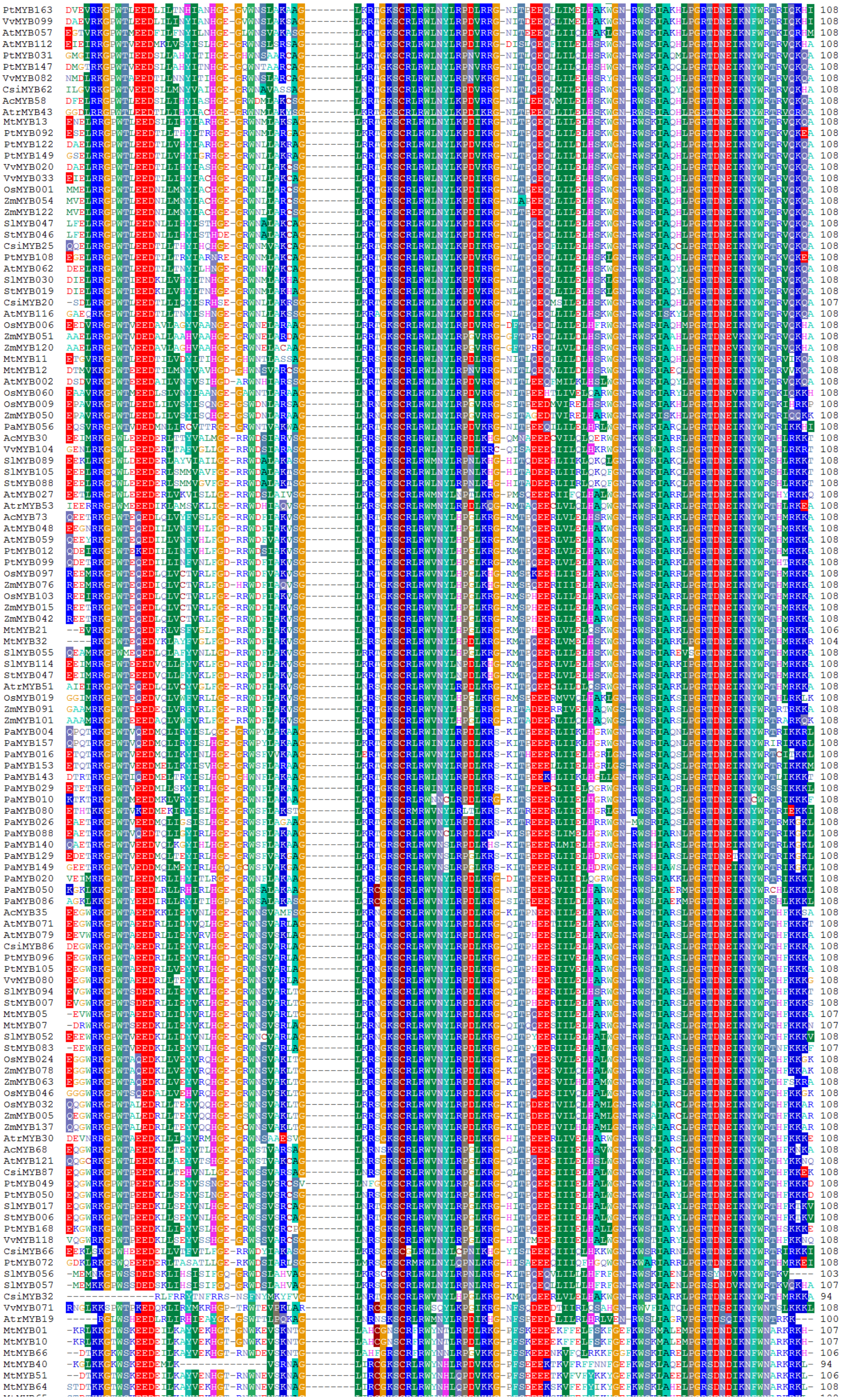

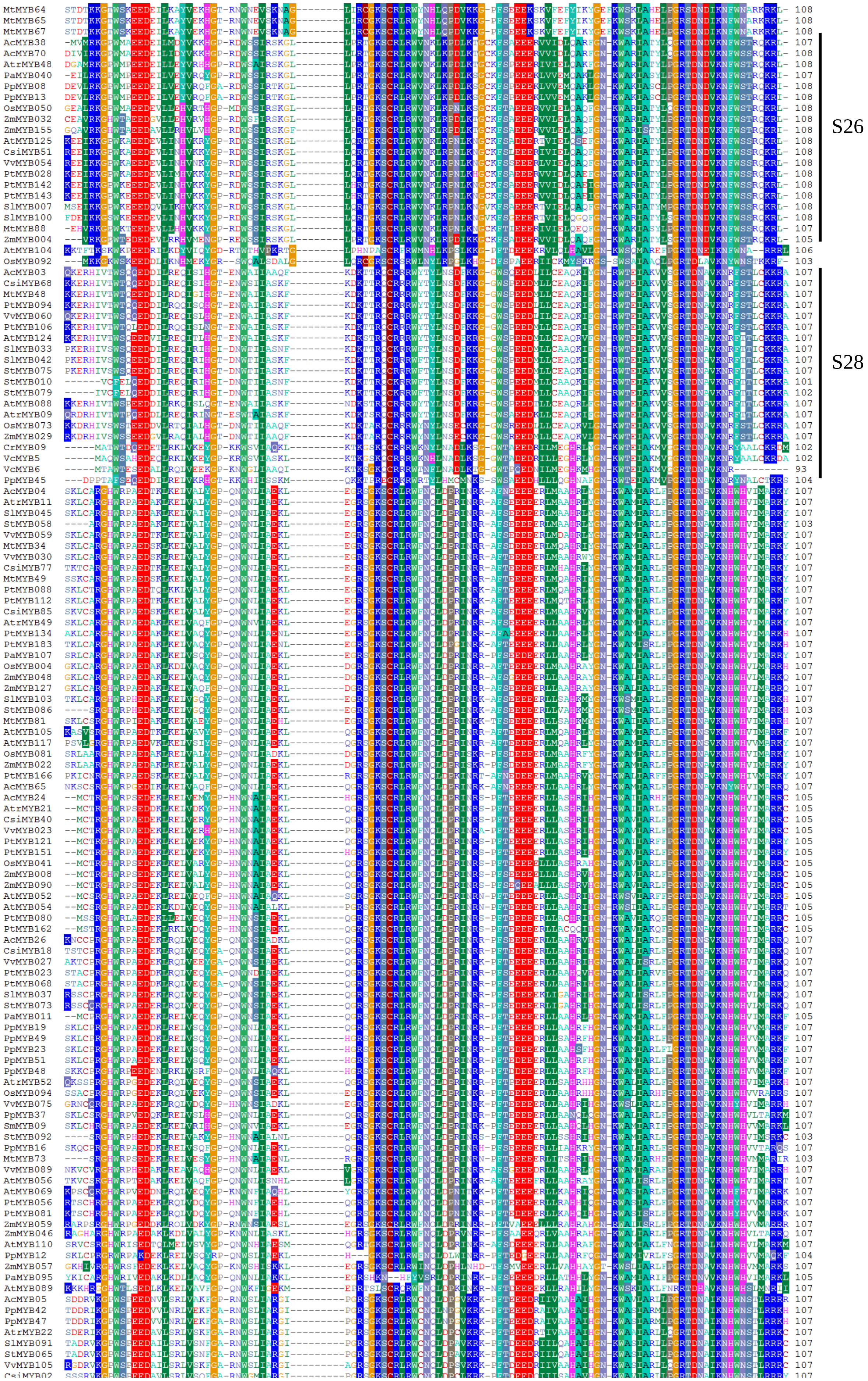
S26
S28

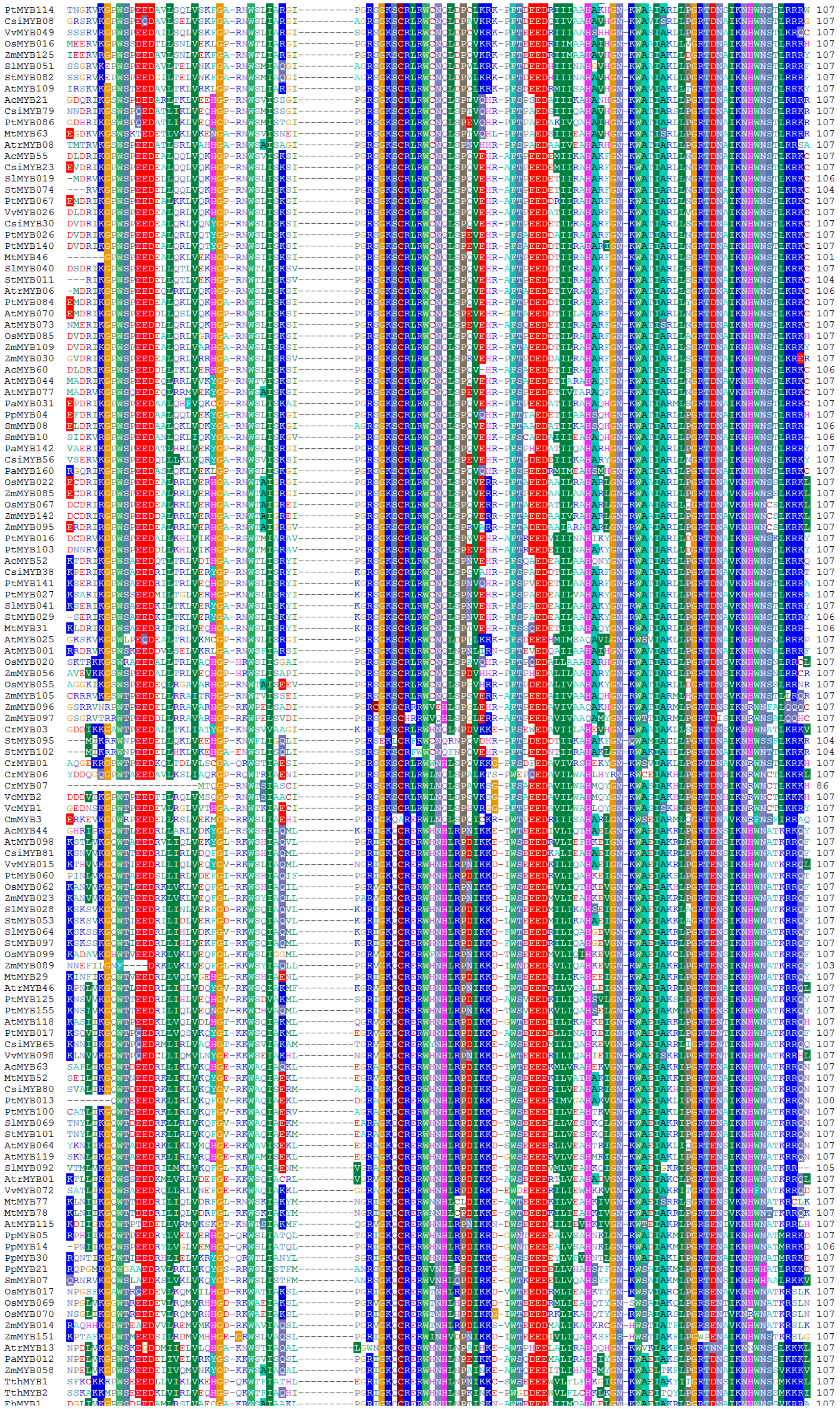

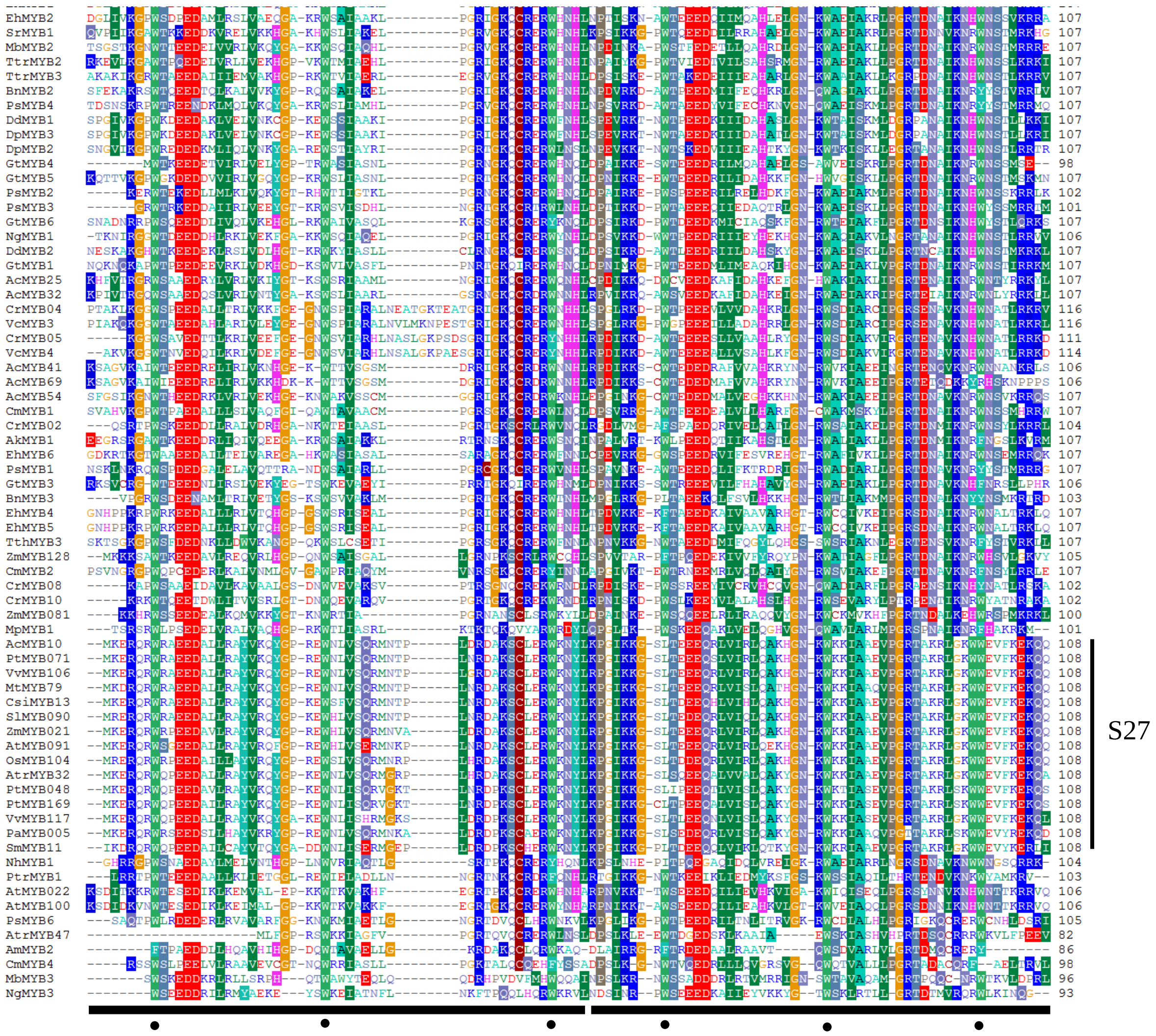

Supplementary Figure S2. Alignment of the MYB domains of all 2R-MYB proteins used in this study. Multiple alignment using Fast Fourier Transform (MAFFT) was used for the alignment of amino acid sequences of the 1548 eukaryote MYB domains. The shading of the alignment represents different degrees of conservation among sequences. Asterisks indicate conserved tryptophan residues (W), while black arrows indicate conserved residue insertions within the MYB domains. The detail subfamilies of the corresponding proteins belong to could be found in Supplementary Figure S1.

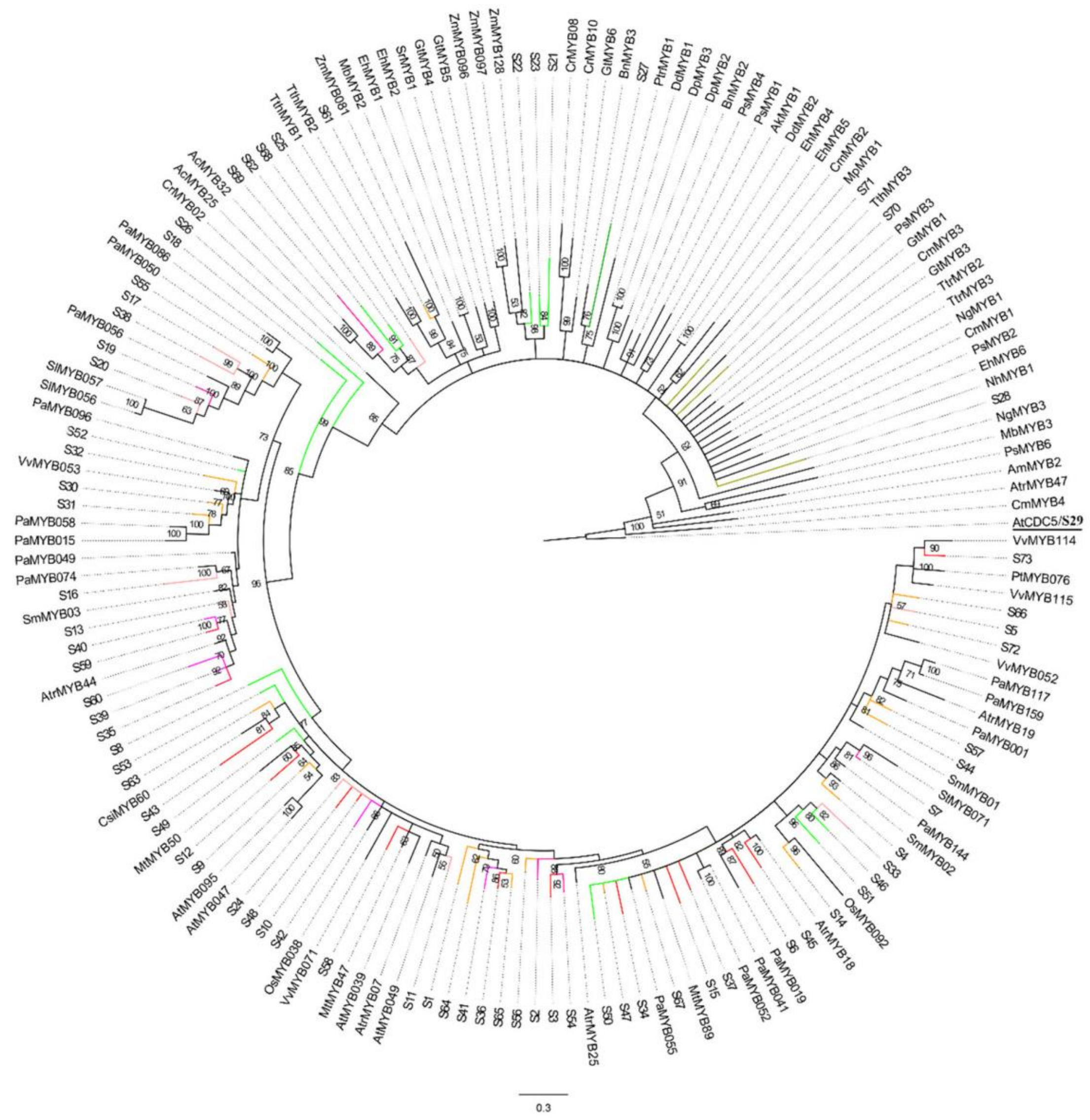

Supplementary Figure S3. Bayesian analysis of one representative from each subfamily of plant 2R-MYB genes (73 subfamilies) and all “orphan” genes The tree was rooted on a CDC5-like sequence (S29 subfamily). Numbers in each clade are posterior probability values, and clades with less than 50% support were collapsed. The colored lines symbolize the species to which the proteins in each subfamily belong, which are consistent with Figure 2.

Supplementary Table S1. The species used in this study and the conrresponding candidate 2R-MYB and 3R-MYB genes identified in each species.

Eukaryote	Lineage	Species	Genome ID NO.	Name	MYB-domain (protein sequence)
	Amoebozoa	*Dictyostelium purpureum*	XP_003286840.1	Dp2R-MYB1	MRGIKGGVWKNTEDEILKVAIMKYGLNQWARISSLLTRKSPAQCKARWYEWLDPNIKKTEWSKEEEEKLLHLAKIFPSQWKTIAPLVGRTSAQCLERYNKLLDEVQ
		Dictyostelium purpureum	XP_003293587.1	Dp2R-MYB2	SPGIVKGPWKDEEDAKLVELVNKCGPKEWSSIAAKIPGRIGKQCRERWFNHLSPEVRKTNWTAEEDKIIIDAHATLGNKWTAISKMLDGRPANAIKNHWNSTLLKRI
		Dictyostelium discoideum	XP_636296.1	Dd2R-MYB1	SPGIVKGPWKDEEDAKLVELVNKCGPKEWSSIAAKIPGRIGKQCRERWFNHLSPEVRKTNWTPEEDKIIIDAHASLGNKWTAISKMLDGRPANAIKNHWNSTLLKKI
		Dictyostelium discoideum	XP_001732948.1	Dd2R-MYB2	NESKAKGHWTKEEDEKLRSLVDLHGTKRWKYIASLLCLRNGRQCRERWSNQLDPSIKRDAWTLEEDRIILDAHSKYGNKWAEISKLLPGRTNCAIKNHWNSTMKRKL
		Dictyostelium discoideum	XP_641787.1	Dd2R-MYB3	MRNVKGGVWKNTEDEILKVAIMKYGLNQWARISSLLTRKSPAQCKARWHEWLDPSIKKTEWSKEEEEKLLHLAKIFPSQWKTIAPLVGRTASQCLERYNRLLDEVQ
		Dictyostelium discoideum	XP_628877.1	Dd3R-MYB1	EKKKTKGKWTSEEDQILIKAVNLHNQKNWKKIAEHFPDRTDVQCHHRYQKVLHPNLVKGAWTKDEDDKVIELVKTYGPKKWSDIALHLKGRMGKQCRERWHNHLNPNIKKEAWSDEEDQIIRDQHAIHGNKWAEIAKFLPGRTDNAIKNHWNSSMKRVS
	Apusozoa	*Thecamonas trahens ATCC 50062*	AMSG_11925	Ttr2R-MYB1	MRIYIKGGLWTNAEDEVLKASIMKYGLRNWARVASLFVRKSAKQCKARWYEWLDPSIKKTQWSREEEEKLLYLAKAMPMQWRTIAPLVGRTPAQCLEHYERLLSQAA
		Thecamonas trahens ATCC 50062	AMSG_05308	Ttr2R-MYB2	RKEVLKGAWTPQEDELVRLLVEKHGPVKWTMIAEHLPGRIGKQCRERWHNHINPAIYKGPWTVIEDTVILSAHSRMGNRWAEIAKLLPGRTDNAIKNHWNSSLKRKI
		Thecamonas trahens ATCC 50062	AMSG_09258	Ttr2R-MYB3	AKAKIKGRWTAEEDAIIIEMVAKHGPRKWNTVIAERLEGRVGKQCRERWHNHLDPSISKEPWTAKEDEIIIEAHARLGNKWAAIAKLLKGRPDNAIKNHWNSTLKRRV
		F. alba ATCC 38817 (V2)	H696_04435	Fa2R-MYB1	MTRIITKGGVWRNTEDEILKAAVMKYGLSQWARISSLLSRKTPKQCKARWENWLDPNIKKTEWSTEEEERLVHLATLMPCQWNTIAKQLNGRTADQCLEHYKSLQLAAV
	Fung	*Allomyces macrogynus ATCC 38327*	AMAG_19492.2	Am2R-MYB1	MRVVIKGGVWKNTEDEILKVAVMKYGKNQWARISSLLTRKTPKQCKARWYEWLDPSIKKTEWSKDEDEKLLHLAKLMPTQWRTIAPIVGRTPAQCLDRYQKLLDDAE
		Allomyces macrogynus ATCC 38327	AMAG_01769.2	Am2R-MYB2	YNERIGCKFTPAEDDLLHQAVHIHGPDQWTAVAELLGKRDAKQCLQRWKAQDLAIRRGRFTRDEDAALRAAVTQCGEKWSDVARLVLGRTDMQCRERY
		Allomyces macrogynus ATCC 38327	AMAG_04267.2	Am3R-MYB1	YNERIGRKFTPAEDDLLHQAVHIHGPDQWTSVADLLGTRDAKQCLQRWKAQDPAIRRGRFTRDEDAALRAAVTQCGEKWSDVARLVLGRTDMQCRERYYNVIKARMEVGPVPTGEWTAEEDAKLLELVAKEGTKWSSVAEQMPGRTDGMVRLRYAKLRTEIE
		Spizellomyces punctatus	SPPG_07992	Sp2R-MYB1	MRILIKGGVWKNTEDEILKAAVMKYGKNQWARISSLLTRKSPKQCKARWYEWLDASIKKTEWSKEEDERLLHLAKLMPTQWRTIAPMVGRTAAQCLERYQKLLDEAE
		Ustilago maydis	XP_760558.1	Um2R-MYB1	RVIIKGGVWKNTEDEILKAAISKYGKNQWARISSLLVRKTPKQCKARWYEWLDPSIKKTEWSKEEDEKLLHLAKLMPTQWRTIAPLVGRTANHCLERYQKLLDQAE
		Ustilago maydis	XP_760248.1	Um3R-MYB1	MAGKRNRWTRNEDEILKKAVLSSAKPRPSWAMIAKHIPGRSSKDCRKRWEHGLNQNLSRGPWSGSEDARLKAAVAEHGLHWALVATKVGKRTSDQCAKRWCDVLDPSLKKSEWTADEDSALLGLYQQLGTAWAKLATHIPGRSALSCRNRACKILVQRG
		Saccharomyces cerevisiae	NP_013940.1	Sc2R-MYB1	MPPVPIYVKGGVWTNVEDQILKAAVQKYGTHQWSKVASLLQKKTARQSELRWNEYLNPKLNFTEFSKEEDAQLLDLARELPNQWRTIADMMARPAQVCVERYNRLLESED
		Saccharomyces cerevisiae	NP_013025.1	Sc3R-MYB1	HRKNGRNSWSKDDDNMLRSLVNESAKELGYENGLEDVKTIQQSNHLSKCIAWDVLATRFKHTVRTSKDVRKRWTGSLDPNLKKGKWTQEEDEQLLKAYEEHGPHWLSISMDIPGRTEDQCAKRYIEVLGPGSKGRLREWTLEEDLNLISKVKAYGTKWRKISSEMEFRPSLTCRNRWRKIITMVV
		Nectria haematococca	XP_003047537.1	Nh2R-MYB1	MGHRRGPWSNAEDAYLMELVNTHGPLNWVRIAQTLGSRTPKQCRERYHQNLKPSLNHEPITPQEGAQIDQLVREIGKRWAEIARRLNGRSDNAVKNWWNGSQNRRK
		Nectria haematococca	XP_003046050.1	Nh2R-MYB2	MPVVKGGVWTNIEDEILKASVSKYGLNQWARVSSLLARKTPKQCKARWNEWLDPSIKKIEWSKDEDEKLLHLAKIMPTQWRTIAPIVGRTANQCLERYQKLLDEAE
		Nectria haematococca	XP_003048416.1	Nh3R-MYB1	HRRPWTSTEDSALRTLVAHFGDSRGPEGRWKDIAAGLQGRTAKDCRKRWFHSLDPSLRKGRWTAQEDELLLSAYARLGPAWHDIALLIPGRKDDQCSKRYNDILNPSAQNRLSDWTAQEDNLLRQGVATLGHRWAAISSRIPGRPPLTCRNRWRTLSRQ
	Ichthyosporea	*S. arctica JP610*	SARC_05959	Sa2R-MYB1	MRMIVKGGVWKNTEDEILKAAVMKYGKNQWARIASLLHRKTAKQCKARWYEWLDPSIKKTEWTREEEEKLLHLAKLFPTQWRTIAPMIGRTGGQCLAHYEYLLDQAQ
		S. arctica JP610	SARC_01616.1	Sa3R-MYB1	KSTKRLKQWTKEEDKILREVVDRYGEKFDMATQYLTHRSEGQIAQRWLKVLSPDIVKGAWTPEEDAKVIELIKENGTKDWSLISGFLPGRVGKQCRERWHNHLNPEVRKGPWTSEEEGTIIEAQARLGNKWSQISALIPGRTDNAIKNYWNSTIKKKV
	Filasterea	*C. owczarzaki ATCC 30864*	CAOG_00996	Co2R-MYB1	MPRVLIKGGVWKNTEDEILKAAVMKYGMNQWSRIASLLHRKTAKQCKARWYEWLDPSIKKTEWSREEEEKLLHLAKLMPTQWRTIAPMIGRTAAQCLEHYERLLDQAQ
		C. owczarzaki ATCC 30864	CAOG_00301.2	Co3R-MYB1	EDEPDAAPWTDKQDAELRYCVDSWDVRKDWTVIAARIPPHTAMQCARRWQKLNPQIVRGSWTPEEDLRVIQLVDQIGACQWPIIASFLIGRIGKQCRERYHNHLNPNIKKDPWSTEEDNLLLELHEQFGNKWAEIAKHMEGRTDNAIKNRYNSTVSRRA
	Choanoflagellata	*M. brevicollis MX1*	MONBRDRAFT_33087	Mb2R-MYB1	MPPVFIKGGVWTNVEDEVLKAAVMKYGPNQWDRVASLLHRKSAKQCKYRWYEWLDPSIKKTEWSREEEEKLLHLAKTMSSQWRTIAPMIGRTPIQCLERYEKLLDQAQ
		M. brevicollis MX1	MONBRDRAFT_33265	Mb2R-MYB2	TSGSTKGNWTTEEDELVVRLVKQYGAKKWSQIAQHLPGRVGKQCRERWHNHLNPDINKAPWSTFEDETLLQAHRDLGNKWAEIAKLLPGRTDNAIKNRWNSTMRRRE
		M. brevicollis MX1	MONBRDRAFT_22022	Mb2R-MYB3	SWSKEDDKRLRELVLSRPHQTWAWYTEQLQQDRHPVDVFMHWQQAINPSLVKRNWSSADDDRLRTVMRRIGNSDWTAVAQAMGDRTPQQCNLRWTKVLDPRL
		M. brevicollis MX1	MONBRDRAFT_27866.2	Mb3R-MYB1	KKKTTTLRWGSDEDRRLRDLVQQRQAKTTLSIDDDHFWLAIAQNMPGRDASHCAARWKNMLDPTLIKGAWTQEEDDLVINLVNKYGPCNWTKIAQHLKGRIGKQCRERWHNALAPHLKRGPWSEDEKRTLIDAHARLGNRWAEISKLLPGRTDNHCKNFWNSMKTKKV
		M. brevicollis MX1	MONBRDRAFT_16076.2	Mb3R-MYB2	GRSHSTRRWTKAEDQRLVEAVERHQGKNWKKVAEEFEGRTDVQCLHRWQKVLNPDLVKGPWTKEEDDLVIQLVDKYGPKRWSLIAGHLKGRIGKQCRERWHNHLHPDIKKTPWTAEEERVIMNAHLRLGNKWAEIAKLLPGRTDNSVKNHWNSTMRRR
		S. rosetta	PTSG_12328	Sr2R-MYB1	QVPIIKGAWTKKEDDKVRELVKKHGAKHWSLIAKELPGRVGKQCRERWHNHLKPSIKKGPWTQEEDDILRRAHAELGNKWAEIAKRLPGRTDNNVKNRWNSTMRKHG
		S. rosetta	PTSG_09300	Sr2R-MYB2	MSARIFIKGGVWKNTEDEILKAAVMKYGPNQWDRVASLLHRKSAAQCKRRWYEWLDPSIKKTEWSREEEEKLLHLAKLMPTQWMTFAPMIGRTASQCLEHYEKLLDQAQ
		S. rosetta	PTSG_00973.1	Sr3R-MYB1	RWTKEEDSRLKTLVKQFEAEGITDDTTLWQKVAEQMPGRDCSHCAHRWKNMLDPSLVKGAWSKEEDAKVVELVKIYGPRNWSKIAQHLKGRIGKQCRERWHNTLNPDLKRGPWSEEEQRILEEAHARLGNKWAAIAKLLPGRTDNHIKNHWNSMMAKQA
		S. rosetta	PTSG_08337.1	Sr3R-MYB2	QMQKQSKSWLPEEDTILRDAITLYGTDNWSAVSNLFYARSQNQCLQRWEKMHNPTIQKGPWTKEEDTLLHQLVQEHGAKNWSEIAKKMKTRVGKQCRERWHNHLDPSVKKGAWSDDEERILLDAHKKLGNKWAEIAKLLPGRTDNAVKNHWNSNMRRRKL
	Metazoa	*Nematostella vectensis*	estExt_gwp.C_1220079	Nv2R-MYB1	MPRIIIKGGVWRNTEDEILKAAVMKYGKNQWSRIASLLHRKSAKQCKARWYEWLDPSIKKTEWSREEDEKLLHLAKLMPTQWRTIAPLIGRTAAQCLERYEYLLDQAQ
		Daphnia pulex v1	stExt_Genewise1.C_40100	Dap2R-MYB1	MPRIMIKGGVWRNTEDEILKAAVMKYGKNQWSRIASLLHRKSAKQCKARWYEWLDPSIKKTEWSREEDEKLLHLAKLMPTQWRTIAPIVGRTAAQCLERYEYLLDQAQ
		Lottia gigantea	estExt_Genewise1.C_sca_340094	Lg2R-MYB1	MPRIMIKGGVWRNTEDEILKAAVMKYGKNQWARIASLLHRKSSKQCKARWYEWLDPSIKKTEWSREEEEKLLHLAKLMPTQWRTIAPIIGRTAAQCLEHYEYLLDKAQ
		Caenorhabditis elegans	NP_492303.1	Ce2R-MYB1	RVIIKGGVWKNTEDEILKAAIMKYGKNQWSRIASLLHRSAKQCKARWFEWLDPGIKKTEWSREEDEKLLHLAKLMPTQWRTIAPGRTSAQCLERYEHLLDEAQ
		Drosophila melanogaster	NP_612033.1	Dm2R-MYB1	MPRIMIKGGVWRNTEDEILKAAVMKYGKNQWSRIASLLHRKSAKQCKARWYEWLDPSIKKTEWSREEDEKLLHLAKLMPTQWRTIAPIIGRTAAQCLERYEYLLDQAQ
		Drosophila melanogaster	NP_511170.1	Dm3R-MYB1	RWSKSEDVLLKQLVETHGENWEIIGPHFKDRLEQQVQQRWAKVLNPELIKGPWTRDEDDMVIKLVRNFGPKKWTLIARYLNGRIGKQCRERWHNHLNPNIKKTAWTEKEDEIIYQAHLELGNQWAKIAKRLPGRTDNAIKNHWNSTMRRKY
		Homo sapiens	NP_001244.1	Hs2R-MYB1	MPRIMIKGGVWRNTEDEILKAAVMKYGKNQWSRIASLLHRKSAKQCKARWYEWLDPSIKKTEWSREEEEKLLHLAKLMPTQWRTIAPIIGRTAAQCLEHYEFLLDKAA
Unikonts		*Homo sapiens*	NP_001155128.1	HsC-MYB	KRHLGKTRWTREEDEKLKKLVEQNGTDDWKVIANYLPNRTDVQCQHRWQKVLNPELIKGPWTKEEDQRVIELVQKYGPKRWSVIAKHLKGRIGKQCRERWHNHLNPEVKKTSWTEEEDRIIYQAHKRLGNRWAEIAKLLPGRTDNAIKNHWNSTMRRKV

		Homo sapiens	NP_001073885.1	HsA-MYB	KKLWNRVKWTRDEDDKLKKLVEQHGTDDWTLIASHLQNRSDFQCQHRWQKVLNPELIKGPWTKEEDQRVIELVQKYGPKRWSLIAKHLKGRIGKQCRERWHNHLNPEVKKSSWTEEEDRIIYEAHKRLGNRWAEIAKLLPGRTDNSIKNHWNSTMRRKV
		Homo sapiens	NP_002457.1	HsB-MYB	RDSKCKVKWTHEEDEQLRALVRQFGQQDWKFLASHFPNRTDQQCQYRWLRVLNPDLVKGPWTKEEDQKVIELVKKYGTKQWTLIAKHLKGRLGKQCRERWHNHLNPEVKKSCWTEEEDRIICEAHKVLGNRWAEIAKMLPGRTDNAVKNHWNSTIKRKV
		Amphimedon queenslandica	XP_003390703.1	Aq2R-MYB1	MPRIMIKGGIWKNTEDEILKAAVMKYGKNQWARIASLLHKKSAKQCKARWYEWLDPSIKKTEWSRDEEEKLLHLAKLMPCQWRTIAPIVGRTPAQCLEHYEYLLDKVQ
	Excavata	*Naegleria gruberi*	estExt_fgeneshHS_pg.C_360048	Ng2R-MYB1	TKNIRGGWTDEEDDHLRKLVEKFGAKKWSQIAQELPGRIGKQCRERWYNHLDPSVKKDWWTPEEDRIIIEYHEKHGNKWAQIAKVLNGRTANAIKNHWNSTLRRVV
		Naegleria gruberi	fgeneshHS_kg.scaffold_62000007	Ng2R-MYB2	KQFVRGGPWKNSEDEILKAAVMKYGKNQWARVASLLNRKSAKQCKARWYEWLDPSIKKTEWTREEEEKLLHLAKIMPSQWKSIAPIVGRTASQCLEHYEQMLDEEQ
		Naegleria gruberi	estExt_fgeneshNG_pg.C_350171	Ng2R-MYB3	SKRKQQVKWSEEDDRILRMYAEKEYSWKEIATNFLNDKFTPQQLHQRWKRVLNDSINREPWSEEEDKAIIEYVKKYGCTWSKLRTLLGTQRTDTMVRQRWLKITNGQG
		Naegleria gruberi	e_gw1.14.170.1	Ng3R-MYB1	MLRAAVQTYGGKNWKKIAETLQNRTSVQCLHRWQKVLNPNLVKGPWTKEEDDKILQLVKTYGPENWSMIASHLPGRIGKQCRERWYNHLDPSIKKEPWTEEEETLLLDAQSKLGNKWAEISKLIVGRTDNACKNHFNSLIAREKKK
		Trypanosoma brucei	XP_844880.1	Tb2R-MYB1	MTRLHFKGGAWTNSEDQVLRASLTVYGLQNWERVASTLVKKTATQCRERWENFLDPRLNINEAWTAEEEENLVQLQSLFPNQWSLISQEVSRRCGMNRPAWLCEEQYHSLLDA
		Leishmania major	|XP_001682333.1	Lm2R-MYB1	MSRLHFKGGAWTNAEDEVMKAALAQYGLRDWERVASMLTKKTATQCRERWENYLDPRLNIHEAWSLEEEEKLVELQALFPHKWRLISEQLTRRTPNHYIRPAWLCEQRYLELKDEQE
	Haptophyta	*Emiliania huxleyi CCMP1516*	estExtDG_Genewise1Plus.C_4620005	Eh2R-MYB1	DGLIVKGPWSDPEDAMLRSLVAEQGAKRWSAIAAKLPGRIGKQCRERWHNHLNPTISKNAWTEEEDQIIMQAHLELGNKWAEIAKRLPGRTDNAIKNHWNSSVKRRA
		Emiliania huxleyi CCMP1516	estExtDG_Genewise1.C_1310030	Eh2R-MYB2	DGLIVKGPWSDPEDAMLRSLVAEQGAKRWSAIAAKLPGRIGKQCRERWHNHLNPTISKNAWTEEEDQIIMQAHLELGNKWAEIAKRLPGRTDNAIKNHWNSSVKRRA
		Emiliania huxleyi CCMP1516	estExtDG_fgenesh_newKGs_kg.C_2520018	Eh2R-MYB3	MRILIKGGVWKNTEDEILKAAVMKYGKNQWSRISSLLVRKSAKQCKARWHEWLDPSIKKTEWSREEEEKLLHLAKLMPTQWRTIAPIVGRTAAQCLEHYERLLDQAT
		Emiliania huxleyi CCMP1516	estExtDG_fgenesh_newKGs_kg.C_1550001	Eh2R-MYB4	GNHPPKRPWRKEEDALLLRLVTQHGPGSWSRISEALPGRIGKQCRERWHNHLTPDVKKEKFTAEEDKAIVAAVARHGTRWCQIVKEIPGRSDNAIKNRWNALTRKLQ
		Emiliania huxleyi CCMP1516	estExtDG_fgenesh_newKGs_kg.C_220091	Eh2R-MYB5	GNHPPKRPWRKEEDALLLRLVTQHGPGSWSRISEALPGRIGKQCRERWHNHLTPDVKKEKFTAEEDKAIVAAVARHGTRWCQIVKEIPGRSDNAIKNRWNALTRKLQ
		Emiliania huxleyi CCMP1516	e_gw1.60.8.1	Eh2R-MYB6	GDKRTKGTWAAEEDAILTELVAREGAHKWASIASALSARGGVERAGKQCRERWFNNLCPEVRKGGWSPEEDRVIFESVREHGTRWAFIVKLLPGRTDNAIKNRWNSEMRRQK
	Rhizaria	*Bigelowiella natans CCMP2755*	estExt_Genewise1.C_230192	Bn2R-MYB1	MRIMIKGGVWKNTEDEILKAAVMKYGKNQWSRISSLLVRKSAKQCKARWFEWLDPSIKKTEWTREEEEKLLHLAKIMPTQWRTIAPIIGRTSSQCLERYEKLLDAAQ
		Bigelowiella natans CCMP2755	estExt_fgenesh1_pg.C_630109	Bn2R-MYB2	SFEKAKRSWTQEEDTQLKALVVKYGPRQWSAIAKELPGRIGKQCRERWHNHLNPDVRKDAWTPEEDMIIFEQHKRLGNQWAGIAKLLPGRTDNAIKNRYYSTVRRLV
		Bigelowiella natans CCMP2755	estExt_Genewise1.C_470048	Bn2R-MYB3	VPGRKMTWSDEENAMLTRLVETYGSKSWSVVAKLMPGRTGKQCRERWTNHLMPGLRKGPLTAEEKQLFSVLHKKHGNRWTLIAKMMPGRTDNALKNYYNSMKRTRD
		Bigelowiella natans CCMP2755	gw1.5.185.1	Bn3R-MYB1	MRRKWTDQEDTLLETLVKKHSAHWSLIAGKMHRRSAAGCKRRWEQVLNVQVKKGRFTEEEDKKICKHVQENGATNWGLLSFSMPQRTSVQCRTRWFEGLDPRLKKSPWSSQEDRLICKYHGILGPRWSCIAEHVKGRTAKAIREHFMQSLQQR
	Stramenopiles	*Aplanochytrium kerguelense PBS07*	estExt_fgenesh1_pg.C_70341	Ak2R-MYB1	EEGRSRGAWTKEEDDRLIQIVQEEGAKRWSAIAKKLRTGRNSKQCRERWSNQINPALVRTKWLPEEDQTIIKAHSTLGNRWALIAKLLPGRTDNMIKNRFNGSLKVRM
		Aplanochytrium kerguelense PBS07	e_gw1.25.266.1	Ak2R-MYB2	RIMIKGGVWKNAEDEILKAAVMKYGKNHWARVASLLSRKSAKQCKARWHEWLDPSIKKTEWTREEDEKLLHLAKLMPTQWRTIAPAVGRTAPQCLERYEKLLDMAQ
		Aplanochytrium kerguelense PBS07	e_gw1.14.209.1	Ak3R-MYB1	MRWSCEEDDLLRKAVDIVGARNWKRISREFMKGKRTDVQCLHRWQKVLRPGLIKGPWTEEEDKTILRAIKSGMTKWSEIANQVPGRIGKQCRERWFNHLDPSIKKGSWSKSEDKILYESHHKFGNRWCEIAKLLPGRSENAVKNRWNSTVRKQ
		Aplanochytrium kerguelense PBS07	e_gw1.19.197.1	Ak3R-MYB2	MLLALVDKFGAKNWSFIAAHLEGRTEIQCLQHWRNVLNPDLIKGSWTSEEDELLRKTVERYGPKKWCAVIAPNLPGRMGKQCRERWFNTLDPSLKKGPWSAKEEETLKEAHAKYGNKWSLISKLLPGRSNNDIKNHFHASLRRTK
		Aplanochytrium kerguelense PBS07	estExt_Genewise1Plus.C_80210	Ak3R-MYB3	VPIRKSRPWSVEEDLLLKRAVIQKGPSRWTAVIAPLVPGKTTAEQCKQHWECVLKFQLTKGRGTWTDEEDEKLSLLVRQLGSKSWKEVAKQLPGRIGKQCRERWINFLDPSLKKTSWTVREKEQLLRLHQEYGGKWSKIAKCIPGRSDNSCKNCWNSMQHRTK
		Phytophthora sojae	gw1.8.4384.1	Ps2R-MYB1	NSKLNKRQWSPDEDGALELAVQTTRANDWSAIARLLPGRCGKQCRERWVNHLSPAVNKEAWTEEEDQLIFKTRDRIGNRWADIARLLPGRTDNAVKNRYYSTMRRRG
		Phytophthora sojae	estExt_Genemark1.C_1_t10388	Ps2R-MYB2	KERKPVCKWTEKEDLLMLKLVQKYGTRHWTIIGTKLPGRNGKQCRERWHNQLDPAIRKEPWSPEEERILRELHDKFGNKWAEIAKMLPGRTDNAIKNHWNSSKRRLK
		Phytophthora sojae	fgenesh1_pg.1_#_347	Ps2R-MYB3	GSTYERRAWTRKEDDAIIRLVEEYGTKRWSVISDHLNGENHGTERTGKQCRTRWLNHLDPTIKKDPWTAEEEQIIEDAQTRLGNKWAEISKLLPGRTDNAIKNHWYSSMRRTM
		Phytophthora sojae	estExt_fgenesh1_pm.C_2_t30351	Ps2R-MYB4	TDSNSKRPWTREENDKLMQLVKQYGAKRWSLIAMHLPGRVGKQCRERWHNHLNPSVRKDAWTAEEDYVIFECHKNVGNQWAEISKMLPGRTDNAIKNRYYSTMRRMQ
		Phytophthora sojae	e_gw1.3.6293.1	Ps2R-MYB5	MRIMIKGGVWKNTEDEILKAAVMKYGMNQWARVASLLSRKSAKQCKARWYEWLDPSIKKTEWSREEEEKLLHLAKLMPSQWRTIAPIVGRTAAQCMEHYERLLDAAQ
		Phytophthora sojae	fgenesh1_pm.8_#_308	Ps2R-MYB6	GSAQTPTKWLRDEDERLRVAVARFGGKNWKMIAETLGNGRTDVQCLHRWNKVLKPGLIKGPWTPEEDRILTNLITRYGVGKIRWCDLALHLPGRIGKQCRERWCNHLDSRI
		Phaeodactylum tricornutum	fgenesh1_pg.C_chr_1000784	Ptr2R-MYB1	SLRRTPKRWTEEEDAALLKLIETGGLREAAERGLPEVWIELADLLNNGRTNKQCRDRFQNHLRTGIKKGNWTKEEIKLIEDMYKSFGSKWSSIAQILTHRTENDVKNKWYAMKRVEE
	Alveolata	*Plasmodium falciparum*	XP_001347611.1	Pf2R-MYB1	RIQIKGGIWKNCEDEVLKAAVMKYGLNNWSRVASLLVRKSAKQCKARWYEWLDPSVRKTEWNKEEEEKLLHLAKLFPTQWRTIAPIVGRTAQQCLEHYEYLLDEAE
		Tetrahymena thermophila	XP_001019592.1	Tth2R-MYB1	SFKCKKRPWSEEEDLLVIKLVKEHGPQKWTFIATHLEGRIGKQCRERWHNHLNPNIKKCSWSEEEEWVLYLFHKGIGNKWAEIAKYIIGRTDNSIKNHWNSGMKKRL
		Tetrahymena thermophila	XP_001019508.1	Tth2R-MYB2	SSKAKKMPWSEEEDKLVIRLVEQHGPQKWTFIAQHIPGRIGKQCRERWHNHLNPKINKEPWGDDEEWVLFLCHKLKGNKWAEITQYLPGRTDNAIKNHWNSSMKKRI
		Tetrahymena thermophila	XP_001017510.1	Tth2R-MYB3	MISKTSGKGPWSFDEDNKLLDWVKANGPQKWSLCSETIPGRSGKQCRERWFNNLNPNVKKGNWTAEEDDMIFQGYLQHGSSWSRIAKNLEGRTENSVKNRFYSTVRKLL
		Tetrahymena thermophila	XP_001027756.2	Tth2R-MYB4	GVWKNVEDEILKAAVMKYGLNQWSRISSLLIRKSAKQCKQRWYEWLDPQIKKTEWTREEDEKLLHLAKIFPCQWRTIAPLVNRTPNQCVERYERLLDIAQ
	Cryptophyta	*Guillardia theta CCMP27*	e_gw1.65.33.1	Gt2R-MYB1	MNQKNGQKAPWTPEEDEEVRKLVDKHGDKSWVLVASFLPNRTGKQIRERWHNQLDPNIMKGPWTEEEDMLIMEAQKIHGNKWAEIAKLVPGRTDNAIKNRWNSTIRRKM
		Guillardia theta CCMP27	estExt_fgenesh2_pg.C_680082	Gt2R-MYB2	MRIMIKGGVWKNTEDEILKAAVMKYGKNQWSRIASLLSRKSAKQCKARWYEWLDPSIKKTEWTREEDEKLLHLAKIMPTQWRTIAPIVGRTAAQCLERYEKLLDEAM
		Guillardia theta CCMP27	e_gw1.48.74.1	Gt2R-MYB3	MGRLKDKSVCRGWTEEEDNLIRSLVEKYEGTSWKEVAEYIPRRTGKQIRERWHNMLDPNIKKSSWTREEEVILFHAHAVYGNRWAEIAKLLPGRTDNAVKNHFNRSLLPHR
		Guillardia theta CCMP27	fgenesh2_pm.63_#_2	Gt2R-MYB4	MWTKEEDETVIRLVELYGPTRWASIASNLPGRNGKQCRERWHNQLDPAIKKESWTEEEDRILMQAHAELGSAWVEISKRLPGRTDNAIKNRWNSSMSE
		Guillardia theta CCMP27	e_gw1.20.104.1	Gt2R-MYB5	KQTTVKGPWGKDEDDVVIRLVGQYGPKRWSLIASNLPGRTGKQCRERWHNQLDPNIKREEWTEEEDRILIDAHKKASSLLAFGNHWVGISKLLPGRTDNAIKNRWNSTMSKMN
		Guillardia theta CCMP27	au.8_g2874	Gt2R-MYB6	SNADNRRPWSQEEDDLIVQLVKEHGLRKWAIVASQLKGRSGKQCRERYKNQLDPSIRKDPWTDEEDKMICIAQSKFGNRWTEIAKFLPGRTDNSIKNHWYSTLQRKS
Bikonts		*Guillardia theta CCMP27*	e_gw1.7.354.1	Gt3R-MYB1	AVRGSKAKWTAEEDSRLAALVDQFHAKNWKKIAERMEGRTDVQCLHRWQKVLNPDLVKGPWTKEEDRTVIELVSQHGPKKWSLIASHLPGRIGKQCRERWHNHLNPDIKKEEWTPLEDSIIIHAHRVYGTKWAKIAALLPGRTDNAIKNHWNSTMKRR

Rhodophyta	*Cyanidioschyzon merolae*	CMJ282C	Cm2R-MYB1	SVAHVKGPWTPAEDALLLSLVAQFGIQAWTAVAACMPGRSGKQCRERWLNQLDPSVRRGAWTFEEDEALVLLHARFGNCWAKMSKYLPGRTDNAIKNRWNSSMHRRW
	Cyanidioschyzon merolae	CMK280C	Cm2R-MYB2	PSVNGRGPWQPCEDERLKALVNMLGVGAWPRIAQYMVNRSGKQCRERYINNLAPGIVKTEWTRNEEMRLVQLQAIYGNRWSVIAKEFPGRTDNAVKNRFNSYLRRLE
	Cyanidioschyzon merolae	CMQ253C	Cm2R-MYB3	ERKEVKGPWRPEEDELLRSLVEKMGPRRWSLIAEHIPGRTGKQARERWLNQLSPQICKRPWTPEEDRIIISAHARLGNRWSEIARMLQGRTDNAVKNRFNSFIRRAQ
	Cyanidioschyzon merolae	CMR098C	Cm2R-MYB4	FHLRSSSDWSLPEELVLRAAVEVCGTNQWRRIASLLPGKTALQCQEHFYSSADPSLKGGGNWTVQEDRLLLQVGRSLVGQWQTVALLLPGRTADACAQRFAELTRVLDASS
	Cyanidioschyzon merolae	CMT134C	Cm3R-MYB1	WPASDDALLREKIQQCPRMDWNQVALAFQGKYTASECSQRWRQLAGRRHLKGAWSPEEDARIIDLVQKLGEKSWSRLATFLEGRTGKQCRERWFHHLAPGVNKAPWTEEEERILREKHAEFGNRWAMIARFLPGRSDNTIKNHWNGTMRRER
Chlorophyta	*Chlamydomonas reinhardtii v5.5*	g16135.t1	Cr2R-MYB01	AQGEKRGPWTPEEDKQLTDLVLSCGAQRWSTIAESIPGRSGKSCRLRWWNHLSPQVKKGPFSDFEDAVIVRSHEKYGNKWSVIAKLLPGRTDNAVKNRWNSTLKRKH
	Chlamydomonas reinhardtii v5.5	Cre02.g108350.t1.2	Cr2R-MYB02	QSRTPYKAWSKEEDDLLRALVDRHGANKWTEIAASLPGRTGKSCRLRWVNQLRGDLVMGAFSPAEDQRIVELQATLGNRWSAIAKELPGRTDNMIKNRWNSYLKRRL
	Chlamydomonas reinhardtii v5.5	g9909.t1	Cr2R-MYB03	GDDIKKGAWTPEEDALLTKLIATYGTKNWSVVAAGIKGRSGKSCRLRWHNQLNPDVKKEPFSEWEDAVIILAHEVHGNKWAAIAKLLSGRTDNSVKNHWNATLKRKV
	Chlamydomonas reinhardtii v5.5	Cre01.g034350.t1.3	Cr2R-MYB04	HPTAKLKGGWSPEEDALLTRLVKKFGEGNWSPIARALNEATGKTEATGRIGKQCRERWNHHLSPGLRKDPWTPEEEVLVVDAHKRLGNRWSDIARCIPGRSENAVKNHWNATLRKRV
	Chlamydomonas reinhardtii v5.5	Cre03.g197100.t1.2	Cr2R-MYB05	KGGWSAVEDTTLKRLVEEFGEGNWSVIARHLNASLGKPSDSGRIGKQCRERYNHHLRPDIKKDAWTEEEESLLVAAHLRYGNRWSDIAKVIRGRTENAVKNHWNATLRRKD
	Chlamydomonas reinhardtii v5.5	Cre14.g621050.t1.3	Cr2R-MYB06	YDDQGQGPWTAEEDAVLKSLIAQRGPRQWTRIAENIPGRSGKSCRLRWLNQLSPALKTSPWEPQEDAVILWAHLHYRNRWCEIAKHLPGRTDNHIKNRWNCTLKKRL
	Chlamydomonas reinhardtii v5.5	Cre07.g345350.t1.3	Cr2R-MYB07	MTQGPRNWTSIASCIPGRSGKSCRLRWLNQLSPAVKSGPFSAEEDAVILWAHMQYGNKWASIAKHLPGRTDNHIKNRWNCTLKKKH
	Chlamydomonas reinhardtii v5.5	Cre03.g149200.t1.3	Cr2R-MYB08	KAPKQSRKWSAAEIDAVLKAVAALGSSDEINWVEVAKSVPTRSGNQCREKWRNDLRPDISKEPWSSREEYIVCRVHCQVGNQWADIARFLPGRAENSIKNHYNATLRSKA
	Chlamydomonas reinhardtii v5.5	Cre02.g103450.t1.3	Cr2R-MYB09	MPLTATWTDQEDETLRKLVKEYGPKKWSVIAQKLKTKGSKQCRRRWKNYLNADLKSGGWTAEEDRILMEGHRLYGNKWTEIAKMVGGRTDNAVKNRYAALCKRDM
	Chlamydomonas reinhardtii v5.5	g2901.t1	Cr2R-MYB10	KRKEASRPWTQEETDWLITVVSRLGTDNYDAINWQEVARQVPGRTGKQCREKWKNDLRPNISKDPWSLKEEYVLALAHSLHGNRWSEVARYLPTRPENTIKNRWYATNRAKA
	Chlamydomonas reinhardtii v5.5	Cre03.g197350.t1.2	Cr2R-MYB11	RIMIKGGVWKNTEDEILKAAVMKYGLNQWARISSLLVRKSAKQCKARWYEWLDPAIKKTEWTREEDEKLLHLAKLMPCQWRTIAPIVGRTPAQCLDRYERLLDQAV
	Volvox carteri v2.0	Vocar20002419m	Vc2R-MYB1	GEDNSKGPWTPEEDEVLRGLVVTHGARNWTKIAECIPGRSGKSCRLRWLNQLSPHVRGGPFTPEEDAVILWAHLQYGNKWASISKHLPGRTDNHIKNRWNCTLKKRY
	Volvox carteri v2.0	Vocar20005463m	Vc2R-MYB2	DDDVTKGPWTPEEDTILRQLVMSQGPRNWTSIAACIPGRSGKSCRLRWLNQLSPSVKSGPFSAEEDAVILWAHMQYGNKWASIAKHLPGRTDNHIKNRWNCTLKKKH
	Volvox carteri v2.0	Vocar20009071m	Vc2R-MYB3	PIAKQKGGWTAEEDAHLARLVLEYGEGNWSPIARALNVLMKNPESTGRIGKQCRERWNHHLSPGLRKGPWGPEEEILLADAHRRLGNKWSDIARCIPGRSENAVKNHWNATLRKRL
	Volvox carteri v2.0	Vocar20005479m	Vc2R-MYB4	AKVKGGWTNVEDQILKRLVDEFGEGNWSVIARHLNSALGKPAESGRIGKQCRERYNHHLRPDIKKDAWTEEEEALLVSAHLKFGNRWSDIAKVIKGRTENAVKNHWNATLRRKD
	Volvox carteri v2.0	Vocar20001155m	Vc2R-MYB5	MAQWSAHEDEQLRKLVKEYGPKRWSVIASKLKTKGSKQCRRRWKNHLNADLKSGGWTADEDRILLEGHRLYGNKWTEIAKMVGGRTDNAVKNRYAALCKRDA
	Volvox carteri v2.0	Vocar20010663m	Vc2R-MYB6	MALKTAWTESEDALLRQLVEEKGPKNWGLIAAQIKTKSGKQCRRRWTNFLNADLKTGGWTPQEDNILMEGHKMHGNKWTEIAKMVGGRTDNAVKNR
	Volvox carteri v2.0	Vocar20005710m	Vc2R-MYB7	MIKGGVWKNTEDEILKAAVMKYGLNQWARISSLLVRKSAKQCKARWYEWLDPSIKKTEWTREEDEKLLHLAKLMPCQWRTIAPIVGRTPAQCLDRYERLLDQAV
	Micromonas pusilla RCC299	113455	Mp2R-MYB1	STKSRSRSWLPSEDELVRALVAQHGPRKWTLIASRLKTKTQKQVYARWRDYLQPGLTTKPWSKEEQAKLVELQGHVGNQWAVLARLMPGRSPNAIKNRFHATKRKME
	Micromonas pusilla RCC299	92928	Mp2R-MYB2	MRILIKGGVWKNTEDEILKAAVMKYGKNQWARISSLLVRKSAKQCKARWYEWLDPSIKKTEWTRQEDEKLLHLAKLMPTQWRTVAPIVGRTPAQCLERYEKLLDAAC
	Ostreococcus lucimarinus	31513	Ol2R-MYB1	IQIKGGVWKNTEDEILKAAVMKYGKNQWPRIASLLNRKSAKQCKARWFEWLDPSIKKTEWTREEDEKLLHLAKLMPTQWRTIAPVVGRTPSQCLERYEKLLDAAC
Bryophyta	*Physcomitrella patens V6.1*	Pp1s6_416V6.1	Pp2R-MYB01	EGGIYKGPWTEAEDTTLSAYIKAHGEGNWRLLPKCAGLSRCGKSCRLRWLNYLRPGLKKGKFSWDEEDLIVTLHALLGNRWSLIAGRIPGRTDNEIKNYWNTHLSRKL
	Physcomitrella patens V6.1	Pp1s61_196V6.1	Pp2R-MYB02	GEGLNKGPWTAEEDSILTAFVRANGEGNWRILPKRAGLKRCRKSCRLRWMNYLRPDLKRGNFSPDEDELIIKLHSLLGNKWSLIAGRIPGRTDNEIKNYWNSRLKRNL
	Physcomitrella patens V6.1	Pp1s1_78V6.1	Pp2R-MYB03	ECGLKKGPWTPDEDQKLLAYIQNHGHGSWRSLPLNAGLQRCGKSCRLRWTNYLRPDIKRGRFSQEEDQMIVHLHAILGNRWSAIASHLPRRTDNEIKNYWNTHLKKRL
	Physcomitrella patens V6.1	Pp1s10_267V6.1	Pp2R-MYB04	EFDRIKGPWSPEEDAALQQLVEKYGARNWSLISKGIPGRSGKSCRLRWCNQLSPQVQHRPFTTAEDETIIAAHSQHGNKWATIARLLPGRTDNAIKNHWNSTLRRRH
	Physcomitrella patens V6.1	Pp1s103_56V6.1	Pp2R-MYB05	RPHIIKGQWTPEEDRYLVELVERHGQQRWSLIATQLTGRIGKQCRERWHNHLRPDIKRDGWNTEEEEALVSAHNKLGNRWADIAKMIPGRTENAIKNHWNATMRRKD
	Physcomitrella patens V6.1	Pp1s106_216V6.1	Pp2R-MYB06	RMSVKKGPWTPEEDQKLVAYIQEHGHGSWRELPEKAGLARCGKSCRLRWTNYLRPDIKRGRFSHEEDQKIIQLHAILGNRWSAIAGHLPQRTDNEIKNYWNTHLKKRL
	Physcomitrella patens V6.1	Pp1s106_224V6.1	Pp2R-MYB07	KMGLKKGPWTPEEDQKLVDYIQRFGHGSWRALPKHAGLSRCGKSCRLRWTNYLRPDIKRGQFSFEEEQVIIHLHGLLGNRWSAIAAYLPGRTDNEIKNHWNTHLKKRL
	Physcomitrella patens V6.1	Pp1s114_136V6.1	Pp2R-MYB08	DEVLRKGPWMPEEDEILVEYVRQFGARDWSSIRTKGLLPRTGKSCRLRWVNKLKPDLKRSGCKFSPEEEKLVVEMQAKLGNKWAKIASCLPGRTDNDVKNFWSTRQKRIL
	Physcomitrella patens V6.1	Pp1s133_87V6.1	Pp2R-MYB09	TDPVSKGPWTEAEDKILTECVEKYGDRCWRQIPKHGGLLRDGKSCRLRWKNYLKPGLKRGKFSKDEDEFIITLHALLGNRWSLIAGRIPGRTDNEIKNHWNTHLKKRL
	Physcomitrella patens V6.1	Pp1s141_40V6.1	Pp2R-MYB10	YEDVRKGAWTAEEDEKLRKHVETYGTGHWRSVGKLAGLQRCGKSCRLRWTNYLRPDIRHGSFSSEEENLIVKLHEAHGSRWSLIAAQMPGRTDNDIKNHWNTRLKKKL
	Physcomitrella patens V6.1	Pp1s149_164V6.1	Pp2R-MYB11	KQKLKKGLWSPEEDEKLVRYVTKHGHGCWSVVPKQAGLQRCGKSCRLRWINYLRPDLKRGVFSAAEENLIIDLHAVLGNRWSQIATQLPGRTDNEIKNFWNSCIKKKL
	Physcomitrella patens V6.1	Pp1s151_41V6.1	Pp2R-MYB12	SKLCPRGRWRPAKDEKLRELVSQYRPQNWSLIAEKLHGKSCRLRWFNQLDLWINRRPFTEDGEERLLAAHRFQGNKWAMIVRLFSGRTDNAVKNHWHVVMAQKF
	Physcomitrella patens V6.1	Pp1s16_281V6.1	Pp2R-MYB13	DEVLRKGPWMPEEDEILVEYVRQFGARDWSSIRTKGLLPRTGKSCRLRWVNKLKPDLKRSGCKFSPEEEKLVVEMQAKLGNKWAKIASCLPGRTDNDVKNFWSTRQKRIL
	Physcomitrella patens V6.1	Pp1s186_70V6.1	Pp2R-MYB14	PNIIKGQWSPEEDRYLVGLVEMHGQQRWSLIATQLPGRIGKQCRERWHNHLRPDIKRDGWNTEEEEALVSAHNKLGNRWADIAKMIPGRTENAIKNHWNATMRRKD
	Physcomitrella patens V6.1	Pp1s189_29V6.1	Pp2R-MYB15	KQKLKKGLWSPEEDEKLVRYITKHGHGCWSAVPKQAGLQRCGKSCRLRWINYLRPDLKRGVFSPAEENLIIDLHAVLGNRWSQIATQLPGRTDNEIKNFWNSCIKKKL
	Physcomitrella patens V6.1	Pp1s2_283V6.1	Pp2R-MYB16	SKQCPRGHWRPAEDDKLRELVSQFGPQNWNLIAEKLQGRSGKSCRLRWFNQLDPRINRHPFSEEEEERLLIAHKRYGNKWALIARLFPGRTDNAVKNHWHVVTARQS
	Physcomitrella patens V6.1	Pp1s2_55V6.1	Pp2R-MYB17	EAGLRKGPWTPDEDRKLVAYIQEHGHGSWRALPKNAGLMRCGKSCRLRWTNYLRPDIKRGRFSEAEDQMIVHLHSILGNRWSTIAAHLPGRTDNEIKNYWNTYLKKRL
	Physcomitrella patens V6.1	Pp1s2_60V6.1	Pp2R-MYB18	QEGLKKGPWTPNEDQKLVAYIQNHGHGSWRSLPLNAGLQRCGKSCRLRWTNYLRPDIKRGRFSQDEDQMIIHLHAILGNRWSAIASHLPRRTDNEIKNYWNTHLKKRL

		Physcomitrella patens V6.1	Pp1s200_41V6.1	Pp2R-MYB19	SKLCPRGHWRPAEDDKLRELVSQYGPQNWNLIAEKLQGRSGKSCRLRWFNQLDPRINRRPFTEEEEDRLLAAHRFHGNKWAMIARLFPGRTDNAVKNHWHVVMARKF
		Physcomitrella patens V6.1	Pp1s204_13V6.1	Pp2R-MYB20	NEDVRKGAWAAEEDEKLRKYVETYGTGHWRSVGKKAGLQRCGKSCRLRWTNYLRPDIRHGSFTQEEENLIVKLHAAHGSRWSLIAAQMPGRTDNDIKNHWNTRLKKKL
		Physcomitrella patens V6.1	Pp1s206_27V6.1	Pp2R-MYB21	RQPGMKGQWGAAEDRVLRKLVKQYGSRRWSLISTFMANRSGKQCRERWVNHLHPDIRKEGWTTEEEELLVHAHSTFGNRWSAIAKMLPGRTDNSIKNHWHATLRKKD
		Physcomitrella patens V6.1	Pp1s211_139V6.1	Pp2R-MYB22	KQKLKKGLWSPEEDDKLVRYITQHGHGCWSAVPKQAGLQRCGKSCRLRWINYLRPDLKRGVFSATEESLIIDWHAALGNRWSQIATQLPGRTDNEIKNFWNSCIKKKL
		Physcomitrella patens V6.1	Pp1s219_84V6.1	Pp2R-MYB23	SKLCPRGHWRPAEDEKLRELVSQYGPQNWNLIAEKLHGRSGKSCRLRWFNQLDPRINRRPFTEDEEERLLAAHSFHGNKWAMIARLFLGRTDNAVKNHWHVVMARKF
		Physcomitrella patens V6.1	Pp1s238_70V6.1	Pp2R-MYB24	GGALKKGPWTSAEDSILISYVTKHGEGNWNSVQKHSGLFRCGKSCRLRWANHLRPNLKKGAFTPEEERIIVELHAKIGNKWARMAAQNFQ
		Physcomitrella patens V6.1	Pp1s24_48V6.1	Pp2R-MYB25	KQKLKKGLWSPEEDEKLVRYISKHGHGCWSAVPKQAGLQRCGKSCRLRWINYLRPDLKRGVFSPAEENLIIDLHAVLGNRWSQIATQLPGRTDNEIKNFWNSCIKKKL
		Physcomitrella patens V6.1	Pp1s257_83V6.1	Pp2R-MYB26	NDTVKRGPWTPEEDDKLVTWIAQYGAGSWRTLPKRAGLRRCGKSCRLRWTNYLRPDLKHGRFSEYEEQLIVHLHATLGSRWSLIAAQLPGRTDNDVKNYWNSRLKRKL
		Physcomitrella patens V6.1	Pp1s277_14V6.1	Pp2R-MYB27	KQKLKKGLWSPEEDEKLVRFITKHGHGCWSAVPKQAGLQRCGKSCRLRWINYLRPDLKRGVFSAAEENLIIDLHAVLGNRWSQIATQLPGRTDNEIKNFWNSCIKKKL
		Physcomitrella patens V6.1	Pp1s284_57V6.1	Pp2R-MYB28	SEDVRKGAWAAEEDEKLRKYVETYGTGHWRSVGKKAGLRRCGKSCRLRWTNYLRPDIRHGSFTPQEEDLIVKLHAAHGSRWSLIAAQMPGRTDNDIKNHWNTRLKKKL
		Physcomitrella patens V6.1	Pp1s295_52V6.1	Pp2R-MYB29	INDVRKGAWGADEDEKLRKYVEKYGTGHWRSVGKKAGLQRCGKSCRLRWTNYLRPDIRHGSFSEEEDLIVKLHTSHGSRWSLIAAQLPGRTDNDIKNHWNTRLKKKL
		Physcomitrella patens V6.1	Pp1s313_81V6.1	Pp2R-MYB30	RQNTIKGLWTIEEDRHLIELVKRYGQQRWTLIANYLSGRIGKQCRERWHNHLRPDIKKEGWSSEEEEYLVFVHNTLGNRWADIAKLIPGRTENAIKNHWNATMRRKD
		Physcomitrella patens V6.1	Pp1s317_55V6.1	Pp2R-MYB31	KMGLKKGPWTPDEDQKLVTYIQRNGHGSWRALPKHAGLLRCGKSCRLRWTNYLRPDIKRGRFSFEEQVIIHLHGILGNRWSAIAAHLPGRTDNEIKNYWNTHLRKRL
		Physcomitrella patens V6.1	Pp1s326_37V6.1	Pp2R-MYB32	KLSLKKGPWTPDEDQKLVAYIQEHGHGSWRALPEKAGLARCGKSCRLRWTNYLRPDIKRGRFSHEEDQMIIQLHAILGNRWSAIAGHLPQRTDNEIKNYWNTHLKKRL
		Physcomitrella patens V6.1	Pp1s36_198V6.1	Pp2R-MYB33	KDSVKRGPWTPEEDAKLLACIAQHGTGSWRTLPKKAGLQRCGKSCRLRWTNYLRPDLKHGRFTDHEEQLIVNLHAALGSRASFLVQPASASGHIGSRTRLSIRVKTLLDASYFPRI CIFREVYEAEYFSRWSLIAAQLPGRTDNDVKNYWNTRL
		Physcomitrella patens V6.1	Pp1s371_6V6.1	Pp2R-MYB34	EEGFYKGPWTEEEDAILIAFIKADGEGNWRTLPKRSGLKRCRKSCRLRWMNYLRPDLERGNFSPEEDELIIKLHSLLENRWSLIAGRIPGRKDNEIKNYWNSRLRRRL
		Physcomitrella patens V6.1	Pp1s389_40V6.1	Pp2R-MYB35	KEVLKKGPWTPDEDQKLVAYIQQHGHGSWRSLPINAGLQRCGKSCRLRWTNYLRPDIKRGRFSHEEDQMIVHLHAILGNRWSAIASHLPRRTDNEIKNYWNTHLKKRL
		Physcomitrella patens V6.1	Pp1s389_41V6.1	Pp2R-MYB36	EGTLRKGPWTTDEDRKLVAYIQEHGHGSWRVLPKNAGLTRCGKSCRLRWTNYLRPDIKRGRFSEAEDQTIVHLHSVLGNRWSTIAAHLPGRTDNEIKNYWNTHVKKRL
		Physcomitrella patens V6.1	Pp1s39_301V6.1	Pp2R-MYB37	SKLCSRGHWRPVEDQKLRELVSLHGPQNWNLIAEKLQGRSGKSCRLRWFNQLDPRINRRPFTEEEEERLLAANQLQGNKWALIARLFPGRTDNAVKNHWHVLTARKM
		Physcomitrella patens V6.1	Pp1s420_4V6.1	Pp2R-MYB38	KVGLRRGPWTSEEDQKLVSHITNNGLSCWRAIPKLAGLLRCGKSCRLRWTNYLRPDLKRGIFSEAEENLILDLHATLGNRWSRIAAQLPGRTDNEIKNYWNTRLKKRL
		Physcomitrella patens V6.1	Pp1s433_19V6.1	Pp2R-MYB39	KVGLKRGPWAADEDKKLVSYINAHGVSCWRAIPKLAGLLRCGKSCRLRWTNYLRPDLKRGGFTDAEENLILDLHATLGNRWSKIAMQLPGRTDNEIKNYWNTHLKKKL
		Physcomitrella patens V6.1	Pp1s45_150V6.1	Pp2R-MYB40	DASLKKGPWTPDEDQKLVAYIQKHGHGSWRSLPLHAGLQRCGKSCRLRWTNYLRPDIKRGRFSQEEDQMIVHLHAILGNRWSAIASHLPRRTDNEIKNYWNTHLRKRL
		Physcomitrella patens V6.1	Pp1s46_302V6.1	Pp2R-MYB41	NEDVRKGAWAAEEDEKLRKYVETHGTGHWRSVGKKAGLQRCGKSCRLRWTNYLRPDIRHGSFDPEEEDLIVKLHAAHGSRWSLIAAQMPGRTDNDIKNHWNTRLKKKL
		Physcomitrella patens V6.1	Pp1s53_216V6.1	Pp2R-MYB42	TDDRIKGPWSPEEDVVLNRLVEKFGARNWSLIARGIPGRSGKSCRLRWCNQLNPGVKRKPFTEEEDRAIVAAHAIHGNKWASIARMLPGRTDNAIKNHWNSTLRRKH
		Physcomitrella patens V6.1	Pp1s641_1V6.1	Pp2R-MYB43	RIMIKGGVWKNTEDEILKAAVMKYGKNQWARISSLLVRKSAKQCKARWYEWLDPSIKKTEWTREEDEKLLHLAKLMPTQWRTISPIVGRTPAQCLERYEKLLDAAC
		Physcomitrella patens V6.1	Pp1s65_211V6.1	Pp2R-MYB44	KDSVKRGPWTPEEDAKLLACIAQHGTGSWRTLPKKAGLQRCGKSCRLRWTNYLRPDLKHGRFTDHEEQLIVNLHAALGSRWSLIAAQLPGRTDNDVKNYWNTRLKKKL
		Physcomitrella patens V6.1	Pp1s65_233V6.1	Pp2R-MYB45	DPPTAFSEQEDDILRELVKRHGTKKWHIISSKMQKKTPRECRKRWRTYLHMCMNKSSWSAEEDHLLLQGHNAFGNRWTEIAKMVPGRTDNAVKNRYNALCTKRS
		Physcomitrella patens V6.1	Pp1s66_200V6.1	Pp2R-MYB46	GGSLKKGPWTSAEDSILISYVTKHGEGNWNSVQKHSGLFRCGKSCRLRWANHLRPNLKKGAFTPEEERTIVELHAKLGNKWARMAAQLPGRTDNEIKNYWNTRIKRRM
		Physcomitrella patens V6.1	Pp1s72_143V6.1	Pp2R-MYB47	TDDRIKGPWSPEEDVVLNRLVEKFGARNWSLIARGIPGRSGKSCRLRWCNQLNPGVKRKPFTEEEDRAIVAAHAIHGNKWASIARMLPGRTDNAIKNHWNSTLRRKH
		Physcomitrella patens V6.1	Pp1s74_264V6.1	Pp2R-MYB48	SKKCPRGHWRPEEDENLRKLVSRFGPQNWNLIAQKLHGRSGKSCRLRWFNQLDPRINRRPFTDDEEERLLAAHRFHGNKWAMIARLFPGRTDNAVKNHWHVVMARKF
		Physcomitrella patens V6.1	Pp1s76_66V6.1	Pp2R-MYB49	SKLCPRGHWRPAEDDKLRELVSQYGPQNWNLIAEKLHGRSGKSCRLRWFNQLDPRINRRPFTEEEEDRLLSAHRFHGNKWAMIARLFPGRTDNAVKNHWHVVMARKF
		Physcomitrella patens V6.1	Pp1s78_152V6.1	Pp2R-MYB50	KAGLKRGPWTVEEDQKLVSYITNNGLGCWRATPKLAGLLRCGKSCRLRWINYLRPDLKRGIFSEEEENLILDAHATLGNRWSRIAAQLPGRTDNEIKNYWNTRLKKRL
		Physcomitrella patens V6.1	Pp1s91_13V6.1	Pp2R-MYB51	SKLCPRGHWRPAEDEKLRELVSQYGPQNWNLIAEKLHGRSGKSCRLRWFNQLDPRINRRPFTEDEEERLLAAHRFHGNKWAMIARLFPGRTDNAVKNHWHVVMARKF
		Physcomitrella patens V6.1	Pp1s36_43V6.1	Pp3R-MYB1	TRRSSKGGWTPEEDETLRRAVQCFNGKNWKKIAEFFTDRTDVQCLHRWQKVLNPDLVKGAWTKEEDDRIMELVNKYGAKKWSVIAQNLPGRIGKQCRERWHNHLNPSIKREA WTQQEDLALIRAHQLYGNKWAEIAKFLPGRTDNSIKNHWNSTMKKKV
		Physcomitrella patens V6.1	Pp1s120_159V6.1	Pp3R-MYB2	TRRSSKGGWTPEEDETLRRAVQCFNGKNWKKIAEFFTDRTDVQCLHRWQKVLNPDLVKGAWTKEEDDRIMELVNKYGAKKWSVIAQNLPGRIGKQCRERWHNHLNPSIKREA WTQQEDLALIRAHQLYGNKWAEIAKYLPGRTDNSIKNHWNSTMKKKV
	Lycopodiophyta	*Selaginella moellendorffii*	98669	Sm2R-MYB01	KVGLNRGPWTPEEDMLLTKHIEQHGEGNWRALPKAAGLLRCGKSCRLRWVNYLRPNVKRGNISEDEEDLIIMLHALLGNRWSLIAARMPGRTDNEIKNYWNTHLSKKL
		Selaginella moellendorffii	109587	Sm2R-MYB02	KVGLNRGPWTREEDALLIKYITAHGEGSWRTLPKQAGLRRCGKSCRLRWINYLRPDLKRGNITEEEDELIIKLHSLLGNRWSLIAGRLAGRTDNEIKNYWNTHLKKKL
		Selaginella moellendorffii	437219	Sm2R-MYB03	KPKLRKGLWSPEEDEKLVRYITKYGHKCWSIVPKHAGLQRCGKSCRLRWINYLRPDLKRGHFSQEEELIVKLHAVLGNRWAQIATQLPGRTDNEIKNFWNSCLRKKL
		Selaginella moellendorffii	81112	Sm2R-MYB04	AAALKKGPWTSAEDAILVAYVSKHGEGNWNNVQKHSGLSRCGKSCRLRWANHLRPNLKKGAFTPDEERLIIELHAKLGNKWARMAAQLPGRTDNEIKNYWNTRIKKRL
		Selaginella moellendorffii	84369	Sm2R-MYB05	AAALKKGPWTSAEDSILVAYVQKHGEGNWNNVQKQSGLYRCGKSCRLRWANHLRPNLKKGAFTPEEERLIIELHAKLGNKWARMATQLPGRTDNEIKNYWNTRIKRRQ
		Selaginella moellendorffii	93784	Sm2R-MYB06	VKGLKKGPWTPSEDAVLVAYVHKHGEGNWNNVQKNCGLSRCGKSCRLRWANHLRPNLKKGAFTPEEERTIIELHAKLGNKWARMASQ
		Selaginella moellendorffii	100734	Sm2R-MYB07	QRNRVKGQWSLAEDKSLVKLVKQYGTRRWSLISTFMANRSGKQCRERWVNHLQPDIRKEGWTKEEEELLVQAHSYFGNKWSAIAKMLPGRTDNSIKNHWHAALRKKV
		Selaginella moellendorffii	99462	Sm2R-MYB08	ELDRIKGPWSPEEDAALQKLVDKYGARNWSLISKGIAGRSGKSCRLRWCNQLSPQVEHRPFTAAEDATIIKAHSQHGNKWATIARLLPGRTDNAIKNHWNSTLRRR
		Selaginella moellendorffii	80553	Sm2R-MYB09	SKLCHRGHWRPAEDEKLKELVRIHGPQNWNVIAEKLEGRSGKSCRLRWFNQLDPRINRKPFSEEEEERLLAAHQLHGNKWAMIARIFPGRTDNAVKNHWHVVMARKL

	Selaginella moellendorffii	77255	Sm2R-MYB10	SIDKVRGPWSPEEDANLQKLIQKYGARNWSLISKGVPGRSGKSCRLRWCNQLSPQVEHKPFSCAEDAIIIEAHAQHGNKWATIARLLPGRTDNAIKNHWNSTLRRR
	Selaginella moellendorffii	140962	Sm2R-MYB11	IATKDRQRWQPEEDAILCAYVTQYGADDWNLISERMGEPLDRDPKSCHERWKNYLKPGIKKGPLTDEEQQLVIKLQTKYGNKWKRIAAEVPGRTAKRLGKWWEVYKERLI
	Selaginella moellendorffii	179572	Sm2R-MYB12	RIMIKGGVWKNTEDEILKAAVMKYGKNQWARISSLLVRKSAKQCKARWYEWLDPSIKKTEWTREEDEKLLHLAKLMPTQWRTIAPIVGRTPAQCLERYEKLLDAAC
	Selaginella moellendorffii	233652	Sm2R-MYB13	RIMIKGGVWKNTEDEILKAAVMKYGKNQWARISSLLVRKSAKQCKARWYEWLDPSIKKTEWTREEDEKLLHLAKLMPTQWRTIAPIVGRTPAQCLERYEKLLDAAC
	Selaginella moellendorffii	113921	Sm2R-MYB14	RIMIKGGVWKNTEDEILKVAVMKYAKNQWPRISSLLARKSAKQCKARWYEWLDPSIKKTEWTREEDEKLLHLAKLMPTQWRTIAPIVGRTPAQCLQRYEKLLDAAC
	Selaginella moellendorffii	420607	Sm2R-MYB15	MRIMIKGWKNTEDEILKVAAMKYAKNQWPRISSLLPRKSAKQCKARWYEWLDLSIKKTEWTCEEDEKLLHLAKLMLTQWRTIAPIVGRPPAQCLERATSRLMIQI
Gymnosperm	*Picea abies V1.0*	MA_10048467g0010	Pa2R-MYB001	DGALHRGSWTPEEDRILKTYIEANGVGRWSTLPQNAGLMRRGKSCRLRWMNHLRPNVKRGHISPDEEELIIRLHNLLGNRWSLIAGRVPGRTDNEVKNYWNTRLSKKL
	Picea abies V1.0	MA_10157944g0010	Pa2R-MYB002	AHTNSKGAWTKEEDKRLIAYIEAHGEGSWRSLPKAAGLLRCGKSCRLRWINYLRPDVKRGNFSEEEDDLIIKLHSLLGNKWSLIAGRLPGRTDNDIKNYWNTHIKKKL
	Picea abies V1.0	MA_101790g0010	Pa2R-MYB003	KARTNKGTWTKEEDHRLVTHIRLHGEGGWRSLPKAAGLLRCGKSCRLRWINYLRPDLKRGSFTEEEDELIIKLHSVVGNKWSLIAGRLPGRTDNEIKNYWNTHIKRKL
	Picea abies V1.0	MA_10190951g0010	Pa2R-MYB004	QPQTRKGPWTVQEDMQLIRYISLQGEGRWPYLAKAAGLKRTGKSCRLRWINYLRPDLKRSKITPEEERLIIKLHGRWGNRWSRIAQNLPGRTDNEIKNYWRTRIKKRL
	Picea abies V1.0	MA_101987g0010	Pa2R-MYB005	MKERQRWRSEEDSLLHAYVKRYGPREWNLVSQRMNKALDRDPKSCAERWKNYLKPGIKKGSLSEDEQRLVISLQAKYGNRWKKIAAQVPGTTAKRLSKWWEVYREKQD
	Picea abies V1.0	MA_10208000g0010	Pa2R-MYB006	GGSLKKGPWTSAEDAILVEYVKKHGEGNWNAVQKHSGLFRCGKSCRLRWANHLRPNLKKGAFTAEEEQIIIELHAKLGNKWARMAAQLPGRTDNEIKNYWNTRIKRRQ
	Picea abies V1.0	MA_10225049g0010	Pa2R-MYB007	HKGLKRGPWTAEEDQKLIHYLQHHGLATWRTVPKLAGLSRCGKSCRLRWTNYLRPDIKRGTFSIHEDLIIIRLHAILGNKWSAIASRLPGRTDNEIKNHWNSRLKKQL
	Picea abies V1.0	MA_10249055g0010	Pa2R-MYB008	DEGFNRAAWTTKEDMILRDYINIHGLEGGWRSLRDKTGLNRSGKSCRLRWMNYLRPNIKRGNISTDEEELIIRLHRLLGNRWSLIAGRLPGRTDNEIKNYWNTHLRKKI
	Picea abies V1.0	MA_10298381g0010	Pa2R-MYB009	YVGLSKGPWTAEEDLRLIKYIETHGEGNWRTLPRKAGLQRCGKGCRLRWMNYLRPNVKRGHISPDEEEMIVRLHKLLGNRWSLIAGRIPGRTDNEVKNYWNTHLRKKV
	Picea abies V1.0	MA_10320g0010	Pa2R-MYB010	KTKTRKGPWTMEEDMKLVRYISLHGEGRWNFLAKAAGLKRTGKSCRLRWNNCLRPDLKRGKITSEEERLIIELHGRWGNRWSRIAQSLPGRTDNEIKNCWRTRIKKKF
	Picea abies V1.0	MA_10425867g0020	Pa2R-MYB011	MCPRGHWRPAEDEKLRELVEQYGPQNWNSIAEKLQGRSGKSCRLRWFNQLDPRINRRPFTEEEEERLLAAHRLHGNKWAMIARLFPGRTDNAVKNHWHVIMARKF
	Picea abies V1.0	MA_10426233g0010	Pa2R-MYB012	NPELVKGPWTKEEDELIVELVRKYGSKKWSVISQSLPGRIGKQCRERWHNHLNPEIKKDAWSQDEEMALIRAHQIYGNKWAEIAKSLPGRTDNSIKNHWNSSIKKKL
	Picea abies V1.0	MA_10429939g0010	Pa2R-MYB013	DEGLNRVAWTTKEDMILRDYINIHGLEGGWRSLRDKTGLNRSGKSCRLRWMNYLRPNIKRGNISTDEEELIIRLHRLLGNRWSLIAGRLPGQTNNEIKNYWNTHLRKKI
	Picea abies V1.0	MA_10430220g0010	Pa2R-MYB014	KMGVKKGPWTLDEDQILVSYINKHGHGNWRALPKQAGLLRCGKSCRLRWTNYLKPDIKRGNFSPEEEDHIIKLNQLIGNRWSTIASHLPGRTDNEIKNVWNTHLKKRL
	Picea abies V1.0	MA_10431212g0010	Pa2R-MYB015	KKAVKRGFWSPEEDMKLISYIATNGHGCWSSLSERAGLQRCGKSCRLRWMNYLRPGIKRGNFSTEEENAIISLQARLNNRWAQIAKHLPGRTDTEIKNYWHSCLKKKA
	Picea abies V1.0	MA_10431610g0040	Pa2R-MYB016	ETQTRKGPWTVEEDMQLIKYINLHGEGRWSFVVKAAGLERTGKSCRLRWVNYLRPDLKRSKITHEEERLIIELHGRLGNRWSRIAQSLPGRTDNDIKNYWRTCITKKL
	Picea abies V1.0	MA_10432903g0010	Pa2R-MYB017	VHTNNKGAWTKEEDERLIAHIEAHGEGSWRSLPKAAGLLRCGKSCRLRWINYLRPDLKRGSISEEEDDLIIKLHSLLGNRWSLIAGRLPGRTDNEIKNYWNTHMKRKL
	Picea abies V1.0	MA_10433518g0010	Pa2R-MYB018	HKGQKRGTWTAEEDQILIHYLQRRGLSNWRALPRLAGLSRCGKSCRLRWTNYLRPGIKRGAFTIQEDLTTIRLHAVFGNKWSAIASHLPGRTDNEIKNRWNSKLKKQL
	Picea abies V1.0	MA_10433651g0010	Pa2R-MYB019	GLKLRKGAWTSEEDLLLKKCIEVHGEGSWPLLPQKTGLQRSGKSCRLRWLNYLSPNVKRGNISEDEELLIIRMHRLLGNRWALIAGRIPGRSDNEIKNYWNTHLSKKV
	Picea abies V1.0	MA_10434028g0010	Pa2R-MYB020	VEIMRKGPWTLEEDMRLIHYITLRGEGRWNFLAKAAGLKRSGKSCRLRWVNYLRPDLKRGDITPEEERLIIDLQGRWGNRWSRIAKKLPGRTDNEIKNYWRTRIKKKI
	Picea abies V1.0	MA_10435380g0010	Pa2R-MYB021	KPKLRKGLWSPEEDDKLIKYMMKNGRGCWSDVAMQAGLQRCGKSCRLSWINYLRPDLKRGAFSPQEEQLIIHLHSILGHRWSQIAARLPGRTDNEIKNFWNSCIKKKL
	Picea abies V1.0	MA_10435612g0010	Pa2R-MYB022	KVGLKKGPWTPEEDQKLVTYIQEHGHGSWRALPQKAGLLRCGKSCRLRWANYLRPDIKRGKFSVQEEQTIIQLHALLGNRWSAIATHLPKRTDNEIKNYWNTHLKKRL
	Picea abies V1.0	MA_10435865g0010	Pa2R-MYB023	GEGIKKGLWSAEEDEKLINFMVKKNRPMGCSWTCVAQQIGLQRCGKSCRLRWINYLRPGLKHCAISAEEEQSIIHLHSVLGNRWSQIAAHLPGRTDNEIKNYWNTRIKRKL
	Picea abies V1.0	MA_10436747g0010	Pa2R-MYB024	NPNLRKGLWSPEEDEKLFSYLINNGTRSPGCSWTCVAKQAGLQRCGKSCRLRWINYLRPGLKRGAFSLEEEGLIIHLQSILGNRWSQISVHLPGRTDNEIKNYWNSCVKKKR
	Picea abies V1.0	MA_10859g0010	Pa2R-MYB025	KANVKKGPWSPEEDCKLKEYIDKHGTGGNWLALPQKVGLKRCGKSCRLRWLNYLRPNIKHGGFSEEEDNIICSLFISIGSRWSIIAAQLPGRTDNDIKNYWNTRLKKKL
	Picea abies V1.0	MA_113824g0010	Pa2R-MYB026	EAETRKGPWTVEEDMQLIGSISLHGEGRWSFLAGAAGLKRTGKSCRLRWVNYLRPDLKRSKITREEERLIIELHRRWGNMWSRIAQSLPGRTDNEIKNFWRTRMKGKL
	Picea abies V1.0	MA_115846g0010	Pa2R-MYB027	MIKGGVWKNTEDEILKAAVMKYGKNQWARISSLLVRKSAKQCKARWYEWLDPSIKKTEWTREEDEKLLHLAKLMPTQWRTIAPIVGRTPSQCLERYEKLLDAAC
	Picea abies V1.0	MA_116862g0010	Pa2R-MYB028	EESLNRGAWTGSEDRILSEYIKTHGVGGWKDLPKKAGLKRCGKSCRLRWLNYLRPDIKRGNISPEEEELLVRLHRLLGNRWSLIAGRLPSRTDNEIKNYWNTHLSKRV
	Picea abies V1.0	MA_117992g0010	Pa2R-MYB029	ETETRKGPWTVEEDMLLSKYIRLHGEGRWNFLAKAAGLKRTGKSCRLRWINYLRPDLKRSKITLEEECLIIELQGRWGNRWSRIAQRLPGRTDNEIKNYWRSSIKKKL
	Picea abies V1.0	MA_118448g0010	Pa2R-MYB030	KKGLKKGPWSHEEDQILLNYIQRNGHASWRALPELAGLLRCGKSCRLRWTNYLRPDIKRGSFTEQEERTIIQLHAILGNRWSSIASQLPGRTDNEIKNFWKTRLQKRL
	Picea abies V1.0	MA_120049g0010	Pa2R-MYB031	EPDRIKGPWSPEEDAALQHFVQKCGPRNWSLISKAIPGRSGKSCRLRWCNQLSPQVEHRPFTPEEDATIIRAHAQHGNKWATIARMLSGRTDNAIKNHWNSTLRRRC
	Picea abies V1.0	MA_1201g0010	Pa2R-MYB032	KMGVKKGSWTSDEDQILVSYINKHGHGNWRALPKQAGLLRCGKSCRLRWTNYLRPDIKRGNFSPEEEDHIIKLHQLIGNKWSTIALHLPGRTDNEIKNVWGTRLKKRL
	Picea abies V1.0	MA_121533g0010	Pa2R-MYB033	KANVKRGPWSPEEDTILKNFVEKHGTGGNWIALPRKAGLKRCGKSCRLRWLNYLRPDIKHGDFSEEEDSIICSLYTSIGSRWSIIAAQLPGRTDNDIKNYWNTRLKKKL
	Picea abies V1.0	MA_12445g0010	Pa2R-MYB034	KEGLNRGAWSAEEDMILWEYIRKHGDGGWSKVPQKTGLKRCGKSCRLRWVNYVRPDIRRGNICPDEEELICRLHRLLGNRWSLIAGRLPGRTGNEIKNYWNTHLKKKL
	Picea abies V1.0	MA_124887g0010	Pa2R-MYB035	SGDGNKGAWTKEEDARLVQHIQAHGEGCWRTLPKAAGLLRCGKSCRLRWINYLRPDLKRGYFSEDEDDLILKLHALLGNKWSLIAGRLPGRTDDEIKNYWNSHLKRNM
	Picea abies V1.0	MA_1261g0010	Pa2R-MYB036	NEGLKRGPWTAEEDMILLEYIRIHGDGGWRKLPEKAGESQQIWYMNADVDSIDRLNKLRKYFFAGLKRCGYSCRLRWVNYLRPDIKRGNIAPDEEELICRLHRLLGNRWSVIAGL LPGRTDNEIKNYWSTRLKKKL
	Picea abies V1.0	MA_128244g0010	Pa2R-MYB037	KTGLKKGPWTSEEDKILVNYIQENGHGSWRALPKLAGLLRCGKSCRLRWTNYLKPDIKRGSFTQEEERTIIQLHAILGNRWSVIASQLLGRTDNEIKNFWNTHLKKRL
	Picea abies V1.0	MA_128871g0010	Pa2R-MYB038	KVGLKKGPWTAEEDKKLINFLATHGQCCWRTVPELAGLSRCGKSCRLRWTNYLRPDLKRGVFSESEEKLIIDIHSHVGNRWSKIATFLPGRTDNEIKNYWNTHIKKKL

Picea abies V1.0	MA_129382g0010	Pa2R-MYB039	KMGLNRGPWTPEEDDLLVKYIQKHGEGGWRTLPKKAGLLRCGKSCRLRWMNYLRPDVKRGQILPDEEDLILRLHRLLGNRWSLIAGRMPGRTDNEIKNYWNTHLSKKL
Picea abies V1.0	MA_130648g0010	Pa2R-MYB040	EIQLRKGPWMPEEDEILVEYVRQYGPRDWSSIRSKGLLPRTGKSCRLRWVNKLKPDLKSGCKFSPEEEKLVVEMQAKLGNKWAKIASYLPGRTDNDVKNFWSTRQKRIM
Picea abies V1.0	MA_130918g0010	Pa2R-MYB041	KVGLNKGAWSAEEDSLLGKYIQTHGEGNWRSLPKKAGLRRCGKSCRLRWLNYLRPCIKRGNITADEEELIIRMHALLGNRWSIIAGRVPGRTDNEIKNYWNTNLSKKL
Picea abies V1.0	MA_130951g0010	Pa2R-MYB042	QPKIRKGPWSPEEDEKLINYMMNNCLRVCSWIYVAKQVGLQRCRRSCRLRWINYLRPGLKRGAISLEEEQLIIHLHSTLGNRWSQIAAHLPGRTDNAIKNYWNTRIKKKL
Picea abies V1.0	MA_134436g0010	Pa2R-MYB043	QVGFHRGPWTAKEDSLLTRWIECHGEGDWRNLPEKAGLLRCGKSCRLRWLNYLRPTIKRGNISPDEEDLIIRLHRLLGNRWSLIAGRLPGRTDNEIKNYWNSHLSKKL
Picea abies V1.0	MA_136123g0010	Pa2R-MYB044	KEGLNRGAWTAKEDMILSEYIRIHGDGGWTNLPQKAGLKRSGKSCRLRWLNYLRPDIKRGNISPDEDRSGLKRSGKSCRLRWLNYLRPDIKRGNISPDEEELIIRLHRLLGNRWSLIAGRLPERTDNEIKNYWNTHLSKKL
Picea abies V1.0	MA_137453g0010	Pa2R-MYB045	KTGLKKVPWTREEDKILVNYIQKNGHGNWRALPKLAGLLRSGKSCRLRWTNYLSPDIKRGSFTEEEEQSIIQLHAILGNKWSAIASQLPGRTDNEIKNFWSSRLKKRL
Picea abies V1.0	MA_137886g0010	Pa2R-MYB046	KPKLRKGFWSPEEDSKLSEYILKNGLMGCWSYVAKQAGLERCGKSCRLRWINYLRPGLKRGPISQQEERLILHLQSILGNRWARIATQLPGRTDNEIKNYWNSCIKKRL
Picea abies V1.0	MA_137934g0010	Pa2R-MYB047	KMGVKKGSWTLDEDQILVSYINKHGHGNWRALPKQAGLLRCGKSCRLRWTNYLRPDLKRGNFSPEEEDHIIKLHQLIGNRWSTIASYLPGRTDNEIKNVWSTRLKKRL
Picea abies V1.0	MA_13869g0010	Pa2R-MYB048	KPKLRKGLWSPEEDEKLISYIMKKGMLGCSWTYVAKRAGLQRCGKSCRLRWINYLRPELKRGTFSSQEEQLIIHLHSVLGNSWCQIAASLPGRTDNDIKNYWNSFIKKKK
Picea abies V1.0	MA_139238g0010	Pa2R-MYB049	KQKLRKGLWSPEEDEKLVRHITKYGHGCWSAVPKQAGLQRCGKSCRLRWINYLRPDLKRGTFSPQEENLIIELHAVLGNRWSQIATHLPGRTDNEIKNLWNSCIKKKL
Picea abies V1.0	MA_139448g0010	Pa2R-MYB050	KGKLKKGPWTFEEDLRLLRHIRLHGEGRWSALAKAAGLQRCGKSCRLRWVNYLRPDLKRGNITPEEEQVIIDLHARWGNRWSLIAERMPGRTDNEIKNYWRCHLKKKL
Picea abies V1.0	MA_139924g0010	Pa2R-MYB051	KAHTNKGAWTKQEDDRLIAHIRAHGEGGWRSLPKAAGLLRCGKSCRLRWINYLRPDLKRGSFTEEEDELIIKLHSFVGNKWSLIAGRLPGRTDNEIKNYWNTHIKRKL
Picea abies V1.0	MA_140579g0010	Pa2R-MYB052	KVGVNKGAWSAEEDNLLVKYIQTHGEGNWRSLPKKAGLRRCGKSCRLRWLNYLRPCIKRGNITADEEELIIRMHALLGNRWSIIAGRVPGRTDNEIKNHWNTKLSRKL
Picea abies V1.0	MA_14061g0010	Pa2R-MYB053	KANVKRGPWSPEEDCKLKEYIAKYGTGGNWIALPQKVGLKRCGKSCRLRWLNYLRPNIKHGGFSEEEDNIISSLYISIGSRWSIIAAQLPGRTDNDIKNYWNTRLKKKV
Picea abies V1.0	MA_14204g0010	Pa2R-MYB054	KEGLNRGAWTAKEDMILSDYIRIHGEGRWRNLAHKAGLMRCGKSCRQRWLNYLRPDIKLGNISPDEEELIVRMHRLLGNRWSLIAGRLPGRTDNEIKNYWNTHLKKKV
Picea abies V1.0	MA_14452g0010	Pa2R-MYB055	REELNRGAWTAEEDSRLSEYIQSHGESGWRSLPKKAGLNRCGKSCRLRWLNYLRPDIKRGNISPDEEELIIRMHRLLGNRWSLIAGRLPGRTDNEIKNYWNTHLSKKL
Picea abies V1.0	MA_15687g0010	Pa2R-MYB056	EQSVRRGPWTVDEDMNLIRCVTTRGEGRWNTVAKWAGLKRTGKSCRLRWLNYLRPDVKRGNITPEEQILILELHRLWGNRWSKIARQLPGRTDNEIKNYWRTRIKKHI
Picea abies V1.0	MA_158099g0010	Pa2R-MYB057	QQKVKRGLWSPDEDEKLINYITKYGYGCWSEVPEKSGLQRCGKSCRLRWINYLRPDIRRGNFSPNEEKLIIHLHTMVGNRWAYIASHLPGRTDNEIKNYWNSWIKKKL
Picea abies V1.0	MA_158319g0010	Pa2R-MYB058	KQAIKRGFWSPEEDKKLICYITAHGHGCWSSLPDRAGLQRCGKSCRLRWMNYLRPGIKHGNFSKEEENIIICLHKVTDNRWAQIAKHLPGRTDSEIKNYWNSYLKKKL
Picea abies V1.0	MA_1589g0010	Pa2R-MYB059	QQKVKRGLWSPEEDEKLINYINNNGYGCWSEVPEKAGLQRCGKSCRLRWINYLRPNIRRGKFTPEEEKLIISLHNIVGNRWAHIATHLPGRTDNEIKNYWNSWIKKKL
Picea abies V1.0	MA_16413g0010	Pa2R-MYB060	KEGLNRGSWTREEDMILSGYIQTHGDGDWRNLPKNAGLKRCGKSCRLRWINYLRPDIKRGSISPDEEDLIIRLHRLLGNRWSLIAGRLPGRTDNEIKNYWNTHLSKKL
Picea abies V1.0	MA_164713g0010	Pa2R-MYB061	IEGLNRGSWTKKEDIILSEYIRVHGDGGWTNLPQRAGLRRCGKSCRLRWLNYLRPDIKRGNISPYEEDLIVRLHRLLGNRWSLIAGRLPGRTDNEIKNYWNTRLSKKL
Picea abies V1.0	MA_173020g0010	Pa2R-MYB062	EDGLNRGAWTAGEDKILSEYVKTHGVGRWRSLPKKAGLRRCGKSCRLRWLNYLRPDIKRGNISPEEEELIIRLYRLLGNRWALISGRLPGRTDNEIKNYWNTHLSKKV
Picea abies V1.0	MA_173523g0010	Pa2R-MYB063	KNGLKKGPWTVEEDQILINYIQKNGHASWRALPXXXARLLRCGKSCRLRWTNYLRPDIKRGSFTQEEERTIIQLHGILGNRWSAIASQLPGRTDNEIKNFWGTRLKKRL
Picea abies V1.0	MA_175772g0010	Pa2R-MYB064	KQGLNRGAWTKVEDMILSDYIRSHGDSEWRNLPRKAGLKRCGKSCRLRWKNYLRPDIKRGNISQDEEDLIFRLHRLLGNRWSLIAGRLPGRTDNEIKNYWNTRLSKKL
Picea abies V1.0	MA_17672g0010	Pa2R-MYB065	KANVKKGPWSPEEDAKLKAFIEQHGTGGNWIALPQKAGLKRCGKSCRLRWLNYLRPDIRHGGFSEDEDSIICGLYASIGSRWSIIAAQLPGRTDNDIKNYWNTRLKKKL
Picea abies V1.0	MA_179894g0010	Pa2R-MYB066	EVGLNHGAWTAKEDKILSEYIKTHGLGPWNSLAKKAGLKRCGKSCRLRWLNYLRPDIKRGNISPEEEELLIRLHLLLGNRWSLIAGRLPGRTDNELKNYWNTHLSKRF
Picea abies V1.0	MA_180449g0010	Pa2R-MYB067	EDGLNRGAWTAGDDKILSEYIKTHGVGRWRSLPKKAGLRRCGKSCRLRWLNYLRPDIKRGNISPEEEELIIRLYRLLGNRWALISGRLPGRTDNEIKNYWNTHLSKKV
Picea abies V1.0	MA_183667g0010	Pa2R-MYB068	DEGFNRAAWTTKEDMILRDYINIHGLEGGWRSVRNKTGLNRSGKSCRLRWTNYLRPNIKRGNISTDEEELIIRLHRLLGNRWSLIAGRLPGRTDNEIKNYWNTQLRKKI
Picea abies V1.0	MA_190973g0010	Pa2R-MYB069	KAYTNKGAWTQEEDACLIAHIRTHGEGAWRSLPRAAGLLRCGNSCRLRWINYLRPNLKRGNFSEEENDLIIRLHNLLGDKWSLIAGRLPGRTDNEIKNYWNTHIKKTK
Picea abies V1.0	MA_19163g0010	Pa2R-MYB070	KSHTNKGAWTKEEDHRLVAHIRAHGEGCWRSLPMAAGLLRCGKSCRLRWINYLRPDLKRGNFSEEEDELIIKLHSLIGNKWSLIAARLPGRTDNEIKNHWNSHIKRKL
Picea abies V1.0	MA_195086g0010	Pa2R-MYB071	YAGLNKRPWTPEEDLRLVKYIQAHGEGNWRTLPKKAGLQRCGKGCRLRWMNYLRPNVKRGHISPEEEEMIIRLHKLLGNRWSLIAGRIPGRTDNEVKKYWNTHLRKKF
Picea abies V1.0	MA_197412g0010	Pa2R-MYB072	KPKLRKGLWSPEEDRKLLTYVMKKGMLGCSWNYVAKQAGLKRCGKSCRLRWINYLRPDLKRGEFSFQEEQLIIHLHSILGNRLPGRTDNDIKNYWNSCVKRKK
Picea abies V1.0	MA_199974g0010	Pa2R-MYB073	KSHTNKGAWTKEEDDRLIAHIRAHGEGCWRSLPKDAGLLRCGKSCRLRWINYLRPDLKRGNFSEEEDELIIKLHSILGNKWSLIAARLPGRTDNEIKNYWNSHIKRKL
Picea abies V1.0	MA_202088g0010	Pa2R-MYB074	KQKFKKGLWSPEEDRSLKAYVTKYGHGCWSSVPKQAGLQRCGKSCRLRWINYLRPGLKRGRFSLLEERTIIEMHAAIGNRWSQIARHLPGRTDNEIKNFWNSYLKKKL
Picea abies V1.0	MA_20462g0010	Pa2R-MYB075	EGALKKGPWTAAEDAILVAYVKENGEGSWNSVQKHSGLSRCGKSCRLRWANHLRPNLKKGAFTPDEERMIIDLHAKLGNKWARMAALMPGRTDNEIKNYWNTRIKRRM
Picea abies V1.0	MA_21440g0010	Pa2R-MYB076	KEGLNRGAWTKKEDMILSEYIRIHGDGGWRNLPKKAGLKRCGKSCRLRWLNYLRPDIKRGNICPDEEELIIRLHRLLGNRWSLIAGRLPGRTDNEIKNYWNTHLSKKL
Picea abies V1.0	MA_22140g0010	Pa2R-MYB077	NGDRNKGVWTKEEDDRLIQYIQAHGEGCWRSLPKAAGLLRCGKSCRLRWINYLRPDLKRGSFSEDEDDLILKLHALLGNKWSLIAGRLPGRTDNEIKNYWNSHLKRKL
Picea abies V1.0	MA_223201g0010	Pa2R-MYB078	NGDRNKGAWTREEDDRLIQYIQAHGEGCWRSLPNAAGLLRCGKSCRLRWINYLRPNLKRGDFSEDEDDLILKLHALLGNRWSLIAGRLPGRTDNEIKNYWNSYLKRKL
Picea abies V1.0	MA_23242g0010	Pa2R-MYB079	KQGLNRGAWTVKEDMILSQYIRLHGDGGWRNLPRKAGLSRCGTSCRLRWTNYLRPDIKRGNISPDEEDLIVRMHRLLGNRWSLIAGRLPGRTDNEIKNYWNTRLNKKL
Picea abies V1.0	MA_23348g0020	Pa2R-MYB080	ETHTKKGPWTVKEDMEKIRYISLHGEGRWSFLAKSTGLKRSGKSCRMRWVNYLRLTLKRSKITREEERLIIELHGRLGNRWSRIAQSLPGRTDNDIKNYWRTRIEKKI
Picea abies V1.0	MA_24271g0020	Pa2R-MYB081	KDGLKKGPWTPEEDQKLTDYIQRHGHGSWRTLPKHAGLLRCGKSCRLRWTNYLRPDIKRGKFSFEEEQTIVQLHGVLGNKWSAIASHLPGRTDNEIKNYWNTHLKKRL
Picea abies V1.0	MA_246142g0010	Pa2R-MYB082	KAHTNKGAWTKEEDHRLIAHIRTHGEGGWRSLPKAAGLLRCGKSCRLRWINYLRPDLKRGSFTEEENELIIKLHSVVGNKWSLIAGRFPGRTDNEIKNYWNTHIKKKL

Picea abies V1.0	MA_246817g0010	Pa2R-MYB083	KEGLNRGTWTAQEDVILSDYIRIHGDGEWKNLPQKAGLKRCGKSCRLRWVNYLRPXXXXXXXXXXXXXXXIKRGNISPDEEELIIRMHRLLGNRWSLIAGRLPGRTDNDIMHYWNIHLRKKL
Picea abies V1.0	MA_278282g0010	Pa2R-MYB084	KVRLNRGAWTIKEDTILSEYIRTHGEGGWTNLPQNAGLKRCGKSCRLRWLNYLRPNIKRGKISPDEDDLIIRMHNLLGNRWSLIAGRLPGRTDNEIKNYWNTHLGKKL
Picea abies V1.0	MA_2797g0010	Pa2R-MYB085	KAHTNKGAWTKEEDEKLIAYIQAHGEGCWRSLPKAAGLLRCGKSCRLRWINYLRPDLKRGNFSEEEDELIIKLHALLGNKWSLIAGRLPGRTDNEIKNYWNTHIKRKL
Picea abies V1.0	MA_30848g0010	Pa2R-MYB086	AGKLKKGPWTYEEDIRLLRYITIHGPGRWSALAKASGLQRCGKSCRLRWVNYLRPDLKRGSITPEEESIILDLHARWGNRWSLIAEKMPGRTDNEIKNYWRSHLKKKL
Picea abies V1.0	MA_308669g0010	Pa2R-MYB087	KAHTNKGAWTPEEDARLIAHIRAHGEGCWRSLPRAAGLLRCGKSCRIRRINYPRPDVKRGNFSEEEDEFIIKLYSTLGNKWSLIARRLPGRTDNEIKNYWNTYIKKKL
Picea abies V1.0	MA_322432g0020	Pa2R-MYB088	EAETRKGPWTVQEDTQLIGYIRLHGEGRWSFLAKAAGLKRTGRSCRLRWVNCLRPDLKRNKISPEEESLIMELHGRWGNRWSHIARNLPGRTDNEIKNYWRTRIKGKL
Picea abies V1.0	MA_323706g0010	Pa2R-MYB089	KEGLNRGAWTKTEDIILCEYIRIHGDGGWRTLPKKAGLKRCGKSCRLRWLNYLRPDIKRGGISPDEEELIIRLHRLLGNRWSLIAGRLPGRTDNEIKNYWNTCLSKKL
Picea abies V1.0	MA_328440g0010	Pa2R-MYB090	MILSDYIRIHGDGEWRNLPQKAGLRRCGKSCRLRWMNYLRPDIKRGNISPYEEDLIVRLHRLLGNQWSLIAGRLPGRTDNEIKNYLNTRLRKKM
Picea abies V1.0	MA_33964g0010	Pa2R-MYB091	KVGLKKGPWTAEEDQKLISFILHNGHCCWRAVPKLAGLLRCGKSCRLRWTNYLRPDLKRGLLSESEEQLIIDLHSQLGNRWSKIAGHLPGRTDNEIKNYWNTHIKKKL
Picea abies V1.0	MA_34517g0010	Pa2R-MYB092	KPKLRKGLWSPEEDERLVTCIMKNGLLGYSWTYVANQAGLQRCGKSCRLRWINYLRPGLKRGAFSPQEERLIIHLQSILGNRWAQIAASLPGRTDNEIKNHWNTLIKKNK
Picea abies V1.0	MA_357440g0010	Pa2R-MYB093	RRRLRKGLWSPDEDNKLTNYIIRNGLMGCWNYVAKQAGLQRCGKSCRLRWINYLRPGLKRGPISRQEERLIIHLQSSLGNSRWSQIAAHLPGRTDNEIKNYWNSSIKKKI
Picea abies V1.0	MA_358042g0010	Pa2R-MYB094	HEGLKSGPWTAEEDQKLIDHVQRHGLGNWRTLPKLAGLSRCGKSCRLRWANYLRPDIKRGTFSMQEDLTVIRLHSVLGNKWSAIASHLPGRTDNEIKNHWNTKLKKRL
Picea abies V1.0	MA_363933g0010	Pa2R-MYB095	YKICARGHWRIVEDAKLKDLLAQYGPQNWNLIAKKLEGRSHKNHFYVSRLDPRINRKPFSEEEEDRLLATHHLYGNKWAMIARIFPGRTDNVVKNHWHVIMARKL
Picea abies V1.0	MA_3665g0010	Pa2R-MYB096	KQKLRKGLWSPEEDEKLMRYVTTHGHGRWSAVPKHAGLERCGKSCRLRWINYLRPDLKRGTFSAHEEKLITDLHAALGNRWSQIATHLPGRTDNEIKNFWNSCIKKKL
Picea abies V1.0	MA_37058g0010	Pa2R-MYB097	EEGLHRGAWTGSEDKILSEYIKTHGVGRWEDLPKKAGLKRGGKSCRLRWLNYLRPDIKRGNISPEEEELLFRLHRLLGNRWSLIAGRLPGRTDNEIKNYWNTQLSKRV
Picea abies V1.0	MA_37256g001	Pa2R-MYB098	KQKVKRGLWSPEEDEKLIKYVTAYGHGCWSVVPKQAGLQRCGKSCRLRWINYLRPDLKRGSFSLQEERLIIDLHKIIGNRWAQIAKHLPGRTDNEVKNFWNSCIKKKL
Picea abies V1.0	MA_37851g0010	Pa2R-MYB099	TPKLRKGLWSPEEDEKLITYIMKKGMLGCSWTNIAKQAGLQRCGKSCRLRWINYLRPDLKRGSFSPQDEQLIRHLHSILGNRWSQIAASLPGRTDNEIKNYWNTCIKKKK
Picea abies V1.0	MA_41194g0010	Pa2R-MYB100	SKPKLRKGLWSPGEDSKLTEYIMKNGLMSCWSYMAKQAGLQRCGKSCRLRWINYLRPGLKRGPFSHQEERLIIHLQSMLGNRWARIATQLPGRTDNEIKNYWNSCIKKII
Picea abies V1.0	MA_437179g0010	Pa2R-MYB101	ETGLKKGPWTPEEDQILLNYIRQNGPGSWRAVPKLAGLRRCGKSCRLRWTNYLRPDIKRGPFTQEEERIIILLHVILGSRWSVIASQLPGRTDNEIKNFWNTHLKKRL
Picea abies V1.0	MA_44263g0010	Pa2R-MYB102	KQKPRKCLWSPEEDHKLTNYIKKHGLLSCWSYVAKQAGLQRCGKSCRLRWINYLRPGLKRGAFSIHEERLIVHLQSILGNRWSQIATHLPGRTDNEIKNFWNSCIKKKL
Picea abies V1.0	MA_446064g0010	Pa2R-MYB103	KEGLNRGVWTAKEDNILSEYIRIHGDGGWRNLPKKAGLKRCGKSCRLRWVNYLRPDIIRGNISPDEEELIIRMHRLLGNRWSLIAGRLRGRTDNEIKNYWNTHLSKKL
Picea abies V1.0	MA_45091g0010	Pa2R-MYB104	KEGLNRAAWTKTEDMILSQYIRVHGDRGGWTNLAKKAGLKRTGKSCRLRWLNYLRPDIKRGNISADEDELIIRLHRLLGNRWSLIAGRLPGRTGNEIKNYWNTHLSKKL
Picea abies V1.0	MA_460508g0010	Pa2R-MYB105	KMAVKKGPWNPEEDRKLMDYIQKYGFGNWRALPKQAGLMRCGKSCRLRWMNYLRPDLKRGSLSPEEEQTIVRLHRLLGNRWSTIASCLPGRTDNEIKNIWNSNLKKRF
Picea abies V1.0	MA_46261g0010	Pa2R-MYB106	KPKIRKGLWSPEEDAKLISYITKNDLVDCSWNLIANQAEGCKEAYNRAMNVHTGLQRHGKSCRLRWKNYLRPGLKRGGISHQEKQLIIGLQSILGNRWSQIAAHLPGRTDNEIKNFWNTSIKKTL
Picea abies V1.0	MA_4704g0010	Pa2R-MYB107	SRLCARGHWRPAEDAKLKELVSQYGPQNWNLIAEKLEGRSGKSCRLRWFNQLDPRINRRPFSEEDEEKLLAAHRLYGNKWAMIARLFPGRTDNAVKNHWHVIMARRY
Picea abies V1.0	MA_48006g0010	Pa2R-MYB108	QAHTNKGAWTQEEDARLIAHIRTHGEGGWSSLPKAAGLRHCRKSCRFRWKNYLRPDLKRGNFSEGEDELIIKLHSLLGNKWSLIAGRLPGRTDNEIKNYWSTHIKRKV
Picea abies V1.0	MA_51173g0010	Pa2R-MYB109	KANVKKGPWSPEEDSELKEYIAKYGTGGNWIALPQKVGLKRCGKSCRLRWLNYLRPNIKHGGFSEEEDNIICSLYLSIGSRWSIIAAQLPGRTDNDIKNYWNTKLKNKL
Picea abies V1.0	MA_53373g0010	Pa2R-MYB110	KVGLHRGPWTAREDLLLTKYIEAHGEGQWRALPKQAGLLRCGKSCRLRWMNYLRPDIKRGNITLDEEELIVRLHKLLGNRWSLIAGRLPGRTDNEIKNFWNTHLSKKL
Picea abies V1.0	MA_53573g0010	Pa2R-MYB111	HEGFNRGAWTAKEDMILTEYIKTHGVGRWRSLPRKSGLKRGGKSCRLRWLNYLRPDIKHGNISPEEEELLIRLHRLLGNRWSLIAGRLPGRTDNEIKNYWNTHLRKKV
Picea abies V1.0	MA_53644g0010	Pa2R-MYB112	MMGVRKGPWSSEEDRKLIDYIEKHGFGNWRALPKQAGVMRCGKSCRLRWMNYLRPDIKRGSLSPDEEQTIIQLHGILGNKWSTIASYLPGRTDNEIKNLWNTHLKKRL
Picea abies V1.0	MA_55123g0010	Pa2R-MYB113	KAHTNKGAWSKEEDEVLIAYIQAHGEGCWRSLPKAAGLQRCGKSCRLRWINYLRPDLKRGNFSPEEDDLIIKLHSMLGNKWSLIASRLPGRTDNEIKNYWNTHIKRKL
Picea abies V1.0	MA_55503g0010	Pa2R-MYB114	GLNRGMWTRDEDLRLRNYIEANGEGGWRTLPMKAGLLRCGKSCRLRWMNYLRPDVKRGHILPDEEDLIVRLHRLLGNRWSLIAGRIPGRTDNEIKNYWNTHLSKKL
Picea abies V1.0	MA_62361g0010	Pa2R-MYB115	KVGLKKGPWTAEEDRKLVNFITMHGHGCWREVPKLAGLLRCGKSCRLRWTNYLRPDLKRGLLSESEEKLIIDLHAAIGNRWSRIAAQLPGRTDNEIKNYWNTRIKKKL
Picea abies V1.0	MA_62488g0010	Pa2R-MYB116	KMGIRKGPWSLEEDRKLIDYIEKHGFGNWRALPKQAGLLRCGKSCRLRWTNYLRPDLKHGSLSSEEEQTIIRLHGILGNRWSTIASYLPGRTDNEIKNIWNTHLKKRL
Picea abies V1.0	MA_62687g0010	Pa2R-MYB117	KDGLNKGPWTQDEDLRLIQFIQAHGEGRWPAVAKNAGLKRCGKSCRFRWVNYLRPNLKRGNISADEEDLIIRLHKLLGNRWSLIAGRLPGRTDNEIKNYWNTRSNKKA
Picea abies V1.0	MA_6285g0010	Pa2R-MYB118	KTGLKKGTWSPEEDKILVNYIENNGHGSWRALPKLAGLLRCGKSCRLRWSNYLRPDIKRGSFTREEERTIIQLHAILGNRWSIIASQLPRRTDNEIKNFWNTNLKKRL
Picea abies V1.0	MA_6324g0010	Pa2R-MYB119	YAGLNKGPWTREEDLRLIKYIEAYGEGNWRTVPRKAGLLRCGKGCRLRWMNYLRPNVKRGHISPDEEEMIIRLHNLLGNRWSLIAGRIPGRTDNEVKNYWNNHLRKKF
Picea abies V1.0	MA_6393g0010	Pa2R-MYB120	KANVKKGPWSPEEDSKLKEYIDKYGTGGNWIALPQKVGLKRCGKSCRLRWLNYLRPNIKHGGFSEEEDNIICNLYISIGSRWSIIAAQLPGRTDNDIKNYWNTRLKKKL
Picea abies V1.0	MA_64405g0010	Pa2R-MYB121	KEGLNRGAWTSKEDMILSEYVRIHGDGRWRNLPQKAGLMRCGKSCRLRWLNYLRPDIKRGNISPDEEELIIRLHRLLGNRWSLIAGRLPGRTDNEIKNYWNTRLNKKR
Picea abies V1.0	MA_66255g0010	Pa2R-MYB122	NIGLKKGPWTPEEDQKLIDYIQRHGHGSWRALPKRAGLLRCGKSCRLRWTNYLRPDIKRGQFSFEEEQTIIELHAVLGNKWSTIAGHLPGRTDNEIKNYWNTHLKKRL
Picea abies V1.0	MA_66845g0010	Pa2R-MYB123	KPMSNKGRWSPEEDNKLTNYIMKNGLLGGWNYVGKKAGLQRCGKSCRLRWINYLRPGLKRGAFSYQEEQLIIHLQSIFGNRWSLIATQLPGRTDNELKNYWNSGLKKKL
Picea abies V1.0	MA_67705g0010	Pa2R-MYB124	KDNVKRGPWSPEEDSKLKEYIEKYGTGANWISLPQKVGLRRCGKSCRLRWLNYLRPNLKHGGFSEEEDNIICSLYLSIGSRWSMIAARLPGRTDNDIKNHWNTRLKKKL
Picea abies V1.0	MA_692795g0010	Pa2R-MYB125	KTGLKKGPWTPKEDKILADYIEKNGDGSWRGLPKLAGLLRCGKSCRLRWTNHLKPGIKRGSFTIEEEQTLISLHAIVGNRWSAIASQIPGRTDNGIKNFWNTHLKKRL
Picea abies V1.0	MA_69407g0010	Pa2R-MYB126	KQGLNRGAWTEKEDMILSEYIRIHGDGKWRYLPEKAGLKRCGKSCRLRWVNYLHPDIKRGNISPYEEELIIRLHRLLGNRWSLIAGRLPGRTDSEIKNYWNCHLGKKL

		Picea abies V1.0	MA_7055g0010	Pa2R-MYB127	NAGLNRGPWTLEEDLRLTNYIEAHGEGGWTNLPKKAGLLRCGKSCRLRWMNYLRPDVKRGHILLDEEDLILRLHRLLGNRWSLIAGRMPGRTDNEIKNYWNTHLSKKL
		Picea abies V1.0	MA_7115g0010	Pa2R-MYB128	KEGLNRGAWSRKEDMILSEYIRIHGDGGWTNLPQRAGLKRCGKSCRLRWTNYLRPDIKLGNISPDEDELIIRMHRLLGNRWSLIAGRLPGRTDNEIKNYWNTRLSKKL
		Picea abies V1.0	MA_73502g0010	Pa2R-MYB129	EDETRKGPWTVEEDMQLTEYIRLHGEGRWSFVAKGAGLKRTGRSCRLRWVNSLRPGLKRSKITPEEERLIIELHDRWGNRWSHIARSLPGRTDNETKNYWRTRIKGKL
		Picea abies V1.0	MA_80033g0010	Pa2R-MYB130	KSNVKKGPWSREEDSKLKEYMDKCGAGGNWLALPQKVGLKRCGKSCRLRWLNYLRPNIKHGGFSEEEDHIICSLYIRIGSRWSIIAAQLPGRTDNDIKNHWNTRLKKKV
		Picea abies V1.0	MA_8069842g0010	Pa2R-MYB131	KPKLRKGLWSPEEDEKLIAYIANNGMVGCSWGYVAKQAGLQRCGKSCRLRWINYLRPGLKRGVFSHQEERLIINLQSIIGNRWSQIAAQIPGRTDNEIKNYWNSTIKRKL
		Picea abies V1.0	MA_8147g0020	Pa2R-MYB132	KEGLNRGAWTKKEDMILSEYIRIHGDGGWRNIPKKAGLKRCGKSCRLRWLNYLRPDIKRGNISPDEEELIVRLHRLLGNRWSLIAGRLPGRTDNEIKNYWNTHMSKKL
		Picea abies V1.0	MA_81763g0010	Pa2R-MYB133	NGDRNKGAWTKEEDDRLIQYIQVHGEGCWRSLPKAAGLLRCGKSCRMRWINFLRPHLKQGNFSEDEDDLILKLHALLGNKWSLIAGRLPGRTDNEIKNYWNSHLQRKL
		Picea abies V1.0	MA_8206949g0010	Pa2R-MYB134	KMGVKKGPWSPEEDRKLIDYVQKHGFGNWRAIPRQAGLLRCGKSCRLRWTNYLRPDLKRGSLSPEEEQTILRLHGIIGNRWSTIASYLPGRTDNEIKNIWNTHLKKRL
		Picea abies V1.0	MA_82197g0010	Pa2R-MYB135	KVGVKKGPWTPEEDIALVSYIQEHGPGNWRLIPARTGLLRCSKSCRLRWTNYLRPGIRRGNFTPYEDRIIIHLQALLGNRWAAIASYLPQRTDNDIKNYWNTHLKKRL
		Picea abies V1.0	MA_8356281g0010	Pa2R-MYB136	KVGLHRGPWTAREDLLLTKYIEAHGEGQWRALPKQAGLLRCGKSCRLRWMNYLRPDIKRGNITLDEEELIVRLHKLLGNRWSLIAGRLPGRTDNEIKNFWNTHLSKKL
		Picea abies V1.0	MA_83918g0010	Pa2R-MYB137	KTGLKKGPWTCEEDQILVNYIQKNGHGSWRALPKLAGLRRCGKSCRLRWTNYLRPDIKRGLFTEEEERTILQLHAILGNRWSGIAAQLPGRTDNEIKNHWNTHLKKRL
		Picea abies V1.0	MA_85363g0010	Pa2R-MYB138	KAEIKKVLWSPEEDEKLFNYIMKNGLLGSTWTYVAKKAGLRRSGKSCRLRWFNYLRPGLIRGAISPQEGHFIIHLQSILGNRWCEIAARLPGRTDSQIKNYWNSCIKKKL
		Picea abies V1.0	MA_8626g0010	Pa2R-MYB139	KEELSRGAWTAMEDVILSRYIRIHGDGGWRNIPKRAGLKRCGKSCRLRWLNYLRPDIKRGNISPDEEELIIRLHRLLGNRWSLIAGRLPGRTDNEIKNYWNTHISKKL
		Picea abies V1.0	MA_87695g0010	Pa2R-MYB140	QAETRKGPWTVEEDVQLKGYIHLHGEGRWSFLAKAAGLKRTGRSCRLRWVNSLRPDLKHSKITPEEERLMIELHGRWGNRWSHIARSLPGRTDNEIKNYWRTRLKGKL
		Picea abies V1.0	MA_8846750g0010	Pa2R-MYB141	SAHTNKGAWTREEDQRLIAYIQTHEEGRWRSLPKAAGFFPALSSSMKLLVACHLIKSLYITSLHLSSAGLLRCGKSCRLRWTNYLRPDLRRGNFSEEEVDLIIELHSSFGNKWSLIAR<br>KLPGRTDNEIKNYWNSSIKKKM
		Picea abies V1.0	MA_89683g0010	Pa2R-MYB142	VAERIKGPWSPEEDATLHRLVEKYGPRNWSQISRGIPGRSGKSCRLRWCNQLSPQVEHRPFSPHEDATIIQAHARHGNKWATIARLLPGRTDNAIKNHWNSTLRRRY
		Picea abies V1.0	MA_93111g0010	Pa2R-MYB143	DTRTRKGPWTIQEDMELTRYISLHGDGHWNFLAKAAGLKRTGKSCRLRWLNYLRPDLKRSKITPEEKHLIIKLHGLLGNRWSRIAQSLPGRTDNEIKNYWRTLIKKKT
		Picea abies V1.0	MA_93127g0010	Pa2R-MYB144	KVGLNRGRWTAEEDEILTKYIQTHGEGSWRSLPQNAGLLRCGKSCRLRWINYLRSDVKRGNICKEEEELIIRLHTLLGNRWSLIAGRMPGRTDNEIKNYWNTHLSRKL
		Picea abies V1.0	MA_93317g0010	Pa2R-MYB145	MARTPLGLNKGSWTRDEDLRLRNYIEAHGEGEWRTLPEKAGLLRGGKSCRLRWMSYLRPDMKRGRILPDEEDLILRMHRLLGNRWSLIAGRIPGRTDNEIKNYWNNHLSKKL
		Picea abies V1.0	MA_9343698g0010	Pa2R-MYB146	KPKLRKGFWSPEEDSKLSEYILKNGLMGCWSYVAKQAERCGKSCRLRWINYLRPGLKRGPISQQEERLILHLQSILGNRWARIATQLPGRTDNEIKNYWNSCIKKRL
		Picea abies V1.0	MA_936250g0010	Pa2R-MYB147	HEGLKSGPWTPEEDQKLIDHVQRHGLGNWRSLPKLAGLSRCGKSCRLRWANYLRPDIKRGTFSMQEDLTVIRLHSVLGNKWSAIASHLPGRTDNEIKNHWNTKLKKRL
		Picea abies V1.0	MA_9374017g0010	Pa2R-MYB148	EMGLKKGPWTPEEDQILISYINKHGHGNWRALPKQAGLMRCGKSCRLRWTNYLRPDIKRGNFSLKEEQTIIHLHQILGNRWSAIASHLPGRTDNEIKNVWNTHLKKRL
		Picea abies V1.0	MA_9381481g0010	Pa2R-MYB149	GEETRKGPWTVEDDMQLMEYIRLHGQGCWSFVAKGAGLKRTGRSCRLRWVNSLRPGLKRSKITPEEERLIIELHDRWGNRWSHIAWSLPGRTDNEIKNYWRTRIKGKL
		Picea abies V1.0	MA_94467g0010	Pa2R-MYB150	QVGLHRGPWTAKEDNLLTRWIEWHGEGDWRNLPEKAGLLRCGKSCRLRWLNYLRPTIKRGNISSDEEDLIIRLHRLLGNRWSLIAGRLPGRTDNEIKNYWNSHLRKKL
		Picea abies V1.0	MA_9483804g0010	Pa2R-MYB151	KVGLKKGPWTPEEDQKLVAYIQEHGHGSWRALPQKAGLLRCGKSCRLRWANYLRPDIKRGKFSTQEEQTIIQLHALLGNRWSTIATHLPKRTDNEIKNYWNTHLKKRL
		Picea abies V1.0	MA_95226g0010	Pa2R-MYB152	KTGIKRGPWTPEEDKILVSYIQKKGHGSWRALPKLAGLLRCGKSCRLRWTNYLRSDIKRGSFTQEEERTIIQLHAILGNRWSAIAAQLPGRTDNEIKNFWNTHLKKRL
		Picea abies V1.0	MA_95339g0010	Pa2R-MYB153	ETQTRKGPWTVEEDMELIKYISVHGEGRWSFLARAAGLKRTGKSCRLRWVNYLRPDLKRSKITPEEELLIIELHGRLGSRWSRIAQSLPGRTDNEIKNYWRTRIKKKM
		Picea abies V1.0	MA_96853g0010	Pa2R-MYB154	TLKKGPWTSAEDAILVEYVKKHGEGNWNAVQKHSGLSRCGKSCRLRWANHLRPNLRKGAFTTEEERKILELHAKLGNKWARMAAQLPGRTDNEIKNYWNTRIKRRQ
		Picea abies V1.0	MA_9818613g0010	Pa2R-MYB155	VDGLNRGAWTANEDKILSEYIKTHGVGRWGDLPKKAGLRRCGKSCRLRWLNYLRPDIKRGNISPEEDELLVRLHRLLGNRWSLIAGRLPSRTDNEIKNYWNTQLSKRV
		Picea abies V1.0	MA_98249g0010	Pa2R-MYB156	KPKIRKRFWAPEEDEKLFNYMMKSRLIRCSWSYVAKQVGIHRCGKSCRLRWINYLRPGLKHGAISPQEEKLIIHLHSILGNRWSQIASHLPGRSDNEIKNYWNSCIKKKL
		Picea abies V1.0	MA_9831998g0010	Pa2R-MYB157	QPQTRKGPWTVQEDMQLIRYISLHGEGRWPYLAKAAGLKRTGKSCRLRWINYLRPDLKRSKITPEEERLIIKLHGRWGNRWSRIAQNLPGRTDNEIKNYWRIRIKKRL
		Picea abies V1.0	MA_98376g0010	Pa2R-MYB158	YAGLNKGPWTPEEDLRLIKYIEAHGQGNWRTLPNKAGLQRCGKGCRLRWMNYLRPNVKRGHISPDEEEMIIRLHKLLGNRWSLIAGRIPGRTDNEVKNYWNTHLRKKF
		Picea abies V1.0	MA_99955g0010	Pa2R-MYB159	KDGLNKGPWTQDEDFRLTQFIQAHGEGRWSALAKNAGLKRCGKSCRLRWMNYLHPNLKRGNISPDEEDLIIRLHKLLGNRWSLIAGRIPGRTDNEIKNYWNTRLNKKA
		Picea abies V1.0	MA_9783816g0010	Pa2R-MYB160	RGQRIKGPWSPEEDASLQKLVEKLGPRNWSLISKGIPGRSGKSCRLRWCNQLSPQVQHRPFSPEEDRMIMEAHSMHGNKWATIARILPGRTDNAIKNHWNSTLRRKC
	Angiosperm	*Amborella trichopoda v1.0*	evm_27.model.AmTr_v1.0_scaffold00001.525	Atr2R-MYB01	KTLLIKGQWSSEEDKMLVRLVDEFGEKKWSQIACRLVGRVGKQCRERWHNHLRPDIKKDAWSEEEERTLVEAHADVGNRWAEIAKCLPGRTENSIKNHWNATKRRQL
		Amborella trichopoda v1.0	evm_27.model.AmTr_v1.0_scaffold00001.72	Atr2R-MYB02	QQKVKRGLWSPEEDEKLVRYITSNGYGCWSEVPEKAGLQRCGKSCRLRWINYLRPDIRRGRFTSEEEKIIINLHAVVGNRWAHIASHLPGRTDNEIKNYWNSWIKKKI
		Amborella trichopoda v1.0	evm_27.model.AmTr_v1.0_scaffold00003.210	Atr2R-MYB03	DSELRRGPWTVEEDMILVNYIANHGEGRWNSLARCAGLKRTGKSCRLRWLNYLRPDVRRGNITPEEQLMILELHSRWGNRWSKIAQHLPGRTDNEIKNYWRTRVQKHA
		Amborella trichopoda v1.0	evm_27.model.AmTr_v1.0_scaffold00003.458	Atr2R-MYB04	KNGLKKGPWTPEEDQKLIDYIKKHGHGSWRTLPKSAGLSRCGKSCRLRWTNYLRPDIKRGRFSFEEEETIIQLHSILGNKWSAIAARLPGRTDNEIKNYWNTHIRKRL
		Amborella trichopoda v1.0	evm_27.model.AmTr_v1.0_scaffold00005.249	Atr2R-MYB05	KQDTNKGAWTKQEDEKLIAYIRKHGEGCWRSLPEAAGLLRCGKSCRLRWINYLRPDLKRGNFAEDEEDLIIKLHALLGNRWSLIAGRLPGRTDNEVKNYWNSHLRRKL
		Amborella trichopoda v1.0	evm_27.model.AmTr_v1.0_scaffold00006.100	Atr2R-MYB06	MDRIKGPWSPEEDEQLRKLVQKHGPRNWSLISKSIPGRSGKSCRLRWCNQLSPQVEHRAFTAEEDETIVRAHAQFGNKWATIARLLSGRTDNAIKNHWNSTLKRKC
		Amborella trichopoda v1.0	evm_27.model.AmTr_v1.0_scaffold00006.298	Atr2R-MYB07	KSGTKKGPWTPEEDQKLVEYIRLLRCGKSCRLRWTNYLRPDIKRGNFSIEEETAIITLHSILGNKWSAIAARLPGRTDNEIKNHWNTHLKKRL
		Amborella trichopoda v1.0	evm_27.model.AmTr_v1.0_scaffold00007.133	Atr2R-MYB08	TMTRVKGSWSPEEDATLSRLVAHHGARNWSAISAGIPGRSGKSCRLRWCNQLSPNVHHRPFSPAEDAAIVEAHARHGNKWATIARLLPGRTDNAIKNHWNSTLRRSA
		Amborella trichopoda v1.0	evm_27.model.AmTr_v1.0_scaffold00010.370	Atr2R-MYB09	QRDRHIVTWTPQEDDLLREQIRINGTESWTAIASKFKDKTSRQCRRRWYTYLNSDFKKGGWSAEEDKLLCEAQKIFGNRWTEIAKVVSGRTDNAVKNRFTTLCKKKA

	Amborella trichopoda v1.0	evm_27.model.AmTr_v1.0_scaffold00010.457	Atr2R-MYB10	KIGVKKGPWTPEEDIILVSYIQEHGPGNWRAVPTNTGLLRCSKSCRLRWTNYLRPGIKRGNFTEHEEKIIIHLQALLGNRWAAIASYLPQRTDNDIKNYWNTHLKKKL
	Amborella trichopoda v1.0	evm_27.model.AmTr_v1.0_scaffold00010.503	Atr2R-MYB11	SKLCARGHWRPAEDAKLKELVALYGPQNWNLIAEKLEGRSGKSCRLRWFNQLDPRINRRAFSEEEEERLMAAHRLYGNKWAMIARLFPGRTDNAVKNHWHVIMARKY
	Amborella trichopoda v1.0	evm_27.model.AmTr_v1.0_scaffold00010.533	Atr2R-MYB12	KVGLKKGPWTPEEDQKLLAYIEEHGHGSWRALPVKAGLLRCGKSCRLRWTNYLRPDIKRGKFSLQEEQTIIQLHALLGNRWSAIATHLPRRTDNEIKNYWNTHLKKRL
	Amborella trichopoda v1.0	evm_27.model.AmTr_v1.0_scaffold00019.457	Atr2R-MYB13	NPDLVKGQWSKEGDDMIIELVLQHGAKNWSTIAQALLGWNGKQCRERWHNHLNPAIHKEAWTPHEELALIRAHQQHGNKWVKLAKFLPGRTNNAIKNNWNSSLKKKL
	Amborella trichopoda v1.0	evm_27.model.AmTr_v1.0_scaffold00022.393	Atr2R-MYB14	NKRLRKGLWSPEEDEKLMRYMMGNGQGCWSDVARNAGLQRCGKSCRLRWINYLRPDLKRGAFSPQEEELIVHLHSILGNRWSQIAMRLPGRTDNEIKNFWNSTIKKRL
	Amborella trichopoda v1.0	evm_27.model.AmTr_v1.0_scaffold00024.221	Atr2R-MYB15	KEGLNRGAWTTQEDKILTDYINTHGEGKWRTLPKKAGLKRCGKSCRLRWLNYLRPDIKRGNISDEEEDLIIRLHKLLGNRWSLIAGRLPGRTDNEIKNYWNTNLGKKV
	Amborella trichopoda v1.0	evm_27.model.AmTr_v1.0_scaffold00024.222	Atr2R-MYB16	KEGLNKGAWTAQEDKILTDYIKTHGEGKWRTLSQSTGLKRCGKSCRLRWLNYLRPDIKRGNFSDQEEDLIIRLHKLLGNRWSLIAGRLPGRTDNEIKNYWNTYLVKKF
	Amborella trichopoda v1.0	evm_27.model.AmTr_v1.0_scaffold00024.223	Atr2R-MYB17	EEGLTKGAWTAQEDTILTRYINTHGEGKWRALPKRSGLKRCGKSCRLRWLNYLRPNIKRGNISCEEEDLIIRLHNLLGNRWSLIAGRLPGRTDNEIKNYWNTNLGKKH
	Amborella trichopoda v1.0	evm_27.model.AmTr_v1.0_scaffold00024.225	Atr2R-MYB18	AKNVNKGAWTAEEDQILSQCIKTHGAKKWQSIPAKAGLKRCGKSCRLRWLNYLRPCIKRGNISEEEEDLIIRLHKLLGNRWSLIAGRLPGRTDNEIKNYWNTYLSKKL
	Amborella trichopoda v1.0	evm_27.model.AmTr_v1.0_scaffold00024.329	Atr2R-MYB19	AKRSGKRLWSHEEDLRLIRHIEAYGKGSWTTLPQKAGLNRNSKSCRLRWMNYLRPDIKHGNFSPEEDDLILRLHRLVENRWSLIAGRVPGRTDSQIKNFWNTRYRGKK
	Amborella trichopoda v1.0	evm_27.model.AmTr_v1.0_scaffold00024.337	Atr2R-MYB20	KLGLNKGPWTPEEDFLLINYINAHGEGGWTNLPKKAGLLRCGKSCRLRWMNYLRPDVKRGYILPEEEDLILRLHRLLGNRWSLIAGRIPGRTDNEIKNYWNTHLSKLL
	Amborella trichopoda v1.0	evm_27.model.AmTr_v1.0_scaffold00024.83	Atr2R-MYB21	MCTRGHWRPSEDDKLKELVDKYGPHNWNAIAEKLQGRSGKSCRLRWFNQLDPRINRSPFTEEEEERLLASHRLHGNRWAIIARLFPGRTDNAVKNHWHVIMARKC
	Amborella trichopoda v1.0	evm_27.model.AmTr_v1.0_scaffold00029.245	Atr2R-MYB22	SDERIKGPWSPEEDAVLSRLVSKFGARNWSLIARGIPGRSGKSCRLRWCNQLDPCVKRKPFTEEEDRTIVAAHAIHGNKWAAIARLLQGRTDNAIKNHWNSTLRRKC
	Amborella trichopoda v1.0	evm_27.model.AmTr_v1.0_scaffold00030.93	Atr2R-MYB23	VGLLRGPWIAREDTLLKRYIETHGEGRWRSLPKKAGLLRCGKSCRLRWMNYLRPNIKRGNIKQDEEELIVRLHALLGNRWSLIAGRLPGRTDNEIKNYWNTHLSKNL
	Amborella trichopoda v1.0	evm_27.model.AmTr_v1.0_scaffold00032.229	Atr2R-MYB24	KSSVRKGPWTPEEDAKILAYISGHGTGNWTAVPKKAGLKRCGKSCRLRWTNYLRPDLKHDRFTPQEEDLIVSLHAAIGSRWSLIAAHLPGRTDNDIKNYWNTKLRKKL
	Amborella trichopoda v1.0	evm_27.model.AmTr_v1.0_scaffold00032.36	Atr2R-MYB25	KVGLNKGAWTPSEDMKLVSYIKNHGHGNWRALPKQAGLLRCGKSCRLRWTNYLRPDIKRGNFSREEEETIIKLHELWGNRWSLIASRLPGRTDNEIKNVWNTHLKKRL
	Amborella trichopoda v1.0	evm_27.model.AmTr_v1.0_scaffold00036.42	Atr2R-MYB26	KPKYKKGLWSPDEDQRLRDYVLRFGHGCWSSVPIRAGLQRNGKSCRLRWINYLRPGLKRGLFTPQEHETILRLHATLGNKWSQIATHLPGRTDNEIKNYWNTYLKKKV
	Amborella trichopoda v1.0	evm_27.model.AmTr_v1.0_scaffold00037.85	Atr2R-MYB27	AASLKKGPWTSAEDAILVEYVKKHGEGNWNAVQKNSGLSRCGKSCRLRWANHLRPNLKKGSFSPEEERLILHLHAKLGNKWARMAAQLPGRTDNEIKNYWNTRIKRRQ
	Amborella trichopoda v1.0	evm_27.model.AmTr_v1.0_scaffold00038.125	Atr2R-MYB28	DNGLKKGPWTPEEDQKLVNYIKKQGHGSWRALPKHAGLNRCGKSCRLRWTNYLRPDIKRGKFSSEEEQTILHLHSILGNKWSAIATHLPGRTDNEIKNFWNTHLKKKL
	Amborella trichopoda v1.0	evm_27.model.AmTr_v1.0_scaffold00038.91	Atr2R-MYB29	GGVLKKGPWTSAEDAVLIEYVKKHGEGNWNAVQKHSGLSRCGKSCRLRWANHLRPNLKKGAFTADEERLIIELHAKIGNKWARMASQLPGRTDNEIKNYWNTRIKRRQ
	Amborella trichopoda v1.0	evm_27.model.AmTr_v1.0_scaffold00039.132	Atr2R-MYB30	DEVNRRGPWTAEEDKLLIQYVRMHGEGRWNSAAESVGLKRSGKSCRLRWVNYLRPDLKKGHITPEEERLIVELHARWGNRWSTIARSLPGRTDNEIKNYWRTHFKKKE
	Amborella trichopoda v1.0	evm_27.model.AmTr_v1.0_scaffold00044.207	Atr2R-MYB31	KANVKKGPWSPEEDAKLKAFIEQHGTGGNWIALPQKIGLKRCGKSCRLRWLNYLRPNIKHGGFSEEEDDIICSLYISIGSRWSIIAAQLPGRTDNDIKNYWNTRLKKKL
	Amborella trichopoda v1.0	evm_27.model.AmTr_v1.0_scaffold00044.255	Atr2R-MYB32	MKERQRWQPEEDALLRAYVKQYGPKEWNLVSQRMGRPLHRDAKSCLERWKNYLKPGIKKGSLSQEEQALVVALQAKYGNKWKKIAAEVPGRTAKRLGKWWEVFKEKQA
	Amborella trichopoda v1.0	evm_27.model.AmTr_v1.0_scaffold00045.155	Atr2R-MYB33	KQKLRKGLWSPEEDEKLLRHITKYGHGCWSSVPKQAGLQRCGKSCRLRWINYLRPDLKRGTFSQQEENMIIELHAVLGNRWSQIAAQLPGRTDNEIKNLWNSCIKKKL
	Amborella trichopoda v1.0	evm_27.model.AmTr_v1.0_scaffold00049.47	Atr2R-MYB34	VGLKRGPWNAEEDKKLMNFILNNGQNCWRLVPKLAGLLRCGKSCRLRWTNYLRPDLKRGALSEFEENKIIELHSRLGNRWSRIASQLPGRTDNEIKNHWNTRIKKKL
	Amborella trichopoda v1.0	evm_27.model.AmTr_v1.0_scaffold00055.19	Atr2R-MYB35	KAHTNKGAWTKEEDERLISHIRAHGEGCWRSLPKAAGLLRCGKSCRLRWINYLRPDLKRGNFTEEEDELIIKLHSLLGNKWSLIAGRLPGRTDNEIKNYWNTHIRRKL
	Amborella trichopoda v1.0	evm_27.model.AmTr_v1.0_scaffold00056.195	Atr2R-MYB36	KANVKRGPWSPEEDNALRNYVQRYGTGGNWIALPQKAGLKRCGKSCRLRWLNYLRPDIKHGGFTEEEDNIIISLYKTIGSRWSVIASQLPGRTDNDVKNYWNTKLKKKL
	Amborella trichopoda v1.0	evm_27.model.AmTr_v1.0_scaffold00067.33	Atr2R-MYB37	RIMIKGGVWKNTEDEILKAAVMKYGKNQWARISSLLVRKSAKQCKARWYEWLDPSIKKTEWTREEDEKLLHLAKLMPTQWRTIAPIVGRTPSQCLERYEKLLDAAC
	Amborella trichopoda v1.0	evm_27.model.AmTr_v1.0_scaffold00068.46	Atr2R-MYB38	KVGMKRGPWSPEEDEVLVAYIAREGEGRWRTLPKRAGLLRCGKSCRLRWMNYLRPCVKRGHIAPDEEDLILRLHRLLGNRWSLIAGRIPGRTDNEIKNFWNTHLSKKL
	Amborella trichopoda v1.0	evm_27.model.AmTr_v1.0_scaffold00068.48	Atr2R-MYB39	KVGMKRGPWTPEEDEVLVAYIAREGEGRWRTLPKRAGLLRCGKSCRLRWMNYLRPCVKRGHIAPDEEDLILRLHRLLGNRWSLIAGRIPGRTDNEIKNFWNTHLSKKL
	Amborella trichopoda v1.0	evm_27.model.AmTr_v1.0_scaffold00068.49	Atr2R-MYB40	KVGMKRGPWTPEEDEVLVAYIAREGEGRWRTLPKRAGLLRCGKSCRLRWMNYLRPCVKRGHIAPDEEDLILRLHRLLGNRWSLIAGRIPGRTDNEVKNFWNTHLSKKL
	Amborella trichopoda v1.0	evm_27.model.AmTr_v1.0_scaffold00068.74	Atr2R-MYB41	KVGLKLGPWTPEEDEVLAAYIGSEGEGRWRTLPKHAGLLRCGKSCRLSWMNYLRPCVKRGRIGADEEDLILRLYRLLGNRWSLIAGRIPGRTDNEIKNYWNTHLSKKL
	Amborella trichopoda v1.0	evm_27.model.AmTr_v1.0_scaffold00076.56	Atr2R-MYB42	KANVKKGPWSPDEDLKLKEYIEKNGTGGNWISLPQKAGLKRCGKSCRLRWLNYLRPNIKHGEFSDEEDRIICNLFASIGSRWSVIAAQLPGRTDNDIKNYWNTKLKKKL
	Amborella trichopoda v1.0	evm_27.model.AmTr_v1.0_scaffold00079.58	Atr2R-MYB43	GGDLRRGPWTLEEDTLLIHYIACHGEGRWNMLAKYSGLKRTGKSCRLRWLNYLKPDIKRGNLTPEEQLLILELHSKWGNRWSRIAQHLPGRTDNEIKNYWRTRVQRQA
	Amborella trichopoda v1.0	evm_27.model.AmTr_v1.0_scaffold00083.33	Atr2R-MYB44	KQKLRKGLWSPQEDEKLMKHITQFGIGCWSSVPKQAGLQRCGKSCRLRWINYLRPDLKRGSFSHQEENIIISLHAVLGNRWSQIAAQLPGRTDNEIKNLWNSCLKKKL
	Amborella trichopoda v1.0	evm_27.model.AmTr_v1.0_scaffold00091.4	Atr2R-MYB45	VGLKKGPWTADEDKKLINFILTNGQCCWRAVPKLAGLLRCGKSCRLRWTNYLRPDLKRGLLSESEEQMVIDIHAYLGNRWSKIAAHLPGRTDNEIKNYWNTHIKKKL
	Amborella trichopoda v1.0	evm_27.model.AmTr_v1.0_scaffold00096.31	Atr2R-MYB46	RPNLVKGQWTLEEDRLLIHLVDQYGVRKWSQIAKMFKGRVGKQCRERWHNHLRPNIKKDTWTEEEEKLLVQAHLEIGNKWAEIARRLPGRTENAIKNHWNATKRRQL
	Amborella trichopoda v1.0	evm_27.model.AmTr_v1.0_scaffold00105.9	Atr2R-MYB47	MLFGPRSWKKIAGFVPGRTQVQCRERWLNSLDPSLKLEEWTDGEDSKLKAAIAEHGYCWSKIASHVHPRTDSQCRRRWKVLFPEEV
	Amborella trichopoda v1.0	evm_27.model.AmTr_v1.0_scaffold00111.43	Atr2R-MYB48	MIDAGAMRKGPWMPEEDEILLEYVRHHGPRDWSAIRSKGLLPRTGKSCRLRWVNKLKPDLKTGCKFSPEEERIVIELQAKFGNKWARIATYLPGRTDNDVKNFWSTRQKRLA

		Amborella trichopoda v1.0	evm_27.model.AmTr_v1.0_scaffold00119.82	Atr2R-MYB49	SKLCARGHWRPAEDAKLKELVAQFGPQNWNVIAERLEGRSGKSCRLRWFNQLDPRINRRAFSEEEEERLLAAHRLYGNKWAMIARLFPGRTDNAVKNHWHVIMARKY
		Amborella trichopoda v1.0	evm_27.model.AmTr_v1.0_scaffold00139.15	Atr2R-MYB50	KAHTNKGAWTKEEDQRLIAYIRAHGEGCWRSLPKAAGLLRCGKSCRLRWINYLRPDLKRGNFTEEEDELIIKLHSLLGNKWSLIAGRLPGRTDNEIKNYWNTHIKRKL
		Amborella trichopoda v1.0	evm_27.model.AmTr_v1.0_scaffold00145.39	Atr2R-MYB51	MACIEIRKGPWTEQEDLQLVCYVGLFGERRWDFIAKVSGLNRSGKSCRLRWVNYLRPDLKRGKITPQEECLILDLQSRWGNRWSRIARKLPGRTDNEIKNYWRTHMRKKA
		Amborella trichopoda v1.0	evm_27.model.AmTr_v1.0_scaffold00186.1	Atr2R-MYB52	QKSSPRGHWRPGEDEKLRQLVEQYGPQNWNSIAEKLQGRSGKSCRLRWFNQLDPRINRRPFTPEEEERLLSAHRHHGNKWALIARLFPGRTDNAVKNHWHVIMARKH
		Amborella trichopoda v1.0	evm_27.model.AmTr_v1.0_scaffold01267.1	Atr2R-MYB53	IEERRRGPWMEEEDIKLAMSVKLIGERRWDHIAQVSGLKRSGKSCRLRWMNYLRPDLKQGRMTAQEECLVLQLHAQWGNKWSRIARRLPGRTDNEIKNYWRTHLRKEA
		Amborella trichopoda v1.0	evm_27.model.AmTr_v1.0_scaffold00012.146	Atr3R-MYB1	EDEILRNAVNYFKGKSWKKIAAYFPHRSEVQCLHRWQKVLNPELVKGPWTKEEDDMIIKLVEKYGAKKWSVIAQSLPGRIGKQCRERWHNHLNPEIKKDAWTVEEELALIRAHQSYGNRWAEIAKFLPGRTDNSIKNHWNSYLKKKL
		Amborella trichopoda v1.0	evm_27.model.AmTr_v1.0_scaffold00109.47	Atr3R-MYB2	TRRSTKGGWTPEEDEILRKAVHYYNGKNWKKIAAFFTDRTDVQCLHRWQKVLNPDLVKGPWSKEEDDMIIELVRQHGAKKWSTIAQALPGRIGKQCRERWHNHLNPAISKEAWTQQEELALIRAHQQYGNKWAELAKFLPGRTDNAIKNHWNSSLKKKL
		Amborella trichopoda v1.0	evm_27.model.AmTr_v1.0_scaffold00009.357	Atr3R-MYB3	TKRSTKGNWTEEEDAILMQAVKQFNGKNWKQIAKCFNDRADVQCLHRWQKVLNPDVVKGPWTKEEDDEIVKQVEKLGTKKWSVVAQSLPGRIGKQCRERWYNQLNPAIKKEPWTQEEDCVLIHAHQIYGNKWAEIAKFIPGRTDNSIKNYWNCTMKKKL
	Monocots	*Zea mays*	AC217264.3_FGT005	Zm2R-MYB001	PVVLKKGPWTASEDAMLMDHVRHHGEGNWNAVQRMTGLLRCGKSCRLRWTNHLRPNLKKGSFSPDEELLIAQLHAQLGNKWARMAAHLPGRTDNEIKNYWNTRTKRRQ
		Zea mays	GRMZM2G024468_T01	Zm2R-MYB002	KAGLKKGPWSPEEDLLLVSYVQEHGPGNWRAVPCSTGLMRCSKSCRLRWTNYLRPGIKRGSFSDQEEKLIIHLQELLGNKWSAIASYLPERTDNDIKNYWNTHLKKKL
		Zea mays	GRMZM2G037650_T01	Zm2R-MYB003	KEGVKRGPWTAEEDKKLISFILTHGRCCWRAVPKLAGLLRCGKSCRLRWTNYLRPDLKRGLLSTAEEQLVIDLHAKLGNRWSQIAAKLPGRTDNEIKNHWNTHIKKKL
		Zea mays	GRMZM2G046443_T01	Zm2R-MYB004	SAWVRKGPWTEDEDEVLRRHVMENGPREWSSIRSKGLLPRTGKSCRLRWVNKLRPDIKKGCKFSADEERVVIDLQAQFGNKWARIATYLSGRTDNDVKNFWSTRQKRLA
		Zea mays	GRMZM2G054111_T01	Zm2R-MYB005	QEGWRKGPWTALEDRLLTEYVQQHGEGSWNSVAKLTGLRRSGKSCRLRWVNYLRPDLKRGKITPDEETVILQLHAMLGNRWSAIARCLPGRTDNEIKNYWRTHFKKAR
		Zea mays	GRMZM2G057027_T02	Zm2R-MYB006	KVGLKRGRWTAEEDQLLANYIAEHGEGSWRSLPKNAGLLRCGKSCRLRWINYLRADVKRGNISKEEEDIIIKLHATLGNRWSLIASHLPGRTDNEIKNYWNSHLSRQI
		Zea mays	GRMZM2G070849_T01	Zm2R-MYB007	AGELRRGPWTVEEDLQLVNYVDAHGEGRWNSLARSAGLKRTGKSCRLRWLNYLRPDLRRGNITPQEQLMILELHSRWGNRWSKIAQHLPGRTDNEIKNYWRTRVQKHA
		Zea mays	GRMZM2G077147_T01	Zm2R-MYB008	MCTRGHWRPSEDEKLKELVALYGPHNWNAIAEKLQGRSGKSCRLRWFNQLDPRINRSPFSEEEEELLLASHRVHGNRWAVIARLFPGRTDNAVKNHWHVIMARRC
		Zea mays	GRMZM2G079123_T01	Zm2R-MYB009	KIGLKKGPWTPEEDQKLLAYIQEHGHGSWRALPVKAGLQRCGKSCRLRWTNYLRPDIKRGKFSLQEEQTIIQLHALLGNRWSAIATHLPNRTDNEIKNYWNTHLKKRL
		Zea mays	GRMZM2G084583_T01	Zm2R-MYB010	KAHTNRGAWTKEEDERLVAYIRAHGEGCWRSLPKAAGLLRCGKSCRLRWINYLRPDLKRGNFTADEDDLIVKLHSLLGNKWSLIAARLPGRTDNEIKNYWNTHVRRKL
		Zea mays	GRMZM2G084799_T01	Zm2R-MYB011	KVGLKRGRWTAEEDQLLANYIAEHGEGSWRSLPKNAGLLRCGKSCRLRWINYLRADVKRGNISKEEEDIIIKLHATLGNRWSLIASHLPGRTDNEIKNYWNSHLSRQI
		Zea mays	GRMZM2G106558_T02	Zm2R-MYB012	KVGLKKGPWTAEEDQKLVSFILGNRQCCWRAVPKLAGLLRCGKSCRLRWTNYLRPDLKRGLLSDAEEKLVIDLHAQLGNRWSKIASHMPGRTDNEIKNHWNTHIKKKL
		Zea mays	GRMZM2G110135_T01	Zm2R-MYB013	ENGLKKGPWTQEEDEKLLHYIQKNGHGSWRTLPRLAGLNRCGKSCRLRWTNYLRPDIKRGKFSREEEQTILHLHSVLGNKWSAIATHLPGRTDNEIKNFWNTHLRKRL
		Zea mays	GRMZM2G121570_T01	Zm2R-MYB014	RAQHHKGPWTEAEDVVLREMVMKHGDRKWAVIAQSLPGRVGKQCRERWTNHLRPDLKKSVWTEEDDMALIKAHKRCGNHWSTIATFLPGRSENAVKNHWNATKRSLK
		Zea mays	GRMZM2G130149_T01	Zm2R-MYB015	REETRKGPWTEQEDLQLVCTVRLFGERRWDFIAKVSGLNRTGKSCRLRWVNYLHPGLKRGRMSPHEERLILELHARWGNRWSRIARRLPGRTDNEIKNYWRTHMRKKA
		Zea mays	GRMZM2G131937_T01	Zm2R-MYB016	KGGVKKGPWTPEEDLVLVSYVQDHGPGNWRAVPTNTGLMRCSKSCRLRWTNHLRPGIKCGNFSDQEEKLIIHLQALLGNRWAAIASYMPERTDNDIKNYWNTHLKKKF
		Zea mays	GRMZM2G143046_T01	Zm2R-MYB017	KPELRRGPWTVDEDLTLVNYIADNGEGRWNNLARAAGLKRTGKSCRLRWLNYLRPDVKRGNFSADEQLLILDLHTRWGNRWSKIAQHLPGRTDNEIKNYWRTRVQKHA
		Zea mays	GRMZM2G147346_T01	Zm2R-MYB018	KEGLRRGAWSAEEDQRLVAYVRQNGHPNWRALPRQAGLLRCGKSCRLRWINYLRPDIKRGNFTPDEEALIVRLHRELGNRWSAIAAQLPGRTDNEIKNVWHTHIKKRL
		Zea mays	GRMZM2G147698_T01	Zm2R-MYB019	KKKLRRGLWSPEEDEKLMNHIAKYGHGCWSSVPKLAGLDRCGKSCRLRWINYLRPDLKRGTFSQEEEDLIIHLHSLLGNKWSQIAAQLPGRTDNEVKNFWNSYIKKRL
		Zea mays	GRMZM2G308034_T01	Zm2R-MYB020	KANVKKGPWTPEEDAKLLAYTSTHGTGNWTNVPQRAGLKRCGKSCRLRYTNYLRPNLKHENFTQEEEDLIVTLHAMLGSRWSLIANQLPGRTDNDVKNYWNTKLSKKL
		Zea mays	GRMZM2G403620_T01	Zm2R-MYB021	MKERQRWRPEEDAVLRAYVRQYGPREWHLVSQRMNVALDRDAKSCLERWKNYLRPGIKKGSLTEEEQRLVIRLQAKHGNKWKKIAAEVPGRTAKRLGKWWEVFKEKQQ
		Zea mays	GRMZM2G428555_T01	Zm2R-MYB022	SRLAARGHWRPAEDAKLRELVALYGPQNWNLIAEKLDGRSGKSCRLRWFNQLDPRISKRPFSDEEEERLMAAHRFYGNKWAMIARLFPGRTDNAVKNHWHVIMARKY
		Zea mays	GRMZM5G870592_T01	Zm2R-MYB023	KANVVKGQWTPEEDRKLVKLVEQFGLRKWSYIAQLLPARVGKQCRERWHNHLRPNIKKDIWSDEEDMVLIEAHKEVGNKWAEIAKRLPGRTENSIKNHWNATKRRQF
		Zea mays	AC165178.2_FGT004	Zm2R-MYB024	KVGVKKGPWTPEEDLMLVSYVQEHGPGNWRAVPTNTGLMRCSKSCRLRWTNYLRPGIKRGNFTDQEEKLIIHLQALLGNRWAAIASYLPERTDNDIKNYWNTHLKKKL
		Zea mays	GRMZM2G032655_T01	Zm2R-MYB025	KEGLKKGPWTPEEDQKLLAYIEQHGHGCWRSLPAKAGLQRCGKSCRLRWTNYLRPDIKRGKFSLQEEQTIIQLHALLGNRWSAIATHLPKRTDNEIKNYWNTHLKKRL
		Zea mays	GRMZM2G038722_T01	Zm2R-MYB026	KVGLNKGSWTPEEDMRLVAYIQKYGHANWRALPKQAGLLRCGKSCRLRWINYLRPDLKRGNFSAEEEETVIKLHGLLGNKWSKIASCLPGRTDNEIKNVWNTHLKKRV
		Zea mays	GRMZM2G048295_T01	Zm2R-MYB027	KMGLKKGPWTPEEDKVLVAHIQSFGHSNWRALPKQAGLLRCGKSCRLRWINYLRPDIKRGNFSKEEEDAIISLHEQLGNRWSAIAARLPGRTDNEIKNVWHTHLKKRL
		Zea mays	GRMZM2G050305_T01	Zm2R-MYB028	KAHTNKGAWTKEEDERLVAHIRAHGEGCWRSLPKAAGLLRCGKSCRLRWINYLRPDLKRGNFTEEEDELIVKLHSVLGNKWSLIAGRLPGRTDNEIKNYWNTHIRRKL
		Zea mays	GRMZM2G064630_T01	Zm2R-MYB029	RKDRHIVSWSSEEDGVLRAQIALHGTDNWTIIAAQFKDKTARQCRRRWYNYLNSECKKGGWSREEDMLLCEAQKVLGNKWTEIAKVVSGRTDNAVKNRFSTLCKRRA
		Zea mays	GRMZM2G087955_T01	Zm2R-MYB030	GVDRIKGPWSPEEDEALQRLVRRHGARNWSLISRSVPGRSGKSCRLRWCNQLSPRVEHRPFTPDEDDAILRAHARFGNKWATIARLLSGRTDNAIKNHWNSTLKRER
		Zea mays	GRMZM2G090837_T01	Zm2R-MYB031	KASVKKGPWSPEEDRKLKEYIDENGTGGNWIALPHKAGLKRCGKSCRLRWLNYLRPNLKHGDFSDDEDRIICSLFATIGSRWSIIAAQLPGRTDNDIKNYWNTKLKKKL
		Zea mays	GRMZM2G105137_T01	Zm2R-MYB032	CEAVRKGHWTAEEDGVLLEHVRVHGPRDWSFIRSKGLLPRTGKSCRLRWVNKLRPDLKTGCKFSAEEERVVLELQAQFGNKWARIATYLPGRTDNDVKNFWSTRQKRLA
		Zea mays	GRMZM2G115859_T01	Zm2R-MYB033	KATVKKGPWAPEEDAVLKGHIDEHGAGGNWIQLPHKIGLNRCGKSCRLRWLNYLRPNIRHGGFTEDEDRLICSLYISIGSRWATIAAQLPGRTDNDVKNHWNTKLKRRL
		Zea mays	GRMZM2G123202_T01	Zm2R-MYB034	SNGIKKGQWALEEDKLLVDYVQANGAGNWRMLPKLAGLNRCGKSCRLRWTNYLRPDIKRGHFTPEEHNSILQLHAIIGNRWSTIAAQLPGRTDNEIKNYWNTHMKQQL
		Zea mays	GRMZM2G124715_T01	Zm2R-MYB035	QAHTNKGAWTKEEDQRLIAYIKAHGEGCWRSLPKAAGLLRCGKSCRLRWINYLRPDLKRGNFSEEEDELIIKFHELFGNKWSLIAGRLPGRTDNEIKNYWNTHIKRKL

Zea mays	GRMZM2G139284_T01	Zm2R-MYB036	KEGVKKGPWTPEEDLVLVSYIQDHGPGNWRAVPTRTGLMRCSKSCRLRWTNYLRPGIRRGNFTEQEEKLIVHLQALLGNRWAAIASYLPERTDNDVKNYWNTHLKRKL
Zea mays	GRMZM2G166337_T01	Zm2R-MYB037	RSGLKKGPWTPEEDEKLVAYIKKHGQGNWRTLPKNAGLARCGKSCRLRWTNYLRPDIKRGRFSFEEEEAIIQLHSILGNKWSAIAARLPGRTDNEIKNYWNTHIRKRL
Zea mays	GRMZM2G176327_T01	Zm2R-MYB038	AGDLRRGPWTVEEDMLLVNYVAAHGEGRWNALARCAGTRCCMLVATLRDDDQTNNTMNCAYAGLRRTGKSCRLRWLNYLRPDLRRGNITAQEQLLILELHSRWGNRWSKIAQHLPGRTDNEIKNYWRTRVQKHA
Zea mays	GRMZM2G001875_T01	Zm2R-MYB039	RAAVKRGPWSPEEDEALRSYVQRHGSGGNWITMPKKAGLKRCGKSCRLRWLNYLRPDIRHGGFTAEEDAVIMSLYAQLGSKWSLIASQMEGRTDNDVKNHWNTKLKKRL
Zea mays	GRMZM2G017520_T01	Zm2R-MYB040	KQKLRKGLWSPEEDEKLMNHITKHGHGCWSTVPKLAGLQRCGKSCRLRWINYLRPDLKRGAFSEEEEDLIVELHAVLGNRWSQIATRLPGRTDNEIKNLWNSSIKKKL
Zea mays	GRMZM2G041415_T01	Zm2R-MYB041	KAHTNKGAWTKEEDQRLVAYIKAHGEGCWRSLPKAAGLLRCGKSCRLRWINYLRPDLKRGNFTQEEDDVIIKLHQVLGNKWSLIAAQLPGRTDNEIKNYWNTHIKRKL
Zea mays	GRMZM2G047626_T01	Zm2R-MYB042	REETRKGPWTEQEDLQLVCTVRLFGERRWDFIAKVSGLNRTGKSCRLRWVNYLHPGLKRGRMSPHEERLILELHARWGNRWSRIARRLPGRTDNEIKNYWRTHMRKKA
Zea mays	GRMZM2G051256_T01	Zm2R-MYB043	KVGLKKGRWTKEEDEVLARYIKEHGEGSWRSLPKNAGLLRCGKSCRLRWINYLRAGLKRGNISEEEEDMIIKLHATLGNRWSLIAGHLPGRTDNEIKNYWNSHLSRRA
Zea mays	GRMZM2G052377_T01	Zm2R-MYB044	EKKVRKGLWSPEEDERLASHIARFGVSCWSSIPELAGLQRCGKSCRLRWMNYLRPDLKRGRFSQQEEDLIIALHKALGNSWSQIAARLPGRSDNEIKNFWNARLRKKI
Zea mays	GRMZM2G064744_T01	Zm2R-MYB045	PPKLRRGLWSPEEDEKLYNHIIRYGVGCWSSVPKLAGLERCGKSCRLRWINYLRPDLKRGSFSQQEEDLIVSLHKILGNRWSQIASQLPGRTDNEIKNFWNSCIKKKL
Zea mays	GRMZM2G083239_T01	Zm2R-MYB046	RAGHARGHWRPAEDAKLKDLVALYGPKNWNLIASKLHGRSGKSCRLRWFNQLDPRVNRRPFSAAEEQRLVAAHRAHGNKWALIARLFPGRTDNAVKNHWHVLTARRQ
Zea mays	GRMZM2G088783_T01	Zm2R-MYB047	KQKVRRGLWSPEEDEKLVKYITAHGHGCWSSVPRQAGLQRCGKSCRLRWINYLRPDLKRGSFSQQEEALIVELHRVLGNRWAQIAKHLPGRTDNEVKNFWNSTIKKKL
Zea mays	GRMZM2G111731_T01	Zm2R-MYB048	GKLCARGHWRPAEDAKLKELVAQYGPQNWNLIAERLDGRSGKSCRLRWFNQLDPRINRRAFSGEEEERLLAAHRAYGNKWALIARLFPGRTDNAVKNHWHVLMARKQ
Zea mays	GRMZM2G139688_T01	Zm2R-MYB049	GTPLKKGPWTSAEDAILVDYVKKNGEGNWNAVQKNTGLFRCGKSCRLRWANHLRPNLKKGAFTPEEERLIIQLHAKMGNKWARMAGHLPGRTDNEIKNYWNTRIKRCQ
Zea mays	GRMZM2G143328_T01	Zm2R-MYB050	EPAVRKGPWTLEEDLILVSYISQHGEGSWDNLARAAGLNRNGKSCRLRWLNYLRPGVRRGSITAGEDTVIRELHARWGNKWSKISKHLPGRTDNEIKNYWRTRIQQKK
Zea mays	GRMZM2G158700_T01	Zm2R-MYB051	AAELRRGPWTVDEDALLAAHVAAHGEGRWNELARDAGLRRTGKSCRLRWLNYLRPGVRRGGFTPREQLLILDLHSRWGNRWSKIAAHLPGRTDNEVKNYWRTRVQKHA
Zea mays	GRMZM2G160838_T01	Zm2R-MYB052	KAHTNKGAWTKEEDQRLIAYIRAHGEGSWRSLPKAAGLLRCGKSCRLRWMNYLRPDLKRGNFTGDDDELIIKLHALLGNKWSLIAGQLPGRTDNEIKNYWNTHIKRKL
Zea mays	GRMZM2G160840_T01	Zm2R-MYB053	KPELRRGPWTVDEDHILVNYIADNGEGRWNSLARAAGLKRTGKSCRLRWLNYLRPDVKRGNFTADEQLLILDLHTRWGNRWSKIAQHLPGRTDNEIKNYWRTRVQKHA
Zea mays	GRMZM2G162709_T01	Zm2R-MYB054	MVELRRGPWTLEEDNLLMNYIACHGEGRWNLLARCSGLKRTGKSCRLRWLNYLKPDIKRGNLAPEEQLLILELHSKWGNRWSRIAQHLPGRTDNEIKNYWRTRVQKQA
Zea mays	GRMZM2G167829_T01	Zm2R-MYB055	KTKVKRGPWSQEEDAVLRSFVQRFGNAGNWIALPQKAGLKRCGKSCRLRWLNYLRPELRHGGFTDEEDSLILSLYGEIGSKWSVIASRLPGRTDNDVKNYWNTKLKKRY
Zea mays	GRMZM2G369799_T01	Zm2R-MYB056	AVEVKKGSWSPEEDALLTRLVEQHGPHRWSLISAPIPGRSGKSCRLRWCNQLSPDVHHRPFTPHEDALILAAHARYGNKWATIARLLPGRTDNSIKNHWNSNLRRCR
Zea mays	GRMZM2G460869_T01	Zm2R-MYB057	GKHIVRGHWRSFEDEKLKELVAQYGPKNWSHISKKLEGRSGKSCRLRWINQLDPHLNHDTFSMVEEERLLVAHHAYGTKWSLIARLFPGRTDNAVKNHWHVMMARKS
Zea mays	GRMZM2G470307_T01	Zm2R-MYB058	NPELVKGPWSKEEDEIIVQMVNKYGPKKWSAIAQALPGRIGKQCRERWHNHLNPAINKEAWTQEEEITLIHAHRMYGNKWAELTKFLPGRTDNAIKNHWNSSVKKKV
Zea mays	GRMZM5G803355_T01	Zm2R-MYB059	RARPSRGHWRPGEDDKLRQLVDKYGPRNWNSIAESLEGRSGKSCRLRWFNQLDPRINRRPFTVAEEELLLRAHRAHGNRWAIISRLFPGRTDNAVKNHWHVVMARRR
Zea mays	GRMZM2G011422_T01	Zm2R-MYB060	KATVKKGPWSPEEDAMLKSYIEEHGTGGNWIALPHKIGLKRCGKSCRLRWLNYLRPNIKHGDFTPEEDGVICSLYISIGSRWSIIAAQLPGRTDNDVKNYWNTKLKKRL
Zea mays	GRMZM2G015021_T01	Zm2R-MYB061	KASVKKGPWSPDEDAKLKAYIEEHGTGGNWIALPHKIGLKRCGKSCRLRWLNYLRPNIKHGDFTEEEEHIICSLYISIGSRWSIIAAQLPGRTDNDIKNYWNTKLKKKL
Zea mays	GRMZM2G017268_T01	Zm2R-MYB062	ENGLKKGPWTQEEDEKLLHYIQKNGHGSWRTLPRLAGLNRCGKSCRLRWTNYLRPDIKRGKFSQEEEQTILHLHSVLGNKWSAIATHLPGRTDNEIKNFWNTHLKKRL
Zea mays	GRMZM2G043792_T01	Zm2R-MYB063	EGGWRKGPWTAQEDKLLVEYVRQHGEGRWNSVAKLTGLKRSGKSCRLRWVNYLRPDLKRGKITPQEESVILHLHAMWGNRWSTIARSLPGRTDNEIKNYWRTHFSKRA
Zea mays	GRMZM2G055158_T01	Zm2R-MYB064	KIGIKKGPWTAEEDQKLVTFLLSHGHCCWRLVPKLAGLLRCGKSCRLRWTNYLRPDLKRGLLSEEEEALVIDLHAQLGNRWSKIAARLPGRTDNEIKNHWNTHIKKKL
Zea mays	GRMZM2G089244_T01	Zm2R-MYB065	QAHTNKGAWTKEEDQRLVAYIKAHGEGCWRSLPKAAGLLRCGKSCRLRWINYLRPDLKRGNFTEEEDDLIIKFHELFGNKWSLIAGRLPGRTDNEIKNYWNTHIKRKL
Zea mays	GRMZM2G108959_T01	Zm2R-MYB066	GVGVKKGPWTPEEDIVLVSYIQQHGPGNWRSVPENTGLMRCSKSCRLRWTNYLRPGIKRGNFTPHEEGIIIHLQALLGNKWAAIASYLPQRTDNDIKNYWNTHLKKKV
Zea mays	GRMZM2G111045_T01	Zm2R-MYB067	NKGLKKGPWTPEEDKLLVDYIQTNGHGSWRLLPKLAGLNRCGKSCRLRWTNYLRPDIKRGPFTAEEEKSIVQLHAIVGNKWSMIAAQLPGRTDNEIKNYWNTHLKKQL
Zea mays	GRMZM2G111117_T01	Zm2R-MYB068	KIGLKKGPWTPEEDEKLLAFVEEHGHGSWRALPAKAGLQRCGKSCRLRWTNYLRPDIKRGKFSLQEEQTIIQLHALLGNRWSAIATHLPNRTDNEIKNHWNTHLKKRL
Zea mays	GRMZM2G127857_T01	Zm2R-MYB069	KMGLKRGPWTAEEDRILVAHVERHGHSNWRALPKQAGLLRCGKSCRLRWINYLRPDIKRGNFSREEEDAIIQLHQMLGNRWSTIAARLPGRTDNEIKNVWHTHLKKRL
Zea mays	GRMZM2G131442_T01	Zm2R-MYB070	DEELRRGPWTVDEDLTLINYIADHGEGRWNALARAAGLKRTGKSCRLRWLNYLRPDVKRGDFTADEQLLILDLHSRWGNRWSKIAAHLPGRTDNEIKNYWRTRVQKHA
Zea mays	GRMZM2G138427_T01	Zm2R-MYB071	KQGVKRGPWTAEEDKKLISFILTHGQCCWRAVPKLAGLLRCGKSCRLRWTNYLRPDLKRGLLSTAEEQLVIDLHAMLGNRWSKIAAKLPGRTDNEIKNHWNTHIKKKL
Zea mays	GRMZM2G162434_T01	Zm2R-MYB072	KEGIKKGPWTPEEDIILVSYIQEHGPGNWRSVPINTGLMRCSKSCRLRWTNYLRPGIRRGNFTPHEEAIIVHLQSLLGNRWAAIASYLPQRTDNDIKNYWNTHLKKKL
Zea mays	GRMZM2G419239_T01	Zm2R-MYB073	KAHTNRGAWTKEEDERLVAYVRAHGEGCWRSLPRAAGLLRCGKSCRLRWINYLRPDLKRGNFTADEDDLIVKLHSLLGNKWSLIAARLPGRTDNEIKNYWNTHIRRKL
Zea mays	GRMZM2G496770_T01	Zm2R-MYB074	KPSYRKGLWSPEEDQKLRDYILLHGHGCWSALPAKAGLQRNGKSCRLRWINYLRPGLKHGTFSPEEEETVMSLHATLGNKWSRIAKHLPGRTDNEVKNHWNSYLKKRV
Zea mays	GRMZM5G833253_T01	Zm2R-MYB075	KVGLNRGSWTPQEDMRLIAYIQKHGHTNWRALPKQAGLLRCGKSCRLRWINYLRPDLKRGNFTDEEEEAIIRLHGLLGNKWSKIAACLPGRTDNEIKNVWNTHLKKKV
Zea mays	GRMZM2G048136_T01	Zm2R-MYB076	REEMRKGPWTEQEDIQLVCTVRLFGDHRWDFIAQVSGLNRTGKSCRLRWVNYLHPGLKHGRMSPQEERIIIELHARWGNRWSRIARRLPGRTDNEIKNYWRTHMRKKA
Zea mays	GRMZM2G001223_T03	Zm2R-MYB077	EAGLKKGPWTSEEDQKLLAFIEQHGHGCWRSLPAKAGLRRCGKSCRLRWTNYLRPDIKRGKFTLQEEQTIIQLHALLGNRWSSIATHLPKRTDNEIKNYWNTHLKKRL
Zea mays	GRMZM2G027697_T01	Zm2R-MYB078	EGGWRKGPWTAQEDKLLVEYVRQHGEGRWNSVAKLTGLKRSGKSCRLRWVNYLRPDLKRGKITPQEESVILQLHALWGNRWSTIARSLPGRTDNEIKNYWRTHFKKAK
Zea mays	GRMZM2G040924_T01	Zm2R-MYB079	TKGLKKGPWTPEEDKLLVDYIQTNGHGSWRLLPKLAGLNRCGKSCRLRWTNYLRPDIKRGPFTSEEQKSIVQLHAIVGNKWSMIAAQLPGRTDNEIKNYWNTHLKKQL

Zea mays	GRMZM2G070523_T01	Zm2R-MYB080	PVVLKKGPWTAAEDAMLMDHVRHHGEGNWNAVQRVTGLLRCGKSCRLRWTNHLRPNLKKGSFSPDEELLIAQLHAQLGNKWARMAAHLPGRTDNEIKNYWNTRTKRRQ
Zea mays	GRMZM2G073836_T01	Zm2R-MYB081	SSKNKKHRWSSEEDEALKQMVKKYGTKNWRTIACAIPGRNANSCLSRWKYLLDPAINKEPWSQQEELRLIRAQQVYGNKWCKMVKHFPGRTNDALKEHWRSPMKRKL
Zea mays	GRMZM2G088189_T01	Zm2R-MYB082	KMNYRKGLWSPEEDQRLRDYILKHGLGCWSAVPAKAGLQRNGKSCRLRWINYLRPGLKRGMFSQEEEDVVIDLQAKLGNKWSQIAMHLPGRTDNEVKNYWNSYLKKRV
Zea mays	GRMZM2G095904_T01	Zm2R-MYB083	KMGLKRGPWTPEEDRILVAHIERHGHSNWRALPKQAGLPRCGKSCRLRWINYLRPDIKRGNFTREEEDAVIQLHQMLGNRWSAIAARLPGRTDNEIKNVWHTHLKKRL
Zea mays	GRMZM2G104789_T01	Zm2R-MYB084	KATVKKGPWSPEEDAKLKSYIEQNGTGGNWIALPQKIGLKRCGKSCRLRWLNYLRPNIKHGGFSDEEDMIILSLYISIGSRWSIIAAQLPGRTDNDIKNYWNTRLKKKL
Zea mays	GRMZM2G145444_T01	Zm2R-MYB085	ECDRIKGPWSPEEDEALRRLVERHGARNWTAIGRGIPGRSGKSCRLRWCNQLSPQVERRPFTPEEDAAILAAHARLGNRWAAIARLLPGRTDNAVKNHWNSSLKRKL
Zea mays	GRMZM2G159547_T01	Zm2R-MYB086	ENGLKKGPWTPEEDQKLMEYIQKNGHGSWRALPRLAGLNRCGKSCRLRWTNYLRPDIKRGKFTQEEEQTILQLHSVLGNKWSAIAKHLPGRTDNEIKNFWNTHLKKKL
Zea mays	GRMZM2G161512_T01	Zm2R-MYB087	SGGLKKGPWTQAEDKLLRDHVRRHGEGNWNAVRRETRLQRCGKSCRLRWANHLRPNLRKCPFSPEEERQILLLHGLIGNKWARISKHLSGRTDNEIKNYWNTRFKRRQ
Zea mays	GRMZM2G170049_T01	Zm2R-MYB088	KPNYRKGLWSPEEDQKLRDYILLHGHGCWSALPAKAGLQRNGKSCRLRWINYLRPGLKHGMFSPEEEETVMSLHATLGNKWSRIARHLPGRTDNEVKNYWNSYLKKRV
Zea mays	GRMZM2G302549_T01	Zm2R-MYB089	NNEFILGNFDRKLVKLVEQFGLRKWSYIAQLLPGRVGKQCRERWHNHLRPNIKKDIWNDEEDSVLIQAHKEVGNRWAEIAKRLPGRTENSIKNHWNATKRRQF
Zea mays	GRMZM2G455869_T01	Zm2R-MYB090	MCTRGHWRPSEDEKLKELVALYGPHNWNAIAEKLQGRSGKSCRLRWFNQLDPRINRSPFSEQEEALLLASHRVHGNRWAVIARLFPGRTDNAVKNHWHVIMARRC
Zea mays	GRMZM2G013581_T01	Zm2R-MYB091	GAAMRKGPWTDEEDEQLVRFVRLFGERRWDFLAKVSGLRRTGKSCRLRWVNYLHPGLRRGRITADEERRIVELHAQWGSRWSRIARSLPGRTDNEIKNFWRTRTRKKA
Zea mays	GRMZM2G048910_T01	Zm2R-MYB092	KVGVKKGPWTAEEDQKLVGFLLTHGHCCWRVVPKLAGLLRCGKSCRLRWTNYLRPDLKRGLLSDDEERLVIDLHAQLGNRWSKIAAQLPGRTDNEIKNHWNTHIRKKL
Zea mays	GRMZM2G069325_T02	Zm2R-MYB093	KASVKRGPWSPEEDEQLRSYVQLNGIGGNWIALPQKAGLNRCGKSCRLRWLNYLRPNIKHGGYTDEEDRIIWSLYSSIGSRWSIIASKLPGRTDNDVKNYWNTKLKKKA
Zea mays	GRMZM2G077789_T01	Zm2R-MYB094	KAHTNKGAWTKEEDERLVAYVRAHGEGCWRSLPKAAGLLRCGKSCRLRWMNYLRPDLKRGNFTDDEDELIIRLHSLLGNKWSLIAGQLPGRTDNEIKNYWNTHIKRKL
Zea mays	GRMZM2G078820_T01	Zm2R-MYB095	ERDRIRGPWSPEEDEALRRLVERHGARNWTAIGRGVPGRSGKSCRLRWCNQLSPRVARRPFTADEDAAIARAHARLGNRWAAIARLLPGRTDNAVKNHWNCSLKRKL
Zea mays	GRMZM2G093647_T01	Zm2R-MYB096	GSRRVNRPWTPEEDELLRRAVARHGPRKWPELSADIPGRCGKSCRRRWVEHLSPGLEHRAFTPEEDAVIVAAQAKHGNKWATIARMLPGRTDNSIKNRWNFALQQQC
Zea mays	GRMZM2G093660_T01	Zm2R-MYB097	GSGRVTRRWTPEEDDLLRRAVARHGPRKWPELSVDIPGRSGRSCHRRWVQHLSPGLERRAFTPEEDAVIVAAQAMYGNKWTTIARMLPGRTRTDISIKNRWNSALQQHC
Zea mays	GRMZM2G093789_T01	Zm2R-MYB098	TPQMKKGPWSPEEDKRLKDYVEVHGAGNWNKVQQNARLNRCGKSCRLRWTNHLRPDLKKEPFNAEEEEKIIKLHIRRGPKWSMMASYLPGRTDNEIKNFWNTRKRRKQ
Zea mays	GRMZM2G102790_T01	Zm2R-MYB099	DAGVKKGPWTPEEDKLLVDYINEHGHGSWRRLPKLAGLNRCGKSCRLRWTNYLRPDIKRGRFTDDEEDLIVHLHSLLGNKWSSIATKLPGRTDNEIKNYWNTHLRKKL
Zea mays	GRMZM2G175232_T01	Zm2R-MYB100	QPKLRKGLWSPEEDEKLYNHIIRHGVGCWSTVPKLAGLQRCGKSCRLRWINYLRPDLKRGSFSQQEEDLIVALHEILGNRWSQIASHLPGRTDNEIKNFWNSCLKKKL
Zea mays	GRMZM2G305856_T01	Zm2R-MYB101	AAAMRKGPWTEEEDAQLVWFVRLFGERRWDFLAKVSGLRRTGKSCRLRWVNYLHPGLRRGRITADEERLILQLHAQWGSRWSRIARSLPGRTDNEIKNFWRTRARKQK
Zea mays	GRMZM2G343068_T01	Zm2R-MYB102	GGGLKKGPWTKAEDKLLRDHVERNGEGNWNAVRRETELQRCGKSCRLRWTNHLRPSLRKGPFSTEEERTILVLHGLIGKKWATIASHLPGRTDNEIKNYWNTRVKRRQ
Zea mays	GRMZM2G423833_T01	Zm2R-MYB103	APQLKKGPWSPEEDKRLKDYVEVHGAGNWNKVQKNAMLNRCGKSCRLRWTNHLRPDLKKDPFDAEEEEKIIKLYIRWGPKWSMMASYLPGRTDNEIKNFWNTRKRRKQ
Zea mays	GRMZM2G701063_T01	Zm2R-MYB104	KEGVKRGAWTAKEDDTLAAYVKAHGEGKWREVPQKAGLRRCGKSCRLRWLNYLRPNIKRGNISYDEEDLIVRLHKLLGNRWSLIAGRLPGRTDNEIKNYWNSTLGRRA
Zea mays	AC213884.3_FGT002	Zm2R-MYB105	CRRRVKGSWTPEEDELLRRAITRHGPRNWTVISSEIPGRSGKSCRLRWCNQLSPSVERRAFTPEEDAIIVAAHAQHGNRWATIARMLHGRTDNSVKNHWNSTLLRQR
Zea mays	GRMZM2G000818_T01	Zm2R-MYB106	KAHTNKGAWTKEEDDRLVAYIKAHGEGCWRSLPKAAGLVRCGKSCRLRWINYLRPDLKRGNFTEEEDELIIKLHSLLGNKWSLIAGRLPGRTDNEIKNYWNTHIRRKL
Zea mays	GRMZM2G031323_T01	Zm2R-MYB107	KSGLKKGPWMPEEDEKLVAYIKKHGQGNWRTLPKNAGLARCGKSCRLRWTNYLRPDIKRGRFSFDEEETIIQLHSILGNKWSAIAARLPGRTDNEIKNYWNTHIRKRL
Zea mays	GRMZM2G045748_T01	Zm2R-MYB108	KMGVKKGPWTPEEDLMLVSYVQEHGPGNWRAVPTNTGLMRCSKSCRLRWTNYLRPGIKRGNFTEQEEKLIVHLQALLGNRWAAIASYLPKRTDNDIKNYWNTHLKKKL
Zea mays	GRMZM2G050550_T01	Zm2R-MYB109	DVDRIKGPWSPEEDEALQRLVARHGARNWSLISRSIPGRSGKSCRLRWCNQLSPQVEHRPFTAEEDDTILRAHARFGNKWATIARLLSGRTDNAIKNHWNSTLKRKY
Zea mays	GRMZM2G056407_T01	Zm2R-MYB110	KEGVKKGPWTPEEDLVLVSYIQEHGPGNWRAVPARTGLMRCSKSCRLRWTNYLRPGIKRGNFTEQEEKLIVHLQALLGNRWAAIASYLPERTDNDIKNYWNTHLKRKL
Zea mays	GRMZM2G104551_T01	Zm2R-MYB111	KLGVKRGPWTAEEDRKLINFILTNGHCCWRAVPKLAGLLRCGKSCRLRWTNYLRPDLKRGLLTDAEEQVVIDLHAKLGNRWSKIAAKLPGRTDNEIKNHWNTHIKKKL
Zea mays	GRMZM2G117244_T01	Zm2R-MYB112	KATVKKGPWAPEEDAVLKAYIDEHGAGGNWIQLPHKIGLNRCGKSCRLRWLNYLRPNIRHGGFTEEEDRLICNLYISIGGRWATIAAQLPGRTDNDVKNHWNTKLKRRL
Zea mays	GRMZM2G126566_T01	Zm2R-MYB113	KVGLKKGPWSAEEDEKLVTFLLTNGQCCWRAVPKLAGLLRCGKSCRLRWTNYLRPDLKRGLLSSEEETTVIDLHAQLGNRWSKIASHLPGRTDNEIKNHWNTHIKKKL
Zea mays	GRMZM2G150841_T01	Zm2R-MYB114	KKKLRRGLWSPEEDDKLVNHIAKYGHGCWSSVPKLAGLERCGKSCRLRWINYLRPDLKRGAFSQEEEDLIIHLHSMMGNKWSQIASQLPGRTDNEVKNFWNSYVKKKL
Zea mays	GRMZM2G169356_T01	Zm2R-MYB115	KVGLKKGPWTAEEDQKLVTFLLINGQCCWRAVPKLAGLLRCGKSCRLRWTNYLRPDLKRGLLSEEEERTVIDLHAQLGNRWSKIASHLPGRTDNEIKNHWNTHIKKKL
Zea mays	GRMZM2G172327_T01	Zm2R-MYB116	KQKVKRGLWSPEEDEKLVKYITAHGHSCWSAVPKHAGLQRCGKSCRLRWINYLRPDLKRGTFSDHEERTIIDVHRILGNRWAQIAKHLPGRTDNEVKNFWNSCIKKKL
Zea mays	GRMZM2G003406_T01	Zm2R-MYB117	QQKLRKGLWSPEEDEKLYNHIIRYGVGCWSSVPKLAGLQRCGKSCRLRWINYLRPDLKRGSFSQQEEDAIVGLHQILGNRWSQIASHLPGRTDNEIKNFWNSCLKKKL
Zea mays	GRMZM2G006352_T01	Zm2R-MYB118	KTSVKRGPWSPEEDDLLRSYVQNHGTGGNWIALPHKAGLNRCGKSCRLRWLNYLRPDIKHGGYTEQEDRIICSLYNCIGSRWSIIASKLPGRTDNDVKNYWNTKLKKKA
Zea mays	GRMZM2G028054_T01	Zm2R-MYB119	GAPLKKGPWTDAEDAILIDYVKKHGVGNWNAVRKNTELLRCGKSCRLRWANHLRPNLKKEAFTPEEERLIIQLHAKLGNKWSRMAIHLPGRTDNEIKNYWNTRKKRCE
Zea mays	GRMZM2G047600_T01	Zm2R-MYB120	AAELRRGPWTVEEDAVLAGHVAAHGEGRWNELACAAGLRRTGKSCRLRWLNYLRPGVRRGGFTPREQLLVLDLHSRWGNRWSRIAAHLPGRTDNEVKNYWRTRVQKHA
Zea mays	GRMZM2G051528_T01	Zm2R-MYB121	KVGLKKGRWTREEDEILARYIEEHGEGSWRSLPKNAGLLRCGKSCRLRWINYLRAGLKRGNITEEEEDVIVKLHATLGNRWSLIAGHLPGRTDNEIKNHWNSHLRRRG
Zea mays	GRMZM2G096358_T01	Zm2R-MYB122	MVELRRGPWTLEEDNLLMNYIACHGEGRWNLLARCSGLKRTGKSCRLRWLNYLKPDIKRGNLTPEEQLLILELHSKWGNRWSRIAQHLPGRTDNEIKNYWRTRVQKQA
Zea mays	GRMZM2G119693_T01	Zm2R-MYB123	QPKLRKGLWSPEEDERLYGHIIRHGVGCWSSVPKLAGLQRCGKSCRLRWINYLRPDLKRGGFSQQEEDLIVALHEILGNRWSQIASHLPGRTDNEIKNLWNSCLKKKL
Zea mays	GRMZM2G151205_T01	Zm2R-MYB124	KTRVKRGPWSQEEDAVLRSFVQRFGNAGNWIALPQRAGLKRCGKSCRLRWLNYLRPELRHGGFTDEEDRLILSLYGEIGSKWSVMASRLPGRTDNDVKNYWNTKLKKRY

Zea mays	GRMZM2G169316_T01	Zm2R-MYB125	IEERVRGPWSPEEDAVLSNLVEKFGARNWTLIARGIPGRSGKSCRLRWCNQLDPQVKRKPFTEEEDRIIMAAHAVHGNKWASIAKLLDGRTDNAIKNHWNSTLRRRY
Zea mays	GRMZM2G171781_T01	Zm2R-MYB126	KQKLRKGLWSPEEDEKLMNHITKHGHGCWSSVPKLAGLQRCGKSCRLRWINYLRPDLKRGAFAQDEEDLIIELHAVLGNRWSQIAAQLPGRTDNEIKNLWNSCIKKKL
Zea mays	GRMZM2G312419_T01	Zm2R-MYB127	GKLCARGHWRPAEDAKLKELVAQFGPQNWNLIAERLDGRSGKSCRLRWFNQLDPRINRRAFSEEEEERLLAAHRAYGNKWALIARLFPGRTDNAVKNHWHVLMARRQ
Zea mays	GRMZM2G322490_T01	Zm2R-MYB128	MKKKSAWTKEEDAVLREQVRLHGPQNWSAISGALLGRNPKSCRLRWCQHLSPVVDTARPFTPQEDEKIVVFYRQYPNKWATIAGFLPGRTDNAIKNRWHSVLGKVY
Zea mays	GRMZM2G330475_T01	Zm2R-MYB129	ATELRRGPWTVDEDILLVNYIAAHGEGRWNSLARSAGLRRTGKSCRLRWLNYLRPDVRRGNITVEEQLLILELHSRWGNRWSKIAQHLPGRTDNEIKNYWRTRVQKHA
Zea mays	GRMZM2G395672_T01	Zm2R-MYB130	IRTLKRGAWTPEEDELLARAVAKDGEGRWRTLPRRAGLLRCGKSCRLRWMNYLRPDIKRGPIAADEEDLILRLHRLLGNRWSLIAGRLPGRTDNEVKNYWNSHLSKKL
Zea mays	GRMZM2G405094_T01	Zm2R-MYB131	KAHTNKGAWTKEEDQRLIAYIKAHGEGCWRSLPKAAGLLRCGKSCRLRWMNYLRPDLKRGNFTDDDDEVIIKLHALLGNKWSLIAGQLPGRTDNEIKNYWNTHIKRKL
Zea mays	GRMZM2G431156_T01	Zm2R-MYB132	KGHTNKGAWTKEEDERLVAYIRSHGEGCWRSLPSAAGLLRCGKSCRLRWMNYLRPDLKRGNFTDDEDELIIRLHALLGNKWSLIAGQLPGRTDNEIKNYWNTHIKRKL
Zea mays	GRMZM2G004090_T01	Zm2R-MYB133	APQLKKGPWSPDEDKLLKNYVEAHGEGKWNKVQRNSGLNRCGKSCRLRWTNHLRPDLKKGSFDNEEIDKILKFYLKWGPKWAKIASRLPGRTDNEIKNFWNTRIKKTE
Zea mays	GRMZM2G005066_T01	Zm2R-MYB134	KEGVKRGAWTSKEDDALAAYVKAHGEGKWREVPQKAGLRRCGKSCRLRWLNYLRPNIRRGNISYDEEDLMIIRLHRLLGNRWSLIAGRLPGRTDNEIKNYWNSTLGRRA
Zea mays	GRMZM2G022686_T01	Zm2R-MYB135	KVGLKRGRWTKEEDQILANYIAEHGEGSWRSLPKNAGLLRCGKSCRLRWINYLRADVKRGNISKEEEDVIIKLHATLGNRWSLIASHLPGRTDNEIKNYWNSHLSRQI
Zea mays	GRMZM2G044824_T01	Zm2R-MYB136	DAGVKKGPWTPEEDKLLVDYINGNGHGSWRRLPKHAGLNRCGKSCRLRWTNYLRPDIKRGRFTDDEEKLIIHLHSLLGNKWSAIATKLPGRTDNEIKNYWNTHLRKKL
Zea mays	GRMZM2G089686_T01	Zm2R-MYB137	QQGWRKGPWTALEDRLLTEYVQQHGEGCWNSVAKLTGLRRSGKSCRLRWVNYLRPDLKRGKITPDEETVILHLHAMLGNRWSAIARCLPGRTDNEIKNYWRTHFKKAR
Zea mays	GRMZM2G098179_T01	Zm2R-MYB138	KESVKKGPWTPEEDLVLVSYVQEHGPGNWRAVPANTGLLRCSKSCRLRWTNYLRPGIRRGGFSDQEDRLIVHLQALLGNRWAAIASYLPDRTDNDVKNYWNTHLKKKL
Zea mays	GRMZM2G134279_T01	Zm2R-MYB139	AGELRRGPWTVEEDLLLVNYVAAHGEGRWNSLARSAGLRRTGKSCRLRWLNYLRPDLRRGNITPQEQLLILELHSRWGNRWSKIAQHLPGRTDNEIKNYWRTRVQKHA
Zea mays	GRMZM2G167088_T01	Zm2R-MYB140	APQLKKGPWSPDEDKLLKNYVEAHGEGKWNKVQRNSGLNRCGKSCRLRWTNHLRPDLKKGPFDNEEIDKILKFYLKWGPKWAKIASRLPGRTDNEIKNFWNTRIKKTE
Zea mays	GRMZM2G416652_T01	Zm2R-MYB141	APQLKKGPWSPDEDKLLKNYVEAHGEGKWNKVQRNSGLNRCGKSCRLRWTNHLRPDLKKGPFDNEEIDKILKFYLKWGPKWAKIASRLPGRTDNEIKNFWNTRIKKTE
Zea mays	GRMZM5G803308_T01	Zm2R-MYB142	DCDRIRGPWSPEEDDALRRLVERHGARNWTAIGREIPGRSGKSCRLRWCNQLSPQVERRPFTAEEDAAIVRAHARLGNRWAAIARLLPGRTDNAVKNHWNCSLKRKL
Zea mays	AC197146.3_FGT002	Zm2R-MYB143	KAGVKKGPWTPEEDIVLVSYVQEHGPGNWRAVPVSTGLMRCSKSCRLRWTNYLRPGIRRGNFTPHEEGIIVHLQALLGNRWAAIASYLPQRTDNDIKNYWNTHLKKKL
Zea mays	AC206901.3_FGT005	Zm2R-MYB144	KMGLKRGPWTPEEDKILVAHIQSFGHSNWRALPKQAGLLRCGKSCRLRWINYLRPDIKRGNFSKEEEDAIITLHEQLGNRWSAIAARLPGRTDNEIKNVWHTHLKKRL
Zea mays	GRMZM2G001824_T01	Zm2R-MYB145	ADDLRRGPWTVDEDILLVNYIAAHGEGRWNSLARSAGLKRTGKSCRLRWLNYLRPNVRRGNITAEEQLLILDLHSRWGNRWSKIAQQLPGRTDNEIKNYWRTRVQKHA
Zea mays	GRMZM2G052606_T01	Zm2R-MYB146	AAKLRKGLWSPEEDERLVAYMLRSGQGSWSDVARNAGLQRCGKSCRLRWINYLRPDLKRGAFSPQEEELIVSLHAILGNRWSQIAARLPGRTDNEIKNFWNSTIKKRL
Zea mays	GRMZM2G081557_T01	Zm2R-MYB147	DAGVKKGPWTEEEDRALVDHIQRHGGHVSSWRNLPKAAGLNRCGKSCRLRWTNYLRPDIKRGNFTDDEERLIIGLHAQLGNRWSTIATHLDGRTDNEIKNYWNTHIRKKL
Zea mays	GRMZM2G097636_T01	Zm2R-MYB148	KVGLNKGSWTPEEDMRLIAYIQKYGHANWRALPKQAGLLRCGKSCRLRWINYLRPDLKRGNFTAEEEEAIIKLHGLLGNKWSKIASCLPGRTDNEIKNVWNTHLKKRV
Zea mays	GRMZM2G097638_T01	Zm2R-MYB149	KVGLNKGSWTPEEDMRLIAYIQKYGHANWRALPKQAGLLRCGKSCRLRWINYLRPDLKRGNFTAEEEEAIIKLHGLLGNKWSKIASCLPGRTDNEIKNVWNTHLKKRV
Zea mays	GRMZM2G127490_T01	Zm2R-MYB150	KQKLRKGLWSPEEDEKLMNHITKHGHGCWSSIPKLAGLQRCGKSCRLRWINYLRPDLKRGAFSQDEEDLIIELHAVLGNRWSQIAAQLPGRTDNEIKNLWNSCIKKKL
Zea mays	GRMZM2G150680_T01	Zm2R-MYB151	KPTAFKGPWTMEEDSILRDMVMHHGEGRWSLVAQSLPGRIGKQCRERWINHVDPNIKKNDIWTEEEDIVLIQAHKSFGSHWSTIAKFLPGWPENAIKNHWNSTKRSLG
Zea mays	GRMZM2G172487_T01	Zm2R-MYB152	TAAMKKGPWMPEEDLVLVSYIHEHGPTKWRHVPASTGLMRCSKSCRLRWTNYLRPGIRRGNFTPREERVIVHLHSLLGNRWAAIASHLPQRTDNDIKNYWNTHLKKKL
Zea mays	GRMZM2G172575_T01	Zm2R-MYB153	TTAIKKGPWMPEEDLVLVSYIHEHGPSKWRHVAASTGLMRCSKSCRLRWTNYLRPGIRRGHFTAREERVIVHLHSLLGNRWAAIASHLPQRTDNDIKNYWNTHLKKKK
Zea mays	GRMZM2G173633_T01	Zm2R-MYB154	KDSVKRGQWTPEEDNKLLSYITQYGTRNWRLIPKNAGLQRCGKSCRLRWTNYLRPDLKHGEFTDTEEQTIIKLHSVVGNRWSVIAAQLPGRTDNDVKNHWNTKLKKKL
Zea mays	GRMZM2G311059_T01	Zm2R-MYB155	GQAVRKGHWTAEEDAVLLRHVLVHGPRDWSSIRSKGFLPRTGKSCRLRWVNKLRPDLKTGCKFSAEEERVVLELQAQFGNKWARISTYLPGRTDNDVKNFWSTRQKRIA
Zea mays	GRMZM2G325907_T01	Zm2R-MYB156	QQKVKRGLWSPEEDEKLIRYITTHGYGCWSEVPEKAGLQRCGKSCRLRWINYLRPDIRRGRFTAEEEKLIISLHAIVGNRWAHIASHLPGRTDNEIKNYWNSWIKKKI
Zea mays	GRMZM2G425427_T01	Zm2R-MYB157	AADLRRGPWTAEEDILLVNYIAAHGEGRWNSLALSAGLKRTGKSCRLRWLNYLRPNVRRGNITAEEQLLILDLHSRWGNRWSKIAQHLPGRTDNEIKNYWRTRVQKHA
Zea mays	AC203535.4_FGT001	Zm2R-MYB158	MRIMIKGGVWKNTEDEILKAAVMKYGKNQWARISSLLVRKSAKQCKARWYEWLDPSIKKTEWTREEDEKLLHLAKLMPTQWRTIAPIVGRTPSQCLERYEKLLDAAC
Zea mays	GRMZM2G081919_T01	Zm3R-MYB1	PIRRTKGGWTLKEDETLRKAVEAFKGRNWKKIAEFFQDRTEVQCLHRWQKVLNPELIKGPWTQEEDEKIIDLVRKYGPTKWSIIAKSLPGRIGKQCRERWHNHLNPEIRKDAWTP<br>EEERALIDAHQVFGNKWAEIAKVLPGRTDNSIKNHWNSSLKKKL
Oryza sativa v7.0	LOC_Os01g03720.1	Os2R-MYB001	MMELRRGPWTLEEDNLLMNYIACHGEGRWNLLARCSGLKRTGKSCRLRWLNYLKPDIKRGNLTPEEQLLILELHSRWGNRWSRIAQHLPGRTDNEIKNYWRTRVQKQA
Oryza sativa v7.0	LOC_Os01g07450.1	Os2R-MYB002	KKKLRKGLWSPEEDERLATHIARFGVSCWSSVPDLAGLQRCGKSCRLRWMNYLRPDLKRGRFSHHEEELILALHDKLGNSWSQIAARLPGRSDNEIKNFWNARLRKKL
Oryza sativa v7.0	LOC_Os01g09590.1	Os2R-MYB003	REAVKRGPWSPEEDDALRDYINRHGTAGNWISLPNKAGLRRCGKSCRLRWLNYLRPDIRHGAFTDEEDAIITSLYSKLGSKWSTIAAQLERRTDNDVKNHWNTKLKRRL
Oryza sativa v7.0	LOC_Os01g16810.1	Os2R-MYB004	GKLCARGHWRPAEDAKLKDLVAQYGPQNWNLIAEKLDGRSGKSCRLRWFNQLDPRINRRAFTEEEEERLMAAHRAYGNKWALIARLFPGRTDNAVKNHWHVLMARRH
Oryza sativa v7.0	LOC_Os01g18240.1	Os2R-MYB005	KQKLRKGLWSPEEDEKLMNHITKHGHGCWSTVPKLAGLQRCGKSCRLRWINYLRPDLKRGAFSQEEEDLIVELHAVLGNRWSQIATRLPGRTDNEIKNLWNSCIKKKL
Oryza sativa v7.0	LOC_Os01g19330.1	Os2R-MYB006	EEDVRRGPWTVEEDAVLAGYVAANGEGRWNELARAAGLRRTGKSCRLRWLNYLRPDVRRGDFTPQEQLLILELHFRWGNRWSRIAQHMPGRTDNEIKNYWRTRVQKHA
Oryza sativa v7.0	LOC_Os01g19970.1	Os2R-MYB007	KVGLRRGRWTKEEDEKLARYIRENGEGAWRSMPKNAGLLRCGKSCRLRWINYLRADLKRGNISPQEEDIILNLHATLGNRWSLIAGHLPGRTDNEIKNYWNSHLSRKG
Oryza sativa v7.0	LOC_Os01g36460.1	Os2R-MYB008	KPKLRRGLWSPEEDEKLFNHISRYGVGCWSSVPKLAGLERCGKSCRLRWINYLRPDLKRGSFSQQEEELIISLHKILGNRWSQIAAQLPGRTDNEIKNFWNSCLKKKL
Oryza sativa v7.0	LOC_Os01g45090.1	Os2R-MYB009	EPAVRKGPWTLEEDLILVSYISQNGEGSWDNLARSAGLNRNGKSCRLRWLNYLRPGVRRGSITPEEDMVIRELHSRWGNRWSKIAKHLPGRTDNEIKNYWRTKIHRKP

Oryza sativa v7.0	LOC_Os01g49160.1	Os2R-MYB010	KASVKRGPWSPEEDELLRSYVRSHGTGGNWIALPQKAGLNRCGKSCRLRWLNYLRPDIKHGGYTDQEDRIICSLYNSIGSRWSIIASKLPGRTDNDVKNYWNTKLKKKA
Oryza sativa v7.0	LOC_Os01g50110.1	Os2R-MYB011	AAALKRGPWTPEEDEVLARFVAREGCDRWRTLPRRAGLLRCGKSCRLRWMNYLRPDIKRCPIADDEEDLILRLHRLLGNRWSLIAGRLPGRTDNEIKNYWNSHLSKKL
Oryza sativa v7.0	LOC_Os01g50720.1	Os2R-MYB012	QQKLRKGLWSPEEDEKLYNHIYRYGVGCWSSVPKLAGLQRCGKSCRLRWINYLRPDLKRGSFSQQEEDAIVGLHEILGNRWSQIASHLPGRTDNEIKNFWNSCLKKKL
Oryza sativa v7.0	LOC_Os01g51260.1	Os2R-MYB013	KQKVRRGLWSPEEDEKLVKYISTHGHGCWSSVPRLAGLQRCGKSCRLRWINYLRPDLKRGSFSQQEESLIIELHRVLGNRWAQIAKHLPGRTDNEVKNFWNSTIKKKL
Oryza sativa v7.0	LOC_Os01g52410.1	Os2R-MYB014	KASVKRGPWSEEEDAILRSFVERFGNAGNWIALPHKAGLKRCGKSCRLRWLNYLRPAIRHGGFTDEEDNLILSLYGEMGSKWSVIASKLPGRTDNDVKNYWNTKLKKRY
Oryza sativa v7.0	LOC_Os01g59660.1	Os2R-MYB015	GPPLKKGPWTSAEDAILVDYVKKHGEGNWNAVQKNTGLFRCGKSCRLRWANHLRPNLKKGAFTAEEERLIIQLHSKMGNKWARMAAHLPGRTDNEIKNYWNTRIKRCQ
Oryza sativa v7.0	LOC_Os01g63160.1	Os2R-MYB016	MEERVKGPWSSDEDTLLSNLVEKLGPRNWTLIARGIPGRSGKSCRLRWCNQLDPQVKRKPFTEEEDRIIMAAHAIHGNKWAAIAKLLVGRTDNAIKNHWNSTLRRRH
Oryza sativa v7.0	LOC_Os01g63680.1	Os2R-MYB017	NPGSFKGAWTRQEDEVLKQMVILHGDRKWATIAKSLPGRIGKQCRERWTNHLRPDIKKDVWTEEDDRMLIEAHKTYGNRWSVIARCLPGRSENAVKNHWNATKRSLK
Oryza sativa v7.0	LOC_Os01g65370.1	Os2R-MYB018	KAHTNKGAWTKEEDQRLIAYIKAHGEGCWRSLPKAAGLLRCGKSCRLRWMNYLRPDLKRGNFTDDDDELIIKLHALLGNKWSLIAGQLPGRTDNEIKNYWNTHIKRKL
Oryza sativa v7.0	LOC_Os01g74410.1	Os2R-MYB019	GGIMRKGPWTEQEDVQLVWFVRLLGERRWDFLAKVSGLQRSGKSCRLRWVNYLHPGLKRGRMSPEEERMVVQLHAKLGNRWSRIAKSIPGRTDNEIKNYWRTHLRKLK
Oryza sativa v7.0	LOC_Os01g74590.1	Os2R-MYB020	SKTRKKGSWRAEEDALLTRLVAQHGPHRWSIISGAIPGRSGKSCRLRWCNQLSPAVQHRPFTPQEDALLAAAHARHGNKWATIARLLPGRTDNSVKNHWNSNLRRCL
Oryza sativa v7.0	LOC_Os02g02370.1	Os2R-MYB021	KMNYRKGLWSPEEDQRLRDYIVKHGLGCWSAVPAKAGLQRNGKSCRLRWINYLRPGLKRGMFSQEEEDIVINLQAKLGNKWSQIAMHLPGRTDNEVKNYWNSYLKKRV
Oryza sativa v7.0	LOC_Os02g09480.1	Os2R-MYB022	ECDRIKGPWSPEEDEALRRLVERHGARNWTAIGRGIPGRSGKSCRLRWCNQLSPQVERRPFTAEEDAAILRAHARLGNRWAAIARLLPGRTDNAVKNHWNSSLKRKL
Oryza sativa v7.0	LOC_Os02g36890.1	Os2R-MYB023	EAGLKKGPWTPEEDQKLLAYIEQHGHGCWRSLPTKAGLRRCGKSCRLRWTNYLRPDIKRGKFTLQEEQTIIQLHALLGNRWSAIATHLPKRTDNEIKNYWNTHLKKRL
Oryza sativa v7.0	LOC_Os02g40530.1	Os2R-MYB024	EGGWRKGPWTAQEDKLLVEYVRQHGEGRWNSVAKITGLKRSGKSCRLRWVNYLRPDLKRGKITPQEESVILELHALWGNRWSTIARSLPGRTDNEIKNYWRTHFKKGK
Oryza sativa v7.0	LOC_Os02g41510.1	Os2R-MYB025	KMGLKRGPWTAEEDRILVAHIERHGHSNWRALPRQAGLLRCGKSCRLRWINYLRPDIKRGNFTREEEDAIIHLHDLLGNRWSAIAARLPGRTDNEIKNVWHTHLKKRL
Oryza sativa v7.0	LOC_Os02g42850.2	Os2R-MYB026	RPNYRKGLWSPEEDQKLRDYILRHGHGCWSALPANAGLQRNGKSCRLRWINYLRPGLKHGVFSPEEEETVMSLHAALGNKWSRIARHLPGRTDNEVKNYWNSYLKKRV
Oryza sativa v7.0	LOC_Os02g42870.1	Os2R-MYB027	KKGLKKGPWTPEEDKLLVDYIQANGHGSWRLLPKLAGLNRCGKSCRLRWTNYLRPDIKRGPFTAEEQKSIVQLHGIVGNKWSMIAAQLPGRTDNEIKNYWNTHLKKQL
Oryza sativa v7.0	LOC_Os02g46780.1	Os2R-MYB028	KVGLNRGSWTPQEDMRLIAYIQKHGHANWRALPKQAGLLRCGKSCRLRWINYLRPDLKRGNFTADEEDTIIKLHGLLGNKWSKIASCLPGRTDNEIKNVWNTHLKKRV
Oryza sativa v7.0	LOC_Os02g49986.1	Os2R-MYB029	KVGLKKGPWTADEDQKLVTFLLSNGHCCWRLVPKLAGLLRCGKSCRLRWTNYLRPDLKRGLLSEEEEKLVIDLHEQLGNRWSKIAARLPGRTDNEIKNHWNTHIKKKL
Oryza sativa v7.0	LOC_Os02g51799.1	Os2R-MYB030	ENGLKKGPWTTEEDEKLMEYIQKNGHGSWRALPKLAGLNRCGKSCRLRWTNYLRPDIKRGKFTSAEKDTILQLHAVLGNKWSAIAKHLPGRTDNEIKNYWNTHLKKDL
Oryza sativa v7.0	LOC_Os02g54520.1	Os2R-MYB031	KASVKKGPWSPEEDAKLKAYIEENGTGGNWIALPQKIGLKRCGKSCRLRWLNYLRPNIKHGDFTEEEEHIICSLYISIGSRWSIIAAQLPGRTDNDIKNYWNTKLKKKL
Oryza sativa v7.0	LOC_Os03g04900.1	Os2R-MYB032	QQGWRKGPWTALEDRLLTEYVQQHGEGSWNSVAKLTGLRRSGKSCRLRWVNYLRPDLKRGKITPDEETVILQLHAMLGNRWSAIARCLPGRTDNEIKNYWRTHFKKAR
Oryza sativa v7.0	LOC_Os03g13310.1	Os2R-MYB033	QEAGKKVGWSKEEDKLLRELVRKQGGKDW***GVIATAFPGRTDKSCRLRWRQHLDPSVDVAL***PFSAGEDRKIVELHRVHGNRWATIAAFLPGRSDNAIKNRWNTHLRKRH
Oryza sativa v7.0	LOC_Os03g18480.1	Os2R-MYB034	KANVKKGPWTAEEDAKLLAYTSTHGTGNWTSVPQRAGLKRCGKSCRLRYTNYLRPNLKHENFTQEEEELIVTLHAMLGSRWSLIANQLPGRTDNDVKNYWNTKLSKKL
Oryza sativa v7.0	LOC_Os03g19120.1	Os2R-MYB035	KVGLKRGRWTTEEDEKLAGYIAKHGEGSWRSLPKNAGLLRCGKSCRLRWINYLRAGVKRGNISNQEEDVIIKLHATLGNRKSYVVKRMDYVCLGARDYCFQQNTHVRWSLIAS<br>HLPGRTDNEIKNYWNSHLSRQI
Oryza sativa v7.0	LOC_Os03g20090.1	Os2R-MYB036	GGDLRRGPWTVEEDLLLVNYIAAHGEGRWNSLARSAGLKRTGKSCRLRWLNYLRPDLRRGNITPQEQLLILELHSRWGNRWSKIAQHLPGRTDNEIKNYWRTRVQKHA
Oryza sativa v7.0	LOC_Os03g25550.1	Os2R-MYB037	KKKLRRGLWSPEEDEKLMNHIAKYGHGCWSSVPKLAGLERCGKSCRLRWINYLRPDLKRGTFSQEEEDLIIQLHSMLGNKYVLYSSSTHICQSLSRSVPSLTMAAATATALARWS<br>QIAAQLPGRTDNEVKNFWNSYIKKKL
Oryza sativa v7.0	LOC_Os03g26130.1	Os2R-MYB038	KEGVKKGPWTPEEDMVLASYVQEHGPGNWRAVPPRTGLLRCSKSCRLRWTNYLRPGIRRGGFSHHEERLILHLQALLGNRWAAIASYLPHRTDNDVKNFWNTHLKKKL
Oryza sativa v7.0	LOC_Os03g27090.1	Os2R-MYB039	ESSVKKGPWTREEDEKLVAYVDQHGGHGSWRSLPKRAGLNRCCKSCRLRWINYLRPDIKRGNFTPEEEQAIITLHSVLGNKWSTIATRLPGRTDNEIKNYWNTRLKKRL
Oryza sativa v7.0	LOC_Os03g29614.1	Os2R-MYB040	KEGLNRGAWTAMEDDILVSYIAKHGEGKWGALPKRAGLKRCGKSCRLRWLNYLRPGIKRGNISGDEEELILRLHTLLGNRWSLIAGRLPGRTDNEIKNYWNSTLSKRV
Oryza sativa v7.0	LOC_Os03g38210.1	Os2R-MYB041	PVVLKKGPWTTAEDAVLVQHVRQHGEGNWNAVQRMTGLLRCGKSCRLRWTNHLRPNLKKGSFSPDEELLIAQLHAQLGNKWARMASHLPGRTDNEIKNYWNTRTKRRQ
Oryza sativa v7.0	LOC_Os03g51110.1	Os2R-MYB042	MCTRGHWRPSEDEKLKELVARYGPHNWNAIAEKLQGRSGKSCRLRWFNQLDPRINRSPFTEEEEELLLASHRAHGNRWAVIARLFPGRTDNAVKNHWHVIMARRC
Oryza sativa v7.0	LOC_Os03g56090.1	Os2R-MYB043	KASVKKGPWSPEEDAKLKSYIEQNGTGGNWIALPQKIGLKRCGKSCRLRWLNYLRPNIKHGGFSEEEDRIILSLYISIGSRWSIIAAQLPGRTDNDIKNYWNTRLKKKL
Oryza sativa v7.0	LOC_Os04g28090.1	Os2R-MYB044	RIMIKGGVWKNTEDEILKAAVMKYGKNQWARISSLLVRKSAKQCKARWYEWLDPSIKKTEWTREEDEKLLHLAKLMPTQWRTIAPIVGRTPSQCLERYEKLLDAAC
Oryza sativa v7.0	LOC_Os04g38740.1	Os2R-MYB045	KEGLKKGPWTPEEDQKLLAYIEQHGHGCWRSLPSKAGLQRCGKSCRLRWTNYLRPDIKRGKFSLQEEQTIIQLHALLGNRWSAIATHLPKRTDNEIKNYWNTHLKKRL
Oryza sativa v7.0	LOC_Os04g39470.1	Os2R-MYB046	KDNVKRGQWTPEEDNKLLSYITQYGTRNWRLIPKNAGLQRCGKSCRLRWTNYLRPDLKHGEFTDAEEQTIIKLHSVVGNRWSVIAAQLPGRTDNDVKNHWNTKLKKKL
Oryza sativa v7.0	LOC_Os04g42950.1	Os2R-MYB047	GGGWRKGPWTSQEDALLVEHVRQHGEGRWNSVSKLTGLKRSGKSCRLRWVNYLRPDLKRGKITPQEESIIVQLHALWGNRWSTIARSLPGRTDNEIKNYWRTHFKKGK
Oryza sativa v7.0	LOC_Os04g43680.1	Os2R-MYB048	KMGLKKGPWTPEEDKVLVAHIQRHGHGNWRALPKQAGLLRCGKSCRLRWINYLRPDIKRGNFSKEEEDTIIHLHELLGNRWSAIAARLPGRTDNEIKNVWHTHLKKRL
Oryza sativa v7.0	LOC_Os04g45020.1	Os2R-MYB049	KVHYRKGLWSPEEDQKLRDFILRYGHGCWSAVPVKAGLQRNGKSCRLRWINYLRPGLKHGMFSREEEETVMNLHATMGNKWSQIARHLPGRTDNEVKNYWNSYLKKRV
Oryza sativa v7.0	LOC_Os04g45060.1	Os2R-MYB050	SKVLKKGPWTPDEDKLLVDYVQANGSGNWRLLPKLAGLNRCGKSCRLRWTNYLRPDIKRGPFTPEEHKSILQLHAIVGNKWSMIAAQLPGRTDNEIKNYWNTNVKKQL
Oryza sativa v7.0	LOC_Os04g46384.1	Os2R-MYB051	GEALRKGPWMAEEDEVLLEHVRTHGPMDWSSIRSKGLLPRTGKSCRLRWVNKLRPNLKSGCKFTAEEERVVIELQAQFGNKWARIATYLQGRTDNDVKNFWSTRQKRLA
Oryza sativa v7.0	LOC_Os04g50680.1	Os2R-MYB052	DAGVKKGPWTEEEDRALVEHIKKQGGHVGSWRGLPRAAGLNRCGKSCRLRWTNYLRPDIRRGNFSDDEERLIIRLHAALGNKWSTIATHLDGRTDNEIKNYWNTHIKKKL
Oryza sativa v7.0	LOC_Os04g50770.1	Os2R-MYB053	KVGLNKGSWTPEEDMRLVAYIQKYGHANWRALPKQAGLLRCGKSCRLRWINYLRPDLKRGNFTAEEEETIIKLHGLLGNKWSKIASCLPGRTDNEIKNVWNTHLKKRV

Oryza sativa v7.0	LOC_Os05g04210.1	Os2R-MYB054	EGDLRRGPWTVEEDMLLVDYIANHGEGRWNSLARCAGLRRTGKSCRLRWLNYLRPDVRRGNITADEQLLILDLHSRWGNRWSKIAQYLPGRTDNEIKNYWRTRVQKHA
Oryza sativa v7.0	LOC_Os05g04820.1	Os2R-MYB055	KQKLRKGLWSPEEDEKLMNHITKHGHGCWSSVPKLAGLQRCGKSCRLRWINYLRPDLKRGAFSQEEEDLIIELHAVLGNRWSQIAAQLPGRTDNEIKNLWNSCIKKKL
Oryza sativa v7.0	LOC_Os05g28320.1	Os2R-MYB056	AGGKIKGSWSPEEDEQLRGAVARHGPRNWTAISEEVPGRSGKSCRLRWCNQLSPGVHRRPFTPDEDALIVAAHAKYGNKWATIARLLDGRTDNSVKNHWNSSLRRNR
Oryza sativa v7.0	LOC_Os05g35500.1	Os2R-MYB057	KAHTNKGAWTKEEDQRLIAYIRAHGEGCWRSLPKAAGLLRCGKSCRLRWMNYLRPDLKRGNFTDDEDELIIRLHSLLGNKWSLIAGQLPGRTDNEIKNYWNTHIKRKL
Oryza sativa v7.0	LOC_Os05g41166.1	Os2R-MYB058	LKKGPWTSWEDSILEKYIKKHGERNWKLVQKNTGLLRCGKSCRLRWMNHLRPNLKKGAFSKEEENKIINLHRKMGNKWSRMAADLPGRTDNEIKNYWNTRIKKCK
Oryza sativa v7.0	LOC_Os05g46610.1	Os2R-MYB059	QAKLRKGLWSPEEDDKLYNHIIRHGVGCWSSVPKLAGLQRCGKSCRLRWINYLRPDLKRGSFSQQEEDLIVALHEILGNRWSQIASHLPGRTDNEIKNFWNSCLKKKL
Oryza sativa v7.0	LOC_Os05g48010.1	Os2R-MYB060	KASVKRGPWSPEEDEQLRSYVQSHGIGGNWIALPQKAGLNRCGKSCRLRWLNYLRPDIKHGGYTEQEDHIICSLYNSIGSRWSIIASKLPGRTDNDVKNYWNTKLKKKA
Oryza sativa v7.0	LOC_Os05g49310.1	Os2R-MYB061	EAAVRKGPWTMEEDLSLVNYIAANGEGAWNTLARAAGLNRTGKSCRLRWLNYLRPDVRRGNITPEEHTLIVELQARWGNRWSKIARYLPGRTDNEVKNFWRTKIQKKH
Oryza sativa v7.0	LOC_Os06g02250.1	Os2R-MYB062	DADVKKGPWTPEEDKLLVEYIGKNGHGSWRRLPKLAGLNRCGKSCRLRWTNYLRPDIKRGGFSDDEERLIIHLHATLGNKWSSIATKLKGRTDNEIKNYWNTHLRKKL
Oryza sativa v7.0	LOC_Os06g06740.1	Os2R-MYB063	KANVVKGQWTLEEDRKLVKLVEQFGLRKWSHIAQILPGRVGKQCRERWHNHLRPNIKKDTWSEEEDIVLIQTHKEVGNKWAEIAKHLPGRTENSIKNHWNATKRRQF
Oryza sativa v7.0	LOC_Os06g10350.1	Os2R-MYB064	KEGMKRGAWTSKEDDVLASYIKSHGEGKWREVPQRAGLRRCGKSCRLRWLNYLRPNIKRGNIDDDEELIVRLHTLLGNRWSLIAGRLPGRTDNEIKNYWNSTLSRKI
Oryza sativa v7.0	LOC_Os06g11780.1	Os2R-MYB065	ENGLKKGPWTPEEDEKLMSYIQKHGHASWRVLPELAGLNRCGKSCRLRWTNYLKPDIKRGNFSREEEQTILQLHSILGNKWSAIAKHLPGRTDNEIKNFWNTHLRKKL
Oryza sativa v7.0	LOC_Os06g14670.1	Os2R-MYB066	KVGLKKGPWTAEEDQKLVAFLLTHGHCCWRVVPKLAGLLRCGKSCRLRWTNYLRPDLKRGLLSDDEERLVIDLHAQLGNRWSKIAARLPGRTDNEIKNHWNTHIKKKL
Oryza sativa v7.0	LOC_Os06g40330.1	Os2R-MYB067	RIPLKKGPWTPDEDKRLREYVEAHGEGNWNRVQRNAGLNRCGKSCRLRWANHLKPDLKKGPFSKEEEEMIIKLHLWLGNKWAKMANSLPGRTDNEIKNFWNTRCKRIQ
Oryza sativa v7.0	LOC_Os06g43090.1	Os2R-MYB068	DCDRIRGPWSPEEDEALRRLVERHGARNWTAIGREIPGRSGKSCRLRWCNQLSPQVERRPFTAEEDATILRAHARLGNRWAAIARLLQGRTDNAVKNHWNCSLKRKL
Oryza sativa v7.0	LOC_Os06g46560.1	Os2R-MYB069	GGGLKKGPWTQAEDKLLVDHVRRHGEGNWNAVRRETGLQRCGKSCRLRWANHLRPDLRKGPFSPDEERLILRLHGLLGNKWARISSYLHGRTDNEIKNYWNTRLKRRV
Oryza sativa v7.0	LOC_Os07g12130.1	Os2R-MYB070	NPGLVKGGWTREEDEVLRQMVRHHGDRKWAEIAKSLPGRVGKQCRERWTNHLHPDIKKDIWTEEEDRMLIEAHQTYGNSWSAIAKQLPGRSENTIKNHWNATKRSLN
Oryza sativa v7.0	LOC_Os07g14110.1	Os2R-MYB071	NSGLIKGGWTREEDEVLRQMVRHHGDRKWAEIAKSLPGRIGKQCRERWTNHLHPDIKKGIWTEEEDRKLIKAHQTYGNRWSAIARSLPGRSENTVKNRWNATKRSLN
Oryza sativa v7.0	LOC_Os07g31470.1	Os2R-MYB072	KQKVKRGLWSPEEDEKLVRYISEHGHSCWSSVPKHAGLQRCGKSCRLRWINYLRPDLKRGTFSEQEERTIIDVHRILGNRWAQIAKHLPGRTDNEVKNFWNSCIKKKL
Oryza sativa v7.0	LOC_Os07g37210.1	Os2R-MYB073	KSGLKKGPWTPEEDEKLIAYIKEHGQGNWRTLPKNAGLSRCGKSCRLRWTNYLRPDIKRGRFSFEEEEAIIQLHSILGNKWSAIAARLPGRTDNEIKNYWNTHIRKRL
Oryza sativa v7.0	LOC_Os07g43420.1	Os2R-MYB074	KKDRHIVSWSTEEDDVLRTQIALHGTDNWTIIAAQFKDKTARQCRRRWYNYLNSECKKGGWSREEDLLLCEAQKVLGNKWTEIAKVVSGRTDNAVKNRFSTLCKRRA
Oryza sativa v7.0	LOC_Os07g43580.1	Os2R-MYB075	KDGVKKGPWTPEEDLVLVSYVQEHGPGNWRAVPTRTGLMRCSKSCRLRWTNYLRPGIKRGNFTDQEEKLIVHLQALLGNRWAAIASYLPERTDNDIKNYWNTHLKRKL
Oryza sativa v7.0	LOC_Os07g44090.3	Os2R-MYB076	RKKLRRGLWSPEEDEKLMNHIAKYGHGCWSSVPKLAGLERCGKSCRLRWINYLRPDLKRGAFSQEEEDLIIHLHSMLGNKWSQIAAQLPGRTDNEVKNFWNSYIKKKL
Oryza sativa v7.0	LOC_Os07g48870.1	Os2R-MYB077	GDELRRGPWTVEEDLLLVNYIAAHGEGRWNALARCAGLKRTGKSCRLRWLNYLRPDVRRGNMTAEEQLLILELHGRWGNRWSKIAQHLPGRTDNEIKNYWRTRVQKHA
Oryza sativa v7.0	LOC_Os08g05520.1	Os2R-MYB078	QQKVKRGLWSPEEDEKLIRYITTHGYGCWSEVPEKAGLQRCGKSCRLRWINYLRPDIRRGRFTAEEEKLIISLHAIVGNRWAHIASHLPGRTDNEIKNYWNSWIKKKI
Oryza sativa v7.0	LOC_Os08g15020.1	Os2R-MYB079	KATVKKGPWSPEEDAMLKNYIEEHGTGGNWIALPHKIGLKRCGKSCRLRWLNYLRPNIKHGDFTPEEDSIICSLYISIGSRWSIIAAQLPGRTDNDVKNYWNTKLKKRL
Oryza sativa v7.0	LOC_Os08g33150.1	Os2R-MYB080	KVGLKKGPWTAEEDQKLITFLLTNGQCCWRAVPKLAGLLRCGKSCRLRWTNYLRPDLKRGLLSDAEEKIVVDLHAQLGNRWSKIASHLPGRTDNEIKNHWNTHIKKKL
Oryza sativa v7.0	LOC_Os08g33660.1	Os2R-MYB081	KIGLKKGPWTPEEDQKLLAYIEEHGHGSWRALPSKAGLQRCGKSCRLRWTNYLRPDIKRGKFSLQEEQTIIQLHALLGNRWSAIATHLPKRTDNEIKNYWNTHLKKRL
Oryza sativa v7.0	LOC_Os08g33800.1	Os2R-MYB082	SRLAARGHWRPAEDAKLRELVALYGPQNWNLIADKLDGRSGKSCRLRWFNQLDPRISKRPFSDEEEERLMAAHRFYGNKWAMIARLFPGRTDNAVKNHWHVIMARKY
Oryza sativa v7.0	LOC_Os08g33940.1	Os2R-MYB083	KAEVKKGPWTPEEDLMLVAYVQEHGPGNWRAVPTNTGLMRCSKSCRLRWTNYLRPGIKRGNFTDQEEKLIVHLQALLGNRWAAIASYLPERTDNDIKNYWNTHLKKKL
Oryza sativa v7.0	LOC_Os08g34960.1	Os2R-MYB084	SGGGRKTPWTQEEDEALRRAVREHRRQNWAEIALALPRRGP***KSCRLRWCQHLSPELDSRVFTAEEDAIILAQ***QRVHGNKWATIARCLPGRYDNAVKNRWNSALRKLL
Oryza sativa v7.0	LOC_Os08g37970.1	Os2R-MYB085	ESGLKKGPWTPEEDEKLLHYIQKNGHGSWRTLPRLAGLNRCGKSCRLRWTNYLRPDIKRGKFSQEEEQTILHLHSILGNKWSAIATHLPGRTDNEIKNFWNTHLKKRL
Oryza sativa v7.0	LOC_Os08g43550.1	Os2R-MYB086	KEHTNKGAWTKEEDERLVAYIRAHGEGCWRSLPKAAGLLRCGKSCRLRWINYLRPDLKRGNFTADEDDLIIKLHSLLGNKWSLIAARLPGRTDNEIKNYWNTHIRRKL
Oryza sativa v7.0	LOC_Os09g01960.1	Os2R-MYB087	DVDRIKGPWSPEEDEALQRLVGRHGARNWSLISKSIPGRSGKSCRLRWCNQLSPQVEHRPFTPEEDDTILRAHARFGNKWATIARLLAGRTDNAIKNHWNSTLKRKH
Oryza sativa v7.0	LOC_Os09g23620.1	Os2R-MYB088	KVGLKKGPWTAEEDQKLVSFLLGNGQCCWRAVPKLAGLLRCGKSCRLRWTNYLRPDLKRGLLSETEEKTVIDLHEQLGNRWSKIASHLPGRTDNEIKNHWNTHIKKKL
Oryza sativa v7.0	LOC_Os09g24800.1	Os2R-MYB089	KVGVKKGPWTPEEDLMLVSYIQEHGAGNWRAVPTNTGLMRCSKSCRLRWTNYLRPGIKRGNFTEQEEKLIVHLQALLGNRWAAIASYLPERTDNDIKNYWNTHLKKKL
Oryza sativa v7.0	LOC_Os09g26170.1	Os2R-MYB090	KATVKKGPWAPEEDAALKAYVDAHGTGGNWIALPHKIGLNRCGKSCRLRWLNYLRPNIRHGGFTEDEDRLICSLYIAIGSRWATIAAQLPGRTDNDIKNYWNSKLKRRL
Oryza sativa v7.0	LOC_Os09g36250.1	Os2R-MYB091	KLGVKRGPWTAEEDKKLMSFILTNGHCCWRAVPKLAGLLRCGKSCRLRWTNYLRPDLKRGLLTDAEEQLVIDLHAKLGNRWSKIAAKLPGRTDNEIKNHWNTHIKKKL
Oryza sativa v7.0	LOC_Os09g36730.1	Os2R-MYB092	KAHTNKGAWTKEEDDRLIAYIKAHGEGCWRSLPKAAGLLRCGKSCRLRWINYLRPDLKRGNFTEEEDELIIKLHSLLGNKWSLIAGRLPGRTDNEIKNYWNTHIRRKL
Oryza sativa v7.0	LOC_Os10g33810.1	Os2R-MYB093	KEGLRRGAWSPEEDDRLVAYIRRHGHPNWRALPKQAGLLRCGKSCRLRWINYLRPDIKRGNFTADEEDLIVRLHNSLGNRWSAIAAQMPGRTDNEIKNVWHTHLKKRL
Oryza sativa v7.0	LOC_Os10g35660.1	Os2R-MYB094	MKKGKWSKEEDDLIKNHMEKYGIGRSWQALSDALGLQRCGRSCRSRWLNYLRPGLKHGDFSPAEERIICKMYSKKGSSWSAIAAQLPGRTDLAVKNYWNSTLKKRF
Oryza sativa v7.0	LOC_Os11g03440.1	Os2R-MYB095	KEGIKKGPWTPEEDIILVSYIQEHGPGNWRSVPINTGLMRCSKSCRLRWTNYLRPGIKRGNFTAHEEGIIVHLQSLLGNRWAAIASYLPQRTDNDIKNYWNTHLKKKL
Oryza sativa v7.0	LOC_Os11g10130.1	Os2R-MYB096	SSACPRGHWRPGEDEKLRQLVEKYGPQNWNSIAEKLEGRSGKSCRLRWFNQLDPRINKRPFTEEEEERLLAAHRHHGNKWALIARHFPGRTDNAVKNHWHVVRARRS
Oryza sativa v7.0	LOC_Os11g35390.1	Os2R-MYB097	GVGVKKGPWTPEEDIILVSYIQQHGPGNWRSVPENTGLMRCSKSCRLRWTNYLRPGIKRGNFTPHEEGIIIHLQALLGNKWAAIASYLPQRTDNDIKNYWNTHLKKKV
Oryza sativa v7.0	LOC_Os11g45740.1	Os2R-MYB098	SAELRRGPWTVDEDLTLINYISDHGEGRWNALARAAGLKRTGKSCRLRWLNYLRPDVKRGNFTAEEQLLILDLHSRWGNRWSKIAQHLPGRTDNEIKNYWRTRVQKHA

	Oryza sativa v7.0	LOC_Os11g47460.1	Os2R-MYB099	REEMRKGPWTEQEDLQLVCTVRLFGDRRWDFVAKVSGLNRTGKSCRLRWVNYLHPGLKHGRMSPKEEHLIIELHARWGNRWSRIARRLPGRTDNEIKNYWRTHMRKKA
	Oryza sativa v7.0	LOC_Os12g03150.1	Os2R-MYB100	KEGIKKGPWTPEEDIILVSYIQEHGPGNWRSVPINTGLMRCSKSCRLRWTNYLRPGIKRGNFTAHEEGIIVHLQSLLGNRWAAIASYLPQRTDNDIKNYWNTHLKKKL
	Oryza sativa v7.0	LOC_Os12g07610.1	Os2R-MYB101	KADAVKGHWTVEEDRKLVKLVEQFGLKKWSLIGGMLPGRVGKQCRERWFNHLRPNIKKDTWSEEEDMVLIQIHKEVGNRWAEIAKCLPGRTENSIKNHWNATKRRQF
	Oryza sativa v7.0	LOC_Os12g07640.1	Os2R-MYB102	KAHTNKGAWTKEEDQRLIAHINQHGEGCWRSLPKAAGLLRCGKSCRLRWINYLRPDLKRGNFTDEEDELIIKLHELLGNKWSLIAGRLPGRTDNEIKNYWNTHIKRKL
	Oryza sativa v7.0	LOC_Os12g33070.1	Os2R-MYB103	GGKVRKGLWSPEEDEKLVAYMLRSGQGSWSDVARNAGLQRCGKSCRLRWINYLRPDLKRGAFSPQEEDLIVNLHAILGNRWSQIAARLPGRTDNEIKNFWNSTIKKRL
	Oryza sativa v7.0	LOC_Os12g37690.1	Os2R-MYB104	QLELRRGPWTVDEDLTLVNYIADHGEGRWNSLARAAGLKRTGKSCRLRWLNYLRPDVKRGNFTADEQLLILDLHSRWGNRWSKIAQHLPGRTDNEIKNYWRTRVQKHA
	Oryza sativa v7.0	LOC_Os12g37970.1	Os2R-MYB105	REEIRKGPWTEQEDLQLVCTVRLFGERRWDFIAKVSGLNRTGKSCRLRWVNYLHPGLKRGRMSPHEERLILELHARWGNRWSRIARRLPGRTDNEIKNYWRTHMRKKA
	Oryza sativa v7.0	LOC_Os12g38400.2	Os2R-MYB106	MRERQRWRPEEDAILLAYVRQYGPREWSLVSQRMNRPLHRDAKSCLERWKNYLRPGIKKGSLTDDEQRLVIRLQAKHGNKWKKIAAEVPGRTAKRLGKWWEVFKEKQQ
	Oryza sativa v7.0	LOC_Os01g62410.1	Os3R-MYB1	PIRRAKGGWTPEEDETLRKAVEAYKGRNWKKIAECFPYRTEVQCLHRWQKVLNPELIKGPWTQEEDDQIIDLVKKYGPTKWSVIAKALPGRIGKQCRERWHNHLNPEIRKDAWT TEEEQALINAHRIYGNKWAEIAKVLPGRTDNSIKNHWNSSLRKKQ
	Oryza sativa v7.0	LOC_Os05g38460.1	Os3R-MYB2	PVRRAKGGWTPEEDEKLRKAVDIYNGKNWKKIEVQCLHRWQKVLDPELIKGPWTQEEDDVIINMVKKHGPKKWSVIARSLNGRIGKQCRERWHNHLDPQIRKEAWTVEEERVL ARAHCMYGNKWAEIAKLLPGRTDNSIKNHWNSSLRKKI
Eudicots	*Aquilegia coerulea v1.1*	Aquca_034_00352.1	Ac2R-MYB01	RIMIKGGVWKNTEDEILKAAVMKYGKNQWARISSLLVRKSAKQCKARWYEWLDPSIKKTEWTREEDEKLLHLAKLMPTQWRTIAPIVGRTPSQCLERYEKLLDAAC
	Aquilegia coerulea v1.1	Aquca_014_00928.2	Ac2R-MYB02	GTVLKKGPWTSTEDAILVDYVKKHGEGNWNAVQKHSGLFRCGKSCRLRWANHLRPNLKKGAFTAEEEHKIIELHAKMGNKWARMAAHLPGRTDNEIKNYWNTRIKRRQ
	Aquilegia coerulea v1.1	Aquca_002_00543.1	Ac2R-MYB03	QKERHIVTWSQQEDDILREQISIHGTENWAIIAAQFKDKTTRQCRRRWYTYLNSDFKKGGWSQEEDLILCEAQKIYGNRWTEIAKVVSGRTDNAVKNRFSTLCKKRA
	Aquilegia coerulea v1.1	Aquca_002_00639.1	Ac2R-MYB04	SKLCARGHWRPAEDTKLKELVALYGPQNWNLIAEKLEGRSGKSCRLRWFNQLDPRINRKAFNEEEEERLMAAHRLYGNKWAMIARLFPGRTDNSVKNHWHVIMARKY
	Aquilegia coerulea v1.1	Aquca_009_00809.1	Ac2R-MYB05	SDDRVKGPWSPEEDNVLSRLVAKFGPRNWSLIARGIPGRSGKSCRLRWCNQLDPGVKRKPFTEEEDRIIVAAHAIHGNKWAVLARLLPGRTDNAIKNHWNSTLRRRR
	Aquilegia coerulea v1.1	Aquca_025_00152.1	Ac2R-MYB06	KSNVKRGLWTAEEDAKILAYVSDHGPGNWTSVPKKAGLRRCGKSCRLRYTNYLRPNLKHDNFTPQEEELIINLHAAIGSRWSLIAQQLPGRTDNDVKNHWNTKLKKRL
	Aquilegia coerulea v1.1	Aquca_002_00671.1	Ac2R-MYB07	MRHGHGSWRALPAKAGLQRCGKSCRLRWTNYLRPDIKRGKFSLQEEQTIIQLHALLGNRWSAIATHLPKRTDNEIKNYWNTHLKKRL
	Aquilegia coerulea v1.1	Aquca_028_00141.1	Ac2R-MYB08	KVGLNKGLWSAEEDEILRNYIEENGEGSWRSLPKNAGLNRCGKSCRLRWVNYLRGGLMRGNITSEEEDIIIKMHNKIGNRWSVIARHLPGRTDNEIKNHWNSYLSRRI
	Aquilegia coerulea v1.1	Aquca_011_00538.1	Ac2R-MYB09	KQKLRKGLWSPEEDEKLVNYISRNGIGCWSSVPKQAGLQRCGKSCRLRWINYLRPDLKRGMFSQQEENTIINLHEILGNRWAQISAHLPGRTDNEIKNFWNSCLKKKL
	Aquilegia coerulea v1.1	Aquca_027_00369.1	Ac2R-MYB10	MKERQRWRAEEDALLRAYVKQYGPREWNLVSQRMNTPLDRDAKSCLERWKNYLKPGIKKGSLTEEEQRLVIRLQAKHGNKWKKIAAEVPGRTAKRLGKWWEVFKEKQQ
	Aquilegia coerulea v1.1	Aquca_001_00247.1	Ac2R-MYB11	KANVKKGPWSPEEDAKLKEYIEQHGTGGNWIALPQKIGLKRCGKSCRLRWLNYLRPNLKHGGFSEEEDHMICSLYMSIGSRWSIIAAQLPGRTDNDIKNYWNTKLKKKL
	Aquilegia coerulea v1.1	Aquca_005_00315.1	Ac2R-MYB12	KDNVKRGQWTPEEDNKLSSYIAQHGTRNWRLIPKNAGLQRCGKSCRLRWTNYLRPDLKHGEFQESEEQTIVKLHSVVGNRWSLIAAQLPGRTDNDVKNHWNTKLKKKL
	Aquilegia coerulea v1.1	Aquca_002_00594.1	Ac2R-MYB13	KVGVKKGPWTPEEDIILVSYIQEHGPGNWRAVPTNTGLLRCSKSCRLRWTNYLRPGIKRGNFTDHEEKMIIHLQALLGNRWAAIASYLPQRTDNDIKNYWNTHLKKKL
	Aquilegia coerulea v1.1	Aquca_010_00195.1	Ac2R-MYB14	ENGLKKGPWTPEEDHKLVQYIQKHGHGSWRALPKLAGLNRCGKSCRLRWTNYLRPDIKRGKFSQEEEQTILNLHSFLGNKWSAIATHLPGRTDNEIKNFWNTHLKKKL
	Aquilegia coerulea v1.1	Aquca_030_00066.1	Ac2R-MYB15	KANVKKGPWSPEEDTRLKEYIEKFGTGGNWIALPQKAGLKRCGKSCRLRWLNYLRPNIKHGQFSDDEDKVICSLFASIGSRWSIIAAQLPGRTDNDIKNYWNTKLKKKF
	Aquilegia coerulea v1.1	Aquca_003_00440.1	Ac2R-MYB16	SLDLRRGPWTVEEDLTLINYISNHGEGRWNSLARCAGLKRTGKSCRLRWLNYLRPDVRRGNITLEEQLLILELHSRWGNRWSKIAQHLPGRTDNEIKNYWRTRVQKHA
	Aquilegia coerulea v1.1	Aquca_013_00356.1	Ac2R-MYB17	KLGLKKGPWAPEEDELLTDYIKKNGHGSWRSLPKLAGLLRCGKSCRLRWTNYLRPDIKRGPFTAEEEKTIIQLHGILGNRWASIASQLPGRTDNEIKNFWNTHLKKRL
	Aquilegia coerulea v1.1	Aquca_009_00496.1	Ac2R-MYB18	KEGLKRGPWTPEEDEVLSDYIRREGEGRWRTLPKRAGLLRCGKSCRLRWMNYLRPSLKRGQIAPDEEDLILRLHRLLGNRWALIAGRIPGRTDNDIKNYWNTHLSKKL
	Aquilegia coerulea v1.1	Aquca_002_00119.1	Ac2R-MYB19	KMNAARGQWTTEEDRKLVGLVKKYGDKHWTYIAHKMNGRIGKQCRERWHNHLRPNIKKETWTEDEDNALIKAHKELGNKWAEIAKRLPGRTENSIKNHWNATKRKQF
	Aquilegia coerulea v1.1	Aquca_013_00661.1	Ac2R-MYB20	KLGVKKGPWTAEEDKKLVNFILTNGQCCWRAVPKLAGLLRCGKSCRLRWTNYLRPDLKRGLLTEAEEQLVIDLHARLGNRWSKIAGRLPGRTDNEIKNHWNTHIKKKL
	Aquilegia coerulea v1.1	Aquca_045_00169.1	Ac2R-MYB21	GDQRIKGSWSPDEDARLTKLVEEHGPRNWSVISSGIPGRSGKSCRLRWCNQLSPLVQHRPFSPSEDAIIIKAHANHGNKWATIARLLPGRTDNAIKNHWNSTLRRRR
	Aquilegia coerulea v1.1	Aquca_006_00250.1	Ac2R-MYB22	KAHTNKGAWTKEEDDRLIAHIRAHGEGCWRSLPKAAGLLRCGKSCRLRWINYLRPDLKRGNFTEEEDELIIKLHSLLGNKWSLIAGRLPGRTDNEIKNYWNTHIRRKL
	Aquilegia coerulea v1.1	Aquca_017_00716.1	Ac2R-MYB23	KMGLKRGPWTPEEDQVLIAFIQNYGHGNWRALPKQAGLLRCGKSCRLRWTNYLRPDIKRGNFSNEEEETIIKLHEMLGNRWSAIAGRLPGRTDNEIKNVWHTHLKKRL
	Aquilegia coerulea v1.1	Aquca_002_01251.1	Ac2R-MYB24	MCTRGHWRPSEDEKLRELVEMYGPHNWNAIAEKLHGRSGKSCRLRWYNQLDPRINRSPFTEEEEERLLASHRIHGNRWAIIARHFPGRTDNAVKNHWHVIMARRC
	Aquilegia coerulea v1.1	Aquca_003_00309.1	Ac2R-MYB25	SQGWRKGPWTAEEDKLLTEYVTLHGEGRWSTVARSAGLNRNNKSCRLRWVNYLRPGLKRGQLTPEEESIIIELHAVWGNKWSMIARCLPGRTDNEIKN
	Aquilegia coerulea v1.1	Aquca_022_00293.1	Ac2R-MYB26	AKNCCPRGHWRPAEDEKLRQLVEQYGPQNWNSIADKLQGRSGKSCRLRWFNQLDPRINRRPFSEEEEERLLAAHRVHGNKWALIARLFPGRTDNSVKNHWHVIMARRQ
	Aquilegia coerulea v1.1	Aquca_030_00294.1	Ac2R-MYB27	KQKVKRGLWSPEEDEKLVKCINTHGHSCWSAVPKLAGLQRCGKSCRLRWINYLRPDLKRGSFSAQEERIIIDVHRILGNRWAQIAKHLPGRTDNEVKNFWNSCIKKKL
	Aquilegia coerulea v1.1	Aquca_030_00385.1	Ac2R-MYB28	KIGLKRGPWTPAEDKRLISYVEKNGHANWRALPTQAGLLRCGKSCRLRWINYLRPDIKRGNFSQEEETIIRLHESLGNKWSKIASHLPGRTDNEIKNVWNTHLKKRV
	Aquilegia coerulea v1.1	Aquca_073_00013.1	Ac2R-MYB29	HSHFNKGAWTEQEDQILINYIKLHGEGSWRQLPKAAGLLRCGKSCRLRWTNYLRPDLRRGNFTVEEDEIIIKLHSLHGNKWSLIAERLPGRTDNEIKNYWNTHIRRKL
	Aquilegia coerulea v1.1	Aquca_030_00038.1	Ac2R-MYB30	EEIMRKGPWLEEEDERLTTYVALMGERRWDSIARVSGLRRSGKSCRLRWLNYLRPDLKHGQMNAEEECVILQLQERWGNKWSKIARRLPGRTDNEIKNYWRTHLRKKT
	Aquilegia coerulea v1.1	Aquca_014_00947.1	Ac2R-MYB31	KVGIKKGPWTPEEDIILVSYIQEHGPGNWRSVPTNTGLLRCSKSCRLRWTNYLRPGIKRGNFNSHEEGMIVHLQALLGNKWAAIASYLPQRTDNDIKNYWNTHLKKKM
	Aquilegia coerulea v1.1	Aquca_042_00052.1	Ac2R-MYB32	KPIVTRGQWSAAEDQSLVRLVNTYGAKSWSLIAARLGSRNGKQCRDRWNNHLRPVIKRQAWSVEEDKAFIDAHKEIGNRWAEIAKRIPGRTEIAIKNRWNLYRRKLL
	Aquilegia coerulea v1.1	Aquca_005_00496.1	Ac2R-MYB33	EFGLKKGPWTPEEDDMLVDYIKRHGHGSWRALPKLAGLNRCGKSCRLRWTNYLRPDIKRGKFSEEEEQLIISLHSVLGNKWSQIATHLPGRTDNEIKNYWNTHLKKKL
	Aquilegia coerulea v1.1	Aquca_003_00583.1	Ac2R-MYB34	KVGLKKGRWTAEEDEILSKYIDANGEGSWRSLPKNAGLKRCGKSCRLRWINYLRGDLKRGNITPEEEDTIIKMHNTLGNRWSLIAGHLPGRTDNEIKNYWNSHLSRRI

Aquilegia coerulea v1.1	Aquca_025_00059.1	Ac2R-MYB35	EEGWRKGPWTAEEDKKLIEYVNLHGEGRWNSVAMFSGLKRNGKSCRLRWVNYLRPDLKRGKITPNEENIILELHARWGNRWSTIARSLPGRTDNEIKNYWRTHFKKSA
Aquilegia coerulea v1.1	Aquca_041_00154.1	Ac2R-MYB36	KQDKNKGAWSKQEDDKLIDYIQAHGEGCWRSLPQAAGLNRCHKSCRLRWINYLRPDLKHGNFAEDEEDLIIKLHALLGNRWSLIAGRLPGRTDNEVKNYWNSYLKRKL
Aquilegia coerulea v1.1	Aquca_068_00041.1	Ac2R-MYB37	KANVKKGPWAPEEDAKLKAYIEEYGTGGNWIALPQKIGLKRCGKSCRLRWLNYLRPNIKHGGFSDEEDSIICSLYISIGSRWSIIAAQLPGRTDNDIKNYWNTRLKKKL
Aquilegia coerulea v1.1	Aquca_002_00029.1	Ac2R-MYB38	MVMRKGPWMAEEDEILMDYVKKHGPRDWSSIRSKGLLPRTGKSCRLRWVNKLKPDLKTGCKFSAEEERVVIDLQARFGNKWARIATYLQGRTDNDVKNFWSTRQKRLA
Aquilegia coerulea v1.1	Aquca_011_00322.1	Ac2R-MYB39	KIGLKRGPWSIEEDYKLMNFIINNGTQCWRMVPKHAGLLRCGKSCRLRWINYLRPDLKRGPLSEVEEDQIIQLHARLGNRWSKIAEHFPGRTDNEIKNHWNTRIKRRL
Aquilegia coerulea v1.1	Aquca_038_00198.1	Ac2R-MYB40	KNGLKKGPWTPDEDQKLMDYIQKNGHGSWRTLPKNAGLQRCGKSCRLRWTNYLRPDIKRGRFSFEEEETIIQLHSVLGNKWSAIAARLPGRTDNEIKNYWNTHIRKRL
Aquilegia coerulea v1.1	Aquca_015_00086.1	Ac2R-MYB41	SSGLKKGKWSEEEDDLLKKCIEKYGEGNWHKVPLRTGLNRCRKSCRLRWCNYLNPKAKRGEFSWDEIDLIIKMQKLLGNRWSLIAARLQGRTSNDIKNLWTSHISKSL
Aquilegia coerulea v1.1	Aquca_025_00333.1	Ac2R-MYB42	MEKEGSYYKKGLWTEEEDKILMDYISVHGKGRWSRIARITGLKRCGKSCRLRWMNYLSPNVKKGDFSEEEEDLIIRLHKLLGNRWSLIAGRVPGRTDNQVKNHWNTHLSKKL
Aquilegia coerulea v1.1	Aquca_034_00423.1	Ac2R-MYB43	KAMYRKGLWSPEEDEKLRDYIFRYGHGCWSSLPSRVGLQRNGKSCRLRWINYLRPGLKRGVFTSQEDQTILALHQSLGNRWSQIAQQLPGRTDNEIKNYWHSCLKKKV
Aquilegia coerulea v1.1	Aquca_015_00088.1	Ac2R-MYB44	TTGLKKGTWTEEEDTLLKRCIEKYGEGNWHKVPLRAGLNRCRKSCRLRWINYLNPNIKREELSWDEIDLIIKMHKLLGNRWSLIAARLPGRTANDIKNRWNTHIFKSL
Aquilegia coerulea v1.1	Aquca_015_00085.1	Ac2R-MYB45	SSSLKRGKWSEEEDDLLKKCIEKYGEGNWHKVPPRAGLKRCRKSCRLRWCNYLNPNTNRREFSWDEIDLIIKMQKLLGNRWSLIAARLPGRTANDIKNRWTTHISKSL
Aquilegia coerulea v1.1	Aquca_020_00444.1	Ac2R-MYB46	KQKLRKGLWSPEEDEKLLRHITKYGHGCWSSVPKQAGLQRCGKSCRLRWINYLRPDLKRGTFSQQEENLIIELHAVLGNRWSQIAAQLPGRTDNEIKNLWNSCIKKKL
Aquilegia coerulea v1.1	Aquca_003_00245.1	Ac2R-MYB47	KQKVRRGLWSPEEDEKLINYISTYGHGCWSSVPRLAGLQRCGKSCRLRWINYLRPDLKRGSFSRQEASLIIELHRILGNRWAQIAKHLPGRTDNEVKNFWNSSIKKKL
Aquilegia coerulea v1.1	Aquca_009_00493.1	Ac2R-MYB48	KVGLKRGPWTPEEDRILSNYIKKEGEGRWRMLPKRAGLLRCGKSCRLRWMNYLRPSVKRGQIAPDEEDLILRLHRLLGNRWALIAGRIPGRTDNEIKNYWNTHLSKKL
Aquilegia coerulea v1.1	Aquca_034_00364.1	Ac2R-MYB49	KMGLKRGPWTPEEDHLLVSYIQENGHGNWRALPKLAGLLRCGKSCRLRWTNYLRPDIKRGNFTDEEEETIMQLHEKLGNRWSAIAARLPGRTDNEIKNVWHTHLKKRL
Aquilegia coerulea v1.1	Aquca_013_00221.1	Ac2R-MYB50	QQKVKRGLWSPEEDEKLIRYITTHGYGCWSEVPEKAGLQRCGKSCRLRWINYLRPDIRRGRFTPEEEKLIISLHGVVGNRWAHIASHLPGRTDNEIKNYWNSWIKKKIR
Aquilegia coerulea v1.1	Aquca_002_00170.1	Ac2R-MYB51	MEARSQYKRGLWTVEEDQILMDYIRVHGKGRWNRVAKLTGLKRCGKSCRLRWINYLSPSVKKGDFTEEEDDLIIRLHKLLGNRWSLIAGRVPGRTDNQVKNHWNTHLSKKL
Aquilegia coerulea v1.1	Aquca_007_00580.1	Ac2R-MYB52	KTDRIKGPWSAEEDQTLTRLVDIHGARNWTLISRYIPGRSGKSCRLRWCNQLSPNVEHRPFSQAEDEAILAAHQNYGNRWATIAKLLPGRTDNAVKNHWNSTLKRKQ
Aquilegia coerulea v1.1	Aquca_002_00135.1	Ac2R-MYB53	EGEVRKGPWTMEEDLILINYIANHGEGVWNSLARSAGLKRTGKSCRLRWLNYLRPDVRRGNITPEEQLLIMELHARWGNRWSKIARHLPGRTDNEIKNFWRTKIQKHI
Aquilegia coerulea v1.1	Aquca_002_00118.1	Ac2R-MYB54	SFGSIKGNWTHEEDRKLVRLVEKHGEKNWAKVSSCMGGRIGKQCRDRWKNHLEPGINKGCWTEDEDMALVEGHKKHNNRWAKIAEEIPGRTDNAVKNRWNSVKRRQS
Aquilegia coerulea v1.1	Aquca_007_00776.1	Ac2R-MYB55	DLDRIKGPWSPEEDEALQQLVQKHGPRNWSVISKSIPGRSGKSCRLRWCNQLSPQVEHRAFTPEEDEMIIKAHAKFGNKWATIARLLNGRTDNAIKNHWNSTLKRKC
Aquilegia coerulea v1.1	Aquca_011_00570.1	Ac2R-MYB56	MDTKRLVNKGTWTAEEDQRLAEYIEIHGAKKWKSVAVKAGLNRCEKSCRLRWLNYLRPNIKRGHFTDQEEDLIIRLHKLLGNRWSLIAGRIPGRTDNDIKNYWNSHLSKKL
Aquilegia coerulea v1.1	Aquca_076_00099.1	Ac2R-MYB57	KQGMNRGAWSAHEDKILTDYIKIHGQGKWRELPKRAGLKRCGKSCRLRWLNYLRPDIVRGNINRDEEELIVKLHKLLGNRWSLIAGRLPGRTDNEIKNYWNTNLAKRV
Aquilegia coerulea v1.1	Aquca_041_00011.1	Ac2R-MYB58	DFELRRGPWTLEEDSLLIHYIASHGEGRWDMLAKCSGLKRTGKSCRLRWLNYLKPDVRRGNLTLEEQVMILELHSKWGNRWSRIAQHLPGRTDNEIKNYWRTRVQKQA
Aquilegia coerulea v1.1	Aquca_011_00323.1	Ac2R-MYB59	KIGLKRGPWTIEEDHKLMSFIINNGIHCWRMIPKHAGLLRCGKSCRLRWINYLRPDLKRGTLSEFEENQIIQLHARLGNRWSKIAAHFPGRTDNEIKNHWNTRIKKRL
Aquilegia coerulea v1.1	Aquca_015_00089.1	Ac2R-MYB60	TTALKKGAWTEEEDTLLKRCIEKHGEGNWHKVPLRAGLNRCRKSCRLRWFNYLHPNIKRKWSLIAARLPGRTANDIKNRWNTHIYKSI
Aquilegia coerulea v1.1	Aquca_062_00019.1	Ac2R-MYB61	KANVKKGPWSPEEDSKLKDYIDKHGTGGNWIALPQKAGLKRCGKSCRLRWLNYLRPNIKHGEFSDDEDRIICSLFASIGSRWSIIAAQLPGRTDNDIKNYWNTKLKKKL
Aquilegia coerulea v1.1	Aquca_030_00245.1	Ac2R-MYB62	KTGLKRGAWTPEEDGLLSAYIMRYGHWNWREIPKYAGLSRCGKSCRLRWMNYLRPNIKRGNYTREEEELIINLHEKFGKRWSAIAASLPGRTDNEIKNHWHTHLKKRT
Aquilegia coerulea v1.1	Aquca_042_00080.1	Ac2R-MYB63	MLNGRIGKQCRERWQNHLCPDIKKQDWCVEEDKAFIDAHKEFGNHWAKIAKLIPGRTENAIKNRWNTYRRKYL
Aquilegia coerulea v1.1	Aquca_045_00020.1	Ac2R-MYB64	STTLIKGQWTEQEDRLLVKLVKENGVKKWSQIAHKMVGRAGKQCRERWHNHLRPDIKKDSWSEEEEIMLIEAHEKFGNRWAEIAKQIPGRSENAIKNHWNATKRRQT
Aquilegia coerulea v1.1	Aquca_083_00054.1	Ac2R-MYB65	DLDRIKGPWSPEEDDLLTKLVERHGPRNWSLISKSIPGRSGKSCRLRWCNQLSPQVEHRAFSAEEDETIIRAHAKFGNKWATIARLLSGRTDNAIKNHWNSTLKRKC
Aquilegia coerulea v1.1	Aquca_005_00025.1	Ac2R-MYB66	SVTLKKGPWTAAEDAILIEYVRKHGEGNWNAVQKNSGLSRCGKSCRLRWANHLRPNLKKGSFSPEEERLILELHAKLGNKWARMAAQLPGRTDNEIKNYWNTRVKRRQ
Aquilegia coerulea v1.1	Aquca_073_00028.1	Ac2R-MYB67	DFDIKRGPWTIDEDLLLVNYIATHGEGRWSSLARSAGLKRTGKSCRLRWLNYLRPDVRRGNITLEEQLTILQLHSQWGNRWSKIAQHLPGRTDNEIKNYWRTRVQKHA
Aquilegia coerulea v1.1	Aquca_002_00902.1	Ac2R-MYB68	EQGWRKGPWTAEEDKLLTEYVTLHGEGRWSTVARSAGLNRNSKSCRLRWVNYLRPGLKRGQLTPEEESIIIELHAVWGNKWSMIARCLPGRTDNEIKNYWRTHFKNKA
Aquilegia coerulea v1.1	Aquca_030_00246.1	Ac2R-MYB69	KTGLTRGTWTPEEDRLLTAYIKRYGHWNWRELPKYAALCIWLIFLFGDEGLGRLPESEEVTDSNKIAMQSLNSELGLSRCGKSCRLRWMNYLRPNIKRGDYTREEDELIINLHAEL GNRWSAIAARLPGRTDNEIKNHWHTHLQKRT
Aquilegia coerulea v1.1	Aquca_081_00025.1	Ac2R-MYB70	DIVIRKGPWMAEEDEILIDYVKKHGPRDWSSIRSKGLLLRTGKSCRLRWVNKLKPDLKTGCKFSAEEERVVIDLQARFGNKWARIATYLQGRTDNDVKNFWSSRQKRLA
Aquilegia coerulea v1.1	Aquca_014_00829.1	Ac2R-MYB71	NVKLRKGLWSPEEDDKLMNYMLSNGQGCWSDVARNAGLQRCGKSCRLRWINYLRPDLKRGAFSPQEEELIIHLHSVLGNRWSQIAARLPGRTDNEIKNFWNSTIKKRL
Aquilegia coerulea v1.1	Aquca_047_00006.1	Ac2R-MYB72	NSNVRKGAWNQDEDLLLKRCIEKYGEGNWHQVPMRAGNTRWTDPRLNRCRKSCRLRWLNYLHPNIKRGGFKEDEIDLIMKLHKLLGNRWSLIAGRLPGRTANDIKNYWNSHLS KKL
Aquilegia coerulea v1.1	Aquca_053_00075.1	Ac2R-MYB73	VKSCSRGHWKPGEDARLIELVSQFGPQNWNLIAEKLEGRSGKSCRLRWFNQLDPRINRKAFSQEEEEKLLAARRFYGNKWAVIARLFPGRTDNAVKNHWHVTMARRH
Aquilegia coerulea v1.1	Aquca_009_00018.1	Ac2R-MYB74	KDGIKKGPWTPEEDIILASYIQEHGPGNWRSVPTNTGLLRCSKSCRLRWTNYLRPGIKRGNFTSHEERMIVHLQALLGNKWASIASYLPQRTDNDIKNYWNTHLKKKI
Aquilegia coerulea v1.1	Aquca_015_00090.1	Ac2R-MYB75	SFTLNKGAWTEEEDRLLKKCIEIHGEGNWHKVPLRAGLNRCRKSCRLRWFNYLHPNIKRGEFAWDEIDLIIRMHKLLGNRWSLIAARLPGRTANDIKNRWNTHIHKSL
Aquilegia coerulea v1.1	Aquca_001_00435.1	Ac3R-MYB1	TRRSTKGQWTAEEDALLCRAVQRFQGKNWKKIAECFKERTDVQCLHRWQKVLNPELVKGPWSKEEDDIIVEMVKKYGAKKWSTIAQALPGRIGKQCRERWHNHLNPAINKDA WTQEEELTLIRAHQLYGNKWAELTKFLPGRTDNAIKNHWNSSVKKKL
Aquilegia coerulea v1.1	Aquca_013_00366.1	Ac3R-MYB2	TRRSTKGGWTEQEDDMLTKAVKRYNGKNWKKIAECFPGRSDVQCLHRWQKVLNPDLVKGPWSKEEDERILELVDKYGCKKWSVIAQSLPGRIGKQCRERWHNHLNPAIKKHA WTKEEELALIQAHQIYGNKWAEIAKFLPGRADNSIKNHWNCSVKKKL
Aquilegia coerulea v1.1	Aquca_003_00045.1	Ac3R-MYB3	PIRRAKGGWTPQEDETLRSAVEAFKGKCWKKIAEFFPDRSEVQCLHRWQKVLNPELIKGPWTQEEDDKIIELVAKYGPTKWSVIAKSLPGRIGKQCRERWHNHLNPMIKKDAWTL EEELSLMNAQREYGNKWAEIAKVLPGRTDNSIKNHWNSSLKKKL

Solanum tuberosum v3.4	PGSC0003DMP400000076	St2R-MYB001	KNGLTRGPWTPEEDEKLIQFINKNGHGSWRSLPKLAGLLRCGKSCRLRWTNYLRPNIIRGPFSPEEQKLVIQLHGILGNRWAAIASQLPGRTDNEIKNLWNTHLKKRL
Solanum tuberosum v3.4	PGSC0003DMP400000691	St2R-MYB002	KTKVKRGPWSPEEDNILKNYLEKNGTGGNWISLPQKAGLKRCGKSCRLRWLNYLRPDIKHGGFTEEEDNIILTLYRQIGSRWSVIAANLPRRTDNDVKNHWNTKLKKKH
Solanum tuberosum v3.4	PGSC0003DMP400000947	St2R-MYB003	DVEVRKGPWTMEEDLILINYIANHGEGVWNSLARSAGLKRTGKSCRLRWLNYLRPDVRRGNITPEEQLLIMELHAKWGNRWSKIAKHLPGRTDNEIKNFWRTRIQKHI
Solanum tuberosum v3.4	PGSC0003DMP400002378	St2R-MYB004	KTGVKKGPWTPEEDIMLVSFVQEHGPGNWRTVPTHTGSFLFLLDLYSQSEFRARLIVWLRRCSKSCRLRWTNYLRPGIKRGSFTDQEEKMIIQLQALLGNKWAAIASYLPERTDND IKNYWNTHLKKKL
Solanum tuberosum v3.4	PGSC0003DMP400002398	St2R-MYB005	KVGLKKGPWTPEEDQKLLAYIEEHGHGSWRALPAKAGLQRCGKSCRLRWTNYLRPDIKRGKFTLQEEQTIIQLHALLGNRWSAIATHLPKRTDNEIKNYWNTHLKKRL
Solanum tuberosum v3.4	PGSC0003DMP400002480	St2R-MYB006	EQGWRKGPWTPEEDKLLSEYVNLHGEGRWSSVSRCAGLNRTGKSCRLRWVNYLRPGLKRGHITPQEEGIIIELHALWGNKWSTIARYLPGRTDNEIKNYWRTHFKTKV
Solanum tuberosum v3.4	PGSC0003DMP400002707	St2R-MYB007	EVGWRKGPWTSDEDRLLIEYVKLHGEGRWNSVARLTGLRRNGKSCRLRWVNYLRPDLKRGQITPHEERIILELHARWGNRWSTIARSLPGRTDNEIKNYWRTHFKKKS
Solanum tuberosum v3.4	PGSC0003DMP400003606	St2R-MYB008	KANVKKGPWSPEEDAKLKEYIDKFGTGGNWIALPQKAGLRRCGKSCRLRWLNYLRPNIKHGEFSDEEDRIICSLYANIGSRWSIIAAQLPGRTDNDIKNYWNTKLKKKL
Solanum tuberosum v3.4	PGSC0003DMP400005110	St2R-MYB009	KEGLKKGPWTHDEDQKLLAYVEEHGYGSWSDLPLRAGLQRCGRSCRLRWINYLRPNIKRGKFSSEEERTIFKLHALLGNRWSTIASHLPNRSDNEIKNYWNTHLKKRL
Solanum tuberosum v3.4	PGSC0003DMP400005118	St2R-MYB010	VCFELQEDDILREQIRIHGIDDNWTIIASNFKDKTTRQCKRRWFTYLNSDFKKGGWSPEEDMLLCAAQKIFGNRWTEIAKVVSGRTDNAVKNRFTTICKKKA
Solanum tuberosum v3.4	PGSC0003DMP400005949	St2R-MYB011	RIKGPWSPEEDELLQTLVEKHGPRNWTLISKSVPGRSGKSCRLRWCNQLSPQVEHRAFTPEEDDTIIRAHAKYGNKWATIARLLSGRTDNAIKNHWNSTLKRKC
Solanum tuberosum v3.4	PGSC0003DMP400006918	St2R-MYB012	KANVKKGPWSPEEDAKLKEYIDKFGTGGNWIALPQKAGLRRCGKSCRLRWLNYLRPNIKHGEFSDEEDRIICSLYGNIGSRWSIIAAQLPGRTDNDIKNYWNTKLKKKL
Solanum tuberosum v3.4	PGSC0003DMP400007770	St2R-MYB013	KVGLKRGRWTVEEDERLTNYIQANGEGSWRTLPKNAGLLRCGKSCRLRWINYLKSDLKRGNITSDEEAIIIKLRATLGNRWSLIAKHLPHRTDNEIKNYWNSRLSRKV
Solanum tuberosum v3.4	PGSC0003DMP400008158	St2R-MYB014	ELRRGPWSVEEDFVLMNYISHHGEGRWNSLSRCAGLKRTGKSCRLRWLNYLRPDVRRGNITLEEQLLILQLHSRWGNRWSKIAQHLPGRTDNEIKNYWRTRVQKHA
Solanum tuberosum v3.4	PGSC0003DMP400008159	St2R-MYB015	NLDLKRGSWTVEEDFTLMNHIALHGEGRWNSLARSAGLKRTGKSCRLRWLNYLRPDVRRGNITLEEQLLILQLHSRWGNRWSKIAQHLPGRTDNEIKNYWRTRVQKQA
Solanum tuberosum v3.4	PGSC0003DMP400008160	St2R-MYB016	ELRRGSWTVEEDYILMNYISHHGEGRWNSLSRCAGLKRTGKSCRLRWLNYLRPNVRHGNITLEEQLLILQLHFRWGNRWSKIAEHLPGRTDNEIKNYWRTRVQKHA
Solanum tuberosum v3.4	PGSC0003DMP400008480	St2R-MYB017	KLGVKKGPWTAEEDKKLINFILTNGQCCWRAVPKLAGLRRCGKSCRLRWTNYLRPDLKRGLLSEAEEKLVIDLHARLGNRWSKIAARLPGRTDNEIKNHWNTHIKKKL
Solanum tuberosum v3.4	PGSC0003DMP400008822	St2R-MYB018	KNNVKRGPWSPEEDAKLKSYIEQNGTGGNWIALPQKIGLKRCGKSCRLRWLNYLRPNIKHGGFSEEEDRIICSLYISIGSRWSIIAAQLPGRTDNDIKNYWNTKLKKKL
Solanum tuberosum v3.4	PGSC0003DMP400009936	St2R-MYB019	DIELRRGPWTIEEDKLLVHYITNHGEGRWNMLAKHAGLKRTGKSCRLRWLNYLKPDVKRGNLTPQEQLLILELHSKLGNRWSKIAQYLPGRTDNEIKNYWRTRVQKQA
Solanum tuberosum v3.4	PGSC0003DMP400010367	St2R-MYB020	KEGLRKGPWSTKEDLLLANYIKENGEGQWRNLPNKAGLLRCGKSCRLRWMNYLRPGIKRGNFSQDEEDLIIRLHSLLGNRWSLIAGRLPGRTDNEIKNYWNTHVIKKL
Solanum tuberosum v3.4	PGSC0003DMP400010496	St2R-MYB021	GLPLKKGPWTSAEDAILVEYVTKHGEGNWNAVQKHSGLARCGKSCRLRWANHLRPDLKKGAFTPEEERHIIELHAKMGNKWARMAAELPGRTDNEIKNYWNTRIKRRQ
Solanum tuberosum v3.4	PGSC0003DMP400010956	St2R-MYB022	KAHTNKGAWTKEEDERLISYIRTHGEGCWRSLPKAAGLLRCGKSCRLRWINYLRPDLKRGNFTEEEDELIIKLHSLLGNKWSLIAGRLPGRTDNEIKNYWNTHIRRKL
Solanum tuberosum v3.4	PGSC0003DMP400011365	St2R-MYB023	ENGLKKGPWTPEEDQILVDYIDKHGHGSWRALPKLAGLNRCGKSCRLRWTNYLRPDIKRGKFSEEEEQTILHLHSILGNKWSAIATHLSGRTDNEIKNFWNTHLKKKL
Solanum tuberosum v3.4	PGSC0003DMP400012169	St2R-MYB024	LGKLRKGLWSPEEDEKLMSYMLTNGQGCWSDIARNAGLQRCGKSCRLRWINYLRPDLKRGAFSLQEEELIVHLHSILGNRWSQIAARLPGRTDNEIKNFWNSTIKKRL
Solanum tuberosum v3.4	PGSC0003DMP400012270	St2R-MYB025	KVGVKKGPWTPEEDIILVSYVQQHGPGNWRALPTKTGLHRCSKSCRLRWTNYLRPGIKRGNFTDQEEKMIIQLQALLGNKWAAIASYLPERTDNDIKNYWNTYLKKKM
Solanum tuberosum v3.4	PGSC0003DMP400012935	St2R-MYB026	KVGLKRGRWTSEEDEIITNYIQANGEGSWRSLPKNAGLLRCGKSCRLRWINYLKTDLKRGNITSDEEAIIIKLRATLGNRWSLIVEHLPGRTDNEIKNYWNSHLSRKV
Solanum tuberosum v3.4	PGSC0003DMP400012972	St2R-MYB027	KQKVKRGLWSPEEDEKLIKHINTHGNSCWSSVPKLAGLQRCGKSCRLRWINYLRPDLKRGCFSEQEERIIIDVHRILGNRWAQIAKHLPGRTDNEVKNFWNSCIKKKL
Solanum tuberosum v3.4	PGSC0003DMP400013034	St2R-MYB028	NVKLRKGLWSPEEDEKLMHYMLTNGQGCWSDVARNAGLQRCGKSCRLRWINYLRPDLKRGAFSPQEEEHIIHLHSILGNRWSQIAARLPGRTDNEIKNFWNSTLKKRL
Solanum tuberosum v3.4	PGSC0003DMP400014055	St2R-MYB029	SERIKGPWSAEEDKILTKLVERYGARNWSLISKYIKGRSGKSCRLRWCNQLSPNVEHRPFSPAEDEAILAAHAKYGNRWATIARLLPGRTDNAVKNHWNSTLKRRY
Solanum tuberosum v3.4	PGSC0003DMP400014627	St2R-MYB030	IKKGAWSPEEDQKLRGYIMKYGIWNWKQMPKFAGLSRTGKSCRLRWMNYLRPDVRRGPFTMEEVEIVIKTYQELGNSWSAIAAKLPGRTDNEVKNFFHAHLKKHL
Solanum tuberosum v3.4	PGSC0003DMP400014987	St2R-MYB031	KANVKRGPWTAEEDAKILAYVATHGIGNWTLVPQKAGLNRCGKSCRLRWTNYLRPDLKHDNFTPQEEDCILELHKTIGSRWSLIAKQLPGRTDNDVKNYWNTKLKKKL
Solanum tuberosum v3.4	PGSC0003DMP400015271	St2R-MYB032	KDNVKRGQWTPEEDHKLSSYIAQHGTRNWRLIPKHAGLQRCGKSCRLRWTNYLRPDLKHGQFSEGEEQTIVTLHSVLGNRWSVIAAQLPGRTDNDVKNHWNTKLKKKL
Solanum tuberosum v3.4	PGSC0003DMP400015397	St2R-MYB033	DMDLRRGPWTVEEDFTLMNYIAHHGEGRWNSLARCAGLKRTGKSCRLRWLNYLRPDVRRGNITLEEQLLILELHSRWGNRWSKIAQHLPGRTDNEIKNYWRTRVQKHA
Solanum tuberosum v3.4	PGSC0003DMP400015891	St2R-MYB034	KVGIKRGRWTAEEDQILTNYILSNGEGSWRSLPKNAGLLRCGKSCRLRWINYLRSDLKRGNITSQEEDIIIKLHATLGNRWSLIAGHLSGRTDNEIKNYWNSHLSRKV
Solanum tuberosum v3.4	PGSC0003DMP400019896	St2R-MYB035	KSKVKKGPWTTSEDLKLISFIQKHGHGNWRALPKQAGLLRCGKSCRLRWINYLRPDVKRGNFTPQEEDTIINLHRAFGNRWSKIASHLPGRTDNEIKNVWNTHLKKRL
Solanum tuberosum v3.4	PGSC0003DMP400019914	St2R-MYB036	KNGLKKGPWTTEEDQKLIDYIQKNGSGNWRILPKNAGLQRCGKSCRLRWTNYLRPDIKRGKFSLEEEETIIHLHSILGNKWSAIAARLPGRTDNEIKNYWNTHIRKKL
Solanum tuberosum v3.4	PGSC0003DMP400020363	St2R-MYB037	RIMIKGGVWKNTEDEILKAAVMKYGKNQWARISSLLVRKSAKQCKARWYEWLDPSIKKTEWTREEDEKLLHLAKLMPTQWRTIAPIVGRTPSQCLERYEKLLDAAC
Solanum tuberosum v3.4	PGSC0003DMP400021198	St2R-MYB038	KIGLKRGPWTIEEDHRLMNFILNNGIQCWRLVPKLAGLMRCGKSCRLRWINYLRPDLKRGALTEAEEDMIIKLHSQLGNRWSKIAGHFPGRTDNEIKNHWNTRIKKKL
Solanum tuberosum v3.4	PGSC0003DMP400021429	St2R-MYB039	KEGLRKGPWSTKEDLLLTNYINQYGEGQWRSLPKNAGLLRCGKSCRLRWVNYLKPGIKRGNFSEDEEDLIVRLHSLLGNRWSLIAGRLPGRTDNEIKNYWNTHLIKKL
Solanum tuberosum v3.4	PGSC0003DMP400022407	St2R-MYB040	RVGLKRGPWTIEEDHKLVHFILNNGIQCWRTVPKLAGLQRCGKSCRLRWINYLRPDLKRGALSEAEEDQIIELHARLGNRWSKIASYFPGRTDNEIKNHWNTRIKKRL
Solanum tuberosum v3.4	PGSC0003DMP400023376	St2R-MYB041	KAHTNKGAWTKEEDERLISYIRTHGEGCWRSLPKAAGLLRCGKSCRLRWINYLRPDLKRGNFSEEEDELIIKLHSLLGNKWSLIAGRLPGRTDNEIKNYWNTHIRRKL
Solanum tuberosum v3.4	PGSC0003DMP400023726	St2R-MYB042	KTSLKKGPWDHEEDQILISYINKNGHSNWRALPKQAGLLRCGKSCRLRWMNYLSPDIKRGNFTKEEEDNIINLHEILGNRWSAIAARLPGRTDNEIKNIWHTHLKKRL
Solanum tuberosum v3.4	PGSC0003DMP400024309	St2R-MYB043	KSGLKKGTWTLEEDKKLAAYVTKYGCWNWRQLPKFAGLSRCGKSCRLRWLNYLQPNIKRGNYSREEDQIIMKLHAEIGNKWSAIAIHLPGRSDNEIKNHWHTSLKKRS
Solanum tuberosum v3.4	PGSC0003DMP400024514	St2R-MYB044	ARQLKKGPWTTTEDAILMEYVKKHGEGNWNAVQRNSGLMRCGKSCRLRWANHLRPHLKKGAFSLEEERLIVELHAKLGNKWARMAAQLPGRTDNEIKNYWNTRLKRRQ

Solanum tuberosum v3.4	PGSC0003DMP400025180	St2R-MYB045	KQKHKKGLWSPDEDDRLKNYIIKHGHGCWSSVPINAGLQRNGKSCRLRWINYLRPGLKRGAFSLEEEDTILTLHAMFGNKWSQIAQHLPGRTDNEIKNHWHSYLKKRV
Solanum tuberosum v3.4	PGSC0003DMP400025629	St2R-MYB046	ELFELRRGPWTLEEDNLLIHYISTHDEGRWNALAKCAGLKRTGKSCRLRWLNYLKPDIKRGNLTPQEQLLILELHSKWGNRWSKIAQHLPGRTDNEIKNYWRTRVQKQA
Solanum tuberosum v3.4	PGSC0003DMP400027224	St2R-MYB047	EEIMRRGPWTEEEDVQLLFYVKLFGDRRWDFLAKVSGLKRTGKSCRLRWVNYLNPDLKHGKMTPQEERLVLELHSKWGNRWSRIARKIPGRTDNEIKNYWRTHMRKKA
Solanum tuberosum v3.4	PGSC0003DMP400027453	St2R-MYB048	KVGLKKGPWTAEEDKKLINFILNNGQCCWRAVPKLAGLLRCGKSCRLRWTNYLRPDLKRGLLSEYEEKMVIDLHAQLGNRWSKIASHLPGRTDNEIKNHWNTHIKKKL
Solanum tuberosum v3.4	PGSC0003DMP400027651	St2R-MYB049	KQKLRKGLWSPEEDEKLINHITKYGHGCWSSVPKLAALQRCGKSCRLRWINYLRPDLKRGTFSQQEENLIIQLHSLLGNKWSQIASRLPGRTDNEIKNLWNSSIKKKL
Solanum tuberosum v3.4	PGSC0003DMP400028466	St2R-MYB050	KMGLKKGPWSPEEDNILISYIQNNGHSNWRALPKLAGLLRCGKSCRLRWTNYLRPDIKRGNFTKEEEDTIIKLHENLGNRWSAIAARLPGRTDNEIKNIWHTNLKKKL
Solanum tuberosum v3.4	PGSC0003DMP400030100	St2R-MYB051	KMGLKKGPWTPEEDQILISYIQSNGHGNWRALPKLAGLLRCGKSCRLRWTNYLRPDIKRGNFTREEEDSIIQLHEMLGNRWSAIAARLPGRTDNEIKNVWHTHLKKRL
Solanum tuberosum v3.4	PGSC0003DMP400030609	St2R-MYB052	DEDLKKGPWTSEEDEKLMDFISKNGHTNWQLIPKKAGLNRCGKSCRLRWTNYLRPDIKRGEFSIEEEEIIINLHSLLGNKWSRIAAHLPGRTDNEIKNFYNTHLRKKL
Solanum tuberosum v3.4	PGSC0003DMP400031526	St2R-MYB053	KSKSVKGQWTIEEDRILIDLVERFGDRKWSQIAQVLKGRIGKQCRERWHNHLRPDIKKDLWTEEEDMILIKAHAEIGNKWAEIAKKLAGRTENSIKNHWNATKRRQF
Solanum tuberosum v3.4	PGSC0003DMP400031557	St2R-MYB054	KQKLRKGLWSPEEDEKLCNYITNFGIGSWSSVPKLAGLQRCGKSCRLRWINYLRPDLKRGMFSQDEEDKIISLHQVLGNRWAQIAAQLPGRTDNEIKNFWNSSLKKKL
Solanum tuberosum v3.4	PGSC0003DMP400031848	St2R-MYB055	KQKLRKGLWSPEEDEKLIKHITKFGHGCWSSVPKLAGLQRCGKSCRLRWINYLRPDLKRGTFSQDEENLIIELHAVLGNKWSQIAARLPGRTDNEIKNLWNSSIKKKL
Solanum tuberosum v3.4	PGSC0003DMP400031943	St2R-MYB056	KNGLKKGPWTPEEDNKLIQYIQLHGPGNWRTLPKNAGLQRCGKSCRLRWTNYLRPDIKRGRFSFDEEETIIQLHSVLGNKWSAIAARLPGRTDNEIKNYWNTHIRKRL
Solanum tuberosum v3.4	PGSC0003DMP400032077	St2R-MYB057	KEGLKKGPWTPEEDQKLLSFIDKHGCGSWRALPAKAGLQRCGKSCRLRWINYLRPDIKRGKFSLQEERTIIQLHALLGNRWSAIAAYLPSRTDNEIKNYWNSRLKKRL
Solanum tuberosum v3.4	PGSC0003DMP400032103	St2R-MYB058	ARGHWRPAEDAKLKELVAIYGPQNWNLIAEKLEGRSGKSCRLRWFNQLDPRINRRAFSEEEEERLMAAHRLYGNKWAMIARLFPGRTDNAVKNHWHVIMARKY
Solanum tuberosum v3.4	PGSC0003DMP400032125	St2R-MYB059	EEGLKKGPWTHEEDQKLLSFIDKHGCGSWRGLPAKAGLQRCGKSCRLRWINYLRPDIKRGKFSLQEERTIIHLHALLGNRWSAIATYLPSRTDNEIKNYWNSRLKKRL
Solanum tuberosum v3.4	PGSC0003DMP400032674	St2R-MYB060	KANVKKGPWSPEEDATLKAYIEENGTGNNWIALPQKIGLKRCGKSCRLRWLNYLRPNIKHGGFTEEEDNIICSLYISIGSRWSIIAAQLPGRTDNDIKNYWNTRLKKKL
Solanum tuberosum v3.4	PGSC0003DMP400033945	St2R-MYB061	KIGVKKGPWTPEEDIILVSYIQQHGPGNWRAVPSNTGLLRCSKSCRLRWTNYLRPGIKRGNFTEHEEKMIIHLQALLGNRWAAIASYLPQRTDNDIKNYWNTHLRKKL
Solanum tuberosum v3.4	PGSC0003DMP400034714	St2R-MYB062	YVKKGAWSPEEDQKMIDYIMQYRIWNWSHMPKFAGLSRTGKSCRLRWINYLKPDLKKGPFSMEEVEIVFRMYQSLGNKWSAIAKELPGRTDNEIKNFYHTHLKKHH
Solanum tuberosum v3.4	PGSC0003DMP400034715	St2R-MYB063	MDYVKKGAWSPEEDQKLIDYIMQYRIWNWSHMPKFAGLSRTGKSCRLRWINYLRPDLKKGPFSIEEVEIVIRMYQSLGNRWSAIAKELPGRTDNEIKNFYHTHLKKHL
Solanum tuberosum v3.4	PGSC0003DMP400034798	St2R-MYB064	KLGLKRGPWSKEEDDLLINYIKKNGHPNWRALPKLAGLLRCGKSCRLRWTNYLRPDIKRGNFTHQEEDTIIKLHQVLGNSWSAIAARLPGRTDNEIKNIWHTRLKKKM
Solanum tuberosum v3.4	PGSC0003DMP400035326	St2R-MYB065	TADRVKGPWSPEEDAILSRLVSNFGARNWSLIARGIPGRSGKSCRLRWCNQLDPAVKRKPFTDEEDRIILQAHAIHGNKWASIARLLPGRTDNAIKNHWNSTLRRRC
Solanum tuberosum v3.4	PGSC0003DMP400037090	St2R-MYB066	KANVKRGPWSPEEDSKLKAYIEQHGTGGNWITLPQKVGLKRCGKSCRLRWLNYLRPNIKHGEFTDEEDNIICTLYMSIGSRWSIIAAQLPGRTDNDIKNYWNTRLKKKL
Solanum tuberosum v3.4	PGSC0003DMP400037546	St2R-MYB067	KKGLKKGPWTPEEDEKLVEYIKNHGHGSWRSLPHLAGLARCGKSCRLRWTNYLRPDIKRGPFSHDEEKLVIQLHGILGNRWAAIASQLPGRTDNEIKNLWNTHLKKRL
Solanum tuberosum v3.4	PGSC0003DMP400038822	St2R-MYB068	KNGLKKGPWTPEEDQKLIDYIQKHGYGNWRTLPKNAGLQRCGKSCRLRWTNYLRPDIKRGRFSFEEEETIIQLHSILGNKWSAIAARLPGRTDNEIKNYWNTHIRKRL
Solanum tuberosum v3.4	PGSC0003DMP400039333	St2R-MYB069	SVPLKKGPWTSAEDVILVDYVMTHGEGNWNAVQRHSGLARCGKSCRLRWANHLRPDLKKGAFTPEEEQRIVELHAKMGNKWARMAVELPGRTDNEIKNYWNTRIKRRQ
Solanum tuberosum v3.4	PGSC0003DMP400040480	St2R-MYB070	KMGLKKGPWTQEEDQILITFIQKYGHENWRALPKQAGLLRCGKSCRLRWTNYLRPDIKRGNFSEEEEQIIIKLHQLLGNRWSAIASRLPGRTDNEIKNFWHTHLKKRL
Solanum tuberosum v3.4	PGSC0003DMP400040530	St2R-MYB071	NFGLKKGKWTDEEDELLVKYIQVNGEGSWRSLPKNAGLSRCGKSCRLRWTNYLRPNLKRGKFTAEEDEIIVKLHSTLGNRWSSIAQLLSGRTDNDIKNYWNSNLRRRI
Solanum tuberosum v3.4	PGSC0003DMP400042037	St2R-MYB072	KVGLKKGPWTPEEDQKLMDYIEKNGCGSWRALPTKAGLKRCGKSCRLRWINYLRPDIKRGKFSLQEEQTIIQLHALLGNRWSAIATHLANRTDNEIKNYWNTHLKKRL
Solanum tuberosum v3.4	PGSC0003DMP400043024	St2R-MYB073	RSSCQRGHWRPAEDERLRQLVEQYGPQNWNSIAEKLQGRSGKSCRLRWFNQLDPRINRRPFSEDEEERLIGAHRIHGNKWALISRLFPGRTDNAVKNHWHVLMARKQ
Solanum tuberosum v3.4	PGSC0003DMP400043359	St2R-MYB074	RVKGPWSPEEDELLQQLVQKHGPRNWSLISKSIPGRSGKSCRLRWCNQLSPQVEHRAFTPEEDETIIRAHARFGNKWATIARLLNGRTDNAIKNHWNSTLKRKC
Solanum tuberosum v3.4	PGSC0003DMP400044377	St2R-MYB075	PKERHIVSWSQEEDDILREQIRIHGTDNWTIIASKFKDKTTRQCRRRWFTYLNSDFKKGGWSPEEDMLLCEAQKIFGNRWTEIAKVVSGRTDNAVKNRFTTLCKKRA
Solanum tuberosum v3.4	PGSC0003DMP400045417	St2R-MYB076	ESGRKKGTWTLEEDKKLAAYITKYGSWNWRQLPKYAGLARCGKSCRLRWINHLRPNVKRGNYTKEEDEIILNLHAQLGNKWSTIAAHLPERSDNEIKNHWHTTLKKRA
Solanum tuberosum v3.4	PGSC0003DMP400045418	St2R-MYB077	ENGRKKGTWTPEEDRKLAAYITKYGSWNWRQLPKYAGLARCGKSCRLRWMNYLSPNVKRGNYTKEEDEIILNLHAQLGNKWSAIAVHLPGRSDNEIKNHWHTALKKRT
Solanum tuberosum v3.4	PGSC0003DMP400046268	St2R-MYB078	KNGLKKGPWTPEEDQKLIQYIQVHGPGNWRNLPKNAGLQRCGKSCRLRWTNYLRPDIRRGRFSFEEEETIIQLHSVLGNKWSAIAARLPGRTDNEIKNYWNTHIRKRL
Solanum tuberosum v3.4	PGSC0003DMP400046481	St2R-MYB079	ITTCIDVCFELQEDDILREQIRIHGIDDNWTIIASNFKDKTTRQCKRRWFTYLNSDFKKGGWSPEEDMLLCAAQKIFGNRWTEIAKVVSGRTDNAVKNRFTTICKKKA
Solanum tuberosum v3.4	PGSC0003DMP400047028	St2R-MYB080	KEKTRKGLWSPEEDEKLYNYITRFGVGCWSSVPKLAGLQRCGKSCRLRWINYLRPDLKRGMFSQEEEDMIITLHKVVGNRWAQIAAKLPGRTDNEIKNFWNSNLKKKL
Solanum tuberosum v3.4	PGSC0003DMP400047194	St2R-MYB081	VMELRRGPWTVEEDFTLINFIAHHGEGRWNSLSRCAGLKRTGKSCRLRWLNYLRPDVRRGNITLEEQLLILELHSRWGNRWSKIAQHLPGRTDNEIKNYWRTRVQKHA
Solanum tuberosum v3.4	PGSC0003DMP400047253	St2R-MYB082	SSGRVKEPWSPEEDGILTELVNKFGARNWSMIAQGIAGRSGKSCRLRWCNQLDPVLKRKPFTDEEDHIIINAHAVHGNKWAAIAKLLPGRTDNAIKNHWNSTLRRRF
Solanum tuberosum v3.4	PGSC0003DMP400047280	St2R-MYB083	EEWRKGPWTVEEDKLLIEYVNLHGEGRWNSVARLAGLKRNGKSCRLRWVNYLRPDLKRGQITPYEERIILELHAIWGNRWSTIARSLPGRTDNEIKNYWRTNFKKKF
Solanum tuberosum v3.4	PGSC0003DMP400047694	St2R-MYB084	GSPLKKGPWIKAEDAILMDYVAKYGEGNWSSVHRRTGLARCGKSCRLRWANHLKPDLKKGAFTQEEEELIIEMHAKIGNKWARMAAEFPGRTDNDIKNFWNTRLKRQR
Solanum tuberosum v3.4	PGSC0003DMP400047957	St2R-MYB085	VKKGAWSPQEDKKLIDYIAKYRNWNWSQMPKFAGLSRTGKSCRLRWTNYLRPDLKRGPFSLEETQTVIRMYQSLGSRWSAIAKELPGRTDNEIKNFYHTHLKKHL
Solanum tuberosum v3.4	PGSC0003DMP400048854	St2R-MYB086	SRGHWRPHEDAKLKELVGQYGPQNWNLIAEKLEGRSGKSCRLRWFNQLDPRINRKAFSEEEEERLLVAHKMYGNKWSMIARLFPGRTDNAVKNHWHVIMARKH
Solanum tuberosum v3.4	PGSC0003DMP400048867	St2R-MYB087	KIGIKKGPWTPEEDIILVSYIQEHGPGNWRSVPTNTGLMRCSKSCRLRWTNYLRPGIKRGNFTPHEEGMIIHLQALLGNKWAAIASYLPQRTDNDIKNYWNTHLKKKL
Solanum tuberosum v3.4	PGSC0003DMP400049170	St2R-MYB088	EEELRRGQWLEEEDERLSMMVGVFGERRWDALAKTSGLKRSGKSCRLRWLNYLRPNLKHGHITADEERLIIRLQKQFGNKWSKIAKQLPGRTDNEIKNYWRSHLRKKT
Solanum tuberosum v3.4	PGSC0003DMP400050448	St2R-MYB089	RQALKKGPWTAGEDAILMDYVKKHGEGNWNAVQRNSGLMRCGKSCRLRWANHLRPHLKKGAFTPEEERIIIDLHAKLGNKWARMAAQLPGRTDNEIKNYWNTRLKRRQ

Solanum tuberosum v3.4	PGSC0003DMP400053171	St2R-MYB090	KEEMHKGAWSKQEDQKLIDYITKHGEGCWRDLPKAAGLLRCGKSCRLRWMNYLNPNLKRGNFSEDEDDLIIKLHALLGNRWSLIAGRLPGRTDNEVKNYWNSYLRRKL
Solanum tuberosum v3.4	PGSC0003DMP400053853	St2R-MYB091	RMKRGFWKPEEDLILKNCVETHGEGNWATISEKSGLMRSGKSCRLRWKNYLRPNIKRGMMSEDEKDLIIRLHKLLGNRWSLIAGRLPGRTDNEVKNFWNTHLNNKR
Solanum tuberosum v3.4	PGSC0003DMP400054072	St2R-MYB092	SRGHWRPHEDEKLRELVAKYGPHNWNAIALNLQGRSGKSCRLRWYNQLDPRINRSPFTEEEEERLLSSHRIHGNRWAMIARLFPGRTDNAVKNHWHVIMSRKC
Solanum tuberosum v3.4	PGSC0003DMP400054558	St2R-MYB093	KAHTNKGAWTKEEDQRLINYIRAHGEGCWRSLPKAAGLSRCGKSCRLRWINYLRPDLKRGNFTEEEDELIIKLHSLLGNKWSVIAGRLPGRTDNEIKNYWNTHIKRKL
Solanum tuberosum v3.4	PGSC0003DMP400055794	St2R-MYB094	KTGVKKGPWTPEEDIILVSYIQEHGPGNWRNIPTNTGLLRCSKSCRLRWTNYLRPGIKRGNFTEHEEKMIIHLQALLGNRWAAIASYLPQRTDNDIKNYWNTHLKKKL
Solanum tuberosum v3.4	PGSC0003DMP400056681	St2R-MYB095	MIKRRWNPEEDELLQKLVEEHGPKNWFLIGQLIPGRSEKLCRFRWCNQRNPQVDHRPFTLDEDDTIIKAHAKFGNQWAMIACLLPGRTDNAIKNHWSSFLKRKR
Solanum tuberosum v3.4	PGSC0003DMP400057504	St2R-MYB096	KEGLKKGSWTKKEDLLLTEYIKDFGEGTGWRSLPKKAGLLRCGKSCRLRWVNYLKPGLNRGTFTPEEDDLIVRLYTLLGSRWSLIAGRISGRTDNDVKNYWNTSLLKKL
Solanum tuberosum v3.4	PGSC0003DMP400058999	St2R-MYB097	KSKSSKGQWTIEEDRLLIHLVEKFGIRKWSQIAQMLKGRIGKQCRERWHNHLRPDIKKDFWTEEEDRILIQAHGEVGNKWAEIAKRLPGRTENSIKNHWNATKRRQF
Solanum tuberosum v3.4	PGSC0003DMP400059142	St2R-MYB098	KQKVKRGLWSPEEDEKLINYITNYGHACWSSVPKLAGLQRCGKSCRLRWINYLRPDLKRGSFSPQEAALIIELHGILGNRWAQIAKHLPGRTDNEVKNFWNSSIKKKL
Solanum tuberosum v3.4	PGSC0003DMP400061017	St2R-MYB099	KSKVKKGAWSCEEDLRLITFIQKHGHQNWRSLPKQAGLLRCGKSCRLRWINYLRPDLKRGNFTPQEEDTIIKLHQSFGNKWSKIASHLPGRTDNEIKNVWNTHLKKRR
Solanum tuberosum v3.4	PGSC0003DMP400065030	St2R-MYB100	STNMKKGAWSPDEDQKLKSYIMKFGIWNWSHMPKFAGLSRTGKSCRLRWMNYLRPDIKRGPFTMEEVEIVIKTYQELGNRWSAIAGRLPGRTDNEIKNFFHTHLKKQL
Solanum tuberosum v3.4	PGSC0003DMP400066245	St2R-MYB101	TNYLIKGQWTDEEDRKLLRLVKQFGVRKWAQIAEKMEARAGKQCRERWHNHLRPDIKKDTWSEEEELLLVESHKQLGNKWAEIAKRIPGRTENAIKNHWNATKRRQN
Solanum tuberosum v3.4	PGSC0003DMP400066962	St2R-MYB102	MIKRRWNPEEDELLHKLVKEHGAENWSLISQLISSRSGKSCRFWWCNQFNPQVEHRPFTLEEDNTIIRAHAKLGNRWAKIAHLLPGRTDNAIKNHWNTTLKRKR
Solanum tuberosum v3.4	PGSC0003DMP400015671	St3R-MYB1	PIRRAKGGWTPEEDDTLKRAVSVYRGKCWKKIAEFFPDRSEVQCLHRWQKVINPELVKGPWTREEDDKIIELVAKYGPIKWSVIAKSLTGRIGKQCRERWHNHLNPNIKKDAWTL<br>EEERALIDAHRIHGNKWAEIAKVLPGRTDNGIKNHWNSSLKKKL
Solanum tuberosum v3.4	PGSC0003DMP400005488	St3R-MYB2	GPTKRSSQAGWTEEEDNLLTEVVERFKGRNWKKIGKTIYSVMLYTFQLAECLNGRTDVQCLHRWQKVLNPELVKGPWTKEEDDLIIELVEKHGCKKWSNIANSLPGRIGKQCRE<br>RWHNHLDPTITKDAWTEQEELVLCHYHQLYGNKWAKIARFLPGRTDNAIKNHWNSTLKKRL
Solanum lycopersicum v2.3	Solyc01g005660.1	Sl2R-MYB001	KIGLKRGPWTPEEDELLMNYVSKEGEGRWRTLPKKAGLFRCGKSCRLRWMNYLRPSVKRGHILPEEEDLILRLHRLLGNRWSLIAGRIPGRTDNEIKNYWNTHLSKKL
Solanum lycopersicum v2.3	Solyc01g009070.2	Sl2R-MYB002	SVPLKKGPWTSAEDVILVDYVMTHGEGNWNAVQRHSGLARCGKSCRLRWANHLRPDLKKGAFTPEEEQRIVELHAKMGNKWARMAVELPGRTDNEIKNYWNTRIKRRQ
Solanum lycopersicum v2.3	Solyc01g010910.1	Sl2R-MYB003	KVGLKKGPWTPEEDQKLMDYIEKNGCGSWRALPTKAGLKRCGKSCRLRWINYLRPDIKRGKFSLQEEQTIIQLHALLGNRWSAIATHLANRTDNEIKNYWNTHLKKRL
Solanum lycopersicum v2.3	Solyc01g057910.2	Sl2R-MYB004	DMDLRRGPWTVEEDLTLMNYIAHHGEGRWNTLARCAGLKRTGKSCRLRWLNYLRPDVRRGNITLEEQLLILELHSRWGNRWSKIAQHLPGRTDNEIKNYWRTRVQKHA
Solanum lycopersicum v2.3	Solyc01g079620.2	Sl2R-MYB005	KVGIKRGRWTAEEDQILTNYIISNGEGSWRSLPKNAGLLRCGKSCRLRWINYLRSDLKRGNITSQEEDIIIKLHATLGNRWSLIAEHLSGRTDNEIKNYWNSHLSRKV
Solanum lycopersicum v2.3	Solyc01g087130.2	Sl2R-MYB006	LGKLRKGLWSPEEDEKLMSYMLRNGQGCWSDIARNAGLQRCGKSCRLRWINYLRPDLKRGAFSLQEEELIVHLHSILGNRWSQIAARLPGRTDNEIKNFWNSTIKKRS
Solanum lycopersicum v2.3	Solyc01g090530.1	Sl2R-MYB007	MSEIKKGPWKEEEDQVLIKHVKKYGPRDWSSIRSKGLLQRTGKSCRLRWVNKLRPNLKNGVKFSAEEERTVIELQAQFGNKWARIATYMPGRTDNDVKNFWSSRQKRLA
Solanum lycopersicum v2.3	Solyc01g094360.2	Sl2R-MYB008	KNGLTRGPWTPEEDEKLVQFINKNGHGSWRSLPKLAGLLRCGKSCRLRWTNYLRPDIIRGPFSPEEQKLVIQLHGILGNRWAAIASQLPGRTDNEIKNLWNTHLKKRL
Solanum lycopersicum v2.3	Solyc01g102340.2	Sl2R-MYB009	KQKLRKGLWSPEEDEKLIKHITKFGHGCWSSVPKLAGLQRCGKSCRLRWINYLRPDLKRGTFSQDEENLIIELHAVLGNKWSQIAARLPGRTDNEIKNLWNSSIKKKL
Solanum lycopersicum v2.3	Solyc01g111500.2	Sl2R-MYB010	KAHTNKGAWTKEEDERLISYIKTHGEGCWRSLPKAAGLLRCGKSCRLRWINYLRPDLKRGNFTEEEDELIIKLHSLLGNKWSLIAGRLPGRTDNEIKNYWNTHIRRKL
Solanum lycopersicum v2.3	Solyc02g067340.2	Sl2R-MYB011	KMGVKKGPWTPEEDIMLVSYVQQHGPGNWRALPTKTGLRRCSKSCRLRWTNYLRPGIKRGNFTDQEEKMIIQLQALLGNKWAAIASYLPERTDNDIKNYWNTHLKKKM
Solanum lycopersicum v2.3	Solyc02g067760.2	Sl2R-MYB012	DVEVRKGPWTMEEDLILINYIANHGEGVWNSLAKSAGLKRTGKSCRLRWLNYLRPDVRRGNITPEEQLLIMELHAKWGNKWSKIAKHLPGRTDNEIKNYWRTRIQKHI
Solanum lycopersicum v2.3	Solyc02g079280.2	Sl2R-MYB013	KNGLKKGPWTPEEDQKLIDYIQKHGYGNWRTLPKNAGLQRCGKSCRLRWTNYLRPDIKRGRFSFEEETIIQLHSILGNKWSAIAARLPGRTDNEIKNYWNTHIRKRL
Solanum lycopersicum v2.3	Solyc02g082040.2	Sl2R-MYB014	KQKVKRGLWSPEEDEKLIKHIKTHGHSCWSSVPKLAGLQRCGKSCRLRWINYLRPDLKRGCFSEQEERIIIDVHRILGNRWAQIAKHLPGRTDNEVKNFWNSCIKKKL
Solanum lycopersicum v2.3	Solyc02g087960.2	Sl2R-MYB015	KTGVKKGPWTPEEDIMLVSFVQEHGPGNWRTVPTHTGLRRCSKSCRLRWTNYLRPGIKRGSFSDQEEKMIIQLQALLGNKWAAIASYLPERTDNDIKNYWNTHLKKKL
Solanum lycopersicum v2.3	Solyc02g088190.2	Sl2R-MYB016	KVGLKKGPWTPEEDQKLLAYIEEHGHGSWRALPTKAGLQRCGKSCRLRWTNYLRPDIKRGKFTLQEEQTIIQLHALLGNRWSAIATHLSKRTDNEIKNYWNTHLKKRL
Solanum lycopersicum v2.3	Solyc02g089190.1	Sl2R-MYB017	EQGWRKGPWTPEEDKLLSEYVNLHGEGRWSSVSRCAGLNRTGKSCRLRWVNYLRPGLKRGHITPQEEGIIIELHALWGNKWSTIARYLPGRTDNEIKNYWRTHFKTKV
Solanum lycopersicum v2.3	Solyc02g091980.1	Sl2R-MYB018	KANVKRGPWSPEEDSKLKAYIEQHGTGGNWITLPQKVGLKRCGKSCRLRWLNYLRPNIKHGEFTDEEDNIICTLYMSIGSRWSIIAAQLPGRTDNDIKNYWNTRLKKKL
Solanum lycopersicum v2.3	Solyc02g092930.1	Sl2R-MYB019	MDRVKGPWSPEEDELLQQLVQKHGPRNWSLISKSIPGRSGKSCRLRWCNQLSPQVEHRAFTPEEDETIIRAHARFGNKWATIARLLNGRTDNAIKNHWNSTLKRKC
Solanum lycopersicum v2.3	Solyc02g093740.2	Sl2R-MYB020	RVGLKRGPWTIEEDHKLVHFILNNGIQCWRTVPKLAGLQRCGKSCRLRWINYLRPDLKRGALSEAEEDQIIELHARLGNRWSKIASYFPGRTDNEIKNHWNTRIKKRL
Solanum lycopersicum v2.3	Solyc03g005570.2	Sl2R-MYB021	KTSLKKGSWDHEEDQILISYINKNGHGNWRALPRQAGLLRCGKSCRLRWMNYLSPHIKRGNFTKEEEDNIINLHEILGNRWSAIAARLPGRTDNEIKNIWHTHLKKRL
Solanum lycopersicum v2.3	Solyc03g025870.2	Sl2R-MYB022	KQKHKKGLWSPDEDDRLKNYMIKHGHGCWSSVPINAGLQRNGKSCRLRWINYLRPGLKRGAFSLEEEDIILTLHAMFGNKWSQIAQQLPGRTDNEIKNHWHSYLKKRV
Solanum lycopersicum v2.3	Solyc03g059200.1	Sl2R-MYB023	KANVKRGPWTAEEDAKILAYVATHGIGNWTLVPQKAGLNRCGKSCRLRWTNYLRPDLKHDNFTPQEEDCILELHKTIGSRWSLIAKQLPGRTDNDVKNYWNTKLKKKL
Solanum lycopersicum v2.3	Solyc03g093890.2	Sl2R-MYB024	EAIIKKGAWSPEEDQKLRGYIMKYGIWNWRQMPKFAGLSRTGKSCRLRWMNYLRPDVKRGPFTTEEVEIVIKTYQELGNSWSAIAAKLPGRTDNEVKNFFHTHLKKHL
Solanum lycopersicum v2.3	Solyc03g093930.2	Sl2R-MYB025	STNMKKGAWSPEEDQKLKSYIMKFGIWNWSHMPKFAGLSRTGKSCRLRWMNYLRPDVKRGPFTMEEVEIVIKTYQELGNRWSAIAGRLPGRTDNEIKNFFHTHLKKQL
Solanum lycopersicum v2.3	Solyc03g093940.1	Sl2R-MYB026	MEDIIIIKKGAWSPEEDQKLKDYVMRFGIWNWNLMPKFAGLSRTGKSCRLRWMNYLRPDVKRGPFSMEERERVIKMYQQLGNRWAAIAGELPGRTDNEVKNFFHTHLKKNL
Solanum lycopersicum v2.3	Solyc03g112390.2	Sl2R-MYB027	KQKLRKGLWSPEEDEKLSNYITNFGIGSWSSVPKLAGLQRCGKSCRLRWINYLRPDLKRGMFSQDEEDKIISLHQVLGNRWAQIAAQLPGRTDNEIKNFWNSSLKKKL
Solanum lycopersicum v2.3	Solyc03g112890.1	Sl2R-MYB028	KSKSVKGQWTIEEDRILINLVERFGDRKWSQIAQVLKGRIGKQCRERWHNHLRPDIKKDLWTEEEDMILIKAHSEIGNKWAEIAKKLAGRTENSIKNHWNATKRRQF
Solanum lycopersicum v2.3	Solyc03g116100.2	Sl2R-MYB029	KIGVKKGPWTPEEDIILVSYIQQHGPGNWRAVPSNTGLLRCSKSCRLRWTNYLRPGIKRGNFTEHEEKMIIHLQALLGNRWAAIASYLPQRTDNDIKNYWNTHLRKKL

	Solanum lycopersicum v2.3	Solyc03g119370.1	Sl2R-MYB030	DIELRRGPWTIEEDKLLVHYITNHGEGRWNMLAKHAGLKRTGKSCRLRWLNYLKPDVKRGNLTPQEQLLILELHSKLGNRWSKIAQYLPGRTDNEIKNYWRTRVQKQA
	Solanum lycopersicum v2.3	Solyc03g121740.1	Sl2R-MYB031	KQKVKRGLWSPEEDEKLINYITTYGHACWSSVPKLAGLQRCGKSCRLRWINYLRPDLKRGSFSPQEAALIIELHGILGNRWAQIAKHLPGRTDNEVKNFWNSSIKKKL
	Solanum lycopersicum v2.3	Solyc04g005600.1	Sl2R-MYB032	KDGLKKGPWTHDEDQKLLAYVDEHGYGSWSDLPLRAGLQRCGRSCRLRWINYLRPNIKRGKFSSEEERTIFQLHALLGNRWSIIASHLPNRSDNEIKNYWNTRLKKRL
	Solanum lycopersicum v2.3	Solyc04g005710.1	Sl2R-MYB033	PKERHIVSWSQEEDDILREQIRIHGIDDNWTIIASNFKDKTTRQCKRRWFTYLNSDFKKGGWSPEEDMLLCAAQKIFGNRWTEIAKVVSGRTDNAVKNRFTTICKKKA
	Solanum lycopersicum v2.3	Solyc04g009950.2	Sl2R-MYB034	RIMIKGGVWKNTEDEILKAAVMKYGKNQWARISSLLVRKSAKQCKARWYEWLDPSIKKTEWTREEDEKLLHLAKLMPTQWRTIAPIVGRTPSQCLERYEKLLDAAC
	Solanum lycopersicum v2.3	Solyc04g014470.2	Sl2R-MYB035	KNEVKKGPWSSEEDKKLTNFILNNGQCCWRSLPKLAGLLRCGKSCRLRWTNYLRPDLKRGLLSEYEEKMVIDLHAQLGNRWSKIASHLPGRTDNEIKNHWNTHIKKKL
	Solanum lycopersicum v2.3	Solyc04g056310.2	Sl2R-MYB036	MGRSPFCDEIGLKKGPWTPEEDQKLINHIKKHGHGSWRALPKLAGLNRCGKSCRLRWTNYLRPDIKRGKFSQEEEQTILNLHAILGNKWSAIATHLPGRTDNEIKNFWNTHLKKK L
	Solanum lycopersicum v2.3	Solyc04g064540.2	Sl2R-MYB037	MAEGGGSGDDTTRSSCPRGHWRPAEDEKLRQLVEQYGPQNWNSIAEKLQGRSGKSCRLRWFNQLDPRINRRPFSEDEEEKLIGAHRIHGNKWALISRLFPGRTDNAVKNHWHVL MARKQ
	Solanum lycopersicum v2.3	Solyc04g074170.1	Sl2R-MYB038	ENGLKKGPWTPEEDQILVDYIDKHGHGSWRALPKLAGLNRCGKSCRLRWTNYLRPDIKRGKFSEEEEQTILHLHSILGNKWSAIATHLSGRTDNEIKNFWNTHLKKKLI
	Solanum lycopersicum v2.3	Solyc04g077260.2	Sl2R-MYB039	KNNVKRGPWSPEEDAKLKSYIEQNGTGGNWIALPQKIGLKRCGKSCRLRWLNYLRPNIKHGGFSEEEDRIICSLYISIGSRWSIIAAQLPGRTDNDIKNYWNTKLKKKL
	Solanum lycopersicum v2.3	Solyc04g078420.1	Sl2R-MYB040	DSDRIKGPWSPEEDELLQTLVEKHGPRNWTLISKSVPGRSGKSCRLRWCNQLSPQVEHRAFTPEEDDTIIRAHAKFGNKWATIARLLSGRTDNAIKNHWNSTLKRKC
	Solanum lycopersicum v2.3	Solyc04g079360.1	Sl2R-MYB041	KSERIKGPWSAEEDKILTKLVERYGARNWSLISKYIKGRSGKSCRLRWCNQLSPNVEHRPFSPAEDEAILAAHAKYGNRWATIARLLPGRTDNAVKNHWNSTLKRRY
	Solanum lycopersicum v2.3	Solyc05g007160.2	Sl2R-MYB042	PKERHIVSWSQEEDDILREQIRIHGTDNWTIIASKFKDKTTRQCRRRWFTYLNSDFKKGGWSPEEDMLLCEAQKIFGNRWTEIAKVVSGRTDNAVKNRFTTLCKKRA
	Solanum lycopersicum v2.3	Solyc05g007690.1	Sl2R-MYB043	EEGLKKGPWTHEEDQKLLSFIDKHGCGSWRGLPAKAGLQRCGKSCRLRWINYLRPDIKRGKFSLQEERTIIHLHALLGNRWSAIATYLPSRTDNEIKNYWNSRLKKRL
	Solanum lycopersicum v2.3	Solyc05g007710.2	Sl2R-MYB044	KEGLKKGPWTPEEDQKLLSFIDTYGCGSWRALPAKAGLQRCGKSCRLRWINYLRPDIKRGKFSLQEERTIIQLHALLGNRWSAIATYLPSRTDNEIKNYWNSRLKKRL
	Solanum lycopersicum v2.3	Solyc05g007870.1	Sl2R-MYB045	SKLCARGHWRPAEDAKLKELVAIYGPQNWNLIAEKLEGRSGKSCRLRWFNQLDPRINRRAFSEEEEERLMAAHRLYGNKWAMIARLFPGRTDNAVKNHWHVIMARKY
	Solanum lycopersicum v2.3	Solyc05g008250.1	Sl2R-MYB046	NKEEMHKGAWSKQEDQKLIDYITKHGEGCWRNLPKAAGLLRCGKSCRLRWMNYLNPNLKRGNFSEDEDDLIIKLHALLGNRWSLIAGRLPGRTDNEVKNYWNSHLTRKL
	Solanum lycopersicum v2.3	Solyc05g009230.1	Sl2R-MYB047	LFELRRGPWTLEEDNLLIHYISTHGEGRWNALAKCAGLKRTGKSCRLRWLNYLKPDIKRGNLTPQEQLLILELHSKWGNRWSKIAQHLPGRTDNEIKNYWRTRVQKQA
	Solanum lycopersicum v2.3	Solyc05g014290.2	Sl2R-MYB048	KIGLKRGPWTIEEDHKLMNFILNNGIQCWRLVPKLAGLMRCGKSCRLRWINYLRPDLKRGALTEAEEDMIIKLHSQLGNRWSKIAGHFPGRTDNEIKNHWNTRIKKKL
	Solanum lycopersicum v2.3	Solyc05g048830.2	Sl2R-MYB049	KKGLKKGPWTPEEDEKLVEYIKNHGHGSWRSLPHLAGLARCGKSCRLRWTNYLRPDIKRGPFSHDEEKLVIQLHGILGNRWAAIASQLPGRTDNEIKNLWNTHLKKRL
	Solanum lycopersicum v2.3	Solyc05g051550.1	Sl2R-MYB050	ENGLKKGPWTPEEDQKLTNHINKHGHGSWRALPKLAGLNRCGKSCRLRWSNYLRPDIKRGKFSQEEEQTILNLHAVLGNKWSAIATHLPGRTDNEIKNFWNTHLKKKL
	Solanum lycopersicum v2.3	Solyc05g052850.2	Sl2R-MYB051	SSGRVKEPWSPEEDAVLTELVNKFGARNWSMIAQGIAGRSGKSCRLRWCNQLDPVLKRKPFTDEEDHIIINAHGVHGNKWAAIAKLLPGRTDNAIKNHWNSTLRRRF
	Solanum lycopersicum v2.3	Solyc05g053150.1	Sl2R-MYB052	EEEWRKGPWTVEEDKLLIDYVNLHGEGRWNCVARLAGLKRNGKSCRLRWVNYLRPDLKRGQITPYEERIILELHAIWGNRWSTIARNLPGRTDNEIKNYWRTHFKKKV
	Solanum lycopersicum v2.3	Solyc05g053330.2	Sl2R-MYB053	VMDLRRGPWTVEEDFTLINFIAHHGEGRWNSLARCAGLKRTGKSCRLRWLNYLRPDVRRGNITVEEQLLILELHSRWGNRWSKIAQHLPGRTDNEIKNYWRTRVQKHA
	Solanum lycopersicum v2.3	Solyc05g055030.1	Sl2R-MYB054	KMGLKKGPWTQEEDQILINFIQKYGHENWRALPKQAGLLRCGKSCRLRWTNYLRPDIKRGNFSEEEEQIIIKLHQLLGNRWSAIASRLPGRTDNEIKNFWHTHLKKRL
	Solanum lycopersicum v2.3	Solyc06g005330.2	Sl2R-MYB055	QEAMRKGPWMEQEDLQLAFYVNLLGDRRWDFLAKVSGLRRTGKSCRLRWVNYLHPGLKRGKMTPQEERLILELHSKWGNRWSRIAREVSGRTDNEIKNYWRTHMRKKA
	Solanum lycopersicum v2.3	Solyc06g009410.1	Sl2R-MYB056	SMEMNKGPWSSDEDSKLIHSISIFGQGRWDSLAHVAGLKRSCKSCRLRWLNYLRPNLRRGKITPQEQVLILLLHFRFGNRYGNNYFIFFSKIKNRWSKIAENLPGRSYNDVKNYWR TKV
	Solanum lycopersicum v2.3	Solyc06g009480.1	Sl2R-MYB057	SMEMKKGPWSSDEDSKLIHSISIFGQGRWDSLAHVAGLKRSGKSCRLRWLNYLRPNLRRGKITPQEQLLILLLHFRFGNRWSKIAENLPGRSDNDVKNYWRTKVQKHA
	Solanum lycopersicum v2.3	Solyc06g009710.2	Sl2R-MYB058	KVGLKRGRWTSEEDEILTNYIQANGEGSWRSLPKNAGLLRCGKSCRLRWINYLKSDLKRGNITSDEEAIIIKLRATLGNRWSLIAEHLPGRTDNEIKNYWNSHLRRKV
	Solanum lycopersicum v2.3	Solyc06g033870.1	Sl2R-MYB059	MGYVKKGAWSPQEDKKLIDHIAKYRIWNWSQMPKFAGLSRTGKSCRLRWINYLRPDIKRGTFSLEETQTIIKMYQSLGNRRSAIAKELPGRTDNEIKNFYHTHLKKHV
	Solanum lycopersicum v2.3	Solyc06g034000.1	Sl2R-MYB060	MDYVKKGAWSPDEDQKLVDYIMKYRIWNWSHMPKFAGLSRTGKSCRLRWINYLRPDLKKGPFSIEEVEIVIRMYQSLGNRWSAIAKELPGRTDNEIKNFYHTHLKKHL
	Solanum lycopersicum v2.3	Solyc06g053610.2	Sl2R-MYB061	KMGLKKGPWTPQEDNILISYIQNNGHSNWRALPKLAGLLRCGKSCRLRWTNYLRPDIKRGNFTKEEEDTIIKLHENLGNRWSAIAARLPGRTDNEIKNIWHTNLKKKL
	Solanum lycopersicum v2.3	Solyc06g065100.2	Sl2R-MYB062	KAHTNKGAWTKEEDQRLINYIRAHGEGCWRSLPKAAGLSRCGKSCRLRWINYLRPDLKRGNFTEEEDELIIKLHSLLGNKWSVIAGRLPGRTDNEIKNYWNTHIKRKL
	Solanum lycopersicum v2.3	Solyc06g069850.2	Sl2R-MYB063	KSGVKKGPWTPEEDIILVSYIQEHGPGNWRNIPTNTGLLRCSKSCRLRWTNYLRPGIKRGNFTEHEEKMIIHLQALLGNRWAAIASYLPQRTDNDIKNYWNTHLKKKL
	Solanum lycopersicum v2.3	Solyc06g071520.1	Sl2R-MYB064	KSKSSKGQWTIEEDRLLIHLVDKFGVRKWSQIAQMLKGRIGKQCRERWHNHLRPDIKKDFWTEEEDRILIQAHGEVGNKWAEIAKRLPGRTENSIKNHWNATKRRQF
	Solanum lycopersicum v2.3	Solyc06g071690.2	Sl2R-MYB065	KEKTRKGLWSPEEDEKLYNYITRFGVGCWSSVPKLAGLQRCGKSCRLRWINYLRPDLKRGMFSQEEEDMIITLHKVLGNRWAQIAAKLPGRTDNEIKNFWNSNLKRKL
	Solanum lycopersicum v2.3	Solyc06g073640.2	Sl2R-MYB066	GLPLKKGPWTSAEDAILVEYVTKHGEGNWNAVQKHSGLARCGKSCRLRWANHLRPDLKKGAFTPEEERHIIELHAKMGNKWARMAAELPGRTDNEIKNYWNTRIKRRQ
	Solanum lycopersicum v2.3	Solyc06g074910.2	Sl2R-MYB067	KNNVKRGPWSPEEDAKLKEFIEKYGTGGNWIALPLKAGLKRCGKSCRLRWLNYLRPNIKHGDFSDEEDRVICSLYASIGSRWSIIAAQLPGRTDNDIKNYWNTKLKKKL
	Solanum lycopersicum v2.3	Solyc06g074920.1	Sl2R-MYB068	KINVKKGPWSPEEDAKLKEFIEKYGNGGNWIALPQKVGLMRCGKSCRLRWLNYLRPNIRHGNFSQEEDRVTCSLYARIGSRWSFIAAQLSERTDNNIKNYWNTKLKKKF
	Solanum lycopersicum v2.3	Solyc06g076840.1	Sl2R-MYB069	TNYLIKGQWTDEEDRKLLRLVKQFGVRKWAQIAEKMEARAGKQCRERWHNHLRPDIKKDTWSEEEELLLVESHKQLGNKWAEIAKRIPGRTENAIKNHWNATKRRQN
	Solanum lycopersicum v2.3	Solyc06g083900.2	Sl2R-MYB070	KLGLKRGPWSKEEDYLLINYIKKNGHPNWRALPKLAGLLRCGKSCRLRWTNYLRPDIKRGNFTHQEEDTIIKLHQVLGNSWSAIAARLPGRTDNEIKNIWHTRLKKKR
	Solanum lycopersicum v2.3	Solyc07g006750.2	Sl2R-MYB071	KANVKKGPWSPEEDATLKAYIEENGTGNNWIALPQKIGLKRCGKSCRLRWLNYLRPNIKHGGFTEEEDNIICSLYISIGSRWSIIAAQLPGRTDNDIKNYWNTRLKKKL
	Solanum lycopersicum v2.3	Solyc07g008010.2	Sl2R-MYB072	MKDKDVKTRMKRGFWKPEEDLILKNCVETHGEGNWATISEKSGLMRSGKSCRLRWKNYLRPNIKRGMMSEDEKDLIIRLHKLLGNRWSLIAGRLPGRTDNEVKNFWNTHLNNK R
	Solanum lycopersicum v2.3	Solyc07g052300.2	Sl2R-MYB073	RQALKKGPWTAGEDAILMDYVKKHGEGNWNAVQRNSGLMRCGKSCRLRWANHLRPHLKKGAFTPEEERIIIDLHAKLGNKWARMAAQLPGRTDNEIKNYWNTRLKKRRQ

	Solanum lycopersicum v2.3	Solyc07g053230.2	Sl2R-MYB074	ENGRKKGTWTPEEDRKLAAYITKYGSWNWRQLPKYAGLARCGKSCRLRWMNYLRPNVKRGNYTKEEDEIILNLHAQLGNKWSAIAIHLPGRSDNEIKNHWHTTLKKRA
	Solanum lycopersicum v2.3	Solyc07g053240.2	Sl2R-MYB075	EHGRKKGTWTPEEDRKLAAYITKYGSWNWRQLPKYAGLARCGKSCRLRWMNYLRPNVKRGNYTKEEDEIILNLHAQLGNKWSTIASHLPGRSDNEIKNHWHTTVKKRA
	Solanum lycopersicum v2.3	Solyc07g054840.2	Sl2R-MYB076	KNGLKKGPWTPEEDNKLIQYIQLHGPGNWRTLPKNAGLQRCGKSCRLRWTNYLRPDIKRGRFSFDEEETIIQLHSVLGNKWSAIAARLPGRTDNEIKNYWNTHIRKRL
	Solanum lycopersicum v2.3	Solyc07g054960.1	Sl2R-MYB077	KNGIKRGAWSEDEDNKLRAFIQKFGHPNWRQLPKYAGLMRCGKSCRLRWMNYLRPGLKKGNYSHEEEQLIIKLHNKLGNRWSEIAAKLPGRSDNDVKNHWHAHLKKRP
	Solanum lycopersicum v2.3	Solyc07g054980.1	Sl2R-MYB078	KNGRKKGTWTPEEDKKLEAYITKYGCWNWRQLPKYAGLARCGKSCRLRWMNHLRPNVKRGNYTKEEDELILNLHAQLGNRWSAIAIHLPGRSDNEIKNHWHTSLKKRA
	Solanum lycopersicum v2.3	Solyc07g055000.1	Sl2R-MYB079	ENGRKKGSWTLEEDKKLAAYITKYGSWNWRQLPKYAGLARCGKSCRLRWINHLRPNVKRGNYTKEDEIILNIHAQLGNKWSTIAAHLPERSDNEIKNYWHTTLKKRA
	Solanum lycopersicum v2.3	Solyc08g005870.1	Sl2R-MYB080	KEGLKKGSWTKKEDLLLTEYIKEYGEGTGWRSLPKKAGLLRCGKSCRLRWVNYLKPGLNRGTFTPEEDDLIVRLYTLLGSRWSLIAGRIPGRTDNDIKNYWNTSLLKKL
	Solanum lycopersicum v2.3	Solyc08g008480.2	Sl2R-MYB081	KEGLRKGPWSTNEDLLLTNYIKENGEGQWRNLPNKAGLLRCGKSCRLRWMNYLRPGIKRGNFSQDEEDLIIRLHSLLGNRWSLIAGRLPGRTDNEIKNYWNTHVIKKL
	Solanum lycopersicum v2.3	Solyc08g065910.1	Sl2R-MYB082	KSKVKRGQWSPEEDEILKNHIFNHGNPGSWIALPKRAGLNRCGKSCRLRWLNYLRPNIKLGNFTQDEDNTICSLYNQLGSRWSVIASKLPGRTDNEIKNHWNTKLKKKV
	Solanum lycopersicum v2.3	Solyc08g076700.1	Sl2R-MYB083	DEDLKKGPWTSEEDEKLVEYIKENGHGNWQLIPKRAGLNRCGKSCRLRWTNYLRPDIKRGAFSEEEEEIIINLHSLLGNKWSKIASHLPGRTDNEIKNLWNTHLKKKL
	Solanum lycopersicum v2.3	Solyc08g076710.2	Sl2R-MYB084	DENLKKGPWTSEEDEKLMDYITKNGHTNWQLIPKKAGLNRCGKSCRLRWTNYLRPDIKRGEFSIEEEEIIINLHSLLGNKWSKIAGHLPGRTDNEIKNFYNTHLRKKL
	Solanum lycopersicum v2.3	Solyc08g079270.2	Sl2R-MYB085	KLGVKKGPWTAEEDKKLINFILTNGQCCWRAVPKLAGLRRCGKSCRLRWTNYLRPDLKRGLLSEAEEKLVIDLHARLGNRWSKIAARLPGRTDNEIKNHWNTHIKKKL
	Solanum lycopersicum v2.3	Solyc08g081500.1	Sl2R-MYB086	QQKVKRGLWSPEEDEKLIRYITSHGYGCWSEVPEKAGLQRCGKSCRLRWINYLRPDIRRGRFTPEEEKLIISLHGAVGNRWAHIASHLPGRTDNEIKNYWNSWIKKKL
	Solanum lycopersicum v2.3	Solyc08g082890.2	Sl2R-MYB087	KEGLRKGPWSTKEDLLLTNYINQYGEGQWRSLPKNAGLLRCGKSCRLRWVNYLKPGIKRGNFSEDEEDLIVRLHSLLGNRWSLIAGRLPGRTDNEIKNYWNTHLTKKL
	Solanum lycopersicum v2.3	Solyc09g008250.2	Sl2R-MYB088	KANVKKGPWSPEEDAKLKEYIDKFGTGGNWIALPQKAGLRRCGKSCRLRWLNYLRPNIKHGEFSDEEDRIICSLYANIGSRWSIIAAQLPGRTDNDIKNYWNTKLKKKL
	Solanum lycopersicum v2.3	Solyc09g008390.1	Sl2R-MYB089	EEKLRKGPWLDEEDERLAYVIAILGERRWDALAKASGLRRSGKSCRLRWMNYLRPNLKHGHITQDEEHLIIKLQKQLGNKWSKIAKQLPGRTDNEIKNYWRSHLRKKT
	Solanum lycopersicum v2.3	Solyc09g010840.1	Sl2R-MYB090	MRERQRWRAEEDALLRAYVRQYGPKEWHLVSQRMNTPLNRDAKSCLERWKNYLKPGIKKGSLTEDEQRLVIQLQAKHGNKWKKIAAEVPGRTAKRLGKWWEVFKEKQQ
	Solanum lycopersicum v2.3	Solyc09g011780.2	Sl2R-MYB091	TADRVKGPWSPEEDAILSRLVSNFGARNWSLIARGIPGRSGKSCRLRWCNQLDPAVKRKPFTDEEDRIILQAHAIHGNKWASIARLLPGRTDNAIKNHWNSTLRRRC
	Solanum lycopersicum v2.3	Solyc09g055650.1	Sl2R-MYB092	VTMLVKGQWTEEEDRILMKLVKQFGLKRWAQIAENMVGRAGKQCRERWHNHLRPDIKKETWSEEEEVMLVEAHKQIGNKWAEIGKRIPGRTENAIKNHWNATKRR
	Solanum lycopersicum v2.3	Solyc09g090130.2	Sl2R-MYB093	KMGLKKGPWTPEEDQILISYIQSNGHGNWRALPKLAGLLRCGKSCRLRWTNYLRPDIKRGNFTREEEDSIIQLHEMLGNRWSAIAARLPGRTDNEIKNVWHTHLKKRL
	Solanum lycopersicum v2.3	Solyc09g090790.2	Sl2R-MYB094	EVGWRKGPWTSDEDRLLIEYVKLHGEGRWNSVARLTGLRRNGKSCRLRWVNYLRPDLKRGQITPHEERIILELHSRWGNRWSTIARSLPGRTDNEIKNYWRTHFKKKT
	Solanum lycopersicum v2.3	Solyc10g005240.2	Sl2R-MYB095	KQKVKRGLWSPEEDEKLMRHISTQGHGCWSSVPRLAGLQRCGKSCRLRWINYLRPDLRRGSFSEQEERTIIDIHRILGNRWAQIAKHLPGRTDNEVKNFWNSCIKKKL
	Solanum lycopersicum v2.3	Solyc10g005460.2	Sl2R-MYB096	KNGLKKGPWTTEEDQKLIDYIQKYGSGNWRILPKNAGLQRCGKSCRLRWINYLRPDIKRGKFSFEEEETIIHLHSILGNKWSAIAARLPGRTDNEIKNYWNTNIRKKL
	Solanum lycopersicum v2.3	Solyc10g005550.1	Sl2R-MYB097	KSKVKKGPWTPSEDLKLISFIQKHGHGNWRALPKQAGLLRCGKSCRLRWINYLRPDVKRGNFTPQEEDTIINLHRAFGNRWSKIASHLPGRTDNEIKNVWNTHLKKRL
	Solanum lycopersicum v2.3	Solyc10g005760.1	Sl2R-MYB098	KDNVKRGQWTPEEDHKLSSYIAQHGTRNWRLIPKHAGLQRCGKSCRLRWTNYLRPDLKHGQFSEAEEQTIVTLHSVLGNRWSVIAAQLPGRTDNDVKNHWNTKLKKKL
	Solanum lycopersicum v2.3	Solyc10g008700.1	Sl2R-MYB099	KNGLKKGPWTPEEDQKLIQYIQLHGPGNWRNLPKNAGLQRCGKSCRLRWTNYLRPDIRRGRFSFEEEETIIQLHSVLGNKWSAIAARLPGRTDNEIKNYWNTHIRKRL
	Solanum lycopersicum v2.3	Solyc10g019260.1	Sl2R-MYB100	FDEIKKGSWKEEEDEVLINHVKKYGPRDWSSIRSKGLLQRTGKSCRLRWVNKLRPNLKNGVKFSGEEERTVIELQGQFGNKWARIATYLSGRTDNDVKNFWSSRQKRLA
	Solanum lycopersicum v2.3	Solyc10g044680.1	Sl2R-MYB101	KKKLRKGLWCPEEDEKLINHVTKYGHGCWSSVPKLAALQRCGKSCRLRWINYLRPDLKRGTFSQQEENLIIQLHSLLGNKWSQIASRLPGRTDNEIKNLWNSSIKKKL
	Solanum lycopersicum v2.3	Solyc10g055410.1	Sl2R-MYB102	KAHTNKGAWTKEEDERLISYIRAHGEGCWRSLPKAAGLLRCGKSCRLRWINYLRPDLKRGNFTEEEDELIIKLHSLLGNKWSLIAGRLPGRTDNEIKNYWNTHIRRKL
	Solanum lycopersicum v2.3	Solyc10g081320.1	Sl2R-MYB103	TKLCARGHWRPHEDAKLKELVGQYGPQNWNLIAEKLEGRSGKSCRLRWFNQLDPRINRKAFSEEEEERLLSAHKMYGNKWSMIARLFPGRTDNAVKNHWHVIMARKH
	Solanum lycopersicum v2.3	Solyc10g081490.1	Sl2R-MYB104	KIGIKKGPWTPEEDIILVSYIQEHGPGNWRSVPTNTGLMRCSKSCRLRWTNYLRPGIKRGNFTPHEEGMIIHLQALLGNKWAAIASYLPQRTDNDIKNYWNTHLKKKL
	Solanum lycopersicum v2.3	Solyc10g083900.1	Sl2R-MYB105	EEELRRGQWLEEEDERLSMMVAVFGERRWDALAKTSGLKRSGKSCRLRWLNYLRPNLKHGHITADEERLIIRLQKQFGNKWSKIAKQLPGRTDNEIKNYWRSHLRKKT
	Solanum lycopersicum v2.3	Solyc10g086250.1	Sl2R-MYB106	SLGVRKGSWTEQEDSLLRDCIQKYGEGKWHLVPARAGLNRCRKSCRLRWLNYLRPHIKRGDFAPDEVDLILRLHKLLGNRWSLIAGRLPGRTANDVKNYWNTHFHKKLS
	Solanum lycopersicum v2.3	Solyc10g086260.1	Sl2R-MYB107	SLGVRKGSWTDEEDFLLRKCIDKYGEGKWHLVPIRAGLNRCRKSCRLRWLNYLRPHIKRGDFEQDEVDLILRLHKLLGNRWSLIAGRLPGRTANDVKNYWNTNLLRKL
	Solanum lycopersicum v2.3	Solyc10g086270.1	Sl2R-MYB108	SLGVRKGSWTEQEDLLLRKCINKYGEGKWHLVPIRAGLNRCRKSCRLRWLNYLRPHIKRGDFASDEIDLILRLHKLLGNRWSLIAGRLPGRTANDVKNYWNTNLLRKV
	Solanum lycopersicum v2.3	Solyc10g086290.1	Sl2R-MYB109	TLGVRKGSWTEDEDILLRKCIDKYGEGKWHLVPFRAGLNRCRKSCRLRWLNYLRPHIKRGDFALDEIDLILRLHKLLGNRWSLIAGRLPGRTANDVKNYWNTHLHKKL
	Solanum lycopersicum v2.3	Solyc11g011050.1	Sl2R-MYB110	DKVGLKKGPWTAEEDKKLINFILNNGQCCWRAVPKLAGLLRCGKSCRLRWTNYLRPDLKRGLLSEYEEKMVIDLHAQLGNRWSKIASHLPGRTDNEIKNHWNTHIKKKL
	Solanum lycopersicum v2.3	Solyc11g065840.1	Sl2R-MYB111	NVKLRKGLWSPEEDEKLMHYMLTNGQGCWSDVARNAGLQRCGKSCRLRWINYLRPDLKRGAFSPQEEEHIIHLHSILGNRWSQIAARLPGRTDNEIKNFWNSTLKKRL
	Solanum lycopersicum v2.3	Solyc11g069030.1	Sl2R-MYB112	KANVKRGPWSPEEDAKLKDFIHKFGTAGNWIALPQKAGLRRCGKSCRLRWLNYLRPNIKHGDFSDDEDRVICNLYANIGSRWSIIAAQLPGRTDNDIKNYWNTKLKKKL
	Solanum lycopersicum v2.3	Solyc11g072060.1	Sl2R-MYB113	GTPLKKGPWLKAEDAILRDYVAEYGEGNWSSVHRRTGLARCGKSCRLRWSNHLKPYLKKGAFTQEEEQLIVAMHAQIGNKWARIASEFPGRTDNDIKNFWNTRLKRQR
	Solanum lycopersicum v2.3	Solyc11g073120.1	Sl2R-MYB114	EEIMRRGPWTEEEDVQLLFYVKLFGDRRWDFLAKVSGLKRTGKSCRLRWVNYLNPDLKHGKMTPQEERLVLELHSKWGNRWSRIARKIPGRTDNEIKNYWRTHMRKKA
	Solanum lycopersicum v2.3	Solyc12g005640.1	Sl2R-MYB115	KSGLKKGTWTLEEDKKLAAYVNKYGCWNWRQLPKFAGLSRCGKSCRLRWLNYLRPNIKRGNYTREEDQIIMKLHAEIGNKWSAIAIHLPGRSDNEIKNHWHTSLKKRS
	Solanum lycopersicum v2.3	Solyc12g008670.1	Sl2R-MYB116	KTKVKRGPWSPEEDNILKNYLEKNGTSGNWISLPQKAGLRRCGKSCRLRWLNYLRPDIKHGGFTEEEDNIILTLYRQIGSRWSVIAANLSGRTDNDVKNHWNTKLKKKH
	Solanum lycopersicum v2.3	Solyc12g049300.1	Sl2R-MYB117	KEGLNKGAWTPLEDKILMDYIKVHGEGKWRNLPKGAGLKRCGKSCRLRWLNYLRPDIKRGNISPDEEDLIIRLHKLLGNRWSLIAGRLPGRTDNEIKNYWNTNIGRRL
	Solanum lycopersicum v2.3	Solyc12g049350.1	Sl2R-MYB118	KVGLKRGRWTIEEDERLTNYIQANGEGSWRTLPKNAGLLRCGKSCRLRWINYLKSDLKRGNITSEEEAIIIKLRATLGNRWSLIAEYLPHRTDNEIKNYWNSRLCRKV

Solanum lycopersicum v2.3	Solyc12g096200.1	Sl2R-MYB119	KVGMKKGPWSIEEDMLLTSYIHLHGEGNWRSLPMNAGLLRCSKSCRLRWVNYLRPGIKRGNFSPEEDDLIIPLYSLLGGRWSLIAGRLSGRTDNEIKNHWHTNLLKKL
Solanum lycopersicum v2.3	Solyc12g099120.1	Sl2R-MYB120	IMELRRGPWTVEEDLVLMNYISHHGEGRWNSLSRCAGLKRTGKSCRLRWLNYLRPDVRHGNITLEEQLLILQLHSRWGNRWSKIAQHLPGRTDNEIKNYWRTRVQKHA
Solanum lycopersicum v2.3	Solyc12g099130.1	Sl2R-MYB121	IMELRRGPWTVEEDFILMNYISHHGEGRWNSLSRCAGLKRTGKSCRLRWLNYLRPNIRHGNITLEEQLLILQLHFRWGNRWSKIAEHLPGRTDNEIKNYWRTRVQKHA
Solanum lycopersicum v2.3	Solyc12g099140.1	Sl2R-MYB122	NLDLKRGSWTVEEDFTLMNHIALHGEGRWNSLARSAGLKRTGKSCRLRWLNYLRPDVRRGNITLEEQLLILQLHSRWGNRWSKIAQYLPGRTDNEIKNYWRTRVQKQA
Solanum lycopersicum v2.3	Solyc08g068320.2.1	Sl3R-MYB1	TRRSTKGQWTPEEDDTLCKAVQRFKGKNWKKIAECFKDRTDVQCLHRWQKVLNPELVKGPWSKEEDEVIGELVKKYGPKKWSTIAQHLPGRIGKQCRERWHNHLNPGINKEA WTQEEEMTLVHAHQIYGNKWAELTKFLPGRTDNAIKNHWNSSVKKKL
Solanum lycopersicum v2.3	Solyc11g071300.1.1	Sl3R-MYB2	GPKRRSSHWTPEEDEILRQAVQQFKGKSWKKIAECFKDRSDVQCLHRWQKVLDPELVKGSWSKEEDDKLIGLVNIHGPKKWSTIAQELAGRIGKQCRERWHNHLNPAIKKEAWT QEEELTLIRAHEAHGNKWAELSKYLPGRTDNAIKNHWHSSVKKKR
Solanum lycopersicum v2.3	Solyc08g080580.2.1	Sl3R-MYB3	GPTKRSSQAGWTEEEDNLLTEVVERFKGRNWKRIAECLNGRTDVQCLHRWQKVLNPELVKGPWTKEEDDLIIELVEKHGCKKWSSIANSLPGRIGKQCRERWHNHLDPTITKDA WTEQEEMVLCHYHQLYGNKWAKIAKFLPGRTDNAIKNHWNSTLKKRL
Solanum lycopersicum v2.3	Solyc09g010820.2.1	Sl3R-MYB4	PIRRAKGGWTPEEDDTLKQAVSVYRGKCWKKIAEFFPDRSEVQCLHRWQKVINPELVKGPWTREEDDKIIELVAKYGPIKWSVIAKSLTGRIGKQCRERWHNHLNPNIKKDAWTL EEERALIDAHRIHGNKWAEIAKVLPGRTDNGIKNHWNSSLKKKL
Vitis vinifera Genoscope.12X	GSVIVT01016800001	Vv2R-MYB001	KQKVKRGLWSPEEDEKLINYISSYGHGCWSSVPRLAGLQRCGKSCRLRWINYLRPDLKRGSFSPQEAALIIELHRILGNRWAQIAKHLPGRTDNEVKNFWNSSIKKKL
Vitis vinifera Genoscope.12X	GSVIVT01000232001	Vv2R-MYB002	KEHTNKGAWTKEEDQRLIAYIKTHGEGCWRSLPKAAGLLRCGKSCRLRWINYLRPDLKRGNFTDEEDELIINLHSFFGNKWSLIAGRLPGRTDNEIKNYWNTHIKRKL
Vitis vinifera Genoscope.12X	GSVIVT01000449001	Vv2R-MYB003	KSGLKKGTWTPEEDHKLVAYITRYGCWNWRRLPKYAGLMRCGKSCRLRWMNYLRPNIKRGNYSKEEEDTIIKLHASLGNRWSAIAAQLPGRTDNEIKNYWHTNLKKRL
Vitis vinifera Genoscope.12X	GSVIVT01000450001	Vv2R-MYB004	KYGRKKGAWSPEEDEKLRAYVQRYGHWNWRELPRFAGLSRCGKSCRLRWLNYLRPNVKRGNYSKAEEETIKKLHEELGNKWSAIAKKLPGRTDNEIKNYWHTHLKKRE
Vitis vinifera Genoscope.12X	GSVIVT01000460001	Vv2R-MYB005	KNGLKKGPWTPEEDDKLSHYIQLHGPGNWRTLPKNAGLQRCGKSCRLRWTNYLRPDIKRGRFSFEEEETIIQLHSILGNKWSAIAARLPGRTDNEIKNYWNTHIRKRL
Vitis vinifera Genoscope.12X	GSVIVT01003662001	Vv2R-MYB006	KNGLKKGPWTPEEDQKLIDYIQKNGYGNWRTLPKNAGLQRCGKSCRLRWTNYLRPDIKRGRFSFEEEETIIQLHSILGNKWSAIAARLPGRTDNEIKNYWNTHIRKRL
Vitis vinifera Genoscope.12X	GSVIVT01003675001	Vv2R-MYB007	VSTLKKGSWSPEEDQKLKAYIKRYGIWNWTEMPKAAGLSRSGKSCRLRWVNYLKPGIKRGSFSCEEEKTILEMHKKLGNRWSAIAERLPGRTDNEIKNYWHTKLRKLK
Vitis vinifera Genoscope.12X	GSVIVT01003966001	Vv2R-MYB008	KVGLKRGPWTIEEDHKLMSFILNNGIQCWRMVPKLAGLLRCGKSCRLRWINYLRPDLKRGALSEVEENQIIQLHSRLGNRWSKIASHFPGRTDNEIKNHWNTRIKKRL
Vitis vinifera Genoscope.12X	GSVIVT01004317001	Vv2R-MYB009	KLGVKKGPWTAEEDKKLINFILTNGQCCWRAVPKLAGLRRCGKSCRLRWTNYLRPDLKRGLLTESEEQLVIDLHARLGNRWSKIAARLPGRTDNEIKNHWNTHIKKKL
Vitis vinifera Genoscope.12X	GSVIVT01004464001	Vv2R-MYB010	KANVKKGPWSPEEDAKLKTYIEKNGTGGNWIALPQKIGLKRCGKSCRLRWLNYLRPNIKHGGFSEEEDNIICSLYISIGSRWSVIATQLPGRTDNDIKNYWNTRLKKKL
Vitis vinifera Genoscope.12X	GSVIVT01004851001	Vv2R-MYB011	ESGLKKGPWTPEEDQKLVKYIQKQGHGSWRALPKLAGLNRCGKSCRLRWTNYLRPDIKRGKFSREEEETILNLHSILGNKWSAIASHLPGRTDNEIKNFWNTHLKKKL
Vitis vinifera Genoscope.12X	GSVIVT01006275001	Vv2R-MYB012	KKVMNKGAWTSEEDRKLAEYIEVHGAKRWKTVAFKSGLNRCGKSCRLRWLNYLRPNIKRGNISDDEEDLILRLHKLLGNRWSLIAGRLPGRTDNEIKNYWNSHLSKKL
Vitis vinifera Genoscope.12X	GSVIVT01007670001	Vv2R-MYB013	KQKLRKGLWSPEEDEKLYNYITRFGVGCWSSVPKLAGLQRCGKSCRLRWINYLRPDLKRGMFSQQEEDIIISLHQVLGNRWAQIAAQLPGRTDNEIKNFWNSCLKKKL
Vitis vinifera Genoscope.12X	GSVIVT01007731001	Vv2R-MYB014	KTHVVKGQWTIEEDRLLIQLVEQYGVRKWSLIAQMLNGRIGKQCRERWHNHLRPNIKKDIWSEEEDKILIQAHAEIGNKWAEIAKRLPGRTENSIKNHWNATKRRQL
Vitis vinifera Genoscope.12X	GSVIVT01007740001	Vv2R-MYB015	ANGYRKGLWTVEEDRILMDYIRVHGKGKWNRIAKVTGLKRCGKSCRLRWMNYLSPSVKRGDFSQEEDDLIIRLHNLLGNRWSLIAGRVPGRTDNQVKNHWNTHLSKKL
Vitis vinifera Genoscope.12X	GSVIVT01007981001	Vv2R-MYB016	TTSLKRGPWTPEEDRKLLAYIQEHGHGSWRCVPENAGLQRCGKSCRLRWTNYLRPDIKRGKFSLREEQTIIQLHALLGNRWSAIATHLPNRTDNEIKNYWNTHLKKRL
Vitis vinifera Genoscope.12X	GSVIVT01008005001	Vv2R-MYB017	KIGVKKGPWTPEEDIILVSYIQEHGPGNWRAVPTSTGLLRCSKSCRLRWTNYLRPGIKRGNFTDQEEKTIIHLQALLGNRWAAIASYLPQRTDNDIKNYWNTHLKKKL
Vitis vinifera Genoscope.12X	GSVIVT01008090001	Vv2R-MYB018	KLNVKRGLWTAEEDAKILAHVSKHGTGNWTAVPKKAGLRRCGKSCRLRWTNYLRPDLKHESFTPQEEELIVRLHATIGSRWSIIAQQLPGRTDNDVKNYWNTKLRKKL
Vitis vinifera Genoscope.12X	GSVIVT01008303001	Vv2R-MYB019	DAELRRGPWTLEEDTLLIHYIASHGEGRWNLLAKCSGLRRTGKSCRLRWLNYLKPDVKRGNLTPQEQLLILELHSKWGNRWSKIAQHLPGRTDNEIKNYWRTRVQKQA
Vitis vinifera Genoscope.12X	GSVIVT01008401001	Vv2R-MYB020	KKFTNKGAWSKQEDQKLIDYIQKHGEGCWSSLPQSAGLLRCGKSCRLRWVNYLKPDVKRGNFGEDEEDLIIKLHALLGNRWSLIAGRLPGRTDNEVKNYWNSHLKKKL
Vitis vinifera Genoscope.12X	GSVIVT01008402001	Vv2R-MYB021	KKDTNKGAWSKEEDQKLIDYIQKHGEGSWRSLPQAAGLLRCGKSCRLRWLNYLRPDLKRGNFAEDEEDLIVKLHALLGNRWSLIAGRLPGRTDNEVKNYWNSHLKRKL
Vitis vinifera Genoscope.12X	GSVIVT01008484001	Vv2R-MYB022	MCTRGHWRPAEDEKLRELVERHGPHNWNAIAEKLPGRSGKSCRLRWFNQLDPRINRAPFTEEEEERLLASHRIHGNRWAVIARLFPGRTDNAVKNHWHVIMARRC
Vitis vinifera Genoscope.12X	GSVIVT01009032001	Vv2R-MYB023	ENGLKKGPWTPEEDQKLVSYVQKHGHGSWRALPKLAGLNRCGKSCRLRWTNYLRPDIKRGKFSQEEEQTILHLHSILGNKWSAIASHLPGRTDNEIKNFWNTHLKKKL
Vitis vinifera Genoscope.12X	GSVIVT01009280001	Vv2R-MYB024	KANVKKGPWSPEEDAKLKSYIEQHGTGGNWIALPQKIGLKRCGKSCRLRWLNYLRPNIRHGGFSEEEDNIICSLYISIGSRWSVIAAQLPGRTDNDIKNYWNTRLKKKL
Vitis vinifera Genoscope.12X	GSVIVT01009424001	Vv2R-MYB025	DLDRIKGPWSPEEDEALQRLVQKHGPRNWSLISKSIPGRSGKSCRLRWCNQLSPQVEHRAFTPEEDATIIRAHARFGNKWATIARLLVGRTDNAIKNHWNSTLKRKC
Vitis vinifera Genoscope.12X	GSVIVT01010006001	Vv2R-MYB026	AKTCPRGHWRPAEDEKLRQLVEEYGAQNWNSIAEKLQGRSGKSCRLRWFNQLDPRINRKPFTEEEEERLLAAHRIHGNKWALISRLFPGRTDNAVKNHWHVIMARKQ
Vitis vinifera Genoscope.12X	GSVIVT01010086001	Vv2R-MYB027	KQKVKRGLWSPEEDEKLIRYITTNGHGCWSSVPKLAGLQRCGKSCRLRWINYLRPDLKRGSFSAQEERTIIDVHRIVGNKWAQIAKYLPGRTDNEVKNFWNSCIKKKL
Vitis vinifera Genoscope.12X	GSVIVT01011417001	Vv2R-MYB028	KIGVKKGPWTPEEDIILVSYIQEHGPGNWRAVPTNTGLLRCSKSCRLRWTNYLRPGIKRGNFTDHEERMIIHLQALLGNRWAAIASYLPQRTDNDIKNYWNTHLKKKL
Vitis vinifera Genoscope.12X	GSVIVT01011436001	Vv2R-MYB029	SKLCARGHWRPAEDSKLRELVALYGPQNWNLIAEKLEGRSGKSCRLRWFNQLDPRINRRAFTEEEEERLMAAHRWYGNKWAMIARLFPGRTDNAVKNHWHVIMARKY
Vitis vinifera Genoscope.12X	GSVIVT01011447001	Vv2R-MYB030	KVGLKKGPWTPEEDQKLLAYIEEHGHGSWRALPSKAGLQRCGKSCRLRWTNYLRPDIKRGKFSLQEEQTIIQLHALLGNRWSAIATHLPKRTDNEIKNYWNTHLKKRL
Vitis vinifera Genoscope.12X	GSVIVT01011768001	Vv2R-MYB031	KQDTNKGAWSKQEDQKLIDYIRKNGEGCWRTLPQAAGLLRCGKSCRLRWINYLRPDLKRGNFAEDEEDLIIKLHALLGNRWSLIAGRLPGRTDNEVKNYWNSHLRRKL
Vitis vinifera Genoscope.12X	GSVIVT01011872001	Vv2R-MYB032	EIELRRGPWTLEEDTLLIHYIACHGEGRWNLLAKCAGLKRTGKSCRLRWLNYLKPDIKRGNLTPQEQLLILELHSKWGNRWSRIAQHLPGRTDNEIKNYWRTRVQKQA
Vitis vinifera Genoscope.12X	GSVIVT01012149001	Vv2R-MYB033	MGCPTALRKGCWTQEEDSLLRKYVKEFGEENWKHIAAKSGLRRCRKSCRLRWLNYLRPNIKRGNFGADEDDLIMRLHRLLGNRWSMIAGRIPGRTPNEIKNYWKSYLSKKI
Vitis vinifera Genoscope.12X	GSVIVT01012150001	Vv2R-MYB034	NTALRKGTWSEEEDRLLREYVDKYGQGNWKHITSKTGLRRCRKSCRLRWLNYLRPDIKRGSFGVDEDDLIIRLHRLLGNRWTLIAGRIPGRTSNDIKNYWNTYLSKKI
Vitis vinifera Genoscope.12X	GSVIVT01012447001	Vv2R-MYB035	GIVLKKGPWTSSEDAILVEYVKKHGEGNWNAVQKHSGLFRCGKSCRLRWANHLRPNLKKGAFTAEEERLIIELHAKMGNKWARMAAHLPGRTDNEIKNYWNTRIKRRQ
Vitis vinifera Genoscope.12X	GSVIVT01012777001	Vv2R-MYB036	KAHTNRGAWTKEEDDRLIEYIRMNGHGSWRSLPKAAGLLRCGKSCRLRWINYLRPDVKRGNFTIDEDELIIKLHKLLGNKWSLIAGRLPGRTDNEIKNHWNTHIRRRL

Vitis vinifera Genoscope.12X	GSVIVT01012778001	Vv2R-MYB037	KAHTNRGSWSKEEDDRLIHYIRLNGHGFWRSLPKAAGLLRCGKSCRLRWINYLRPDVKRGNFAIDEDELIIKLHKLLGNKWSLIAGRLPGRTDNEIKNHWNTHIRRKL
Vitis vinifera Genoscope.12X	GSVIVT01013126001	Vv2R-MYB038	DGSLKKGPWTPDEDQKLVDYIKRHGHGSWRALPKLAGLNRCGKSCRLRWTNYLRPDIKRGKFSDEEERIIIKLHSVLGNKWSRIAVHLPGRTDNEIKNFWNTHVKKKF
Vitis vinifera Genoscope.12X	GSVIVT01013735001	Vv2R-MYB039	EDNYKKGLWTKEEDRILLDYIQVHGRGRWNRISKITGLKRYGKSCRLRWMNYLSPSVKHGEFTEQEEDLIIRLHNLLGNRWSLIAGRVPGRTDNQVKNHWNSHLCKRL
Vitis vinifera Genoscope.12X	GSVIVT01014454001	Vv2R-MYB040	KNGMKKGPWTVEEDQKLLDYIQKHGHGRWRTLPKNAGLKRCGKSCRLRWTNYLRPDIKRGRFSFEEEEAIIQLHGILGNKWSAIAARLPGRTDNEIKNYWNTRIRKKL
Vitis vinifera Genoscope.12X	GSVIVT01014770001	Vv2R-MYB041	KDNVKRGQWTPEEDNKLSSYIAQHGTRNWRLIPKNAGLQRCGKSCRLRWTNYLRPDLKHGQFSDAEEQTIVKLHSVVGNRWSLIAAQLPGRTDNDVKNHWNTKLKKKL
Vitis vinifera Genoscope.12X	GSVIVT01015100001	Vv2R-MYB042	KEGLNRGAWTVVEDKILTEYIKVHGEGRWRNLPKKAGLKRCGKSCRLRWLNYLRPDIKRGNISHDEEDLIVRLHKLLGNRWSLIAGRLPGRTDNEIKNYWNTNLVKKM
Vitis vinifera Genoscope.12X	GSVIVT01015101001	Vv2R-MYB043	KEGLNRGTWSALEDKILCNYIEIYGEGKWKEVPPRAGLKRCGKSCRLRWLNYLRPDIKRGNISEDEEELIIKLHKLLGNRWSLIAGRLPGRTDNEIKNYWNTNLKKKL
Vitis vinifera Genoscope.12X	GSVIVT01015102001	Vv2R-MYB044	KEGLNRGSWSAWEDKILCNYVEVHGEGKWRDLPQRAGLKRCGKSCRLRWLNYLRPDIKRGNISSEEEELIIRLHKLLGNRWSLIAGRLPGRTDNEIKNYWNTNLSKRL
Vitis vinifera Genoscope.12X	GSVIVT01015264001	Vv2R-MYB045	KANVKRGPWSPEEDNILRAFVEAHGTGGNWIALPRKAGLKRCGKSCRLRWLNYLRPDIKHGGFTEDEDNIIVTLYNNIGSRWSVIASHLPRRTDNDVKNYWNTKLKKKL
Vitis vinifera Genoscope.12X	GSVIVT01015575001	Vv2R-MYB046	KPSVKKGLWKPEEDLLLKKYVEAHGEGKWATVSERSGLKRGGKSCRLRWKNYLRPNIKRGEISKEEEDLIIRMHKLLGNRWSLIAGRLPGRTDNEVKNYWNTHLNKGR
Vitis vinifera Genoscope.12X	GSVIVT01015578001	Vv2R-MYB047	KPRVKKGLWKPEEDLLLKKYVEAHGEGKWDAVSERTGLMRSGKSCRLRWKNYLRPNKKRGGISKDEEDIIIRMHKLLGNRWSLIAGRLPGRTDNDVKNYWNTHLNKGR
Vitis vinifera Genoscope.12X	GSVIVT01016393001	Vv2R-MYB048	SSSRVRGPWSPEEDAILSQLVSKFGPRNWSLIARGISGRSGKSCRLRWCNQLDPCVKRKPFTEEEDRIIIAAHSHHGNKWASIARLLPGRTDNAIKNHWNSTLKRQC
Vitis vinifera Genoscope.12X	GSVIVT01016765001	Vv2R-MYB049	RKEMNRGAWTAEEDRKLAEVIEVHGAKRWKMIATTAGLNRCGKSCRLRWMNYLRPNIKRGNISDQEEDLILRLHKLLGNRWSLIAGRLPGRTDNEIKNYWNSHLSKKI
Vitis vinifera Genoscope.12X	GSVIVT01016767001	Vv2R-MYB050	KEGLNRGAWTATEDKILTEYIKVHGEGKWRNLPKKAGLKRCGKSCRLRWLNYLRPDIKRGNITHDEEELIIRLHKLLGNRWSLIAGRLPGRTDNEIKNYWNTNIGKKI
Vitis vinifera Genoscope.12X	GSVIVT01016768001	Vv2R-MYB051	DVGLIRGAWSMQEDEVLINYIRVHGEGNWKSLAQKSGLKRCGKSCRLRWLNYLRPGLKRGSFSPDEEELIIKLHRLLGNRWALIAGRLPGRTDNEIKNYWNTTLCKRL
Vitis vinifera Genoscope.12X	GSVIVT01017716001	Vv2R-MYB052	KQKLKRGLWSPEEDEKLFRYITEHGHGCWSSVPKQAGLQRCGKSCRLRWINYLRPDLKRGAFTGQEEKLIVELHEILGNRWSQIASHLPGRTDNEIKNQWNSSIKKKL
Vitis vinifera Genoscope.12X	GSVIVT01018234001	Vv2R-MYB053	REEIKKGPWKAEEDEVLINHVNKYGPRDWSSIRSKGLLQRTGKSCRLRWVNKLRPNLKNGCKFSSEEERLVIELQAQFGNKWARIATYLPGRTDNDVKNFWSSRQKRLA
Vitis vinifera Genoscope.12X	GSVIVT01018577001	Vv2R-MYB054	KLGVKKGPWTAEEDKKLVTFILTNGHCCWRALPKLAGLRRCGKSCRLRWTNYLRPDLKRGLLNEAEEQLVIDLHARLGNRWSKIAAKLPGRTDNEIKNHWNTHIKKKL
Vitis vinifera Genoscope.12X	GSVIVT01018745001	Vv2R-MYB055	KAHTNKGAWTKEEDDRLIAHIQAHGEGCWRSLPKAAGLLRCGKSCRLRWINYLRPDLKRGNFTDEEDELIIKLHSLLGNKWSLIAGRLPGRTDNEIKNYWNTHIRRKL
Vitis vinifera Genoscope.12X	GSVIVT01019410001	Vv2R-MYB056	QQKVKRGLWSPEEDEKLIRYITTYGYGCWSEVPEKAGLQRCGKSCRLRWINYLRPDIRRGRFTPEEEKLIINLHGVVGNRWAHIASHLPGRTDNEIKNYWNSWIKKKI
Vitis vinifera Genoscope.12X	GSVIVT01019945001	Vv2R-MYB057	KVGLKKGSWTPEEDQKLMSYIEEHGHGSWRALPAKAGLQRCGKSCRLRWTNYLRPDIKRGKFSLQEEQTIIQLHALLGNRWSAIATHLPKRTDNEIKNYWNTHLKKRL
Vitis vinifera Genoscope.12X	GSVIVT01019967001	Vv2R-MYB058	SKLCARGHWRPAEDTKLKELVALYGPQNWNLIAEKLEGRSGKSCRLRWFNQLDPRINRRAFSEEEEERLMDAHRLYGNKWAMIARLFPGRTDNAVKNHWHVIMARKY
Vitis vinifera Genoscope.12X	GSVIVT01020038001	Vv2R-MYB059	QKERHIVTWSQQEDDILREQISLHGTENWAIIASKFKDKTTRQCRRRWYTYLNSDFKKGGWSPEEDLILCEAQKIFGNRWTEIAKVVSGRTDNAVKNRFSTLCKKRA
Vitis vinifera Genoscope.12X	GSVIVT01022654001	Vv2R-MYB060	MESLGVIKGAWSQKEDAPLRKCVEKYGEGKRHLVPPIAGLNRCRKSCRLRWVNYLKPNIKRGEFALDEVDLVIRLHNLLGNRWSLIAGRLPGRTANDVKNYWHTHHFKKK
Vitis vinifera Genoscope.12X	GSVIVT01022656001	Vv2R-MYB061	MKSLGVRKGAWTQEEDVLLRKCIEKYGEGKWHLVPLRAGLNRCLKSCRLRWLNYLKPDIKRGEFALDEVDLMIRLHNLLGNRWSLIAGRLPGRTANDVKNYWHGHHLKKK
Vitis vinifera Genoscope.12X	GSVIVT01022657001	Vv2R-MYB062	MESLGVRKGKWTREEDFLLRKCVEKYGEEKWHLVPLRAGLSRCRKSCRLRWFNYLKPNIKRGKFASDEVDLMIRLHKLLGNRWSLIAGRLPGRTANDVKNYWHHHRFKKM
Vitis vinifera Genoscope.12X	GSVIVT01022659001	Vv2R-MYB063	MESLGVRKGAWIQEEDVLLRKCIEKYGEGKWHLVPLRAGLNRCRKSCRLRWLNYLKPDIKRGEFALDEVDLMIRLHNLLGNRWSLIAGRLPGRTANDVKNYWHSHHFKKE
Vitis vinifera Genoscope.12X	GSVIVT01022661001	Vv2R-MYB064	MESLGVTKGKWTREEDILLRKCVENYGEEKWHLVPLRAGLSRCRKSCRLRWFNYLKPNIKRGKFASDEVDLMIRLHKLLGNRWSLIAGRLPGRTANDVKNYWHHHRFKKM
Vitis vinifera Genoscope.12X	GSVIVT01022664001	Vv2R-MYB065	MESLGVRKGAWTQEEDVLLRKCIEKYGEGKWHLVPLRAGLNRCRKSCRLRWLNYLKPDIKRGEFALDEVDLMIRLHNLLGNRWSLIAGRLPGRTANDVKNYWHGHHLKKK
Vitis vinifera Genoscope.12X	GSVIVT01023527001	Vv2R-MYB066	RIMIKGGVWKNTEDEILKAAVMKYGKNQWARISSLLVRKSAKQCKARWYEWLDPSIKKTEWTREEDEKLLHLAKLMPTQWRTIAPIVGRTPSQCLERYEKLLDAAC
Vitis vinifera Genoscope.12X	GSVIVT01023531001	Vv2R-MYB067	SMGLKKGSWTPEEDQILVSHIQRHGHGNWRALPKEAGLMRCGKSCRLRWTNYLRPDIKRGNFSKEEEETIIKLHQLLGNRRVLFFQFHYMNTDIILINSVKKTKLLWSAIAARLPG RTDNEIKNFWHSHLKKRL
Vitis vinifera Genoscope.12X	GSVIVT01024036001	Vv2R-MYB068	KAHTNKGAWTKEEDDRLIAYIRAHGEGCWRSLPKAAGLLRCGKSCRLRWINYLRPDLKRGNFTEEEDELIIKLHSLLGNKWSLIAGRLPGRTDNEIKNYWNTHIRRKL
Vitis vinifera Genoscope.12X	GSVIVT01024353001	Vv2R-MYB069	ESGLKKGPWTPEEDQKLVEYIQRHGHGSWRALPKLAGLNRCGKSCRLRWTNYLRPDIKRGKFSDEEENTIINLHSVLGNKWSAIATHLPGRTDNEIKNFWNTHLKKKL
Vitis vinifera Genoscope.12X	GSVIVT01024357001	Vv2R-MYB070	RNGLKKSPWTPKEDQKLIRYMKRHGEPTRWTEVPKLARLNRCGKSCRLRWSQYLKPGIKRGNFSQDEEDTIIRLQSAHGNRWVFIATQLPGRTDSEIKNYWNTSLKKKL
Vitis vinifera Genoscope.12X	GSVIVT0102477400	Vv2R-MYB071	SATLIKGQWSAEEDRQLLRLVNEFGEKKWAQIAKKLGGRAGKQCRERWHNHLRPDIRKDEWDEEEERILIEWHKKVGNKWAEIAKRITGRTENTIKNHFNATKRRQD
Vitis vinifera Genoscope.12X	GSVIVT01025269001	Vv2R-MYB072	GSKLRKGLWSPEEDDKLMNYMLNNGQGCWSDVARNAGLQRCGKSCRLRWINYLRPDLKRGAFSPQEEELIIHLHSLLGNRWSQIAARLPGRTDNEIKNFWNSTIKKRL
Vitis vinifera Genoscope.12X	GSVIVT01025452001	Vv2R-MYB073	KVGLKRGPWTPEEDEVLANYIKKEGEGRWRTLPKRAGLLRCGKSCRLRWMNYLRRPSVKRGQIAPDEEDLILRLHRLLGNRWALIAGRIPGRTDNEIKNYWNTHLSKKL
Vitis vinifera Genoscope.12X	GSVIVT01026481001	Vv2R-MYB074	GRNCQRGHWRPAEDEKLRQLVDQYGPHNWNSIADKLEGRSGKSCRLRWFNQLDPRINRKPFTEEEEERLLAAHRIHGNKWSLIARLFPGRTDNAVKNHYHVIMARRH
Vitis vinifera Genoscope.12X	GSVIVT01026868001	Vv2R-MYB075	KRGLKKGPWTPEEDQILVDYINKNGHGSWRSLPKNAGLLRCGKSCRLRWTNYLRPDIKRGPFTTDEEKLVIQLHGILGNRWAAIASQLPGRTDNEIKNLWNTHLKKRL
Vitis vinifera Genoscope.12X	GSVIVT01027182001	Vv2R-MYB076	KVGLHRGSWTAREDTLLTKYIQAHGEGHWRSLPKKAGLLRCGKSCRLRWMNYLRPDIKRGNITPDEDDLIIRLHSLLGNRWSLIAGRLPGRTDNEIKNYWNTHLSKKL
Vitis vinifera Genoscope.12X	GSVIVT01027810001	Vv2R-MYB077	KMGLKKGPWTPEEDKILVAHVQKHGHGNWRALPKQAGLLRCGKSCRLRWVNYLRPDIKRGNFSREEEDAIIDLHEKLGNRWSAIAARLPGRTDNEIKNVWHTNLKKRL
Vitis vinifera Genoscope.12X	GSVIVT01027811001	Vv2R-MYB078	KVGLKKGPWTTQEDQILTAYVLQHGHGNWRALPKQAGLLRCGKSCRLRWINYLRPDIKRGNFSREEEDTIIELHEKLGNKWSAIAASLPGRTDNEIKNVWHTHLKKRL
Vitis vinifera Genoscope.12X	GSVIVT01027949001	Vv2R-MYB079	EEGWRKGPWTAEEDRLLTEYVKLHGEGRWNSVARLAGLRRNGKSCRLRWVNYLRPDLKRGQITPHEENIILELHARWGNRWSTIARSLPGRTDNEIKNYWRTHFKKKG
Vitis vinifera Genoscope.12X	GSVIVT01028091001	Vv2R-MYB080	KVGLKKGRWTAEEDEVLVKYIQANGEGSWRSLPKNAGLLRCGKSCRLRWINYLRADLKRGNFSEEEEEIIIKLHASLGNRWSMIAGQLPGRTDNEIKNYWNSHLSRKV

Vitis vinifera Genoscope.12X	GSVIVT01028171001	Vv2R-MYB081	NMDLRKGPWTAEEDTLLNNYITIHGEGRWNSLARCAGLKRTGKSCRLRWLNYLRPNVKRGNITLQEQLLILELHSRYGNRWSKIAQHLPGRTDNEIKNYWRTRVQKQA
Vitis vinifera Genoscope.12X	GSVIVT01028235001	Vv2R-MYB082	KQKLRKGLWSPEEDEKLLRHITKYGHGCWSSVPKQAGLQRCGKSCRLRWINYLRPDLKRGTFSLQEENLIIELHSVLGNRWSQIAAQLPGRTDNEIKNLWNSCLKKKL
Vitis vinifera Genoscope.12X	GSVIVT01028328001	Vv2R-MYB083	KMGLKKGPWTPEEDQILVNYIHLYGHGNWRALPKQAGLLRCGKSCRLRWTNYLRPDIKRGNFTSEEEETIIELHERLGNRWSAIAAKLPGRTDNEIKNVWHTHLKKRL
Vitis vinifera Genoscope.12X	GSVIVT01028981001	Vv2R-MYB084	KHGVKRGAWTPEEDEILIDYIKKNGHGRWRSLPKHAGLLRCGKSCRLRWTNYLRPDIKRGPFTPEEETAIIQLHSMLGNRWAAIASKIPGRTDNEIKNFWNTHLKKRL
Vitis vinifera Genoscope.12X	GSVIVT01028984001	Vv2R-MYB085	KPKHRKGLWSPEEDARLRNYVLKYGLGCWSSVPVNAGLQRNGKSCRLRWINYLRPGLKRGMFTIEEEETIMALHRLLGNKWSQIAQNFPGRTDNEIKNYWHSCLKKKV
Vitis vinifera Genoscope.12X	GSVIVT01029145001	Vv2R-MYB086	KANVKKGPWSPEEDAKLKTYIEQYGTGGNWIALPQKIGRLKRCGKSCRLRWLNYLRPNIKHGGFSEEEDSIICNLYISIGSRWSVIAAQLPGRTDNDIKNYWNTRLKKKL
Vitis vinifera Genoscope.12X	GSVIVT01029904001	Vv2R-MYB087	KVGIKKGPWTPEEDIILVSYIQEHGPGNWRSVPTNTGLLRCSKSCRLRWTNYLRPGIKRGNFTPHEEGMIIHLQALLGNKWAAIASYLPQRTDNDIKNYWNTHLKKKI
Vitis vinifera Genoscope.12X	GSVIVT01029941001	Vv2R-MYB088	NKVCVRGHWRPHEDAKLREAVAQHGPQNWNLIAEKLVGRSGKSCRLRWFNQLDPRINRRAFSGEEEDRLLAAHRLYGNKWAMIARAFPGRTDNAVKNHWHVIMARRH
Vitis vinifera Genoscope.12X	GSVIVT01030434001	Vv2R-MYB089	NQGLKKGPWTTAEDAVLVEYVKRHGEGNWNSVQKNSGLSRCGKSCRLRWANHLRPNLKKGAFSPEEERLIVELHAKLGNKWARMAAQLPGRTDNEIKNYWNTRVKRRQ
Vitis vinifera Genoscope.12X	GSVIVT01030708001	Vv2R-MYB090	KVGLKKGPWTAEEDKKLINFILTNGQCCWRAVPKLAGLLRCGKSCRLRWTNYLRPDLKRGLLSDFEEQMVIDLHAQLGNRWSKIASHLPGRTDNEIKNHWNTHIKKKL
Vitis vinifera Genoscope.12X	GSVIVT01030819001	Vv2R-MYB091	MEGSLGLRKGAWTSEEDHLLRKCVEKYGEGKWHQVPFRAGLNRCRKSCRLRWLNYLKPDIKRGKFTADEVDLMMRLHKLLGNRWALIAGRLPGRTSNDVKNYWNTHLRKKM
Vitis vinifera Genoscope.12X	GSVIVT01030822001	Vv2R-MYB092	MDGPSGVRKGAWTREEDVLLRTCIEKYGEGKWHQVPFRSGLNRCRKSCRLRWLNYLRPDIKRGKFKTDEVDLIIRLHKLLGNRWALIAGRLPGRTSNDVKNYWNTHLSKKF
Vitis vinifera Genoscope.12X	GSVIVT01030866001	Vv2R-MYB093	KKDTTKGAWSKQEDQRLIDYIKTHGEGCWRSLPKAAGLHRCGKSCRLRWINYLRPDLKRGNFGQDEEDLIIKLHALLGNRWSLIAGRLPGRTDNEVKNYWNSHIRKKL
Vitis vinifera Genoscope.12X	GSVIVT01031341001	Vv2R-MYB094	KQKLRKGLWSPEEDEKLLMHITKYGHGCWSSVPKLAGLQRCGKSCRLRWINYLRPDLKRGAFSQQEESLIIELHAVLGNRWSQIAAQLPGRTDNEIKNLWNSCIKKKL
Vitis vinifera Genoscope.12X	GSVIVT01031496001	Vv2R-MYB095	KANVKKGPWSPEEDSKLKAYIEKYGTGGNWIALPQKAGLKRCGKSCRLRWLNYLRPNIKHGEFSDDEDRIICTLFANMGSRWSVIATRLPGRTDNDIKNYWNTKLKKKV
Vitis vinifera Genoscope.12X	GSVIVT01032088001	Vv2R-MYB096	MSYMLRNGQGCWSDIARNAGLQRCGKSCRLRWINYLRPDLKRGAFSPQEEELIIHLHSILGNRWSQIAARLPGRTDNEIKNFWNSTIKKRL
Vitis vinifera Genoscope.12X	GSVIVT01032442001	Vv2R-MYB097	KLNVVKGQWTPQEDCLLIQMVLNYGEKKWSEIAKHLNGRVGKQCRERWHNHLRPNIKKDPWTEEEDRILIQAHIEIGNRWAEISKRLPGRTENTIKNHWNATKRRHL
Vitis vinifera Genoscope.12X	GSVIVT01032452001	Vv2R-MYB098	DAEVRKGPWTLEEDLILINYIANHGEGVWNSLAKSAGLKRTGKSCRLRWLNYLRPDVRRGNITDEEQQLIMELHAKWGNRWSKIAKHLPGRTDNEIKNFWRTRIQKHI
Vitis vinifera Genoscope.12X	GSVIVT01032467001	Vv2R-MYB099	MEEVNQYKKGLWTLEEDKILMDYVKEHGKGQWNRIAKKTGLRRCGKSCRLRWINYLSPNVKRGDFTAEEEDLIIRLHKLLGNRWSLIAGRVPGRTDNQVKNYWNTHLSKKL
Vitis vinifera Genoscope.12X	GSVIVT01032564001	Vv2R-MYB100	KLNVKRGLWTAEEDAKILAYVSKHGIGNWTLVPKKAGLNRCGKSCRLRWTNYLRPDLKHDSFTPQEEDLIVNLHKAIGSRWSLIAKELPGRTDNDVKNYWNTKLRKKL
Vitis vinifera Genoscope.12X	GSVIVT01033418001	Vv2R-MYB101	KVGLKRGPWTPEEDELLANYVKREGEGRWRTLPKRAGLLRCGKSCRLRWMNYLRPSVKRGQIAPDEEDLILRLHRLLGNRWSLIAGRIPGRTDNEIKNYWNTHLSKKL
Vitis vinifera Genoscope.12X	GSVIVT01033648001	Vv2R-MYB102	KANVKKGPWSPEEDSKLKEYIEKYGTGGNWIALPQKAGLKRCGKSCRLRWLNYLRPNIKHGEFSDDEDRIICSVFASIGSKWSVIANYLPGRTDNDIKNYWNTKLKKKL
Vitis vinifera Genoscope.12X	GSVIVT01033670001	Vv2R-MYB103	GENLRKGSWLEEEDERLTAFVGLLGERRWDSIARASGLKRSGKSCRLRWLNYLRPDLKRCQISAEEEQIILQLHKRWGNKWSWIARSLPGRTDNEIKNYWRTHLRKRT
Vitis vinifera Genoscope.12X	GSVIVT01034041001	Vv2R-MYB104	RGDRVKGPWSPEEDAILSRLVSKFGARNWSLIARGIAGRSGKSCRLRWCNQLDPSVKRKPFTDEEDRIIVAAHAIHGNKWASIARLLQGRTDNAIKNHWNSTLRRRC
Vitis vinifera Genoscope.12X	GSVIVT01034166001	Vv2R-MYB105	MKERQRWRAEEDALLRAYVRQYGPREWNLVSQRMNTPLGRDAKSCLERWKNYLRPGIKKGSLTEEEQRLVIRLQAKHGNKWKKIAAEVPGRTAKRLGKWWEVFKEKQQ
Vitis vinifera Genoscope.12X	GSVIVT01034943001	Vv2R-MYB106	EMDLRRGPWTVEEDLTLVNYIATQGEGRWNSLARCAGLKRTGKSCRLRWLNYLRPDVRRGNITLEEQLLILELHSRWGNRWSKIAQHLPGRTDNEIKNYWRTRVQKHA
Vitis vinifera Genoscope.12X	GSVIVT01035041001	Vv2R-MYB107	KMGLKKGRWTEQEDEILRKYIQANGEGCWRSLPKNAGLLRCGKSCRLRWVNYLRDDLKRGNITAEEEDTIVKLHSSWGNRWSLIAGHLPGRTDNEIKNYWNSHLSRRI
Vitis vinifera Genoscope.12X	GSVIVT01035177001	Vv2R-MYB108	ESGLKKGPWTPEEDQKLVKYIQKQGHGSWRALPKLAGLNRCGKSCRLRWTNYLRPDIKRGKFSREEEETILNLHSILGNKWSAIASHLPGRTDNEIKNFRNTHLKKKL
Vitis vinifera Genoscope.12X	GSVIVT01035332001	Vv2R-MYB109	KANVKRGPWSPEEDATLRNYVQTHGIGGNWIALPRKAGLRRCGKSCRLRWLNYLRPDIKHGGFTEEEDKVICAIYNQMGSRWSVIASQLPGRTDNDVKNYWNTKLKKKL
Vitis vinifera Genoscope.12X	GSVIVT01035459001	Vv2R-MYB110	KEGLNRGAWTALEDKILTAYIKAHGEGKWRNLPKRAGLKRCGKSCRLRWLNYLRPDIKRGNISHDEEELIIRLHKLLGNRWSLIAGRLPGRTDNEIKNYWNTTLGKKI
Vitis vinifera Genoscope.12X	GSVIVT01035461001	Vv2R-MYB111	KEGMNKGAWTAIEDELLVEYIKVHGEGKWSGIPKKSGLKRCGKSCRLRWLNYLRPDIKRGNMSPEEEDLIIRLHKLLGNRWSLIAGRIPGRTDNEIKNYWNTNFAKKA
Vitis vinifera Genoscope.12X	GSVIVT01035462001	Vv2R-MYB112	KEGLNRGAWTALEDKILSDYIKVHGEGKWRNIPKQAGLKRCGKSCRLRWLNYLRPDIKRGNISLEEEDLIIRLHKLLGNRWALIAGRLPGRTDNEIKNYWNTTLRKKV
Vitis vinifera Genoscope.12X	GSVIVT01035463001	Vv2R-MYB113	EDGLNKGVWTAHEDEVLGNYVKLHGEGKWVNVAKATGLKRSAKSCRLRWLNYLKPDIKRGNIALDEEDLIIRLHRLLGNRWSLIAKRLPGRTDNEIKNYWHSTLKKKV
Vitis vinifera Genoscope.12X	GSVIVT01035464001	Vv2R-MYB114	TRRSNRGAWTALEDKLLRDYVKTHGPGKWRNVSQETGLERSGKSCRLRWLNYLRPDIKRGNISPEEEDLIIRLHKLLGNRWSLIAGRLPGRSDNEIKNYWNTTLAKRV
Vitis vinifera Genoscope.12X	GSVIVT01035467001	Vv2R-MYB115	KKEANRGAWTAEEDHKLAQVIAVHGAKRWKCVAMKAGLKRCGKSCRLRWMNYLRPNIKRGNISDQEQDLILRLHKLLGNRWSLIAGRLPGRTDNEIKNYWNSHLSKKT
Vitis vinifera Genoscope.12X	GSVIVT01036446001	Vv2R-MYB116	MKERQRWQPEEDALLRAYVKQYGAKEWNLISHRMGKSLDRDPKSCLERWKNYLKPGIKKGSLTLEEQNLVISLQAKYGNKWKKIASEVPGRTAKRLGKWWEVFKEKQL
Vitis vinifera Genoscope.12X	GSVIVT01036552001	Vv2R-MYB117	VQGWRKGPWTPEEDKLLSEYVSSHGEGRWSSVARCSGLNRSGKSCRLRWVNYLRPGLKRGHITTMEEGIIIELHALWGNKWSTIARYLPGRTDNEIKNYWRTHFKKNQ
Vitis vinifera Genoscope.12X	GSVIVT01036633001	Vv2R-MYB118	AKEGLNRGAWTVVEDNILTDYIQTHGEGGWRSIPKNAGLKRCGKSCRLRWLNYLKPDIKRGNISHAEEDLIIRLHRLLGNRWSMIAKRLPGRTDNEIKNYWNTNLRKKV
Vitis vinifera Genoscope.12X	GSVIVT01036712001	Vv2R-MYB119	KQKVKRGLWSPEEDEKLIRYITAHGHGSWSSVPKLAGLQRCGKSCRLRWINYLRPELKRGSFSAQEEQSIIDVHRILGNRWAQIAKHLPGRTDNEVKNFWNSCIKKKL
Vitis vinifera Genoscope.12X	GSVIVT01036802001	Vv2R-MYB120	KSKVKRGPWSPAEDMRLVSFIQKNGHENWRSLPKQAGLLRCGKSCRLRWINYLRPDVKRGNFSKEEEDAIIKLHQTLGNKWSKIASHFPGRTDNEIKNVWNTHLKKRL
Vitis vinifera Genoscope.12X	GSVIVT01037362001	Vv2R-MYB121	GGPLKKGPWTSAEDAVLIDYVTKHGEGNWNAVMKHSGLSRCGKSCRLRWANHLRPDLKKGAFTPEEEHRIIELHARMGNKWARMAAELPGRTDNEIKNYWNTRIKRLQ
Vitis vinifera Genoscope.12X	GSVIVT01037667001	Vv2R-MYB122	GVALKKGPWTAAEDAILVEYVTKHGEGNWNAVQKNSGLARCGKSCRLRWANHLRPNLKKGSFSAEEERVILELHAKLGNKWARMAAQLPGRTDNEIKNYWNTRIKRRL
Vitis vinifera Genoscope.12X	GSVIVT01025034001	Vv2R-MYB123	QEEIRKGPWTEKEDFQLVCFVGLFGDRRWDFIAKVSGLNRTGKSCRLRWVNYLHPGLKRGKMTPHEERLVLELHSQWGNRWSRIARKLPGRTDNEIKNYWRTHMRKKA
Vitis vinifera Genoscope.12X	GSVIVT01009566001	Vv2R-MYB124	KSERIKGSWSAEEDRVLTRLVERYGARNWSLISRYIKGRSGKSCRLRWCNQLSPSVEHRPFSAAEDDTILAAHARYGNRWATIARLLPGRTDNAVKNHWNSTLEAAF
Vitis vinifera Genoscope.12X	GSVIVT01013979001	Vv2R-MYB125	KNGLKKGPWTVEEDQKSPHYKEQRHGETSSWKNVPTRAGLSRSPKSCRLRWNSKLKPGINEKPFSEEEEATIIELQKQHGNKWATIAKCLPGRTDTGIKNFWNNHLKKKL

		Vitis vinifera Genoscope.12X	GSVIVT01035664001	Vv3R-MYB1	TRRSTTGKWTVEEDDMLREAVQCYKGKNWKKIVECLKDRTVIQCQHRWQKVLNPEIVKGSWTKEEDEKMMKLVKIYGPKKWSNIAKHLPGRIGKQCRERWHNHLNPAINKEAWTEEEDLALMHAHQIHGNKWAELTKFLPGRTDNAIKNHWNCSVKKKM
		Vitis vinifera Genoscope.12X	GSVIVT01015370001	Vv3R-MYB2	TRRSTKGQWTAEEDELLCKAVQRFKGKNWKKIAECFKDRTDVQCLHRWQKVLNPELIKGPWSKEEDEIIIELVNKYGPKKWSTIAQALPGRIGKQCRERWHNHLNPAINKEAWTQEEELALIHAHQIYGNKWAELTKFLPGRTDNAIKNHWNSSVKKKL
		Vitis vinifera Genoscope.12X	GSVIVT01035663001	Vv3R-MYB3	TRRSTKGQWTAEEDEILCKAVQRYKGKNWKKIAECFKDRTDVQCLHRWQKVLNPELVKGPWSKEEDEVIIELVNKYGAKKWSTIAQHLPGRIGKQCRERWHNHLNPAINKEAWTQEEELALVRAHQIYGNKWAELTKFLPGRTDNAIKNHWNSSVKKKL
		Vitis vinifera Genoscope.12X	GSVIVT01019834001	Vv3R-MYB4	TRRSTKGGWTEEEDNLLTTVVKNFNGRNWKKIAEYLHGRTDIQCLHRWQKVLNPELVKGPWTKEEDDCIVESVKKYGCKRWSMIAKALPGRIGKQCRERWHNHLDPAIKKDAWTKEEEAILTYYHQLYGNKWAEIARFLPGRNDNAIKNHWNCSIKKKS
		Vitis vinifera Genoscope.12X	GSVIVT01034171001	Vv3R-MYB5	PIRRAKGGWTPQEDDTLRTAVAYFKGKSWKKIAEFFPDRSEVQCLHRWQKVLNPELVKGPWTQEEDDKITELVSKYGPTKWSVIAKSLPGRIGKQCRERWHNHLNPDIKKDAWTLDEELALMNAHSKHGNKWAEIAKVLPGRTDNSIKNHWNSSLKKKL
		Vitis vinifera Genoscope.12X	GSVIVT01027493001	Vv3R-MYB6	ARRSTKGFWTEQKDRVLAYAVNKFKGKNWKKIAECVTGTTDVQCLHRWQKVLDPNLVKGPWTKEEDDLIIELVGKQGNKKWAEVAKCLTGRIGKQCRERWHNHLNPAINKAPWTKEEELVLIQAHQKYGNKWAEIAKILPGRTENSIKNHWNCSLKKRL
		Citrus sinensis v1.1	orange1.1g016021m	Csi2R-MYB01	GVILKKGPWTSAEDAILIDYVKKHGEGNWNAVQKNSGLFRCGKSCRLRWANHLRPNLKKGAFTQEEEQMIVELHAKMGNKWARMAAHLPGRTDNEIKNYWNTRIKRRQ
		Citrus sinensis v1.1	orange1.1g012494m	Csi2R-MYB02	SSSRVKGPWSPEEDAVLSRLVSQFGARNWGMIARGIPGRSGKSCRLRWCNQLDPCLKRKPFTDEEDQIIISAHAVHGNKWAVIARLLPGRTDNAIKNHWNSTLRRRY
		Citrus sinensis v1.1	orange1.1g013300m	Csi2R-MYB03	KQKLRKGLWSPEEDEKLLRHITKYGHGCWSSVPKQAGLQRCGKSCRLRWINYLRPDLKRGTFSQQEENLIIELHAVLGNRWSQIAAQLPGRTDNEIKNLWNSCLKKKL
		Citrus sinensis v1.1	orange1.1g014135m	Csi2R-MYB04	KQKLRKGLWSPEEDEKLLNYITKHGHGCWSSVPKLAGLQRCGKSCRLRWINYLRPDLKRGAFSVQEESLIVELHAVLGNRWSQIAAQLPGRTDDEIKNLWNSSIKKKL
		Citrus sinensis v1.1	orange1.1g014829m	Csi2R-MYB05	KVGLKKGRWTAEEDDILTKYIQANGEGSWRSLPKNAGLLRCGKSCRLRWINYLRADLKRGNITAEEEEIIIKLHASLGNRWSLIASNMPGRTDNEIKNYWNSHLSRKI
		Citrus sinensis v1.1	orange1.1g016021m	Csi2R-MYB06	KQKLRKGLWSPEEDEKLYNYITRFGVGCWSSVPKLAGLQRCGKSCRLRWINYLRPDLKRGMFSQQEEDLIISLHEVLGNRWAQIASQLPGRTDNEIKNFWNSCLKKKL
		Citrus sinensis v1.1	orange1.1g016708m	Csi2R-MYB07	KVGLKKGPWTPEEDQKLLAYIEEHGHGSWRALPAKAGLQRCGKSCRLRWTNYLRPDIKRGKFSLQEEQTIIQLHALLGNRWSAIATHLPKRTDNEIKNYWNTHLKKRL
		Citrus sinensis v1.1	orange1.1g017566m	Csi2R-MYB08	GRSRVKGPWSPEQDAVLSELVSKFGARNWSLIARGIAGRSGKSCRLRWCNQLDPAVKRKPFTDEEDQIIVAAHAVHGNKWAVISRLLPGRTDNAIKNHWNSTLRRRG
		Citrus sinensis v1.1	orange1.1g017727m	Csi2R-MYB09	KANVKKGPWSPEEDAKLKDYIEKYGTGGNWIALPQKIGLKRCGKSCRLRWLNYLRPNIKHGGFSEEEDNIICSLYISIGSRWSIIAAQLPGRTDNDIKNYWNTRLKKKL
		Citrus sinensis v1.1	orange1.1g018154m	Csi2R-MYB10	KSGLKKGPWTPEEDQKLIDYIQKHGYGNWRTLPKNAGLQRCGKSCRLRWTNYLRPDIKRGRFAFEEEETIIQLHSILGNKWSAIAARLPGRTDNEIKNYWNTHIRKRL
		Citrus sinensis v1.1	orange1.1g018559m	Csi2R-MYB11	KVGIKKGPWTPEEDIILVSYIQEHGPGNWRSVPTNTGLLRCSKSCRLRWTNYLRPGIKRGNFTPHEEGMIIHLQALLGNKWAAIASYLPQRTDNDIKNYWNTHLKKKL
		Citrus sinensis v1.1	orange1.1g018746m	Csi2R-MYB12	KIGIKKGPWTPEEDIILVSYIQEHGPGNWRAVPTNTGLLRCSKSCRLRWTNYLRPGIKRGNFTDQEEKMIIHLQALLGNRWAAIASYLRQRTDNDIKNYWNTHLKKKV
		Citrus sinensis v1.1	orange1.1g018768m	Csi2R-MYB13	MKERQRWRAEEDALLRAYVKQYGPKEWSFVSQRMNTPLNRDAKSCLERWKNYLKPGIKKGSLTDEEQHLVIHLQAKHGNKWKKIAAEVPGRTAKRLGKWWEVFKEKQQ
		Citrus sinensis v1.1	orange1.1g019307m	Csi2R-MYB14	KIGVKKGPWTPEEDIILVSYIQEHGPGNWRSVPTNTGLLRCSKSCRLRWTNYLRPGIKRGNFTDHEEKMIIHLQALLGNRWAAIASYLPQRTDNDIKNYWNTHLKKKL
		Citrus sinensis v1.1	orange1.1g019619m	Csi2R-MYB15	ANKLRKGLWSPEEDDKLIKYMLTNGQGCWSDIARNAGLQRCGKSCRLRWINYLRPDLKRGAFSPQEEELIIHLHSILGNRWSQIAARLPGRTDNEIKNFWNSTLKKIL
		Citrus sinensis v1.1	orange1.1g019787m	Csi2R-MYB16	KANVKKGPWSPEEDSKLKEYIEKYGTGGNWIALPQKAGLKRCGKSCRLRWLNYLRPNIKHGEFTDEEDRLICSLFASIGSRWSIIAAQLPGRTDNDIKNYWNTKLKKKL
		Citrus sinensis v1.1	orange1.1g019911m	Csi2R-MYB17	DLDLRRGPWTVEEDFKLINYISNHGEGRWNCLARCAGLKRTGKSCRLRWLNYLRPDVRRGNITLEEQLMILELHSRWGNRWSKIAQHLPGRTDNEIKNYWRTRVQKHA
		Citrus sinensis v1.1	orange1.1g020106m	Csi2R-MYB18	TSTCPRGHWRPAEDEKLRQLVEQYGAQNWNSIAEKLQGRSGKSCRLRWFNQLDPRINRRPFTEDEEERLLAAHRIHGNKWALIARLFPGRTDNAVKNHWHVIMARKQ
		Citrus sinensis v1.1	orange1.1g020197m	Csi2R-MYB19	DLDMRKGPWTVEEDFKLINYIVTHGEGRWNRLARCAGLKRTGKSCRLRWLNYLRPDVRLGKITLEEQLLILELHSRWGNRWSKLAQHLPGRTDNEIKNYWRTRVQKQA
		Citrus sinensis v1.1	orange1.1g020603m	Csi2R-MYB20	SDQLRRGPWTLEEDTLLIQYISRHSEGRWNLLAKRSGLKRTGKSCRLRWLNYLKPDVKRGNLTPQEQMSILELHSKWGNRWSKIAQHLPGRTDNEIKNYWRTRVQKQA
		Citrus sinensis v1.1	orange1.1g020613m	Csi2R-MYB21	QQKVKRGLWSPEEDEKLIRYITTHGYGCWSEVPEKAGLQRCGKSCRLRWINYLRPDIRRGRFTPEEEKLIISLHSVVGNRWAHIASHLPGRTDNEIKNYWNSWIKKKI
		Citrus sinensis v1.1	orange1.1g020997m	Csi2R-MYB22	KANVKKGPWSPEEDAKLKEYIEKNGTGGNWIALPHKAGLMRCGKSCRLRWLNYLRPNINHGEFSDDEDRIICSLFASIGSRWSTIAAQLPGRTDNDIKNYWNTKLKKKL
		Citrus sinensis v1.1	orange1.1g021071m	Csi2R-MYB23	EVDRIKGPWSPEEDEALQQLVQKHGPRNWSLISKSIPGRSGKSCRLRWCNQLSPQVEHRAFTPEEDEMIIRAHARFGNKWATIARLLNGRTDNAIKNHWNSTLKRKC
		Citrus sinensis v1.1	orange1.1g021164m	Csi2R-MYB24	GNKLRKGLWSPEEDDKLMNYMLKNGQGCWSDVARNAGLQRCGKSCRLRWINYLRPDLKRGAFSPQEEELIIHLHSLLGNRWSQIAARLPGRTDNEIKNFWNSTIKKRL
		Citrus sinensis v1.1	orange1.1g021188m	Csi2R-MYB25	QQELRRGPWTLEEDTLLTHYIHQHGEGRWNMVAKCAGLKRTGKSCRLRWLNYLKPDIKRGNLTPQEQFLILELHSKWGNRWSKIAQCLPGRTDNEIKNYWRTRVQKQA
		Citrus sinensis v1.1	orange1.1g021220m	Csi2R-MYB26	KEHTNKGAWTKEEDERLINYIKVHGEGCWRSLPKAAGLLRCGKSCRLRWINYLRPDLKRGNFTEEEDELIINFHSLLGNKWSLIAARLPGRTDNEIKNYWNTHIKRKL
		Citrus sinensis v1.1	orange1.1g021486m	Csi2R-MYB27	KKGLKKGPWAPEEDEILVEYIKRNGHGSWRSLPKLAGLLRCGKSCRLRWTNYLRPDIKRGPFTEEEEKLVIQLHGILGNRWAAIASQLPGRTDNEIKNLWNTHLKKRM
		Citrus sinensis v1.1	orange1.1g021498m	Csi2R-MYB28	KVGLKKGPWTAEEDKKLINFILTNGQCCWRAVPKLAGLLRCGKSCRLRWTNYLRPDLKRGLLSEYEEKMVIDLHAQLGNRWSKIASHLPGRTDNEIKNHWNTHIKKKL
		Citrus sinensis v1.1	orange1.1g021503m	Csi2R-MYB29	KAHTNKGAWTKEEDDRLIAYIRAHGEGCWRSLPKAAGLLRCGKSCRLRWINYLRPDLKRGNFTDEEDELIIKLHSLLGNKWSLIAGRLPGRTDNEIKNYWNTHIRRKL
		Citrus sinensis v1.1	orange1.1g022144m	Csi2R-MYB30	DVDRIKGPWSPEEDEALQRLVQNYGPRNWSLISKSIPGRSGKSCRLRWCNQLSPQVEHRPFTPEEDETILRAHARFGNKWATIARLLSGRTDNAIKNHWNSTLKRKC
		Citrus sinensis v1.1	orange1.1g023056m	Csi2R-MYB31	KAHTNKGAWTKEEDQRLIDYIRAHGEGCWRSLPKAAGLLRCGKSCRLRWINYLRPDLKRGNFTDEEDELIIKLHSLLGNKWSLIAGRLPGRTDNEIKNYWNTHIKRKL
		Citrus sinensis v1.1	orange1.1g023677m	Csi2R-MYB32	RLFRRYTNFRRSNSFNYMKYFVVGLNRTGKSCRLRWVNYLHPGLKRGKMTPQEERLVLELHAKWGNRWSRIARKLPGRTDNEIKNYWRTHMRKKA
		Citrus sinensis v1.1	orange1.1g024441m	Csi2R-MYB33	KLGVKKGPWTAEEDKKLINFILTNGQCCWRAVPKLAGLRRCGKSCRLRWTNYLRPDLKRGLLTEAEEQLVIDLHARLGNRWSKIAARLPGRTDNEIKNHWNTHIKKKL
		Citrus sinensis v1.1	orange1.1g024484m	Csi2R-MYB34	KMGLKKGPWTPEEDRILIVHIKKHGHPNWRALPKQAGLLRCGKSCRLRWINYLRPDIKRGNFSKEEEETIINLHDMLGNRWSAIAGRLPGRTDNEIKNVWHTHLKKKA
		Citrus sinensis v1.1	orange1.1g024492m	Csi2R-MYB35	KMGLKKGPWTPEEDQILINYVKLYGHGNWRALPKQAGLLRCGKSCRLRWINYLRPDIKRGNFTREEEDTIINLHEMLGNRWSAIAARLPGRTDNEIKNVWHTHLKKRL
		Citrus sinensis v1.1	orange1.1g024849m	Csi2R-MYB36	KLGVKKGPWTADEDKKLVTFILTHGHCCWRAVPKLAGLRRCGKSCRLRWTNYLRPDLKRGLLNEAEEQLVIDLHARLGNRWSKIAARLPGRTDNEIKNHWNTHIKKKL
		Citrus sinensis v1.1	orange1.1g024860m	Csi2R-MYB37	KTGLKKGTWTPEEDRKLIAYVTKYGYWNWRQLPKYAGLARCGKSCRLRWMNYLRPNIKRGNYTKEEEDTIIRLHESLGNRWSAIAAQLPGRTDNEIKNHWHTSLKKRL
		Citrus sinensis v1.1	orange1.1g025351m	Csi2R-MYB38	KPERIKGPWSAEEDRILTRLVERYGPRNWSLISRYIKGRSGKSCRLRWCNQLSPSVAHRPFSPAEDDTILAAHARFGNRWATIARLLPGRTDNAVKNHWNSTLKRRT

Citrus sinensis v1.1	orange1.1g025602m	Csi2R-MYB39	GEGLNRGAWKAVEDKILTDYIKAYGEGKWRHIPKAAGLKRCGKSCRLRWMNYLRPDIKRGNFSKDEEDLVIRLHKLLGNRWALIAGRLPGRTDNDIKNYWNTKLSKRV
Citrus sinensis v1.1	orange1.1g025697m	Csi2R-MYB40	MCTRGHWRPAEDEKLRELVERYGPHNWNAIAEKLQGRSGKSCRLRWFNQLDPRINRSPFTEEEEERLLASHRIHGNRWAVIARLFPGRTDNAVKNHWHVIMARRC
Citrus sinensis v1.1	orange1.1g028843m	Csi2R-MYB41	KNVVNRGAWTAEEDRKLTEYIEIHGAKRWKNVATESGLNRCGKSCRLRWLNYLRPNIKRGNISDDEEDLILRLHKLLGSRWSLIAGRLPGRTDNEIKNYWNSHLSKKI
Citrus sinensis v1.1	orange1.1g028922m	Csi2R-MYB42	KKEANRGAWTAEEDQKLAQAIEVHGPKKWKSVAAKAGLNRCGKSCRLRWMNYLRPHIKRGNISDQEEDLILRLHKLLGNRWSLIAGRLPGRTDNEIKNYWNSHLSKKI
Citrus sinensis v1.1	orange1.1g035629m	Csi2R-MYB43	KNGLKKGPWTPEEDHKLISYIQLHGPGNWRNLPKNAGLQRCGKSCRLRWTNYLRPDIKRGRFSFEEEETIIQLHSILGNKWSAIAARLPGRTDNEIKNYWNTHIRKRL
Citrus sinensis v1.1	orange1.1g036087m	Csi2R-MYB44	KQKVKKGLWSPEEDEKLLKHITAHGHGSWSSVPKLAGLQRCGKSCRLRWINYLRPDLRRGSFTEEEEQIIIDVHRILGNRWAQIAKHLPGRTDNEVKNYWNSCIKKKL
Citrus sinensis v1.1	orange1.1g036215m	Csi2R-MYB45	KVGIKKGPWTPEEDEILSNYIKREGEGQWRTLPKRAGLLRCGKSCRLRWMNYLRPSIKRGHIAPDEEDLILRLHRLLGNRWSLIAGRIPGRTDNEIKNYWNTRLRKKL
Citrus sinensis v1.1	orange1.1g036344m	Csi2R-MYB46	KANVKKGPWSPEEDAKLKAYIEQHGTGGNWIALPHKIGLKRCGKSCRLRWLNYLRPNIKHGGFSEEEDNIICSLYISIGSRWSIIAAQLPGRTDNDIKNYWNTRLKKKL
Citrus sinensis v1.1	orange1.1g036591m	Csi2R-MYB47	MDKSSPATFNKGAWTAEEDRKLAEVIATHGPTKWKTIAAKTGLNRCGKSCRLRWMNYLRPNIKRGSISDQEEDLILRLHKLLGNRWSLIAGRLPGRTDNEIKNYWNSHLSKKI
Citrus sinensis v1.1	orange1.1g037024m	Csi2R-MYB48	KANVKRGPWSAEEDSILKNYLEQFGNGGNWIALPKKAGLNRCGKSCRLRWLNYLRPDIKHGGFTKEEDTIICNLYCTMGSRWSVIASQLPGRTDNDVKNYWNTKLKKNV
Citrus sinensis v1.1	orange1.1g037956m	Csi2R-MYB49	KPSLRKGSWTPEEDRKLIAYIRRYGIWNWSEMPKYAGLLRCGKSCRLRWMNYLRPDIRRGNFTQEEDETIIKLHEQLGNRWSAIASRLPGRTDNEIKNHWHSRLSKKR
Citrus sinensis v1.1	orange1.1g037998m	Csi2R-MYB50	ENGLKKGPWTPEEDQKLVEFIQKHGHGSWRALPKLAGLNRCGKSCRLRWTNYLRPDIKRGKFSQDEEQTILHLHSILGNKWSAIATHLPGRTDNEIKNFWNTHLKKKL
Citrus sinensis v1.1	orange1.1g038795m	Csi2R-MYB51	REEIRKGPWKAEEDEVLINHVKKYGPRDWSSIRSKGLLQRTGKSCRLRWVNKLRPNLKNGCKFSLEEERIVIELQAQFGNKWARIATYLPGRTDNDVKNFWSSRQKRLA
Citrus sinensis v1.1	orange1.1g039016m	Csi2R-MYB52	KVGLKKGPWTPEEDQKLLAFIEQHGHGSWRALPAKAGLQRCGKSCRLRWINYLRPDIKRGKFSLQEEQTIIQLHALLGNRWSAIAAHLPKRTDNEIKNYWNTHLKKRL
Citrus sinensis v1.1	orange1.1g039070m	Csi2R-MYB53	KQKVKRGLWSPEEDEKLIKHVTTHGHGSWSSVPKLAGLQRCGKSCRLRWINYLRPDLKRGSFTEQEERIIIDIHRILGNRWAQIAKHLPGRTDNEVKNFWNSCIKKKL
Citrus sinensis v1.1	orange1.1g039198m	Csi2R-MYB54	INNIKKGPWTPEEDQKLIDYIRKHGHGSWRALPKLAGLNRCGKSCRLRWTNYLRPDIKRGKFSEEEEQLIVNLHAVLGNKWSAIATRLPGRTDNEIKNFWNTHLKKKL
Citrus sinensis v1.1	orange1.1g039708m	Csi2R-MYB55	HQSLKKGPWTAAEDAILMEYVKKHGEGNWNAVQKNSGLMRCGKSCRLRWANHLRPNLKKGSFSPEEEKIIIELHAKLGNKWARMAAQLPGRTDNEIKNYWNTRMKRRQ
Citrus sinensis v1.1	orange1.1g040502m	Csi2R-MYB56	VSERVKGPWSPEEDQLLLKLVQRYGARNWSVISKSIPGRSGKSCRLRWCNQLSPEVEHRTFTPDEDEIIIKAHARYGNKWATIARLLNGRTDNAIKNHWNSTLKRKY
Citrus sinensis v1.1	orange1.1g040726m	Csi2R-MYB57	KVGLKRGPWTPEEDELLANYINKEGEGRWRTLPKRAGLLRCGKSCRLRWMNYLRPSVKRGHIAPDEEDLILRLHRLLGNRWSLIAGRIPGRTDNEIKNYWNTHLSKKL
Citrus sinensis v1.1	orange1.1g040841m	Csi2R-MYB58	KVGLQRGPWTPREDTLLTEYIQVHGEGHWRSLPKKAGLLRCGKSCRLRWMNYLRPDIKRGNITPDEDDLIIRLHSLLGNRWSLIAGRLPGRTDNEIKNYWNTHLSKRL
Citrus sinensis v1.1	orange1.1g041081m	Csi2R-MYB59	KIGLKKGRWTAEEDEILTKYIRANGEGSWRSLPKNAGLLRCGKSCRLRWINYLRADLKRGNITAEEEETIVKLHSSLGNRWSLIAAQLPGRTDNEIKNYWNSHLSRKT
Citrus sinensis v1.1	orange1.1g041991m	Csi2R-MYB60	MEKMKRGVWSPEEDAKLIQYIKEHGHGTWRHLPKQAGILRSGKSCRLRWMNYLRPDIKRGPFAPEEVATIIQLHGLFGNKWAAIASHLPGRTDNDIKNLWNTRLKKHL
Citrus sinensis v1.1	orange1.1g042846m	Csi2R-MYB61	ENGMKKGAWSKEEDDKLRAYILKYGHWNWGELPKFAGLSRCGKSCRLRWMNYLRPDIKHGNYTKEEEDTIIRLRQQHGNKWSLIAAKLPGRTDNEIKNYWHTHLKKRK
Citrus sinensis v1.1	orange1.1g043269m	Csi2R-MYB62	ILGVRKGPWTVEEDSLLMNYVAIHGEGRWNAVASSAGLNRTGKSCRLRWLNYLRPDVRRGNITLQEQLMILELHSRWGNRWSKIAQYLPGRTDNEIKNYWRTRVQKHA
Citrus sinensis v1.1	orange1.1g043557m	Csi2R-MYB63	KSNVKRGLWTPEEDAKLLAHVANHGTGNWTLVPKKAGLNRCGKSCRLRWTNYLRPDLRHASFAPHEEEIIINLHKAIGSRWSLIAQQLPGRTDNDVKNFWNTKLKKKL
Citrus sinensis v1.1	orange1.1g043612m	Csi2R-MYB64	KQKVKRGLWSPEEDEKLINYVTTYGHGCWSSVPKHAGLQRCGKSCRLRWINYLRPDLKRGSFSPQEAALIIELHSILGNRWAQIARHLPGRTDNEKKLMSHEI
Citrus sinensis v1.1	orange1.1g044021m	Csi2R-MYB65	KNNIIKGQWTPQEDRMLIRLVAQHGTKKWSVIAKAMTGRVGKQCRERWYNHLKPDIKKEAWSEEEDMILIQAHKEVGNRWAEIARRLIGRTENTIKNHWNATKRRQQ
Citrus sinensis v1.1	orange1.1g044161m	Csi2R-MYB66	EEKLSKGPWHEEEDELLVTFVTLFGERRWDYIAKASGLKRSGKSCGLRWLNYLCPNIKHGYISTEEEQIIIQLHKKWGNKWSRIARNLPGRTDNEIKNYWRTRIRKKI
Citrus sinensis v1.1	orange1.1g044864m	Csi2R-MYB67	KQDTNKGAWSKQEDQKLIDYIRKHGEGCWRTIPQAAGLARCGKSCRLRWINYLRPDLKRGNFAEDEEDLIIKLHALLGNRWSLIAGRLPGRTANEVKNYWNSHLRRKL
Citrus sinensis v1.1	orange1.1g044977m	Csi2R-MYB68	KKERHIVTWTQQEDDILREQISIHGTENWSIIASKFKDKTTRQCRRRWYTYLNSDFKKGGWSPEEDMLLCEAQKIFGNRWTEIAKVVSGRTDNAVKNRFSTLCKKRA
Citrus sinensis v1.1	orange1.1g045384m	Csi2R-MYB69	EAEVRKGPWTMEEDLILINYIANHGEGVWNSLAKAAGLKRTGKSCRLRWLNYLRPDVKRGNITPEEQLLIMELHAKWGNRWSKIAKHLPGRTDNEIKNFWRTRIQKQI
Citrus sinensis v1.1	orange1.1g045387m	Csi2R-MYB70	KNGLKKGPWTAEEDQKLVDYIQKHGHGRWRTLPKNAGLKRCGKSCRLRWTNYLRPDIKRGKFSLEEEEAIIQLHKVLGNKWSAIASRLPGRTDNEIKNYWNTHIKKKL
Citrus sinensis v1.1	orange1.1g045400m	Csi2R-MYB71	KPKHRKGLWSPEEDQRLKNYVLQHGHPCWSSVPINAGLQRNGKSCRLRWINYLRPGLKRGVFNMQEEETILTLHRLLGNKWSQIAQHLPGRTDNEIKNYWHSHLKKKL
Citrus sinensis v1.1	orange1.1g045411m	Csi2R-MYB72	KSQVKRGPWSPGEDLRLITFIQKHGHENWRALPKQAGLLRCGKSCRLRWINYLRPDVKRGNFTQEEEDTIIRLHESLGNRWSKIASQLPGRTDNEIKNVWNTHLKKRL
Citrus sinensis v1.1	orange1.1g045434m	Csi2R-MYB73	ESGLKKGPWTSDEDQKLVKYIQKHGHGSWRALPKLAGLNRCGKSCRLRWTNYLRPDIKRGKFSQEEEQTILNLHSILGNKWSAIAGHLPGRTDNEIKNFWNTHLKKKL
Citrus sinensis v1.1	orange1.1g045583m	Csi2R-MYB74	KANVKRGPWSPEEDATLKRYVETHGTGGNWIALPQKAGLKRCGKSCRLRWLNYLRPDIKHGNFTEDEDHVICTLYSQIGSRWSIIASRLPGRTDNDVKNYWNTKLKKKL
Citrus sinensis v1.1	orange1.1g046075m	Csi2R-MYB75	SRSLKKGPWTPEEDQKLVDYIGRHGHGSWNALPKLAGLNRCGKSCRLRWTNYLRPDIRRGRFSEEEERIIINLHSVLGNKYFSFNMHIYSIEIIYHLPGRTDNEIKNFWNTHIRKKF
Citrus sinensis v1.1	orange1.1g046093m	Csi2R-MYB76	KVGLKKGKWTAKEDEILTKYIQANGIESWRSLPKKAGLLRCGKSCRLRWVNYLRTDLRRGNITTEEEETIIKLHASLGNRWSVIASHLPGRTDNELKNYWNTHLSKKI
Citrus sinensis v1.1	orange1.1g046309m	Csi2R-MYB77	TKTCARGHWRPAEDTKLKELVALYGPQNWNLIAEKLEGRSGKSCRLRWFNQLDPRINRRAFTEEEEERLMHAHRLYGNKWAMIARLFPGRTDNAVKNHWHVIMARKY
Citrus sinensis v1.1	orange1.1g046419m	Csi2R-MYB78	GVQLKKGPWTAAEDAILTEYVRKHGEGNWNAVQRNTGLARCGKSCRLRWANHLRPNLKKGTFSPEEERLIVELHAQLGNKWARMAAQLPGRTDNEIKNYWNTRVKRRH
Citrus sinensis v1.1	orange1.1g046570m	Csi2R-MYB79	NNDRIKGSWSPQEDATLIKLVEQHGPRNWSMISSGIPGRSGKSCRLRWCNQLSPTVQHRPFTPAEDSIIIQAHAVHGNKWATIARSLPGRTDNAIKNHWNSTLRRKR
Citrus sinensis v1.1	orange1.1g046581m	Csi2R-MYB80	SVALIKGQWTDEEDRKLIKLVKQYGVRKWAQISERMAGRAGKQCRERWHNHLRPDIKKDSWSEEEERILVEAHARIGNRWAEIAKHIPGRTENSIKNHWNATKRRQN
Citrus sinensis v1.1	orange1.1g046859m	Csi2R-MYB81	KSNVVKGQWTTEEDRLLIRLVDQFGIRKWSHIAQMLPGRIGKQCRERWHNHLRPDIKKDIWSEEEDKALIEAHAEIGNKWAEIAKRLPGRTENSIKNHWNATKRRQF
Citrus sinensis v1.1	orange1.1g047012m	Csi2R-MYB82	RIMIKGGVWKNTEDEILKAAVMKYGKNQWARISSLLVRKSAKQCKARWYEWLDPSIKKTEWTREEDEKLLHLAKLMPTQWRTIAPIVGRTPSQCLERYEKLLDAAC
Citrus sinensis v1.1	orange1.1g047101m	Csi2R-MYB83	TNGLKRGPWTPEEDQKLLDYIKKHGHGSWRSLPEKADLQRCGKSCRLRWTNYLRPDIKRGKFSLHEEQTIIQLHALLGNRWSAIAAHLPKRTDNEIKNYWNTHIKKRL

Citrus sinensis v1.1	orange1.1g047269m	Csi2R-MYB84	ENEYKKGLWTQEEDRILLEYIRVHGRGQWNRIHKVTGLKRCGKSCRLRWLNYLSPNVKHGEFTEEEENLIIRLHNLLGNRWSLIAGRVPGRTDNQVKNHWNTHLCKKL
Citrus sinensis v1.1	orange1.1g047550m	Csi2R-MYB85	SKVCSRGHWRPAEDSKLKELVALYGPQNWNLIAEKLEGRSGKSCRLRWFNQLDPRINKRAFSEDEEERLMAAHRVYGNKWAMIARLFPGRTDNAVKNHWHVIMARKY
Citrus sinensis v1.1	orange1.1g047839m	Csi2R-MYB86	DEGWRKGPWTAEEDRLLIEYVRLHGEGRWNSVARLAGLRRNGKSCRLRWVNYLRPDLKRGQITPHEESIILELHARWGNRWSTIARSLPGRTDNEIKNYWRTHFKKKA
Citrus sinensis v1.1	orange1.1g047854m	Csi2R-MYB87	EQGWRKGPWTPEEDKLLTEHVNLLGEGRWSSVARSAGLNRSGKSCRLRWVNYLRPGLKRGQLTPQEEGIIIELHALWGNKWSTIARYLPGRTDNEIKNYWRTHFKKEK
Citrus sinensis v1.1	orange1.1g009439m	Csi3R-MYB1	PIRRAKGGWTPEEDETLRNAVSTFKGKSWKKIAEFFPDRSEVQCLHRWQKVLNPDLVKGPWTQEEDDKITELVSKYGPTKWSVIAKSLPGRIGKQCRERWHNHLNPDIKKDAWT<br>LEEELALMNAHRIHGNKWAEIAKVLPGRTDNSIKNHWNSSLKKKL
Citrus sinensis v1.1	orange1.1g009430m	Csi3R-MYB2	PIRRAKGGWTPEEDETLRNAVSTFKGKSWKKIAEFFPDRSEVQCLHRWQKVLNPDLVKGPWTQEEDDKITELVSKYGPTKWSVIAKSLPGRIGKQCRERWHNHLNPDIKKDAWT<br>LEEELALMNAHRIHGNKWAEIAKVLPGRTDNSIKNHWNSSLKKKL
Citrus sinensis v1.1	orange1.1g009391m	Csi3R-MYB3	PIRRAKGGWTPEEDETLRNAVSTFKGKSWKKIAEFFPDRSEVQCLHRWQKVLNPDLVKGPWTQEEDDKITELVSKYGPTKWSVIAKSLPGRIGKQCRERWHNHLNPDIKKDAWT<br>LEEELALMNAHRIHGNKWAEIAKVLPGRTDNSIKNHWNSSLKKKL
Citrus sinensis v1.1	orange1.1g009430m	Csi3R-MYB4	PIRRAKGGWTPEEDETLRNAVSTFKGKSWKKIAEFFPDRSEVQCLHRWQKVLNPDLVKGPWTQEEDDKITELVSKYGPTKWSVIAKSLPGRIGKQCRERWHNHLNPDIKKDAWT<br>LEEELALMNAHRIHGNKWAEIAKVLPGRTDNSIKNHWNSSLKKKL
Arabidopsis thaliana	At3g27920	At2R-MYB000	NQEYKKGLWTVEEDNILMDYVLNHGTGQWNRIVRKTGLKRCGKSCRLRWMNYLSPNVNKGNFTEQEEDLIIRLHKLLGNRWSLIAKRVPGRTDNQVKNYWNTHLSKKL
Arabidopsis thaliana	At3g09230	At2R-MYB001	RRDRVKGPWSKEEDDVLSELVKRLGARNWSFIARSIPGRSGKSCRLRWCNQLNPNLIRNSFTEVEDQAIIAAHAIHGNKWAVIAKLLPGRTDNAIKNHWNSALRRRF
Arabidopsis thaliana	At2g47190	At2R-MYB002	DSDVRKGPWTEEEDAILVNFVSIHGDARWNHIARSSGLKRTGKSCRLRWLNYLRPDVRRGNITLEEQFMILKLHSLWGNRWSKIAQYLPGRTDNEIKNYWRTRVQKQA
Arabidopsis thaliana	At1g22640	At2R-MYB003	KAHMNKGAWTKEEDQLLVDYIRKHGEGCWRSLPRAAGLQRCGKSCRLRWMNYLRPDLKRGNFTEEEDELIIKLHSLLGNKWSLIAGRLPGRTDNEIKNYWNTHIKRKL
Arabidopsis thaliana	At4g38620	At2R-MYB004	KAHTNKGAWTKEEDERLVAYIKAHGEGCWRSLPKAAGLLRCGKSCRLRWINYLRPDLKRGNFTEEEDELIIKLHSLLGNKWSLIAGRLPGRTDNEIKNYWNTHIRRKL
Arabidopsis thaliana	At3g13540	At2R-MYB005	KMGMKRGPWTVEEDEILVSFIKKEGEGRWRSLPKRAGLLRCGKSCRLRWMNYLRPSVKRGGITSDEEDLILRLHRLLGNRWSLIAGRIPGRTDNEIKNYWNTHLRKKL
Arabidopsis thaliana	At4g09460	At2R-MYB006	KAHTNKGAWTKEEDQRLVDYIRNHGEGCWRSLPKSAGLLRCGKSCRLRWINYLRPDLKRGNFTDDEDQIIIKLHSLLGNKWSLIAGRLPGRTDNEIKNYWNTHIKRKL
Arabidopsis thaliana	At2g16720	At2R-MYB007	KEHMNKGAWTKEEDERLVSYIKSHGEGCWRSLPRAAGLLRCGKSCRLRWINYLRPDLKRGNFTHDEDELIIKLHSLLGNKWSLIAARLPGRTDNEIKNYWNTHIKRKL
Arabidopsis thaliana	At1g35515	At2R-MYB008	KAHMNKGAWTKEEDQRLIDYIRNHGEGSWRSLPKSVGLLRCGKSCRLRWINYLRPDLKRGNFTDGEEQIIVKLHSLFGNKWSLIAGKLPGRTDNEIKNYWNTHIKRKL
Arabidopsis thaliana	At5g16770	At2R-MYB009	ENGLKKGPWTQEEDDKLIDHIQKHGHGSWRALPKQAGLNRCGKSCRLRWTNYLRPDIKRGNFTEEEEQTIINLHSLLGNKWSSIAGNLPGRTDNEIKNYWNTHLRKKL
Arabidopsis thaliana	At3g12820	At2R-MYB010	KSQVKRGPWSDEESERLRSFILKNGHQNWRSLPKLAGLMRCGKSCRLRWINYLRPGLKRGNFTKEEEDTIIHLHQAYGNKWSKIASNFPGRTDNEIKNVWNTHLKKRL
Arabidopsis thaliana	At3g62610	At2R-MYB011	KVGIKKGRWTAEEDRTLSDYIQSNGEGSWRSLPKNAGLKRCGKSCRLRWINYLRSDIKRGNITPEEEDVIVKLHSTLGTRWSTIASNLPGRTDNEIKNYWNSHLSRKL
Arabidopsis thaliana	At2g47460	At2R-MYB012	KVGIKRGRWTAEEDQILSNYIQSNGEGSWRSLPKNAGLKRCGKSCRLRWINYLRSDLKRGNITPEEEELVVKLHSTLGNRWSLIAGHLPGRTDNEIKNYWNSHLSRK
Arabidopsis thaliana	At1g06180	At2R-MYB013	KIGLKKGPWSAEEDRILINYISLHGHPNWRALPKLAGLLRCGKSCRLRWINYLRPDIKRGNFTPHEEDTIISLHQLLGNRWSAIAAKLPGRTDNEIKNVWHTHLKKRL
Arabidopsis thaliana	At2g31180	At2R-MYB014	KMGVKRGPWTPEEDQILINYIHLYGHSNWRALPKHAGLLRCGKSCRLRWINYLRPDIKRGNFTPQEEQTIINLHESLGNRWSAIAAKLPGRTDNEIKNVWHTHLKKRL
Arabidopsis thaliana	At3g23250	At2R-MYB015	KMGLKRGPWTPEEDQILVSFILNHGHSNWRALPKQAGLLRCGKSCRLRWMNYLKPDIKRGNFTKEEEDAIISLHQILGNRWSAIAAKLPGRTDNEIKNVWHTHLKKRL
Arabidopsis thaliana	At5g15310	At2R-MYB016	KLGLKKGPWTPEEDQKLLAYIEEHGHGSWRSLPEKAGLHRCGKSCRLRWTNYLRPDIKRGKFNLQEEQTIIQLHALLGNRWSAIATHLPKRTDNEIKNYWNTHLKKRL
Arabidopsis thaliana	At3g61250	At2R-MYB017	KIGLKKGPWTPEEDEVLVAHIKKNGHGSWRTLPKLAGLLRCGKSCRLRWTNYLRPDIKRGPFTADEEKLVIQLHAILGNRWAAIAAQLPGRTDNEIKNLWNTHLKKRL
Arabidopsis thaliana	At4g25560	At2R-MYB018	GERHRKGLWSPEEDEKLRSFILSYGHSCWTTVPIKAGLQRNGKSCRLRWINYLRPGLKRDMISAEEEETILTFHSSLGNKWSQIAKFLPGRTDNEIKNYWHSHLKKKW
Arabidopsis thaliana	At5g52260	At2R-MYB019	KQRQRKGLWSPEEDQKLKSFILSRGHACWTTVPILAGLQRNGKSCRLRWINYLRPGLKRGSFSEEEEETILTLHSSLGNKWSRIAKYLPGRTDNEIKNYWHSYLKKRW
Arabidopsis thaliana	At1g66230	At2R-MYB020	KVGLKKGPWTAEEDRKLINFILTNGQCCWRAVPKLSGLLRCGKSCRLRWTNYLRPDLKRGLLSDYEEKMVIDLHSQLGNRWSKIASHLPGRTDNEIKNHWNTHIKTKL
Arabidopsis thaliana	At3g27810	At2R-MYB021	EAEVRKGPWTMEEDLILINYIANHGDGVWNSLAKSAGLKRTGKSCRLRWLNYLRPDVRRGNITPEEQLIIMELHAKWGNRWSKIAKHLPGRTDNEIKNFWRTRIQKYI
Arabidopsis thaliana	At5g40430	At2R-MYB022	KSDITKKRWTESEDIKLKEMVALEPKKWTKVAKHFEGRTPKQCRERWHNHARPNVKKTTWSEEEDQILIEVHKVIGAKWIQISEQLPGRSYNNVKNHWNTTKRRVQ
Arabidopsis thaliana	At5g40330	At2R-MYB023	EHEYKKGLWTVEEDKILMDYVRTHGQGHWNRIAKKTGLKRCGKSCRLRWMNYLSPNVNRGNFTDQEEDLIIRLHKLLGNRWSLIAKRVPGRTDNQVKNYWNTHLSKKL
Arabidopsis thaliana	At5g40350	At2R-MYB024	DAEVRKGPWTMEEDLILINYIANHGEGVWNSLAKSAGLKRTGKSCRLRWLNYLRPDVRRGNITPEEQLTIMELHAKWGNRWSKIAKHLPGRTDNEIKNFWRTKIQKYI
Arabidopsis thaliana	At2g39880	At2R-MYB025	GKSKVKGPWLPEQDEALTRLVKMCGPRNWNLISRGIPGRSGKSCRLRWCNQLDPILKRKPFSDEEEHMIMSAQAVLGNKWSVIAKLLPGRTDNAIKNHWNSNLRRKP
Arabidopsis thaliana	At3g13890	At2R-MYB026	KQKVKRGLWSPEEDEKLINYINSYGHGCWSSVPKHAGLQRCGKSCRLRWINYLRPDLKRGSFSPQEAALIIELHSILGNRWAQIAKHLPGRTDNEVKNFWNSSIKKKL
Arabidopsis thaliana	At3g53200	At2R-MYB027	EETLRRGPWLEEEDERLVKVISLLGERRWDSLAIVSGLKRSGKSCRLRWMNYLNPTLKRGPMSQEEERIIFQLHALWGNKWSKIARRLPGRTDNEIKNYWRTHYRKKQ
Arabidopsis thaliana	At5g61420	At2R-MYB028	GEGLKKGAWTTEEDKKLISYIHDHGEGGWRDIPQKAGLKRCGKSCRLRWTNYLKPEIKRGEFSSEEEQIIIMLHASRGNKWSVIARHLPRRTDNEIKNYWNTHLKKRL
Arabidopsis thaliana	At5g07690	At2R-MYB029	GEGLKKGAWTAEEDKKLISYIHEHGGGGWRDIPQKAGLKRCGKSCRLRWANYLKPDIKRGEFSYEEEQIIIMLHASRGNKWSVIARHLPKRTDNEIKNYWNTHLKKLL
Arabidopsis thaliana	At3g28910	At2R-MYB030	KGGVKKGPWTPEEDIILVTYIQEHGPGNWRAVPTNTGLLRCSKSCRLRWTNYLRPGIKRGNFTEHEEKMIVHLQALLGNRWAAIASYLPQRTDNDIKNYWNTHLKKKL
Arabidopsis thaliana	At1g74650	At2R-MYB031	KIEVKKGPWTPEEDIILVSYIQQHGPGNWRSVPANTGLLRCSKSCRLRWTNYLRPGIKRGNFTQPEEKMIIHLQALLGNRWAAIASYLPQRTDNDIKNYWNTHLKKKL
Arabidopsis thaliana	At4g34990	At2R-MYB032	KDHTNKGAWTKEEDDKLISYIKAHGEGCWRSLPRSAGLQRCGKSCRLRWINYLRPDLKRGNFTLEEDDLIIKLHSLLGNKWSLIATRLPGRTDNEIKNYWNTHVKRKL
Arabidopsis thaliana	At5g06100	At2R-MYB033	GHALKKGPWSSAEDDILIDYVNKHGEGNWNAVQKHTSLFRCGKSCRLRWANHLRPNLKKGAFSQEEEQLIVELHAKMGNRWARMAAHLPGRTDNEIKNYWNTRIKRRQ
Arabidopsis thaliana	At5g60890	At2R-MYB034	EEGIKKGAWTPEEDQKLIAYLHLHGEGGWRTLPEKAGLKRCGKSCRLRWANYLRPDIKRGEFSPEEDDTIIKLHALKGNKWAAIATSLAGRTDNEIKNYWNTNLKKRL
Arabidopsis thaliana	At3g28470	At2R-MYB035	KSNVKKGLWTEEEDAKILAYVAIHGVGNWSLIPKKAGLNRCGKSCRLRWTNYLRPDLKHDSFSTQEEELIIECHRAIGSRWSSIARKLPGRTDNDVKNHWNTKLKKKL

Arabidopsis thaliana	At5g57620	At2R-MYB036	KANVKKGPWSPEEDVKLKDYIDKYGTGGNWIALPQKIGLKRCGKSCRLRWLNYLRPNIKHGGFSEEEDRIILSLYISIGSRWSIIAAQLPGRTDNDIKNYWNTKLKKKL
Arabidopsis thaliana	At5g23000	At2R-MYB037	KTKVKRGPWSPEEDSKLRDYIEKYGNGGNWISFPLKAGLRRCGKSCRLRWLNYLRPNIKHGDFSEEEDRIIFSLFAAIGSRWSIIAAHLPGRTDNDIKNYWNTKLRKKL
Arabidopsis thaliana	At2g36890	At2R-MYB038	KANVKRGPWSPEEDAKLKDYIEKQGTGGNWIALPHKAGLRRCGKSCRLRWLNYLRPNIRHGDFTEEEDNIIYSLFASIGSRWSVIAAHLQGRTDNDIKNYWNTKLKKKL
Arabidopsis thaliana	At4g17780	At2R-MYB039	DKGVKKGPWLPEEDDKLTAYINENGYGNWRSLPKLAGLNRCGKSCRLRWMNYLRPDIRRGKFSDGEESTIVRLHALLGNKWSKIAGHLPGRTDNEIKNYWNTHMRKKL
Arabidopsis thaliana	At5g14340	At2R-MYB040	KIGLKRGPWTIEEDHRLMNFILNNGIHCWRIVPKLAGLLRCGKSCRLRWINYLRPDLKRGGFTDAEEDRIMELHSQLGNRWSKIASHFSGRTDNEIKNHWNTKIKKKM
Arabidopsis thaliana	At4g28110	At2R-MYB041	KNGVKKGPWTAEEDQKLIDYIRFHGPGNWRTLPKNAGLHRCGKSCRLRWTNYLRPDIKRGRFSFEEEETIIQLHSVMGNKWSAIAARLPGRTDNEIKNHWNTHIRKRL
Arabidopsis thaliana	At4g12350	At2R-MYB042	KLMVKKGPWTAEEDKKLINFILTNGHCCWRALPKLAGLRRCGKSCRLRWTNYLRPDLKRGLLSDAEEQLVIDLHALLGNRWSKIAARLPGRTDNEIKNHWNTHIKKKL
Arabidopsis thaliana	At5g16600	At2R-MYB043	KVGLKKGPWTIEEDKKLINFILTNGHCCWRALPKLSGLLRCGKSCRLRWINYLRPDLKRGLLSEYEEQKVINLHAQLGNRWSKIASHLPGRTDNEIKNHWNTHIKKKL
Arabidopsis thaliana	At5g67300	At2R-MYB044	MADRIKGPWSPEEDEQLRRLVVKYGPRNWTVISKSIPGRSGKSCRLRWCNQLSPQVEHRPFSAEEDETIARAHAQFGNKWATIARLLNGRTDNAVKNHWNSTLKRKC
Arabidopsis thaliana	At3g48920	At2R-MYB045	KEKQRKGLWSPEEDEKLRSHVLKYGHGCWSTIPLQAGLQRNGKSCRLRWVNYLRPGLKKSLFTKQEETILLSLHSMLGNKWSQISKFLPGRTDNEIKNYWHSNLKKGV
Arabidopsis thaliana	At5g12870	At2R-MYB046	VKKMKKGLWSPEEDSKLMQYMLSNGQGCWSDVAKNAGLQRCGKSCRLRWINYLRPDLKRGAFSPQEEDLIIRFHSILGNRWSQIAARLPGRTDNEIKNFWNSTIKKRL
Arabidopsis thaliana	At1g18710	At2R-MYB047	VDGMKKGEWTAEEDQKLGAYINEHGVCDWRSLPKRAGLQRCGKSCRLRWLNYLKPGIRRGKFTPQEEEEIIQLHAVLGNRWAAMAKKMQNRTDNDIKNHWNSCLKKRL
Arabidopsis thaliana	At3g46130	At2R-MYB048	EEGNRKGPWTEQEDILLVNFVHLFGDRRWDFIAKVSGLNRTGKSCRLRWVNYLHPGLKRGKMTPQEERLVLELHAKWGNRWSKIARKLPGRTDNEIKNYWRTHMRKKA
Arabidopsis thaliana	At5g54230	At2R-MYB049	ESEVKKGPWTPEEDEKLVGYIQTHGPGKWRTLPKNAGLKRCGKSCRLRWTNYLRPDIKRGEFSLQEEETIIQLHRLLGNKWSAIAIHLPGRTDNEIKNYWNTHIKKKL
Arabidopsis thaliana	At1g57560	At2R-MYB050	KQKLRKGLWSPEEDEKLLNYITKHGHGCWSSVPKLAGLERCGKSCRLRWINYLRPDLKRGAFSSEEQNLIVELHAVLGNRWSQIAARLPGRTDNEIKNLWNSCIKKKL
Arabidopsis thaliana	At1g18570	At2R-MYB051	ELGLKKGAWTPEEDQKLLSYLNRHGEGGWRTLPEKAGLKRCGKSCRLRWANYLRPDIKRGEFTEDEERSIISLHALHGNKWSAIARGLPGRTDNEIKNYWNTHIKKRL
Arabidopsis thaliana	At1g17950	At2R-MYB052	MMCSRGHWRPAEDEKLRELVEQFGPHNWNAIAQKLSGRSGKSCRLRWFNQLDPRINRNPFTEEEEERLLASHRIHGNRWSVIARFFPGRTDNAVKNHWHVIMARRG
Arabidopsis thaliana	At5g65230	At2R-MYB053	ETGLKKGPWLPEEDDKLINYIHKHGHSSWSALPKLAGLNRCGKSCRLRWTNYLRPDIKRGKFSAEEEETILNLHAVLGNKWSMIASHLPGRTDNEIKNFWNTHLKKKL
Arabidopsis thaliana	At1g73410	At2R-MYB054	MIMCSRGHWRPAEDEKLKDLVEQYGPHNWNAIALKLPGRSGKSCRLRWFNQLDPRINRNPFTEEEEERLLAAHRIHGNRWSIIARLFPGRTDNAVKNHWHVIMARRT
Arabidopsis thaliana	At4g01680	At2R-MYB055	KQKLRKGLWSPEEDEKLLRYITKYGHGCWSSVPKQAGLQRCGKSCRLRWINYLRPDLKRGAFSQDEENLIIELHAVLGNRWSQIAAQLPGRTDNEIKNLWNSCLKKKL
Arabidopsis thaliana	At5g17800	At2R-MYB056	TKVCSRGHWRPTEDAKLKELVAQFGPQNWNLISNHLLGRSGKSCRLRWFNQLDPRINKRAFTEEEEFRLLAAHRAYGNKWALISRLFPGRTDNAVKNHWHVIMARRT
Arabidopsis thaliana	At3g01530	At2R-MYB057	EGTVRKGPWTMEEDFILFNYILNHGEGLWNSVAKASGLKRTGKSCRLRWLNYLRPDVRRGNITEEEQLLIIQLHAKLGNRWSKIAKHLPGRTDNEIKNFWRTKIQRHM
Arabidopsis thaliana	At1g16490	At2R-MYB058	KTKVKRGPWSHDEDLKLISFIHKNGHENWRSLPKQAGLLRCGKSCRLRWINYLRPDVKRGNFSAEEEDTIIKLHQSFGNKWSKIASKLPGRTDNEIKNVWHTHLKKRL
Arabidopsis thaliana	At5g59780	At2R-MYB059	QEEYRKGPWTEQEDILLVNFVHLFGDRRWDFVAKVSGLNRTGKSCRLRWVNYLHPGLKRGKMTPQEERLVLELHAKWGNRWSKIARKLPGRTDNEIKNYWRTHMRKKA
Arabidopsis thaliana	At1g08810	At2R-MYB060	KIGIKKGPWTPEEDIILVSYIQEHGPGNWRSVPTNTGLLRCSKSCRLRWTNYLRPGIKRGNFTPHEEGMIIHLQALLGNKWASIASYLPQRTDNDIKNYWNTHLKKKL
Arabidopsis thaliana	At1g09540	At2R-MYB061	KQKLRKGLWSPEEDEKLLTHITNHGHGCWSSVPKLAGLQRCGKSCRLRWINYLRPDLKRGAFSPEEENLIVELHAVLGNRWSQIASRLPGRTDNEIKNLWNSSIKKKL
Arabidopsis thaliana	At1g68320	At2R-MYB062	DEELRRGPWTLEEDTLLTNYILHNGEGRWNHVAKCAGLKRTGKSCRLRWLNYLKPDIRRGNLTPQEQLLILELHSKWGNRWSKIAQYLPGRTDNEIKNYWRTRVQKQA
Arabidopsis thaliana	At1g79180	At2R-MYB063	KTKVKRGPWSPEEDIKLISFIQKFGHENWRSLPKQSGLLRCGKSCRLRWINYLRPDLKRGNFTSEEEETIIKLHHNYGNKWSKIASQLPGRTDNEIKNVWHTHLKKRL
Arabidopsis thaliana	At5g11050	At2R-MYB064	YKNIIKGQWTADEDRKLIKLVMQHGERKWAVISEKLEGRAGKQCRERWHNHLRPDIKKDSWSEEEERLLVEAHTRIGNKWAEIAKLIQGRTENSIKNHWNATKRRQN
Arabidopsis thaliana	At3g11440	At2R-MYB065	RSVLKKGPWTSTEDGILIDYVKKHGEGNWNAVQKHTSLARCGKSCRLRWANHLRPNLKKGAFSQEEEQLIVEMHAKMGNKWAQMAEHLPGRTDNEIKNYWNTRIKRRQ
Arabidopsis thaliana	At5g14750	At2R-MYB066	NNEYKKGLWTVEEDKILMDYVKAHGKGHWNRIAKKTGLKRCGKSCRLRWMNYLSPNVKRGNFTEQEEDLIIRLHKLLGNRWSLIAKRVPGRTDNQVKNYWNTHLSKKL
Arabidopsis thaliana	At3g12720	At2R-MYB067	KHKVKRGLWSPEEDEKLLRYITTHGHPSWSSVPKLAGLQRCGKSCRLRWINYLRPDLRRGSFNEEEEQIIIDVHRILGNKWAQIAKHLPGRTDNEVKNFWNSCIKKKL
Arabidopsis thaliana	At5g65790	At2R-MYB068	KANVKKGPWSPEEDAKLKDYIENSGTGGNWIALPQKIGLRRCGKSCRLRWLNYLRPNIKHGGFSEEEDNIICNLYVTIGSRWSIIAAQLPGRTDNDIKNYWNTRLKKKL
Arabidopsis thaliana	At4g33450	At2R-MYB069	KPSCQRGHWRPVEDDNLRQLVEQYGPKNWNFIAQHLYGRSGKSCRLRWYNQLDPNITKKPFTEEEEERLLKAHRIQGNRWASIARLFPGRTDNAVKNHFHVIMARRK
Arabidopsis thaliana	At2g23290	At2R-MYB070	EMDRIKGPWSPEEDDLLQSLVQKHGPRNWSLISKSIPGRSGKSCRLRWCNQLSPEVEHRGFTAEEDDTIILAHARFGNKWATIARLLNGRTDNAIKNHWNSTLKRKC
Arabidopsis thaliana	At3g24310	At2R-MYB071	EEGWRKGPWTAEEDRLLIDYVQLHGEGRWNSVARLAGLKRNGKSCRLRWVNYLRPDLKRGQITPHEETIILELHAKWGNRWSTIARSLPGRTDNEIKNYWRTHFKKKT
Arabidopsis thaliana	At1g56160	At2R-MYB072	KNKVKRGPWSPQEDLTLITFIQKHGHQNWRSLPKLAGLLRCGKSCRLRWINYLRPDVKRGNFSKKEEDAIIHYHQTLGNKWSKIASFLPGRTDNEIKNVWNTHLKKRL
Arabidopsis thaliana	At4g37260	At2R-MYB073	NMERIKGPWSPEEDDLLQRLVQKHGPRNWSLISKSIPGRSGKSCRLRWCNQLSPEVEHRAFSQEEDETIIRAHARFGNKWATISRLLNGRTDNAIKNHWNSTLKRKC
Arabidopsis thaliana	At4g05100	At2R-MYB074	KNGLKKGPWTPEEDQKLIDYINIHGYGNWRTLPKNAGLQRCGKSCRLRWTNYLRPDIKRGRFSFEEEETIIQLHSIMGNKWSAIAARLPGRTDNEIKNYWNTHIRKRL
Arabidopsis thaliana	At1g56650	At2R-MYB075	SKGLRKGAWTTEEDSLLRQCINKYGEGKWHQVPVRAGLNRCRKSCRLRWLNYLKPSIKRGKLSSDEVDLLLRLHRLLGNRWSLIAGRLPGRTANDVKNYWNTHLSKKH
Arabidopsis thaliana	At5g07700	At2R-MYB076	GEGLKKGAWTTEEDKKLISYIHDHGEGGWRDIPEKAGLKRCGKSCRLRWTNYLKPDIKRGEFSYEEEQIIIMLHASRGNKWSVIARHLPKRTDNEVKNYWNTHLKKRL
Arabidopsis thaliana	At3g50060	At2R-MYB077	MADRVKGPWSQEEDEQLRRMVEKYGPRNWSAISKSIPGRSGKSCRLRWCNQLSPEVEHRPFSPEEDETIVTARAQFGNKWATIARLLNGRTDNAVKNHWNSTLKRKC
Arabidopsis thaliana	At5g49620	At2R-MYB078	EMDVRRGPWTVEEDLELINYIASHGEGRWNSLARCAELKRTGKSCRLRWLNYLRPDVRRGNITLEEQLLILELHTRWGNRWSKIAQYLPGRTDNEIKNYWRTRVQKHA
Arabidopsis thaliana	At4g13480	At2R-MYB079	MVEEVWRKGPWTAEEDRLLIEYVRVHGEGRWNSVSKLAGLKRNGKSCRLRWVNYLRPDLKRGQITPHEESIILELHAKWGNRWSTIARSLPGRTDNEIKNYWRTHFKKKA
Arabidopsis thaliana	At5g56110	At2R-MYB080	KENVKRGQWTPEEDNKLASYIAQHGTRNWRLIPKNAGLQRCGKSCRLRWTNYLRPDLKHGQFSEAEEHIIVKFHSVLGNRWSLIAAQLPGRTDNDVKNYWNTKLKKKL

Arabidopsis thaliana	At2g26960	At2R-MYB081	KKSFTKGPWTQAEDNLLIAYVDKHGDGNWNAVQNNSGLSRCGKSCRLRWVNHLRPDLKKGAFTEKEEKRVIELHALLGNKWARMAEELPGRTDNEIKNFWNTRLKRLQ
Arabidopsis thaliana	At5g52600	At2R-MYB082	KSYVKRGLWKPEEDMILKSYVETHGEGNWADISRRSGLKRGGKSCRLRWKNYLRPNIKRGSMSPQEQDLIIRMHKLLGNRWSLIAGRLPGRTDNEVKNYWNTHLNKKP
Arabidopsis thaliana	At3g08500	At2R-MYB083	KPKLRKGLWSPDEDEKLIRYMLTNGQGCWSDIARNAGLLRCGKSCRLRWINYLRPDLKRGSFSPQEEDLIFHLHSILGNRWSQIATRLPGRTDNEIKNFWNSTLKKRL
Arabidopsis thaliana	At3g49690	At2R-MYB084	KANVKKGPWSPEEDAKLKSYIENSGTGGNWIALPQKIGLKRCGKSCRLRWLNYLRPNIKHGGFSEEEENIICSLYLTIGSRWSIIAAQLPGRTDNDIKNYWNTRLKKKL
Arabidopsis thaliana	At4g22680	At2R-MYB085	KLGVKKGPWTVEEDKKLINFILTNGHCCWRALPKLAGLRRCGKSCRLRWTNYLRPDLKRGLLSHDEEQLVIDLHANLGNKWSKIASRLPGRTDNEIKNHWNTHIKKKL
Arabidopsis thaliana	At5g26660	At2R-MYB086	KQKLRKGLWSPEEDEKLLNYITRHGHGCWSSVPKLAGLQRCGKSCRLRWINYLRPDLKRGAFSQDEESLIIELHAALGNRWSQIATRLPGRTDNEIKNFWNSCLKKKL
Arabidopsis thaliana	At4g37780	At2R-MYB087	KMAVKKGPWSTEEDAVLKSYIEKHGTGNNWISLPQRIGIKRCGKSCRLRWLNYLRPNLKHGGFTDEEDYIICSLYITIGSRWSIIASQLPGRTDNDIKNYWNTRLKKKL
Arabidopsis thaliana	At2g02820	At2R-MYB088	KKERHIVTWSPEEDDILRKQISLQGTENWAIIASKFNDKSTRQCRRRWYTYLNSDFKRGGWSPEEDTLLCEAQRLFGNRWTEIAKVVSGRTDNAVKNRFTTLCKKRA
Arabidopsis thaliana	At5g39700	At2R-MYB089	KKKHRGGHWTLSEDLKLKELVAVFGPQNWKFIGEKMEPRTSLSCRQRWFNQLDPKINKRNFTDEEEEKLLRAHILYGNKWSKIAKLFNRRTDHAVKNHWHSLMNRII
Arabidopsis thaliana	At1g66390	At2R-MYB090	SKGLRKGAWTAEEDSLLRLCIDKYGEGKWHQVPLRAGLNRCRKSCRLRWLNYLKPSIKRGRLSNDEVDLLLRLHKLLGNRWSLIAGRLPGRTANDVKNYWNTHLSKKH
Arabidopsis thaliana	At2g37630	At2R-MYB091	MKERQRWSGEEDALLRAYVRQFGPREWHLVSERMNKPLNRDAKSCLERWKNYLKPGIKKGSLTEEEQRLVIRLQEKHGNKWKKIAAEVPGRTAKRLGKWWEVFKEKQQ
Arabidopsis thaliana	At5g10280	At2R-MYB092	DSGLKKGPWTPDEDEKLVNYVQKHGHSSWRALPKLAGLNRCGKSCRLRWTNYLRPDIKRGRFSPDEEQTILNLHSVLGNKWSTIANQLPGRTDNEIKNFWNTHLKKKL
Arabidopsis thaliana	At1g34670	At2R-MYB093	ENGLKKGPWTPEEDQKLIDYIHKHGHGSWRALPKLADLNRCGKSCRLRWTNYLRPDIKRGKFSAEEEQTILHLHSILGNKWSAIATHLQGRTDNEIKNFWNTHLKKKL
Arabidopsis thaliana	At3g47600	At2R-MYB094	KIGVKKGPWTPEEDIILVSYIQEHGPGNWRSVPTHTGLRRCSKSCRLRWTNYLRPGIKRGNFTEHEEKMILHLQALLGNRWAAIASYLPERTDNDIKNYWNTHLKKKL
Arabidopsis thaliana	At1g74430	At2R-MYB095	VDGLRKGEWTAEEDRKLVVYINEHGLGEWGSLPKRAGLQRCGKSCRLRWLNYLRPGIKRGKFTPQEEEIIKYHALLGNRWAAIAKQMPNRTDNDIKNHWNSCLKKRL
Arabidopsis thaliana	At5g62470	At2R-MYB096	KIGVKKGPWTPEEDIILVSYIQEHGPGNWRSVPTHTGLRCSKSCRLRWTNYLRPGIKRGNFTEHEEKTIVHLQALLGNRWAAIASYLPERTDNDIKNYWNTHLKKKL
Arabidopsis thaliana	At4g26930	At2R-MYB097	GVVLKKGPWTVAEDETLAAYVREYGEGNWNSVQKKTWLARCGKSCRLRWANHLRPNLRKGSFTPEEERLIIQLHSQLGNKWARMAAQLPGRTDNEIKNYWNTRLKRFQ
Arabidopsis thaliana	At4g18770	At2R-MYB098	KSTLVKGQWTAEEDRVLIQLVEKYGLRKWSHIAQVLPGRIGKQCRERWHNHLRPDIKKETWSEEEDRVLIEFHKEIGNKWAEIAKRLPGRTENSIKNHWNATKRRQF
Arabidopsis thaliana	At5g62320	At2R-MYB099	EVGLRKGPWTVEEDGKLVDFLRARGNCGGGGGGWCWRDVPKLAGLRRCGKSCRLRWTNYLRPDLKRGLFTEEEIQLVIDLHARLGNRWSKIAVELPGRTDNDIKNYWNTHIKR KL
Arabidopsis thaliana	At2g25230	At2R-MYB100	KSDIDKVNWTESEDIKLKEIMALGPKNKWTKVAKKFEGRTGKQCRERWYNHARPNIKKTAWSEEEDQILIEAHKVLGTKWVEIAQQLPGRSDNNIKNHWNTTKRRVQ
Arabidopsis thaliana	At2g32460	At2R-MYB101	GRGLKKGPWTTTEDAILTEYVRKHGEGNWNAVQKNSGLLRCGKSCRLRWANHLRPNLKKGSFTPDEEKIIIDLHAKLGNKWARMASQLPGRTDNEIKNYWNTRMKRRQ
Arabidopsis thaliana	At4g21440	At2R-MYB102	KNGLKKGPWTSEEDQKLVDYIQKHGYGNWRTLPKNAGLQRCGKSCRLRWTNYLRPDIKRGRFSFEEEETIIQLHSFLGNKWSAIAARLPGRTDNEIKNFWNTHIRKKL
Arabidopsis thaliana	At1g63910	At2R-MYB103	QQKVKRGLWSPEEDEKLIRYITTHGYGCWSEVPEKAGLQRCGKSCRLRWINYLRPDIRRGRFSPEEEKLIISLHGVVGNRWAHIASHLPGRTDNEIKNYWNSWIKKKI
Arabidopsis thaliana	At2g26950	At2R-MYB104	KKTFTKSKWKPEEDRILKDYVIQYGDRTWTHVPKRTGLPHNPASCRFRWMNHLKPSLKKGPFTDEEEKRVLQLHAVLGNKWSQMAREFPGRTDNEIKNFWNARRMRLK
Arabidopsis thaliana	At1g69560	At2R-MYB105	KASVSRGHWRPAEDTKLKELVAVYGPQNWNLIAEKLQGRSGKSCRLRWFNQLDPRINRRAFTEEEEERLMQAHRLYGNKWAMIARLFPGRTDNSVKNHWHVIMARKF
Arabidopsis thaliana	At3g01140	At2R-MYB106	KAGLKKGPWTPEEDQKLLAYIEEHGHGSWRSLPEKAGLQRCGKSCRLRWTNYLRPDIKRGKFTVQEEQTIIQLHALLGNRWSAIATHLPKRTDNEIKNYWNTHLKKRL
Arabidopsis thaliana	At3g02940	At2R-MYB107	ESGLKKGPWTPEEDQKLINHIRKHGHGSWRALPKQAGLNRCGKSCRLRWTNYLRPDIKRGNFTAEEEQTIINLHSLLGNKWSSIAGHLPGRTDNEIKNYWNTHIRKKL
Arabidopsis thaliana	At3g06490	At2R-MYB108	EMDLKRGPWTAEEDFKLMNYIATNGEGRWNSLSRCAGLQRTGKSCRLRWLNYLRPDVRRGNITLEEQLLILELHSRWGNRWSKIAQYLPGRTDNEIKNYWRTRVQKHA
Arabidopsis thaliana	At3g55730	At2R-MYB109	IRSKVKGPWSTEEDAVLTKLVRKLGPRNWSLIARGIPGRSGKSCRLRWCNQLDPCLKRKPFSDEEDRMIISAHAVHGNKWAVIAKLLTGRTDNAIKNHWNSTLRRKY
Arabidopsis thaliana	At3g29020	At2R-MYB110	SRVCSRGHWRISEDTQLMELVSVYGPQNWNHIAESMQGRTGKSCRLRWFNQLDPRINKRAFSDEEEERLLAAHRAFGNKWAMIAKLFNGRTDNALKNHWHVLMARKM
Arabidopsis thaliana	At5g49330	At2R-MYB111	KIGLKRGRWTAEEDEILTKYIQTNGEGSWRSLPKKAGLLRCGKSCRLRWINYLRRDLKRGNITSDEEEIIVKLHSLLGNRWSLIATHLPGRTDNEIKNYWNSHLSRKI
Arabidopsis thaliana	At1g48000	At2R-MYB112	EIEIRRGPWTVEEDMKLVSYISLHGEGRWNSLSRSAGLNRTGKSCRLRWLNYLRPDIRRGDISLQEQFIILELHSRWGNRWSKIAQHLPGRTDNEIKNYWRTRVQKHA
Arabidopsis thaliana	At1g66370	At2R-MYB113	PKGLRKGTWTTEEDILLRQCIDKYGEGKWHRVPLRTGLNRCRKSCRLRWLNYLKPSIKRGKLCSDEVDLVLRLHKLLGNRWSLIAGRLPGRTANDVKNYWNTHLSKKH
Arabidopsis thaliana	At1g66380	At2R-MYB114	SKGLRKGAWTAEEDSLLRQCIGKYGEGKWHQVPLRAGLNRCRKSCRLRWLNYLKPSIKRGKFSSDEVDLLLRLHKLLGNRWSLIAGRLPGRTANDVKNYWNTHLSKKH
Arabidopsis thaliana	At5g40360	At2R-MYB115	KDIIGKGQWTPTEDELLVRMVKSKGTKNWTSIAKMFQGRVGKQCRERWHNHLRPNIKKNDWSEEEDQILIEVHKIVGNKWTEIAKRLPGRSENIVKNHWNATKRRLH
Arabidopsis thaliana	At1g25340	At2R-MYB116	GAEQRKGPWTLEEDTLLTNYISHNGEGRWNLLAKSSGLKRAGKSCRLRWLNYLKPDIKRGNLTPQEQLLILELHSKWGNRWSKISKYLPGRTDNDIKNYWRTRVQKQA
Arabidopsis thaliana	At1g26780	At2R-MYB117	PSVLGRGHWRPAEDVKLKELVSIYGPQNWNLIAEKLQGRSGKSCRLRWFNQLDPRINRRAFTEEEEERLMQAHRLYGNKWAMIARLFPGRTDNSVKNHWHVVMARKY
Arabidopsis thaliana	At3g27780	At2R-MYB118	KASIIKGQWTPEEDKLLVQLVDLHGTKKWSQIAKMLQGRVGKQCRERWHNHLRPDIKKDGWTEEEDIILIKAHKEIGNRWAEIARKLPGRTENTIKNHWNATKRRQH
Arabidopsis thaliana	At5g58850	At2R-MYB119	SKNLIKGQWTAEEDRKLIRLVRQHGERKWAMISEKLEGRAGKQCRERWHNHLRPDIKKDGWSEEEERVLVESHMRIGNKWAEIAKLIPGRTENSIKNHWNATKRRQN
Arabidopsis thaliana	At5g55020	At2R-MYB120	GVILKKGPWTAAEDEILAAYVRENGEGNWNAVQKNTGLARCGKSCRLRWANHLRPNLKKGSFTGDEERLIIQLHAQLGNKWARMAAQLPGRTDNEIKNYWNTRLKRLL
Arabidopsis thaliana	At3g30210	At2R-MYB121	QQGCRKGPWTLEEDKLLAEYVTSHGEGRWSTVAKCAGLNRSGKSCRLRWVNYLRPGLKRGQITPQEEGIILELHSLWGNKWSTIARYLPGRTDNEIKNYWRTHYKKNQ
Arabidopsis thaliana	At1g74080	At2R-MYB122	AEGLKKGAWTQEEDQKLIAYVQRHGEGGWRTLPDKAGLKRCGKSCRLRWANYLRPDIKRGEFSQDEEDSIINLHAIHGNKWSAIARKIPRRTDNEIKNHWNTHIKKCL
Arabidopsis thaliana	At5g35550	At2R-MYB123	REELNRGAWTDHEDKILRDYITTHGEGKWSTLPNQAGLKRCGKSCRLRWKNYLRPGIKRGNISSDEEELIIRLHNLLGNRWSLIAGRLPGRTDNEIKNHWNSNLRKRL
Arabidopsis thaliana	At1g14350	At2R-MYB124	KKERHIVTWSQEEDVILREQITLHGTENWAIIASKFKDKSTRQCRRRWYTYLNSDFKRGGWSPEEDMLLCEAQRVFGNRWTEIAKVVSGRTDNAVKNRFTTLCKKRA

Arabidopsis thaliana	At3g60460	At2R-MYB125	KEEIKKGPWKAEEDEVLINHVKRYGPRDWSSIRSKGLLQRTGKSCRLRWVNKLRPNLKNGCKFSADEERTVIELQSEFGNKWARIATYLPGRTDNDVKNFWSSRQKRLA
Arabidopsis thaliana	AT1G09770	AtCDC5	MRIMIKGGVWKNTEDEILKAAVMKYGKNQWARISSLLVRKSAKQCKARWYEWLDPSIKKTEWTREEDEKLLHLAKLLPTQWRTIAPIVGRTPSQCLERYEKLLDAAC
Arabidopsis thaliana	AT4G32730.1	At3R-MYB1	ARRSTKGQWTPEEDEVLCKAVERFQGKNWKKIAECFKDRTDVQCLHRWQKVLNPELVKGPWSKEEDNTIIDLVEKYGPKKWSTISQHLPGRIGKQCRERWHNHLNPGINKNAWTQEEELTLIRAHQIYGNKWAELMKFLPGRSDNSIKNHWNSSVKKKL
Arabidopsis thaliana	AT4G00540.1	At3R-MYB2	TRRSTKGGWTAEEDQILTNVVKKYQGRNWKRIAECLPGSEENRRNDVQCQHRWLKVLDPSLQKGAWKKEEDELLSELVKDYMENDRPPWSKISKELPGRIGKQCRERWHNHLNPTIIKSPWTREEELILVQAQRGNGNKWAEIAKLLPGRTENNIKNHWNCSVKKRL
Arabidopsis thaliana	AT3G09370.1	At3R-MYB3	PIRRAKGGWTPEEDETLRQAVDTFKGKSWKNIAKSFPDRTEVQCLHRWQKVLNPDLIKGPWTHEEDEKIVELVEKYGPAKWSIIAQSLPGRIGKQCRERWHNHLNPDINKDAWTTEEEVALMNAHRSHGNKWAEIAKVLPGRTDNAIKNHWNSSLKKKS
Arabidopsis thaliana	AT5G11510.1	At3R-MYB4	ARRSTRGQWTAEEDEILRKAVHSFKGKNWKKIAEYFKDRTDVQCLHRWQKVLNPELVKGPWTKEEDEMIVQLIEKYGPKKWSTIARFLPGRIGKQCRERWHNHLNPAINKEAWTQEEELLLIRAHQIYGNRWAELTKFLPGRSDNGIKNHWHSSVKKKL
Arabidopsis thaliana	AT5G02320.1	At3R-MYB5	PMRRAKGGWTPEEDETLRRAVEKYKGKRWKKIAEFFPERTEVQCLHRWQKVLNPELVKGPWTQEEDDKIVELVKKYGPAKWSVIAKSLPGRIGKQCRERWHNHLNPGIRKDAWTVEEESALMNSHRMYGNKWAEIAKVLPGRTDNAIKNHWNSSLKKKL
Populus trichocarpa v3.0	Potri.001G139900.1	Pt2R-MYB001	GSDFKKGPWTPEEDEKLVDYIKRNGHENWKALPKLAGLNRCGKSCRLRWTNYLRPDIKRGKFSEEEERVIVNLHSVLGNKWSRIANHLPGRTDNEIKNFWNTHIRKKL
Populus trichocarpa v3.0	Potri.001G005100.1	Pt2R-MYB002	KVGLKRGPWTPEEDELLVNYIKKEGEGRWRTLPKKAGLLRCGKSCRLRWMNYLRPSVKRGQIADDEEDLILRLHRLLGNRWSLIAGRIPGRTDNEIKNYWNTHLSKKL
Populus trichocarpa v3.0	Potri.001G036000.1	Pt2R-MYB003	GGVLKKGPWTSAEDAILIEYVKKHGEGNWNAVQKHSGLFRCGKSCRLRWANHLRPNLKKGAFTQEEEQLIIELHAKMGNKWARMAAHLPGRTDNEIKNYWNTRIKRHQ
Populus trichocarpa v3.0	Potri.001G118800.1	Pt2R-MYB004	KLGVKKGPWTAEEDKKLINFILTNGQCCWRAVPKLAGLRRCGKSCRLRWTNYLRPDLKRGLLTEAEEQLVIDLHARLGNRWSKIAARLPGRTDNEIKNHWNTHIKKKL
Populus trichocarpa v3.0	Potri.001G099800.1	Pt2R-MYB005	QQKVKRGLWSPEEDEKLIRYITTHGYGCWSEVPEKAGLQRCGKSCRLRWINYLRPDIRRGRFTPEEEKLIISLHGVVGNRWAHIASHLPGRTDNEIKNYWNSWIKKKI
Populus trichocarpa v3.0	Potri.001G086700.1	Pt2R-MYB006	KVGLYRGPWTTKEDTLLINYIQAHGEGHWRSLPKKAGLLRCGKSCRLRWMNYLRPDIKRGNITPDEDDLIIRLHSLLGNRWSLIAGRLPGRTDNEIKNYWNSHLSKRL
Populus trichocarpa v3.0	Potri.001G075400.1	Pt2R-MYB007	KQKVKRGLWSPEEDEKLIKYITIHGHGSWSSVPNLAGLERCGKSCRLRWINYLRPDLKRGSITAKEERIIVDTHRILGNKWAQIAKHLPGRTDNEVKNFWNSCIKKKL
Populus trichocarpa v3.0	Potri.001G169600.1	Pt2R-MYB008	MPNKGAWTAEEDRKLAEVIATHGAKRWRTIASKAALNRCGKSCRLRWLNYLRPNIKRGNISDQEEDLILRLHKLLGNRWSLIAGRLPGRTDNEIKNYWNSHLSKKI
Populus trichocarpa v3.0	Potri.001G197000.1	Pt2R-MYB009	KQKVKRGLWSPEEDEKLANYISTYGHGCWSSVPKLAGLQRCGKSCRLRWINYLRPDLKRGSFSPQEAALIIELHSILGNRWAQIAKHLPGRTDNEVKNFWNSSIKKKL
Populus trichocarpa v3.0	Potri.001G224500.1	Pt2R-MYB010	ITPLKKGPWTSAEDAILIDYVKKHGEGNWNSVQKHSGLFRCGKSCRLRWANHLRPDLKKGSFTPEEENRIIELHANMGNKWARMAAEVGNLIPDLKCVRIHNVVWEEEIGRRGEIGSSGEKKLPGRTDNEIKNYWNTRTKRLQ
Populus trichocarpa v3.0	Potri.001G235500.1	Pt2R-MYB011	QDEIRKGPWTEKEDILLINFVHLFGDRRWDSIAKVSGLNRTGKSCRLRWVNYLHPGLKRGKMTPQEERLVLELHAKWGNRWSRIARKLPGRTDNEIKNYWRTHMRKKA
Populus trichocarpa v3.0	Potri.001G250000.1	Pt2R-MYB012	QWTEEEDRKLIRLVKQFGVRKWAQIAEKLDGRAGKQCRERWHNHLRPDIKVSSPFTSSRKDSWSEEEERIMVGAHAKVGNRWAEIAKLIPGRTENAIKNHWNATKRRQN
Populus trichocarpa v3.0	Potri.001G258700.1	Pt2R-MYB013	SNKFKKGLWSPEEDDKLMNYILNNGQGCWSDVARNAGLQRCGKSCRLRWINYLRPDLKRGAFSPQEEEMTIHLHSLLGNRWSQIAARLPGRTDNEIKNFWNSTIKKRL
Populus trichocarpa v3.0	Potri.001G267300.1	Pt2R-MYB014	RAKLRKGLWSPEEDEKLIKYMLTNGQGCWSEIARNAGLQRCGKSCRLRWINYLRPDLKRGAFSPQEEELIIHLHSILGNRWSQIAARLPGRTDNEIKNFWNSTLKKRF
Populus trichocarpa v3.0	Potri.001G300200.1	Pt2R-MYB015	MDCDRVKGPWSPEEDALLKHLVIKHGPRSWTMIARAVPGRSGKSCRLRWCNQLSPVVEHRAFTREEDVIIINAHIKYGNKWAAIARLLDGRTDNAIKNHWNSKLKRKY
Populus trichocarpa v3.0	Potri.001G347200.1	Pt2R-MYB016	KSQVIKGQWTPQEDRLLVQSVKQYGIKKWSQIAKMLEGRVGKQCRERWHNHLRPDIKKDAWSEEEDELLINAHREIGNRWAEIAKRLPGRTENTIKNHWNATKRRQF
Populus trichocarpa v3.0	Potri.001G336700.1	Pt2R-MYB017	KVGLKRGPWTIEEDHKLTNFILNNGIQCWRMVPKLAGLLRCGKSCRLRWINYLRPDLKRGGFTKMEDNQIIQLHSRLGNRWSKIASHFPGRTDNEIKNHWNTRIKKKL
Populus trichocarpa v3.0	Potri.001G408700.1	Pt2R-MYB018	KNGLKKGPWTEEEDKKLISYIQKHGHGRWRLLPKNAGLKRCGKSCRLRWTNYLRPDIKRGKLSFEEEETIIQLHSVLGNKWSAIATRLPGRTDNEIKNYWNTHIRKRL
Populus trichocarpa v3.0	Potri.001G470500.1	Pt2R-MYB019	KDNVKRGQWTPEEDNKLSSYIAQHGTRNWRLIPKNAGLQRCGKSCRLRWTNYLRPDLKHGQFSDAEEQTIVKLHSVVGNRWSLIAAQLTGRTDNDVKNHWNTKLKKKL
Populus trichocarpa v3.0	Potri.002G038500.1	Pt2R-MYB020	KMGLKRGPWTAEEDRILINHIQLYGHGNWRALPKQAGLLRCGKSCRLRWINYLRPDIKRGNFSREEEDTIIKLHEMLGNRWSAIAARLPGRTDNEIKNVWHTHLKKRL
Populus trichocarpa v3.0	Potri.002G073500.1	Pt2R-MYB021	STACPRGHWRPAEDDKLRQLVEQYGAQNWNDIAEKLQGRSGKSCRLRWFNQLDPRINRRPFSEEEEERLLAAHQVHGNKWALIARVFPGRTDNAVKNHWHVIMARKQ
Populus trichocarpa v3.0	Potri.002G096800.1	Pt2R-MYB022	EIGLKKGPWTPEEDEKLVDHIKKHGQGSWRALPKLAGLNRCGKSCRLRWTNYLRPDIKRGKFSQDEEQTILHLHSIHGNKWSAIATHLPGRTDNEIKNFWNTHLKKKL
Populus trichocarpa v3.0	Potri.002G113700.1	Pt2R-MYB023	KANVKKGPWSPEEDAKLKSYIEQHGTGGNWIALPQKIGLKRCGKSCRLRWLNYLRPNIKHGGFSEEEDNIICSLYISIGSRWSIIAAQLPGRTDNDIKNYWNTRLKKKL
Populus trichocarpa v3.0	Potri.002G122600.1	Pt2R-MYB024	DVDRIKGPWSPEEDEALQRLVQTYGPRNWSLISKSIPGRSGKSCRLRWCNQLSPEVEHRPFSAEEDDAIIRAHARFGNKWATIARLLNGRTDNAIKNHWNSTLKRKC
Populus trichocarpa v3.0	Potri.002G128900.1	Pt2R-MYB025	KSARIKGPWSSEEDMILTGLVERHGPKNWSLISRYIKGRSGKSCRLRWCNQLSPNVEHRPFSPAEDEAILVAHARYGNRWATIARLLPGRTDNAVKNHWNSTLKRRA
Populus trichocarpa v3.0	Potri.002G140900.1	Pt2R-MYB026	KEEIRKGPWKAEEDEVLIMHVKKYGPRDWSSIRSKGLLQRTGKSCRLRWVNKLRPNLKHGCKFSAEEERVVIDLQAEFGNKWARIATYLPGRTDNDVKNFWSSRQKRLA
Populus trichocarpa v3.0	Potri.002G173900.1	Pt2R-MYB027	KVGLRRGPWTPREDALLTEYIQAHGEGHWRSLPKKSGLLRCGKSCRLRWMNYLRPDIKRGNITPDEDDLIIRMHSLLGNRWSLIAGRLPGRTDNEIKNHWNTHLSKRL
Populus trichocarpa v3.0	Potri.002G185900.1	Pt2R-MYB028	KQKLRKGLWSPEEDEKLLSHITKYGHGCWSSVPKQAGLQRCGKSCRLRWINYLRPDLKRGTFSHLEENLIIELHAVLGNRWSQIAAQLPGRTDNEIKNLWNSCLKKKL
Populus trichocarpa v3.0	Potri.002G191800.1	Pt2R-MYB029	GMGIRKGPWTIEEDSLLAHYITIHGEGHWNSAARCAGLKRTGKSCRLRWLNYLRPNVRRGNITLQEQLLILQLHSRWGNRWSKIAQMLPGRTDNEIKNYWRTRVQKQA
Populus trichocarpa v3.0	Potri.002G198100.1	Pt2R-MYB030	KVGLKKGRWTAEEDEKLAKYIQANGEGSWRSMPKNAGLLRCGKSCRLRWINYLRADLKRGNISTEEEEIIVQLHASLGNRWSLIASYLPGRTDNEIKNYWNSHLSRRI
Populus trichocarpa v3.0	Potri.002G228700.1	Pt2R-MYB031	NQGLKKGPWTVAEDAILIEYVKKHGEGNWNSVQKNFRLMRCGKSCRLRWANHLRPNLKKGSFTPDEEKIIIELHAKHGNKWARMASQLPGRTDNEIKNYWNTRMKRHQ
Populus trichocarpa v3.0	Potri.003G064600.1	Pt2R-MYB032	TNMPNKGAWTAEEDRKLAEVIAIHGARKWKTIAAKAALNRCGKSCRLRWLNYLRPNIKRGNISDQEEDLILRLHKLLGNRWSLIAGRLPGRTDNEIKNYWNSHLSKKI
Populus trichocarpa v3.0	Potri.003G079100.1	Pt2R-MYB033	KKVMSKGAWTAEEDNKLAHCVEVHGAKRWKTVALKSGLNRCGKSCRLRWMNYLRPNIKRGNISCDEEDLIIRLHKLLGNRWSLIAGRLPGRTDNEIKNYWNSHLSKKI
Populus trichocarpa v3.0	Potri.003G094200.1	Pt2R-MYB034	ASALKKGPWTPDEDEKLIDYIKRNGHENWKALPMLAGLNRCGKSCRLRWTNYLRPDIKRGKFSEEEERVIVNLHSALGNKWSRIANHLPGRTDNEIKNFWNTHIRKKL
Populus trichocarpa v3.0	Potri.003G114100.1	Pt2R-MYB035	KLGVKKGPWTAEEDKKLINFILTNGQCCWRAVPKLAGLRRCGKSCRLRWTNYLRPDLKRGLLTEAEEQLVIDLHARLGNRWSKIAARLPGRTDNEIKNHWNTHIKKKL
Populus trichocarpa v3.0	Potri.003G132000.1	Pt2R-MYB036	QQKVKRGLWSPEEDEKLIRYITTHGYGCWSEVPEKAGLQRCGKSCRLRWINYLRPDIRRGRFTPEEEKLIISLHGVVGNRWAHIASHLPGRTDNEIKNYWNSWIKKKI
Populus trichocarpa v3.0	Potri.003G144200.1	Pt2R-MYB037	KVGLQRGPWTTREDTLLINYIQAHGEGHWRSLPKKAGLLRCGKSCRLRWMNYLRPDIKRGNITPDEDDLIIRLHSLLGNRWSLIAGRLPGRTDNEIKNYWNSHLSKRV

Populus trichocarpa v3.0	Potri.003G144300.1	Pt2R-MYB038	KVGLHRGPWTTREDALLVNYIQKHGEGHWRSLPNKAGLLRCGKSCRLRWLNYLRPDIKRGNITPEEDDLIIRLHSLLGNRWSLIAGRLPGRTDNEIKNYWNSHLSKRL
Populus trichocarpa v3.0	Potri.003G155700.1	Pt2R-MYB039	KRKVKRGLWSPDEDEKLIKYITTHGHGSWSSVPKLAGLQRCGKSCRLRWINYLRPDLKRGSFTAQEESIIVEIHRMLGNRWAQIAKHLPGRTDNEVKNFWNSCIKKKL
Populus trichocarpa v3.0	Potri.003G189700.1	Pt2R-MYB040	GVGLKKGPWTSAEDAILIEYVKKHGEGNWNSVQKHSGLFRCGKSCRLRWANHLRPNLKKGAFTHEEEQLIIELHAKMGNKWARMAAHLPGRTDNEIKNYWNTRIKRRQ
Populus trichocarpa v3.0	Potri.003G219900.1	Pt2R-MYB041	KVGLKRGPWTPEEDELLANYIKKEGEGRWRTLPKKAGLLRCGKSCRLRWMNYLRPSVKRGQISPDEEDLILRLHRLLGNRWSLIAGRIPGRTDNEIKNYWNTHLSKKL
Populus trichocarpa v3.0	Potri.004G026600.1	Pt2R-MYB042	NLPLKRGAWSPEEDQKLIAYINSHGIRNWIEMPKAAGLLRSGKSCRLRWMNYLRPDIKRGNFSMEEVETILKLHGILGNRWSAIAAKLPGRTDNEIKNFWNTRLKKNY
Populus trichocarpa v3.0	Potri.004G033100.1	Pt2R-MYB043	KNGLKKGPWTPEEDQKLVDYIQKHGYGNWRTLPKNAGLQRCGKSCRLRWTNYLRPDIKRGRFSFEEEETIIQLHSILGNKWSAIAARLPGRTDNEIKNYWNTHIRKRL
Populus trichocarpa v3.0	Potri.004G086300.1	Pt2R-MYB044	KVGLKKGPWTSDEDKKLINFILANGQCCWRAVPKLAGLLRCGKSCRLRWTNYLRPDLKRGLLSEYEEKMVIDLHAQLGNRWSKIASHLPGRTDNEIKNHWNTHIKKKL
Populus trichocarpa v3.0	Potri.004G088100.1	Pt2R-MYB045	KQGNNKGAWSTQEDQKLIDYIQTHGEGCWRSLPEAAGLHRCGKSCRLRWINYLRPDIKRGNFGQDEEDLIIKLHALLGNRWALIAGRLPGRTDNEVKNYWNSHLKKKL
Populus trichocarpa v3.0	Potri.004G102600.1	Pt2R-MYB046	MKERQRWQPEEDAVLRAYVKQYGPKEWNLISQRVEATGKTLNRDPKSCLERWKNYLKPGIKKGSLIPEEQTLVISLQAKYGNKWKTIASEVPGRTAKRLSKWWEVFKERQS
Populus trichocarpa v3.0	Potri.T125000.1	Pt2R-MYB047	EQGWRKGPWTPEEDKLLSEYVSSNGEGRWSSVSRCSVLNFGGKSCRLRWVNYLRPGLKRGQITPQEEGIIIELHALWGNKWSTIARYLPGRTDNEIKNYWRTHFKKKD
Populus trichocarpa v3.0	Potri.004G118000.1	Pt2R-MYB048	EQGWRKGPWTPEEDKLLSEYVSLNGEGRWSSVSRCSGLNRSGKSCRLRWVNYLRPGLKRGQITPQEEGIIIELHALWGNKWSTIARYLPGRTDNEIKNYWRTHFKKKD
Populus trichocarpa v3.0	Potri.004G126700.1	Pt2R-MYB049	KIGVMKGPWTPEEDIILVSYIQEHGPGNWRAVPTNTGLLRCSKSCRLRWTNYLRPGIKRGNFTHNEEKMIIHLQALLGNRWAAIASYLPQRTDNDIKNYWNTHLKKLK
Populus trichocarpa v3.0	Potri.004G138000.1	Pt2R-MYB050	KEHTNKGAWTKEEDERLVNYIKAQGEGCWRSLPKAAGLLRCGKSCRLRWINYLRPDLKRGNFSDEEDELIINLHSLLGNKWSLIAARLPGRTDNEIKNYWNTHIKRKL
Populus trichocarpa v3.0	Potri.004G174400.1	Pt2R-MYB051	KAHTNKGAWTKEEDDRLIAYIRTHGEGCWRSLPKAAGLLRCGKSCRLRWINYLRPDLKRGNFTEEEDELIIKLHSLLGNKWSLIAGRLPGRTDNEIKNYWNTHIRRKL
Populus trichocarpa v3.0	Potri.004G215100.1	Pt2R-MYB052	KANVKKGPWSPEEDSKLKEYIEKYGTGGNWIALPQKAGLKRCGKSCRLRWLNYLRPNLKHGEFSDEEDRVICSLFASIGSRWSIIAAQLPGRTDNDIKNYWNTKLKKKL
Populus trichocarpa v3.0	Potri.005G001600.1	Pt2R-MYB053	KQKLRKGLWSPEEDEKLLNYITKHGHGCWSSVPKQADLQRCGKSCRLRWINYLRPDLKRGAFSQQEENLIIELHAVLGNRWSQIAAQLPGRTDNEIKNLWNSCIKKKL
Populus trichocarpa v3.0	Potri.005G063200.1	Pt2R-MYB054	RTSCHRGHWRPAEDEKLRQLVDQYGPHNWNFIAEHLQGRSGKSCRLRWYNQLDPNINKRPFTEEEEERLLRAHHIQGNRWASIARLFPGRTDNAVKNHYHVVMARRK
Populus trichocarpa v3.0	Potri.005G074500.1	Pt2R-MYB055	ESGLKKGPWTPEEDQKLVKYIQKHGHGSWRALPKLAGLNRCGKSCRLRWTNYLRPDIKRGKFSQEEEQTILNLHSILGNKWSAIAGHLPGRTDNEIKNFWNTHLKKKL
Populus trichocarpa v3.0	Potri.005G096600.1	Pt2R-MYB056	KTQVMRGPWSPAEDLRLITFIQKHGHENWRALPKQAGLLRCGKSCRLRWINYLRPDVKRGNFSKEEEDTIIKLHQTLGNKWSKIASHFPGRTDNEIKNVWNTHLKKRL
Populus trichocarpa v3.0	Potri.005G112000.1	Pt2R-MYB057	RAHTNKGAWTREEDKRLVAYIQAHGEGCWRSLPKSAGLLRCGKSCRLRWINYLRPDLKRGNFTEEEDELIIKLHSLLGNNNKVFILLSRWSLIAGRLPGRTDNEIKNYWNTHIRKKL
Populus trichocarpa v3.0	Potri.005G118500.1	Pt2R-MYB058	PINLVKGQWTAEEDSLLIRLVDEFGIRKWSHIAQIFPGRIGKQCRERWHNHLRPDIKKDIWSEEEDRVLIQAHKEIGNKWAEIAKSLPGRTENSIKNHWNATKRRQT
Populus trichocarpa v3.0	Potri.017G126000.1	Pt2R-MYB059	SSGIRKGAWTREEDILLRDCVEKYGEGRWHQVSSKAGLNRCRKSCRLRWLNYLKPGIKRGQYSEDEEDLIIKLHRLLGNRQVKMWSLIAGRLPGRTANDLKNYWNTNLSKKV
Populus trichocarpa v3.0	Potri.017G125900.1	Pt2R-MYB060	SSFVRKGAWTEEEDILLRKCVEKYGEGRWCQIPLKAGLNRCRKSCRMRWLNYLKPNVKRGQFSVGEVDLIIRLHKLLGNRQVKMWSLIAGRLPGRTANDVKNYWNTNLRKKV
Populus trichocarpa v3.0	Potri.017G125800.1	Pt2R-MYB061	MVSSLGVRKGAWTEEEDILLRKCVEKYGEGRWCQIPLQAGLNRCRKSCRMRWLNYLKPNVNRGQFSVGEVDLIIRLHKLLGNRQVKMWSLIAGRLPGRTANDVKNYWNTNLRKKV
Populus trichocarpa v3.0	Potri.017G125700.1	Pt2R-MYB062	MVSLLGVRKGAWTEEEDILLRKCVEKYGEGRWHQVPSKTGLNRCRKSCRLRWLNYLKPNIKRGQFSVDEVDLIIRLHKLLGNRQVQMWSLIAGRLPGRTANDVKNYWNTNLRKKV
Populus trichocarpa v3.0	Potri.017G125600.1	Pt2R-MYB063	SLGVRKGAWTEEEDILLRKCVEKYGEGRWHEVPSRAGLNRCRKSCRMRWLNYLKPNVKRGQFSVDEVDLIIRLHKLLGNRQVKMWSLIAGRLSGRTANDVKNYWNSNQRKKV
Populus trichocarpa v3.0	Potri.005G164900.1	Pt2R-MYB064	ENGLKKGPWTPEEDQKLVDYIQKHGHGSWRALPKLADLNRCGKSCRLRWTNYLRPDIKRGKFSQDEEQTILHLHSIIGNKWSTIATHLPGRTDNEIKNFWNTHLKKKL
Populus trichocarpa v3.0	Potri.005G142600.1	Pt2R-MYB065	EMDRIKGPWSPEEDEALKKLVQRHGPRNWSLISKSIPGRSGKSCRLRWCNQLSPQVEHRAFTPEEDDRIIRAHARFGNKWATIARLLNGRTDNAIKNHWNSTLKRKC
Populus trichocarpa v3.0	Potri.005G186400.1	Pt2R-MYB066	STACPRGHWRPAEDDKLRQLVEQYGAQNWNSIAEKLQGRSGKSCRLRWFNQLDPRINRRPFSEEEEERLLAAHRIHGNKWALIARLFPGRTDNAVKNHWHVIMARRQ
Populus trichocarpa v3.0	Potri.005G224100.1	Pt2R-MYB067	KMGLKKGPWTAEEDQILINYIQLHGHGNWRALPKQAGLLRCGKSCRLRWINYLRPDIKRGNFSREEEDTIIKLHEMLGNRWSAIAARLPGRTDNEIKNVWHTHLKKRL
Populus trichocarpa v3.0	Potri.006G066400.1	Pt2R-MYB068	PHEFKKGLWKPEEDLILKTYVETHGEGNWSTVSKRSGLMRCGKSCRLRWKNYLRPNIRRGGMSQDEEDMIIRMHKLLGNRWSLIAGRLPGRTDNDVKNYWNTHLNKRC
Populus trichocarpa v3.0	Potri.006G085900.1	Pt2R-MYB069	MKERQRWRAEEDALLRAYVKQYGPREWNLVSQRMNTPLNRDAKSCLERWKNYLKPGIKKGSLTEEEQSLVIRLQAKHGNKWKKIAAEVPGRTAKRLGKWWEVFKEKQQ
Populus trichocarpa v3.0	Potri.006G122100.1	Pt2R-MYB070	GDKLRKGSWQEEEDERLTASATLLGERKWDSIARLSGLMRSGKSCRMRWLNYLQPNLKRGHISAEEEQIIIQFHGQWGNKWARIARRLPGRTDNEIKNYWRTHMRKEI
Populus trichocarpa v3.0	Potri.006G123400.1	Pt2R-MYB071	KANMKKGPWSPEEDAKLKEYLEKQGTGGNWIALPQKAGLKRCGKSCRLRWLNYLRPNIKHGEFSDDEDRIICSLYANIGSRWSIIAAQLPGRTDNDIKNYWNTKLKKKL
Populus trichocarpa v3.0	Potri.006G170800.1	Pt2R-MYB072	KNNVKRGPWSPEEDAKLKAYIDHFGTGGNWIALPQKIGLKRCGKSCRLRWLNYLRPNIKHGGFSEEEDNIICNLYISIGSRWSVIAAQLPGRTDNDIKNYWNTRLKKKL
Populus trichocarpa v3.0	Potri.006G221200.1	Pt2R-MYB073	KKEANRGAWTAEEDQKLAQVIEIHGPKRWRSVAAKAGLNRCGKSCRLRWMNHLRPNIKRGNISDQEEDLIVRLHKLLGNRWSLIAGRLPGRTDNEIKNYWNSHLSKKI
Populus trichocarpa v3.0	Potri.006G221500.1	Pt2R-MYB074	TGGIIRGTWTATEDKILTAYVRNYGEGNWARVPKETGLKRCGKSCRLRWLNYLKPDVKRGNISPDEEDLIIRLHKLLGNRWALIAGRIPGRTDNEIKNYWNSTLKRKV
Populus trichocarpa v3.0	Potri.006G221800.1	Pt2R-MYB075	KEGLNRGAWTALEDKILTAYIKAHGEGKWRNLPKRAGLKRCGKSCRLRWLNYLRPDIKRGNISNDEEELIVRLHKLLGNRWSLIAGRLPGRTDNEIKNYWNTTLGKKA
Populus trichocarpa v3.0	Potri.006G234200.1	Pt2R-MYB076	KANVKRGPWSPEEDATLKSYLETHGTGGNWIALPQKAGLKRCGKSCRLRWLNYLRPDIKHGGFTEEEDNIICTLYSQMGSRWSLIAAQLPGRTDNDVKNYWNTKLKKKI
Populus trichocarpa v3.0	Potri.006G275900.1	Pt2R-MYB077	ADGMNKGAWTPLEDEMLVDYVKIHGEGKWSNIVKETGLKRCGKSCRLRWMNYLRPDIKRGNISDDEEDLIIRLHKLLGNRWSLIAGRLPGRTDNEIKNYWHTNIAKKA
Populus trichocarpa v3.0	Potri.007G134500.1	Pt2R-MYB078	MSSRGHWRLAEDEKLLELVEQYGPHNWNSIAEKLQGRSGKSCRLRWFNQLDPRINRSPFTEEEEERLLACHRIHGNKWAVIAKQFPGRTDNAVKNHWHVIMARRC
Populus trichocarpa v3.0	Potri.007G106100.1	Pt2R-MYB079	KTSCHRGHWRPAEDEKLRQLVDQYGPQNWNFIAEHLQGRSGKSCRLRWYNQLDPNINKRPFTEDEEERLLKAHQIHGNRWASISRLFPGRTDNAVKNHYHVVMARRK
Populus trichocarpa v3.0	Potri.007G093900.1	Pt2R-MYB080	ESGLKKGPWTSEEDQKLVKYIQKHGHGSWRALPKLSGLNRCGKSCRLRWTNYLRPDIKRGKFSQEEEQTILNLHSVLGNKWSAIASHLPGRTDNEIKNFWNTHLKKKL
Populus trichocarpa v3.0	Potri.007G067600.1	Pt2R-MYB081	KSQVKRGPWSPAEDLRLIAFIQKHGHENWRALPKQAGLLRCGKSCRLRWINYLRPDVKRGNFSEVEEDTIIKLHQTLGNKWSKIASHLPGRTDNEIKNVWNTHLKKKL

Populus trichocarpa v3.0	Potri.007G048900.1	Pt2R-MYB082	EMDRIKGPWSPEEDEALQKLVQKHGARNWSLISKSIPGRSGKSCRLRWCNQLSPQVEHRPFTPDEDDTIIRAHARFGNKWATIARLLYGRTDNAIKNHWNSTLKRKC
Populus trichocarpa v3.0	Potri.007G007900.1	Pt2R-MYB083	KANVKKGPWSPEEDAILKAYIEQHGTGGNWIALPQKIGLKRCGKSCRLRWLNYLRPNIKHGGFSEEEDNIICSLYISIGSRWSIIAAQLPGRTDNDIKNYWNTRLKKKL
Populus trichocarpa v3.0	Potri.008G070900.1	Pt2R-MYB084	GDHRIKGSWSPQEDATLIKLVEQHGPRNWSMISTGIPGRSGKSCRLRWCNQLSPEVQHRPFTPAEDAKIVQAHAIHGNKWATIARLLPGRTDNAIKNHWNSTLRRKR
Populus trichocarpa v3.0	Potri.008G081600.1	Pt2R-MYB085	KQKVKRGLWSPEEDEKLVKYITSHGHGSWSSVPKFAGLKRCGKSCRLRWINYLRPELKRGSFSAEEEQIIIDVHRILGNRWAQIAKHLPGRTDNEVKNFWNSCIKKKL
Populus trichocarpa v3.0	Potri.008G088000.1	Pt2R-MYB086	SKLCTRGHWRPAEDTQLKKLVALYGPQNWNLIAEKLEGRSGKSCRLRWFNQLDPRINRKAFTEEEEERLMQAHRLYGNKWAMIARLFPGRTDNAVKNHWHVIMARKY
Populus trichocarpa v3.0	Potri.008G089200.1	Pt2R-MYB087	KVGLKKGPWTPEEDQKLLDYIEEHGHGSWRALPAKAGLQRCGKSCRLRWTNYLRPDIKRGKFNLQEEQSIIRLHALLGNRWSAIATHLPKRTDNEIKNYWNTHLKKRL
Populus trichocarpa v3.0	Potri.008G089700.1	Pt2R-MYB088	KVGLKKGPWTPEEDQKLLAYIEEHGHGSWRALPAKAGLRRCGKSCRLRWTNYLRPDIKRGKFNLQEEQSIIQLHALLGNRWSAIATHLPKRTDNEIKNYWNTHLKKRL
Populus trichocarpa v3.0	Potri.008G101400.1	Pt2R-MYB089	MGDLRRGPWTVEEDFKLINYIATHGEGRWNSLARCAGLKRTGKSCRLRWLNYLRPDVRRGNITLEEQLMILELHSRWGNRWSKIAQHLPGRTDNEIKNYWRTRVQKHA
Populus trichocarpa v3.0	Potri.008G122100.1	Pt2R-MYB090	ESELRRGPWTLEEDTLLTHYITRHGEGRWNMLARGAGLKRTGKSCRLRWLNYLKPDIKRGNLTPQEQLLILELHSKWGNRWSKIAQHLPGRTDNEIKNYWRTKVQKEA
Populus trichocarpa v3.0	Potri.008G128500.1	Pt2R-MYB091	KQAKNQGAWSKQEDQKLIDYIRKHGEGCWSYLPQAAGLLRCGKSCRLRWINYLRPDLKRGNFAEDEEDLIIKLHALLGNRWSLIAGRLPGRTDNEVKNYWNSHLRRKL
Populus trichocarpa v3.0	Potri.008G148400.1	Pt2R-MYB092	KKERHIVTWTQQEDDILRQQISQHGTENWAIIASKFKDKTTRQCRRRWYTYLNSDFKKGGWSPEEDLLLCEAQKIFGNRWTEIAKVVSGRTDNAVKNRFSTLCKKRA
Populus trichocarpa v3.0	Potri.008G166700.1	Pt2R-MYB093	KMGLKRGPWTPEEDQILISYVQKYGHSNWRALPKQAGLQRCGKSCRLRWVNYLRPDIKRGNFSKEEEEAIIKLHEILGNRWSAIASRLPGRTDNEIKNVWHTHLLKRL
Populus trichocarpa v3.0	Potri.008G173400.1	Pt2R-MYB094	EEGWRKGPWTAEEDRLLIEYVRLHGDGRWSSVARLAGLKRNGKSCRLRWVNYLRPDLKRGQITPHEESIIVELHARWGNRWSTIARSLPGRTDNEIKNYWRTHFKKKA
Populus trichocarpa v3.0	Potri.009G007100.1	Pt2R-MYB095	KANVKKGPWSPEEDSKLKEYIEKYGTGGNWIALPQKAGLKRCGKSCRLRWLNYLRPNIKHGEFSDEEDRVICSLFANIGSRWSIIAAQLPGRTDNDIKNYWNTKLKKKL
Populus trichocarpa v3.0	Potri.009G018700.1	Pt2R-MYB096	MIPLKKGPWTSAEDAILIDYVKKHGEGNWNAVQKHSGLFRCGKSCRLRWANHLRPDLKKGAFTPEEENRIIELHATMGNKWARMAAELPGRTDNEIKNYWNTRIKRLQ
Populus trichocarpa v3.0	Potri.009G027300.1	Pt2R-MYB097	QDETRKGPWTEQEDILLINFVNLFGDRRWDFIAKVSGLNRTGKSCRLRWVNYLHPGLKRGKMTPQEERLVLELHARWGNRWSRIARKLPGRTDNEIKNYWRTHTRKKA
Populus trichocarpa v3.0	Potri.009G044100.1	Pt2R-MYB098	CATLIKGQWTEEEDRKLIRLVKQFGVRKWAQIAERVAGRAGKQCRERWHNHLRPDIKKDSWSEEEEIILVEAHTKVGNRWAEIAKLIPGRTENAIKNHWNATKRRQN
Populus trichocarpa v3.0	Potri.009G053900.1	Pt2R-MYB099	INKFRKGLWSPEEDDKLMNYMLNNGQGCWSDVARNAGLQRCGKSCRLRWINYLRPDLKRGAFSPQEEEMIIHLHSLLGNRWSQIAARLPGRTDNEIKNFWNSTIKKRL
Populus trichocarpa v3.0	Potri.009G061500.1	Pt2R-MYB100	KAKLRKGLWSPEEDEKLIKYMLTNGQGCWSDIARNAGLQRCGKSCRLRWINYLRPDLKRGAFSPQEEELIIHLHTILGNRWSQIAARLPGRTDNEIKNFWNSTLKKRL
Populus trichocarpa v3.0	Potri.009G096000.1	Pt2R-MYB101	DNNRVKGPWSPEEDDLLKHLVIKHGPRNWTMIARAVPGRSGKSCRLRWCNQLSPEVEHRAFTREEDEIIINAHAKYGNKWATIARLLDGRTDNAIKNHWNSTLKRKY
Populus trichocarpa v3.0	Potri.009G134000.1	Pt2R-MYB102	KAHTNKGAWTKEEDDRLVAYIRAHGEGCWRSLPKAAGLLRCGKSCRLRWINYLRPDLKRGNFTEAEDELIIKLHSLLGNKWSLIAGRLPGRTDNEIKNYWNTHIRRKL
Populus trichocarpa v3.0	Potri.010G064000.1	Pt2R-MYB103	EEGWRKGPWTAEEDRLLVEYVRLHGEGRWNSVARLAGLKRNGKSCRLRWVNYLRPDLKRGQITPHEERIIVELHARWGNRWSTIARSLPGRTDNEIKNYWRTHFKKKA
Populus trichocarpa v3.0	Potri.010G093000.1	Pt2R-MYB104	KKERHIVTWTQLEDDILRQQISLNGTENWAIIASKFKDKTTRQCRRRWYTYLNSDFKKGGWSPEEDMLLCEAQKIFGNRWTEIAKVVSGRTDNAVKNRFSTLCKKRA
Populus trichocarpa v3.0	Potri.010G114000.1	Pt2R-MYB105	KQDTNKGAWSKEEDQKLIDYIRKHGEGCWRSLPQAAGLLRCGKSCRLRWINYLRPDLKRGNFGEDEEDLIIKLHALLGNRWSLIAGRLPGRTDNEVKNYWNSHLRRKL
Populus trichocarpa v3.0	Potri.010G123000.1	Pt2R-MYB106	EGELRRGPWTLEEDTLLTRYIARNREGRWNMLAKCAGLKRTGKSCRLRWLNYLKPDVKRGNLTPQEQLLILELHSKLGNRWSKIAQHLPGRTDNEIKNYWRTKVQKEA
Populus trichocarpa v3.0	Potri.010G141000.1	Pt2R-MYB107	KVGLKKGRWTAEEDEVLTKYILANGEGSWKSLPKNAGLLRCGKSCRLRWINYLRADLKRGNITKEEEETIVKLHTALGNRWSFIAAQLPGRTDNEIKNYWNSHLSRKI
Populus trichocarpa v3.0	Potri.010G149900.1	Pt2R-MYB108	MVDLRRGPWTVEEDFKLIDYIATHGEGRWNSLARCAGLKRTGKSCRLRWLNYLRPDVRRGNITLEEQLMILELHSRWGNRWSKIAQHLPGRTDNEIKNYWRTRVQKHA
Populus trichocarpa v3.0	Potri.010G165700.1	Pt2R-MYB109	KVGLKKGPWTPEEDQKLLAYVEEHGHGSWRALPAKAGLQRCGKSCRLRWTNYLRPDIKRGKFSLQEEQTIIQLHALLGNRWSVIATHLPKRTDNEIKNYWNTHLKKRL
Populus trichocarpa v3.0	Potri.010G167500.1	Pt2R-MYB110	SKLCARGHWRPAEDTKLKELVALYGPQNWNLIAEKLEGRSGKSCRLRWFNQLDPRINRKAFTEEEEERLMQTHRLYGNKWAMIARLFPGRTDNAVKNHWHVIMARKF
Populus trichocarpa v3.0	Potri.010G174500.1	Pt2R-MYB111	KQRVQRGLWSPEEDEKLVKYIATHGHGSWSSVPKFAGLKRCGKSCRLRWLNYLRPELKRGCFSALEEQVIIDAHRILGNRWSQISKHLPGRTDNEVKNFWNSRIKKKL
Populus trichocarpa v3.0	Potri.010G195000.1	Pt2R-MYB112	TNGKVKGPWSPEEDAVLSQLVSKFGARNWSLIARGIPGRSGKSCRLRWCNQLDPCLKRKPFTDEEDRIIIAAHAKHGNKWAAIARLLPGRTDNSIKNHWNSTLRRRW
Populus trichocarpa v3.0	Potri.011G040200.1	Pt2R-MYB113	NLCLKRGAWDPDEDQILRAYIMRYGTRNWNEMPKAAGLLRSGKSCRFRWMNYLRPDIKRGNFSKEEVQTIIKLHEMLGNRWSAIAEKLPGRTDNDIKNFWNTHLRKSF
Populus trichocarpa v3.0	Potri.011G040300.1	Pt2R-MYB114	NLFLKRGTWDPDEDQKLRAYIKRHSIWNWNEMPRAAGLLRSGKSCRLRWMNYLRPDIKRGSFSKEEVQTIIKLQEMLGNRWSTIAAKLPGRTDNDIKNFWNTHVRNRF
Populus trichocarpa v3.0	Potri.011G040400.1	Pt2R-MYB115	NFCLKRGTWSADEDQMLRAYIKRYGIWNWNEMPKAAGLSRSGKSCRLRWMNYLRPGIKRGNLSKEEVQTIIKLHEMLGNRWSTIAAKLPGRTDNEIKNFWNTYLRNRF
Populus trichocarpa v3.0	Potri.011G041600.1	Pt2R-MYB116	KNGLKKGPWTPDEDQKLIDYIQKHGYGNWRTLPKNAGLQRCGKSCRLRWTNYLRPDIKRGRFSFEEEETIIQLHGILGNKWSAIAARLPGRTDNEIKNYWNTHIRKRL
Populus trichocarpa v3.0	Potri.011G125900.1	Pt2R-MYB117	KNGLKKGPWTAEEDQKLITYIQKHGHGRWRILPKNAGLKRCGKSCRLRWTNYLRPDIKRGKFSDEEEETIIQLHGILGNKWSTIATRLPGRTDNEIKNYWNTHIKKRL
Populus trichocarpa v3.0	Potri.011G167600.1	Pt2R-MYB118	KDNVKRGQWTPEEDNKLSSYIAQHGTRNWRLIPKNAGLQRCGKSCRLRWTNYLRPDLKHGQFSDAEEHTIVKLHSVVGNRWSLIAAQLPGRTDNDVKNHWNTKLKRKL
Populus trichocarpa v3.0	Potri.012G039400.1	Pt2R-MYB119	MCTRGHWRPAEDEKLKELVEKYGPHNWNAIAEKLQGRSGKSCRLRWFNQLDPRINRSPFTEEEEERLLASHRIHGNRWAIIARFFPGRTDNAVKNHWHVIMARRY
Populus trichocarpa v3.0	Potri.012G055600.1	Pt2R-MYB120	DAELRRGPWTLEEDTLLVHYIARHGEGRWNLLAKRAGLRRTGKSCRLRWLNYLKPDVKRGNLTPQEQLLILDLHSKWGNRWSKIAQHLPGRTDNEIKNYWRTRVQKQA
Populus trichocarpa v3.0	Potri.012G072500.1	Pt2R-MYB121	RLNVKRGLWTAEEDAKMLAHVAKHGTGNWTAVPKKAGLQRCGKSCRLRWTNYLRPDLKHDSFTPQEEEMIIRLHAAIGSRWSIIAQQLPGRTDNDVKNYWNARLRKKL
Populus trichocarpa v3.0	Potri.012G080400.1	Pt2R-MYB122	NGEYRKGLWTEEEDRILTDHVKVHGKGKWNQIAKVTGLKRCGKSCRLRWMNYLSPSVKRGVFSEEEDDLIIRLHKLLGNRWSLIAGRVPGRTDNQVKNHWNTHLSKRL
Populus trichocarpa v3.0	Potri.012G082000.1	Pt2R-MYB123	KNSVVKGQWTTEEDRLLIHLVEQHGVRKWSDVAKMLSGRIGKQCRERWHNHLRPDIKKDAWSVEEDKILIQAHSVLGNRWAEIAKSLPGRTENSIKNHWNATKRKQY
Populus trichocarpa v3.0	Potri.012G084100.1	Pt2R-MYB124	KQKLRKGLWSPEEDERLFNYITRFGVGCWSSVPKLAGLQRCGKSCRLRWINYLRPDLKRGMFSQQEEDLIISFHEVLGNRWAQIAAQLPGRTDNEIKNFWNSCLKKKL
Populus trichocarpa v3.0	Potri.012G140700.1	Pt2R-MYB125	KDEVKKGPWTPEEDQLLVQYIQKHGLGCWRSLPKNAGLLRCGKSCRLRWTNYLRPDIKRGPFSPAEEATIVHLHGMLGNKWAYMASQLPGRTDNEIKNFWNTHLKKRL
Populus trichocarpa v3.0	Potri.012G140500.1	Pt2R-MYB126	KLRHRKGLWSPEEDQRLGSYVFQHGHGCWSSVPINAGLQRTGKSCRLRWINYLRPGLKRGAFSTDEEETILTLHRMLGNKWSQIAQHLPGRTDNEIKNHWHSYLKKKL

Populus trichocarpa v3.0	Potri.012G127700.1	Pt2R-MYB127	KLGVKKGPWTAEEDKKLVNFILTHGQCCWRAVPKLAGLRRCGKSCRLRWTNYLRPDLKRGLLNEDEEQLVIDLHACLGNRWSKIAARLPGRTDNEIKNHWNTHIKKKL
Populus trichocarpa v3.0	Potri.013G001000.1	Pt2R-MYB128	KQKLRKGLWSPEEDEKLLNYITKHGLGCWSSVPKLAGLQRCGKSCRLRWINYLRPDLKRGAFSQQEENLIIELHAVLGNRWSQIAAQLPGRTDNEIKNLWNSCIKKKL
Populus trichocarpa v3.0	Potri.013G046300.1	Pt2R-MYB129	RIMIKGGVWKNTEDEILKAAVMKYGKNQWARISSLLVRKSAKQCKARWYEWLDPSIKKTEWTREEDEKLLHLAKLMPTQWRTIAPIVGRTPSQCLERYEKLLDAAC
Populus trichocarpa v3.0	Potri.013G056400.1	Pt2R-MYB130	KVGIKRGPWTPEEDELLANYIKNDGEGRWRTLPKQAGLLRCGKSCRLRWMNYLRPSVKRGRIAPDEEDLILRLHRLLGNRWSLIAGRIPGRTDNEIKNYWNTHLSKKL
Populus trichocarpa v3.0	Potri.013G056500.1	Pt2R-MYB131	KVGIKRGPWTPEEDEILANYIKKDGEGRWRTLPKRAGLLRCGKSCRLRWMNYLRPSVKRGRIAPDEEDLILRLHRLLGNRWSLIAGRIPGRTDNEIKNYWNTHLSKKL
Populus trichocarpa v3.0	Potri.013G067000.1	Pt2R-MYB132	AKLCARGHWRPAEDAKLKELVAQYGPQNWNLIAENLEGRSGKSCRLRWFNQLDPRINRRAFAEEEEERLLAAHRLYGNKWAMIARLFPGRTDNAVKNHWHVIMARKH
Populus trichocarpa v3.0	Potri.013G067500.1	Pt2R-MYB133	KVGIKKGPWTPEEDIILVSYIQEHGPGNWRSVPTNTGLLRCSKSCRLRWTNYLRPGIKRGNFTPHEEGMIIHLQALLGNKWAAIASYLPQRTDNDIKNYWNTHLKKKL
Populus trichocarpa v3.0	Potri.013G109300.1	Pt2R-MYB134	KEHTNKGAWTKEEDQRLMDYIRVHGEGCWRSLPKAAGLLRCGKSCRLRWINYLRPDLKRGNFTDEEDEIIIKLHSLLGNKWSLIAGRLPGRTDNEIKNYWNTHIKRKL
Populus trichocarpa v3.0	Potri.013G148600.1	Pt2R-MYB135	KNGLKKGPWTPEEDQKLTAYIQLQGPGNWRSLPKNAGLQRCGKSCRLRWTNYLRPDIKRGRFSFEEEESIIQLHSILGNKWSAIAARLPGRTDNEIKNYWNTHIRKRL
Populus trichocarpa v3.0	Potri.013G149100.1	Pt2R-MYB136	KNGIRKGSWSIEEDEKLRVYIQKYGHWNWRQLPKFAGLSRCGKSCRLRWMNYLRPDVKRGNFSEEEDNLIIQMHEELGNKWSIISGKLPGRTDNEIKNHWHTNLSKRV
Populus trichocarpa v3.0	Potri.013G149200.1	Pt2R-MYB137	KTGLKKGAWTPEEDRKLMAYVTRYGCWNWRQLPKFAGLQRCGKSCRLRWLNYLRPNIKRGNYSKEEEETIISLHETMGNRWSAIAAQLPGRTDNEIKNHWHTNLKKRL
Populus trichocarpa v3.0	Potri.014G022500.1	Pt2R-MYB138	DVDRIKGPWSPEEDEALQRLVQTYGPRNWSLISKSIPGRSGKSCRLRWCNQLSPEVEHRPFSAEEDDTIIRAHARIGNKWATIARLLNGRTDNAIKNHWNSTLKRKC
Populus trichocarpa v3.0	Potri.014G035100.1	Pt2R-MYB139	KSERIKGPWSAEEDRILTRLVEQHGPRNWSLISRYIKGRSGKSCRLRWCNQLSPNVQHRPFSPVEDETILAAHARFGNRWATIARLLPGRTDNAVKNHWNSTLKRRA
Populus trichocarpa v3.0	Potri.014G054700.1	Pt2R-MYB140	KEEIRKGPWKEEEDEVLINHVKKYGPRDWSSIRSKGLLHRTGKSCRLRWVNKLRPNLKNGCKFSAEEERVVIDLQAEIGNRWARIATYLPGRTDNDVKNFWSSRQKRLA
Populus trichocarpa v3.0	Potri.014G054700.1	Pt2R-MYB141	KEEIRKGPWKEEEDEVLINHVKKYGPRDWSSIRSKGLLHRTGKSCRLRWVNKLRPNLKNGCKFSAEEERVVIDLQAEIGNRWARIATYLPGRTDNDVKNFWSSRQKRLA
Populus trichocarpa v3.0	Potri.014G081200.1	Pt2R-MYB142	KNGLKKGAWTPEEDELLVSYIKKNGHGSWRSLPKLAGLLRCGKSCRLRWTNYLRPDIKRHPFTLEEEKLVIQLHGILGNRWAAIASQLPGRTDNEIKNLWNTHLKKRL
Populus trichocarpa v3.0	Potri.014G100800.1	Pt2R-MYB143	KVGLHRGPWTPREDTLLTKYIQAHGEGHWRSLPKKAGLLRCGKSCRLRWMNYLRPDIKRGNITPDEDDLIIRMHSLLGNRWSLIAGRLPGRTDNEIKNYWNTHLVKRL
Populus trichocarpa v3.0	Potri.014G111200.1	Pt2R-MYB144	KQKLRKGLWSPEEDEKLLRHITKYGHGCWSSVPKQAGLQRCGKSCRLRWINYLRPDLKRGTFSQQEENLIIELHAVLGNRWSQIAAQLPGRTDNEIKNLWNSCLKKKL
Populus trichocarpa v3.0	Potri.014G117000.1	Pt2R-MYB145	DMGTRKGPWTVEEDSLLAHYITNHGEGQWNTAARCAGLKRTGKSCRLRWLNYLRPNVRRGNITLQEQLLILQLHSHWGNRWSKIAQQLPGRTDNEIKNYWRTRVQKQA
Populus trichocarpa v3.0	Potri.014G122700.1	Pt2R-MYB146	KVGLKKGRWTAEEDEKLTKYIQANGEGSWRSLPKNAGLLRCGKSCRLRWINYLAADLKRGNISAEEEEIIINLHASLGNRWSLIASHLPGRTDNEIKNYWNSHLSRRI
Populus trichocarpa v3.0	Potri.015G046200.1	Pt2R-MYB147	GSELRRGPWTLEEDTLLVHYIGRHGEGRWNLLAKRAGLRRTGKSCRLRWLNYLKPDVKRGNLTPQEQLLILDLHSKWGNRWSKIAQHLPGRTDNEIKNYWRTRVQKQA
Populus trichocarpa v3.0	Potri.015G041100.1	Pt2R-MYB148	KQYTNKGAWSQQEDQKLIDYIQKHGEGCWRSLPQAAGLLRCGKSCRLRWRNYLRPDLKRDGFGEDEEDLIIRLHALLGNRWSLIAGRLPGRTDNEVKNYWNSHIRKKL
Populus trichocarpa v3.0	Potri.015G033600.1	Pt2R-MYB149	MCTRGHWRPAEDEKLKELVEKYGPHNWNAIAEKLHGRSGKSCRLRWFNQLDPRINRSPFTEEEEERLLASHRIHGNRWAVIARLFPGRTDNAVKNHWHVIMARRY
Populus trichocarpa v3.0	Potri.015G067700.1	Pt2R-MYB150	KFNVKRGLWTADEDAKILAHVAKHGTGNWTAVPKKAGLQRCGKSCRLRWTNYLSPDLKHDNFTPQEEEMIIRLHAAIGSRWSIIAQQLPGRTDNDVKNCWNARLRKKL
Populus trichocarpa v3.0	Potri.015G075600.2	Pt2R-MYB151	SREYRKGLWTVEEDRILMDYVKVHGKGKWNRAAKVAGLKRGGKSCRLRWMNYLSPSVKRGAFSEEEDDLIIRLHKLLGNRFLFISWSLIAGRIPGRTDNQVKNHWNTHLSIKL
Populus trichocarpa v3.0	Potri.015G075800.1	Pt2R-MYB152	SSEYRKAFWTAEEDRILMDYVKAHGKGKWNPKRFECCGLGLKRCGKSCRLRWINYLSPSVKHGGFSEEEDDLIIRLHKLLGNRWSLIAGRVPGRTDNQVKNHWNTRISKKLGIK KGKCKISDSSSKFSKKL
Populus trichocarpa v3.0	Potri.015G077700.1	Pt2R-MYB153	KNSIVKGQWTTEEDRILIQLVEQNGVRKWCHVAQMLPGRIGKQCRERWHNHLRPNIKKDTWSVEEDKVLIQAHSELGNRWAEIAKMLPGRTENSIKNHWNATKRKQY
Populus trichocarpa v3.0	Potri.015G082700.1	Pt2R-MYB154	KQKLRKGLWSPEEDEKLLNYITRFGVGCWSSVPKLAGLQRCGKSCRLRWINYLRPDLKRGMFSQQEEDLIISLHEVLGNRWAQIAAQLPGRTDNEIKNLWNSYLKKKL
Populus trichocarpa v3.0	Potri.015G143500.1	Pt2R-MYB155	QDGVKKGPWTPEEDHLLVQYIQKNGFGSWHSLPKKAGLFRCGKSCRLRWTNYLRPDIKRGRFSPAEEATIIHLHGILGNRWAHIASQLPGRTDNEIKNFWNSHLKKRL
Populus trichocarpa v3.0	Potri.015G129100.1	Pt2R-MYB156	KLGVKKGPWTAEEDKKLVSFILSHGQCCWRAVPKLAGLRRCGKSCRLRWTNYLRPDLKRGLLNDDEEKLVIDLHARLGNRWSKIAARLPGRTDNEIKNHWNTHIKKKL
Populus trichocarpa v3.0	Potri.017G130300.1	Pt2R-MYB157	KVGLKKGPWTSDEDKKLITFILANGQCCWRAVPKLAGLLRCGKSCRLRWTNYLRPDLKRGLLSEYEEKMVIDLHAQLGNRWSKIASHLPGRTDNEIKNHWNTHIKKKL
Populus trichocarpa v3.0	Potri.017G128900.1	Pt2R-MYB158	KQGNNKGAWSVQEDQKLIDYIKTHGEGCWRSLPMAAGLHRCGKSCRLRWINYLRPDIKRGNFGQDEEDLIIKLHALLGNRWSLIAGRLPGRTDNEVKNYWNSHLKKKL
Populus trichocarpa v3.0	Potri.017G017600.1	Pt2R-MYB159	MSTRGHWRPAEDEKLRKLVDQYGPHNWNSIAEKLQGKSGKSCRLRWFNQLDPRINRSPFTEEEEERLLACQQIHGNKWAVIAKQFPGRTDNAVKNHWHIIMARKC
Populus trichocarpa v3.0	Potri.017G071500.1	Pt2R-MYB160	DVEVRKGPWTLEEDLILTNHIANHGEGVWNSLAKAAGLKRTGKSCRLRWLNYLRPDLRRGNITPEEQLLIMELHAKWGNRWSKIAKHLPGRTDNEIKNYWRTRIQKHI
Populus trichocarpa v3.0	Potri.017G075000.1	Pt2R-MYB161	KSNVKRGLWTPEEDAKILAYVSNHGIGNWTSVPKKAGLNRCGKSCRLRWTNYLRPDLKHERFAPEEEELIIKLHKAIGSRWSLIAKKLPGRTDNDVKNYWNTKLRKML
Populus trichocarpa v3.0	Potri.017G082500.1	Pt2R-MYB162	KIGVKKGPWTPEEDIILVSYIQEHGPGNWRAVPTSTGLLRCSKSCRLRWTNYLRPGIKRGNFTDHEEKMIIHLQALLGNRWAAIASYLPQRTDNDIKNFWNTHLKKKL
Populus trichocarpa v3.0	Potri.017G085200.1	Pt2R-MYB163	PKICNRGHWRPAEDSKLKELVALYGPQNWNLIAEKLRGRSGKSCRLRWFNQLDPKINRTAFNEDEEERLMAAHRVYGNKWALIARFFPGRTDNAVKNHWHIVMARKY
Populus trichocarpa v3.0	Potri.017G086300.1	Pt2R-MYB164	KVGLKKGPWTPEEDQKLLAYIEEHGHGSWRALPAKAGLQRCGKSCRLRWTNYLRPDIKRGKFSLQEEQTIIQLHALLGNRWSAIATHLPKRTDNEIKNYWNTHLKKRL
Populus trichocarpa v3.0	Potri.017G099500.1	Pt2R-MYB165	EKGWRKGPWTPEEDKLLIEYVSLHGEGRWSSVSRCPGLNRSGKSCRLRWVNYLRPGLKRGQITPQEEGIIIELHALLGNKWSTIARYLPGRTDNEIKNYWRTHSKKKE
Populus trichocarpa v3.0	Potri.017G112300.1	Pt2R-MYB166	MKERQRWQPEEDAILRAYVKQYGPKEWNLISQRVEASGKTLNRDPKSCLERWKNYLKPGIKKGCLTPEEQALVISLQAKYGNKWKKIASEVPGRTAKRLSKWWEVFKEKQS
Populus trichocarpa v3.0	Potri.018G005300.1	Pt2R-MYB167	KDGLNKGAWTPLEDKMLMDYLKIHGEGKWSNIVKETGLKRCGKSCRLRWMNYLRPNIKRGNFSEDEEDLIIRLHKLLGNRWSLIAGRLPGRTDNEIKNYWHTTIAKKA
Populus trichocarpa v3.0	Potri.018G049600.1	Pt2R-MYB168	KEGLNRGAWTALEDKILMAYIKAHGEGNWRNLPERAGLKRCGKSCRLRWLNYLRPDIKRGNISHDEEELIIRLHNLLGNRWSLIAGRLPGRTDNEIKNYWNTTLGKKA
Populus trichocarpa v3.0	Potri.018G049200.1	Pt2R-MYB169	KEGLNRGAWTALEDKTLMAYIKAHGEGNWRNLPERAGLKRCGKSCRLRWLNYLRPDIRRGNISHDEEELIIRLHNLLGNRWSVIAGRLPGRTDNEIKNYWNTTLGKKA
Populus trichocarpa v3.0	Potri.018G049000.1	Pt2R-MYB170	KKEANKGAWTAEEDQKLARVIEIHGPKRWRSIAAKADLNRCGKSCRLRWMNHLRPNIKRGNISDQEEDLILRLHKLLGNRWSLIAGRLPGRTDNEIKNYWNSHLSKKI

Populus trichocarpa v3.0	Potri.018G095900.1	Pt2R-MYB171	KNNVKKGPWSPEEDAKLKAYIDQFGTGGNWIALPQKVGLKRCGKSCRLRWLNYLRPNIKHGGFSEEEDNIICSLYISIGSRWSIIAAQLPGRTDNDIKNYWNTRLKKKL
Populus trichocarpa v3.0	Potri.018G127700.1	Pt2R-MYB172	KPKAKKGLWKPEEDLILKTYVETHGEGNWSTVSKKSGLMRGGKSCRLRWKNYLRPNIKRGGMSQEEEDMIIRMHKLLGNRWSLIAGRLPGRTDNEVKNYWNTHLNKRL
Populus trichocarpa v3.0	Potri.006G066400.1	Pt2R-MYB173	PHEFKKGLWKPEEDLILKTYVETHGEGNWSTVSKRSGLMRCGKSCRLRWKNYLRPNIRRGGMSQDEEDMIIRMHKLLGNRWSLIAGRLPGRTDNDVKNYWNTHLNKRC
Populus trichocarpa v3.0	Potri.T011400.1	Pt2R-MYB174	KEHTNKGAWTKEEDERLINYIKSHGEGCWRSLPKAAGLLRCGKSCRLRWINYLRPDLKRGNFSDEEDELIINLHSLLGNKWSLIAARLPGRTDNEIKNYWNTHIKRKL
Populus trichocarpa v3.0	Potri.019G018200.1	Pt2R-MYB175	MMGLKKGPWTPEEDQILVSYIRSYGHGNWRALPKQAGLLRCGKSCRLRWINYLRPDIKRGNFSNEEEETIIKLHQILGNRYVFLTRLPAGRTDNEIKNYWHSHLKKRF
Populus trichocarpa v3.0	Potri.019G018400.1	Pt2R-MYB176	RIMIKGGVWKNTEDEILKAAVMKYGKNQWARISSLLVRKSAKQCKARWYEWLDPSIKKTEWTREEDEKLLHLAKLMPTQWRTIAPIVGRTPSQCLERYEKLLDAAC
Populus trichocarpa v3.0	Potri.019G036300.1	Pt2R-MYB177	KVGIKKGPWTPEEDELLANYIRKEGQGRWRTLPKRAGLLRCGKSCRLRWMNYLRPSVKRGRIAPDEEDLILRLHRLLGNRWSMIAGRIPGRTDNEIKNYWNTCLSKKL
Populus trichocarpa v3.0	Potri.019G036400.1	Pt2R-MYB178	KVGIKKGPWTPEEDELLANYIRKEGEGRWRTLPKRAGLLRCGKSCRLRWMNYLRPSVKRGRIAPDEEDLILRLHRLLGNRWSMIAGRIPGRTDNEIKNYWNTCLSKKL
Populus trichocarpa v3.0	Potri.019G040900.1	Pt2R-MYB179	TKLCARGHWRPAEDAKLKELVAQYGPQNWNLIAENLEGRSGKSCRLRWFNQLDPGINRKAFTEEEEERLLAAHRLYGNKWAMISRLFPGRTDNAVKNHWHVIMARKH
Populus trichocarpa v3.0	Potri.019G045900.1	Pt2R-MYB180	KDGIKKGPWTPEEDIILVSYIQEHGPGNWRSVPTNTGLLRCSKSCRLRWTNYLRPGIKRGNFTPHEEGMIIHLQAILGNKWAAIASYLPQRTDNDIKNYWNTHLKKKL
Populus trichocarpa v3.0	Potri.019G050900.1	Pt2R-MYB181	ESALKKGPWTPEEDQKLIDYVQKHGHGSWRALPMLAGLNRCGKSCRLRWTNYLRPDIKRGNFSEEEQQMIIDFHQVLGNKWSAIAQHLPGRTDNEIKNFWNTHLKKKL
Populus trichocarpa v3.0	Potri.019G081500.1	Pt2R-MYB182	KAHTNKGAWTKEEDQRLIDYIRVHGEGCWRSLPKAAGLLRCGKSCRLRWINYLRPDLKRGNFTEEEDEIIIKLHSLLGNKWSLIAGKLPGRTDNEIKNYWNTHIKRKL
Populus trichocarpa v3.0	Potri.019G118200.1	Pt2R-MYB183	KNGLKKGPWTPEEDHKLISFIQLHGPGNWRSLPKNAGLQRCGKSCRLRWTNYLRPDIKRGRFSFEEEETIIQLHSVLGNKWSAIAARLPGRTDNEIKNYWNTHIRKRL
Populus trichocarpa v3.0	Potri.019G118700.1	Pt2R-MYB184	KNGLRKGAWSKEEDDKLRVYVQKYGHWNWRQLPRFAGLSRCGKSCRLRWMNYLRPDVKRGNFSDEEDNLIIQMHEELGNKWSIIAGKLPGRTDNEIKNHWHTNLSKRV
Populus trichocarpa v3.0	Potri.019G118800.1	Pt2R-MYB185	KNGVKKGAWSEEEDDKLREHIEKYGLWNWREIPKFAGLSRCGKSCRLRWMNYLRPDVKHGNYTKEEEDLILKLHEKHGNKWSIIAAKLPGRTDNEVKNYWHAHLKKRT
Populus trichocarpa v3.0	Potri.019G118900.1	Pt2R-MYB186	KTELKKGAWTPEEDMKLMAYVTRYGSWNWRQLPKYAGLQRCGKSCRLRWLNYLRPNIKRGNYTKEEEEIIISLHETLGNRWSAIAAQLPGRTDNEIKNHWHTNLKKRL
Populus trichocarpa v3.0	Potri.006G085600.1	Pt3R-MYB1	PIRRAKGGWTPEEDETLRTAVATYKGKSWKKIAEFFPDRSEVQCLHRWQKVLDPELVKGPWTQEEDDKIVELVAKYGPTKWSVIAKALPGRIGKQCRERWHNHLNPDIKKDAWTLEEELALMNAHRIYGNKWAEIAKVLPGRTDNSIKNHWNSSLKKKL
Populus trichocarpa v3.0	Potri.006G241700.1	Pt3R-MYB2	ARRSTKGQWTAEEDEILRKAVQRFKGKNWKKIAECFKDRTDVQCLHRWQKVLNPELVKGPWSKEEDEIIIELVNKYGPKKWSTIAQHLPGRIGKQCRERWHNHLNPSINKEAWTQQEELALIRAHQIYGNRWAELTKFLPGRTDNAIKNHWNSSVKKKL
Populus trichocarpa v3.0	Potri.018G038000.1	Pt3R-MYB3	ARRSTKGQWKAEEDEILRKAVQRFKGKNWKKIAECFKDRTDVQCLHRWQKVLNPELVKGPWSKEEDEIIIELVNKYGPKKWSTIAQHLPGRIGKQCRERWHNHLNPAINKEAWTQQEEVALIRAHQIYGNRWAELTKFLPGRTDNAIKNHWNSSVKKKL
Populus trichocarpa v3.0	Potri.014G079300.1	Pt3R-MYB4	GPTRRSKKWTGEEDKILADAVKRYNSKNWKKIAECVPDRTDVQCLHRWQKVLDPKLVKGPWKKEEDDLIRELVEKHGNKKWSQVAKHLTGRIGKQCRERWHNHLNPDINRTPWTKEEEAILIKAHGAYGNKWAEIAKLLHGRTENSIKNHWNCSVRKRI
Medicago truncatula 4.0v1	AC229728_1.1	Mt2R-MYB01	KRLKKGTLWSKEEDEILKAYVEKHGTGNWKEVSKNTGLAHCGNSCRFRWYNTLRPDLRKGPFSKEEEEKFFELFSKFGEFKWSKMALEMPGRTDNDIKNFWNARKRKHQR
Medicago truncatula 4.0v1	Medtr1g021230.1	Mt2R-MYB02	EGGLKKGPWTAAEDEILVAHVQKYGEGNWNSVRKCTGLARCGKSCRLRWANHLRPDLRKGAITAEEERRIIELHHKMGNKWAQMAALLPGRTDNEIKNFWNTRCKKRGR
Medicago truncatula 4.0v1	Medtr1g043050.1	Mt2R-MYB03	KKGLKKGPWTLEEDEILTSYINKHGHSNWRALPKDAGLLRCGKSCRLRWINYLRTDIKRGNFSNEEEQIIIKMHELLGNRWSAIAAKLPGRTDNEIKNMWHTHLKKRL
Medicago truncatula 4.0v1	Medtr1g043080.1	Mt2R-MYB04	KKGLKKGPWTLEEDEILTSYINKHGHSNWRALPKHAGLLRCGKSCRLRWINYLKPDIKRGNFTNEEEETIIKMHESLGNRWSAIAAKLPGRTDNEIKNVWHTHLKKRL
Medicago truncatula 4.0v1	Medtr1g045610.1	Mt2R-MYB05	EVWRKGPWTAEEDRLLIDYVRLHGEGRWNSVARLTGLKRNGKSCRLRWVNYLRPDLKRGQITPQEESIILELHARWGNRWSTIARSLPGRTDNEIKNYWRTHFKKKAK
Medicago truncatula 4.0v1	Medtr1g062050.1	Mt2R-MYB06	KSGLKKGPWTQEEDLMLINYINTHGPGNWRNLPKNAGLQRCGKSCRLRWTNYLRPDIKRGRFSFEEEESIIQLHSVLGNNTSQTLRFRYISKSKDFVRPLWSAIAARLPGRTDNEIKNYWNTHIRKRL
Medicago truncatula 4.0v1	Medtr1g073170.1	Mt2R-MYB07	DRWRKGPWTSEEDKLLIEYVKLHGEGRWNSVSRLAGLRRNGKSCRLRWVNYLRPDLKKGQITQQEESIILELHARWGNRWSTIARSLPGRTDNEIKNYWRTHFKKKNK
Medicago truncatula 4.0v1	Medtr1g083630.1	Mt2R-MYB08	TIKLKKGAWSLHEDAILKEYVRKYGEGNWDDVKKKTELFRCGKSCRLRWLNHLHPDLKKGSITREEEQKIVELHAKIGPKWSLMVQELPGRTDNEIKNFWNIRTRKRTK
Medicago truncatula 4.0v1	Medtr1g085040.1	Mt2R-MYB09	KQKLRKGLWSPEEDEKLLNYITKHGHGCWSSVPKLAGLQRCGKSCRLRWINYLRPDLKRGAFSQQEENLIIELHAVLGNRWSQIAAQLPGRTDNEIKNLWNSCLKKRL
Medicago truncatula 4.0v1	Medtr1g085900.1	Mt2R-MYB10	KRLKKGTLWSKEEDEILKAYVEKHGTGNWKEVSKNTGLAHCGNSCRFRWYNTLRPDLRKGPFSKEEEEKFFELFSKFGEFKWSKMALEMPGRTDNDIKNFWNARKRKHQR
Medicago truncatula 4.0v1	Medtr1g086510.1	Mt2R-MYB11	ETGVRKGPWTLEEDTILVDYITIHGEGHWNTLASSAGLRRSGKSCRLRWLNYLRPDLRRGNITLQEQILILDLHSRWGNRWSKIAQHLPGRTDNEIKNYWRTRVIKQA
Medicago truncatula 4.0v1	Medtr1g086530.1	Mt2R-MYB12	DTMVIKKGPWTEEEDTILMNYVAVHGDGHWNSVARCSGLKRTGKSCRLRWLNYLRPNVRRGNITLQEQVLILDLHSRWGNRWSKIAEQLPGRTDNEIKNYWRTRVVKQA
Medicago truncatula 4.0v1	Medtr1g110460.1	Mt2R-MYB13	EENELRRGPWTLEEDSLLIHYIARHGEGRWNMLAKSAGLKRTGKSCRLRWLNYLKPDIKRGNLTPQEQLLILELHSKWGNRWSKIAQHLPGRTDNEIKNYWRTRVQKQA
Medicago truncatula 4.0v1	Medtr2g033170.1	Mt2R-MYB14	DELDLRRGPWTVDEDLALINYIANHGEGRWNTLARSAGLKRTGKSCRLRWLNYLRPDVRRGNITLEEQLLILELHSRWGNRWSKIAQYLPGRTDNEIKNYWRTRVQKHA
Medicago truncatula 4.0v1	Medtr2g034790.1	Mt2R-MYB15	KVGLKKGRWTAEEDKILTDYIKENGEGSWRFLPKKAGLLRCGKSCRLRWINYLRADVKRGNISPHEEEIIVKLHAVLGNRWSVIASHLPGRTDNEIKNYWNSHLRRKI
Medicago truncatula 4.0v1	Medtr2g064160.1	Mt2R-MYB16	KQKIKRGLWSPEEDDKLITYITTHGHKSWSSVPKFAGLQRCGKSCRLRWTNYLRPDLKRGSFTAEEEQIIIDIHRILGNRWAQIAKHLPGRTDNEVKNFWNSCIKKKL
Medicago truncatula 4.0v1	Medtr2g088170.1	Mt2R-MYB17	RTGMKKGPWTPHEDMILTEYVNKHGEGNWNFVQKNSGLLRCGKSCRLRWANHLRPNLKKGSFSEEEEKIIIELHAKLGNKWARMATQLPGRTDNEIKNFWNTRMKRRQR
Medicago truncatula 4.0v1	Medtr2g089420.1	Mt2R-MYB18	NNGLRKGSWTHEEDKKLIAYVTRYGCWNWRQLPRFAGIRKGLERCGKSCRLRWMNYLRPDVKRGNFNQQEEELIIKLHEKLGNRWTVIASNFPGRTDNEIKNHWHTTLKKRFV
Medicago truncatula 4.0v1	Medtr2g089450.1	Mt2R-MYB19	KNGLRKGTWTPEEDKKLIAYVTRYGCWNWRQLPKFAGLERCGKSCRLRWLNYLRPDIKRGSFSHEEEETIIKLHEKLGNRWTMISANLPGRTDNEIKNYWHTTIKKTL
Medicago truncatula 4.0v1	Medtr2g089620.1	Mt2R-MYB20	KSGLKKGPWTQEEDLMLINYINTHGPGNWRNLPKNAGLQRCGKSCRLRWTNYLRPDIKRGRFSFEEEESIIQLHSVLGNNTSQTLRFRYISKSKDFVRPLWSAIAARLPGRTDNEIKNYWNTHIRKRL
Medicago truncatula 4.0v1	Medtr2g096380.1	Mt2R-MYB21	EVRKGPWTEQEDFKLVSFVGLFGDRRWDFIAKVSGLKVAGDSLNRTGKSCRLRWVNYLHPGLKRGKMTPHEERLVLELQSKWGNRWSRIARKLPGRTDNEIKNYWRTHMRKKAQ
Medicago truncatula 4.0v1	Medtr2g097910.1	Mt2R-MYB22	KKKLRKGLWSPEEDEKLMNYMVRNGQGCWSDVARNAGLERCGKSCRLRWINYLRPDLKRGAFSTQEEELIIHFHSLLGNRWSQIAARLPGRTDNEIKNFWNSTIKKRL
Medicago truncatula 4.0v1	Medtr2g099740.1	Mt2R-MYB23	KQKVKRGLWSPEEDEKLINYITNYGHGCWSSVPKLAGLQRCGKSCRLRWINYLRPDLKRGSFSPQEAALIIELHTILGNRWAQIAKHLPGRTDNEVKNFWNSSIKKKL
Medicago truncatula 4.0v1	Medtr3g039990.1	Mt2R-MYB24	KIGVKKGPWTPEEDIILVSYIQEHGPGNWRSVPTTTGLMRCSKSCRLRWTNYLRPGIKRGNFNDHEEKMIIHLQALLGNRWAAIASYLPERTDNDIKNYWNTHLRKKL

Medicago truncatula 4.0v1	Medtr3g052430.1	Mt2R-MYB25	EANLKKGPWTTGEDTILIDYVTKHGEGNWNAVQKNTGLNRCGKSCRLRWANHLRPNLKKGAFSHEEEKLIVELHAQFGNKWARMAALLPGRTDNEIKNYWNTRIKRRQR
Medicago truncatula 4.0v1	Medtr3g052450.1	Mt2R-MYB26	EANLKKGPWTTGEDTILIDYVTKHGEGNWNAVQKNIGLNRCGKSCRLRWANHLRPNLKKGAFSHEEEKIIVELHAQFGNKWARMAALLPGRTDNEIKNYWNTRIKRRQR
Medicago truncatula 4.0v1	Medtr3g065440.1	Mt2R-MYB27	KVGLKKGPWTAEEDKKLINFILTNGQCCWRAVPKLAGLLRCGKSCRLRWTNYLRPDLKRGLLSEYEEKMVIDLHAQLGNRWSKIASHLPGRTDNEIKNHWNTHIKKKL
Medicago truncatula 4.0v1	Medtr3g077110.1	Mt2R-MYB28	KQKLRKGLWSPEEDEKLFNYITMFGVGCWSSVPKLAGLQRCGKSCRLRWINYLRPDLKRGMFSKQEEDLIINLHEALGNRWAQIAAQLPGRTDNEIKNFWNSSLKKKL
Medicago truncatula 4.0v1	Medtr3g077650.1	Mt2R-MYB29	KRVGMNRNNKAYPYASRKPEKVKKKINFIKGQWTVEEDRLLVQLVGEHGLRKWSHIAENLPGRIGKQCRERWHNHLKPDIKKDIWSEEEDNILIKAHEEIGNKWAEIAKRLPGRTENSIKNHWNATKRRQYS
Medicago truncatula 4.0v1	Medtr3g083540.1	Mt2R-MYB30	KVGLKRGPWTVEEDEVLSEYIKKEGEGRWRTLPKRAGLLRCGKSCRLRWMNYLRPSVKRGQIAPDEEDLILRLHRLLGNRWSLIAGRIPGRTDNEIKNYWNTHLSKKL
Medicago truncatula 4.0v1	Medtr3g101290.1	Mt2R-MYB31	NQNKLDRIKGPWSAEEDRILTRLVEQHGARNWSLISRYIKGRSGKSCRLRWCNQLSPTVEHRPFSSQEDETIIAAHAQYGNRWATIARLLPGRTDNAVKNHWNSTLKRR
Medicago truncatula 4.0v1	Medtr4g063100.1	Mt2R-MYB32	RKGPWTEQEDYKLAYFVGLFGDRRWDFIAKVSGLKVAGESLSRSGKSCRLRWVNYLHPDLKRGKMTPQEERLVMELHSKWGNRWSRIARKLPGRTDNEIKNYWRTHMRKKR
Medicago truncatula 4.0v1	Medtr4g073420.1	Mt2R-MYB33	KAHTNKGAWTKEEDDRLISYIRAHGEGCWRSLPKAAGLLRCGKSCRLRWINYLRPDLKRGNFTEEEDELIIKLHSLLGNKWSLIAGRLPGRTDNEIKNYWNTHIRRKL
Medicago truncatula 4.0v1	Medtr4g082040.1	Mt2R-MYB34	EKINLNEDNPNENSYVSGKEVDTGHSKLCARGHWRPAEDSKLKELVALYGPQNWNLIAEKLEGRSGKSCRLRWFNQLDPRINRRAFSDEEEERLMQAHRIYGNKWAMIARLFPGRTDNAVKNHWHVIMARKY
Medicago truncatula 4.0v1	Medtr4g082230.1	Mt2R-MYB35	KVRLKKGPWTPEEDQQLIAYIKEFGHGSWHALPAKAGLPRCGKSCRLRWTNYLRPDIKRGKFSLQEEQTIIQLHALLGNRWSAIAAQLPRRTDNEIKNYWNTHLKKRL
Medicago truncatula 4.0v1	Medtr4g091490.1	Mt2R-MYB36	KNNGLKRGPWTQEEDQKLIDYIQKHGYGNWRLLPKNAGLQRCGKSCRLRWTNYLRPDIKRGRFSFEEEETIIQLHSILGNKWSSIASRLPGRTDNEIKNYWNTHIRKRL
Medicago truncatula 4.0v1	Medtr4g100720.1	Mt2R-MYB37	KETMNRGAWTQEEDQKLAQCIQIHGAKKWKTVANNSGLNRCGKSCRLRWLNYLRPNIKRGNISDEEEDLIIRLHKLLGNRWSLIAGRLPGRTDNEIKNYWNSHLCKKA
Medicago truncatula 4.0v1	Medtr4g108430.1	Mt2R-MYB38	KLGIKKGPWTPEEDIILVSYIQQHGPGNWRSVPTNTGLMRCSKSCRLRWTNYLRPGIKRGNFTDHEEKMIVHLQALLGNRWAAIASYLPQRTDNDIKNYWNTHLKRKM
Medicago truncatula 4.0v1	Medtr4g121460.1	Mt2R-MYB39	KIGLKKGRWTAEEDKILTDYIQEHGEGSWRSLPKNAGLLRCGKSCRLRWINYLRSDVKRGNITPQEEEIIVKLHAVLGNRWSVIAGHLPGRTDNEIKNYWNSHLRRKI
Medicago truncatula 4.0v1	Medtr4g128190.1	Mt2R-MYB40	KGLKKGKWSKEEDEMLKVSRNAGLIRCGKSCRLRWYNHLRPDVKKGPFSEEEKTKVFRFFNNFGEFKWSKLAQELPGRSDNDIKNFWNARKRKLE
Medicago truncatula 4.0v1	Medtr4g128670.1	Mt2R-MYB41	KANVKKGPWSPEEDATLKSYIETNGTGGNWIALPQKIGLKRCGKSCRLRWLNYLRPNIKHGGFTEEEDNIICSLYISIGSRWSIIAAQLPGRTDNDIKNYWNTRLKKKL
Medicago truncatula 4.0v1	Medtr5g007370.1	Mt2R-MYB42	ESGLKKGPWSPEEDKILVDHIQKHGHGSWRALPKLAGLNRCGKSCRLRWNNYLRPDIRRGKFSDEEENLIINLHSVLGNKWAAIATHLPGRTDNEIKNFWNTHLKKKL
Medicago truncatula 4.0v1	Medtr5g010020.1	Mt2R-MYB43	QQKVKRGLWSPEEDEKLIRYITTHGYGCWSEVPEKAGLQRCGKSCRLRWINYLRPDIRRGRFTPEEEKLIISLHGVVGNRWAHIASHLPGRTDNEIKNYWNSWIKKKI
Medicago truncatula 4.0v1	Medtr5g010470.1	Mt2R-MYB44	SIMTKKGVWKNTEDEILKAAVMKYGKNQWARISSLLVCKSAKQCKARWYEWLDPSIKKTEWTREEDEKLLHLAKVMPTQRRTISVGRTPSQCLERYEKLLDAACVKDGN
Medicago truncatula 4.0v1	Medtr5g014990.1	Mt2R-MYB45	KANVKKGPWSPEEDAKLKSYIEQNGTGGNWIALPQKIGLKRCGKSCRLRWLNYLRPNIKHGGFSEEEDDIICSLYVSIGSRWSIIAAQLPGRTDNDIKNYWNTRLKKKL
Medicago truncatula 4.0v1	Medtr5g016510.1	Mt2R-MYB46	GPWSPEEDEALQKLVEKHGPRNWSIISKSIPGRSGKSCRLRWCNQLSPQVEHRAFTPEEDDTIIRAHARFGNKWATIARLLSGRTDNAIKNHWNSTLKRKC
Medicago truncatula 4.0v1	Medtr5g029840.1	Mt2R-MYB47	EIGVKKGPWTQEEDEKLIDYINKHGHGNWGTLSKRAGLNRCGKSCRLRWTNYLRPDIKRGKFTDEEERVIINLHSVLGNKWSKIAAHLPGRTDNEIKNYWNTNIRKKL
Medicago truncatula 4.0v1	Medtr5g038910.1	Mt2R-MYB48	ESKKKERHIVTWTQEEDDILRDQIGIHGTENWAIIASKFKDKTTRQCRRRWYTYLNSDFKKGGWSPEEDMLLCEAQKIFGNRWTEIAKVVSGRTDNAVKNRFSTLCKKRA
Medicago truncatula 4.0v1	Medtr5g041570.1	Mt2R-MYB49	QSSKCARGHWRPAEDSKLKELVALYGPQNWNLIAEKLEGRSGKSCRLRWFNQLDPRINRRAFTEEEEERLMQAHRIYGNKWAMIARLFPGRTDNAVKNHWHVIMARKY
Medicago truncatula 4.0v1	Medtr5g042030.1	Mt2R-MYB50	LGLKKGPWTPKEDKKLIAYVTQHGHRNWHSLPSRAVLRRCGKSCRLRWINYMIPDIKRGNFSSEEDHNIIQLHALLGNKWSTIAALLPRRTDNEIKNHWNINIKKRL
Medicago truncatula 4.0v1	Medtr5g062790.1	Mt2R-MYB51	DTKKGTWSKEEDEILKAYVENHGTRNWNEVSKNAGLIRCGKSCRLRWYNHLQPDVKKGPFSEEEETKVFVFYKKYGEFKWSKLAEELPGRSDNDIKNFWNARKRKLER
Medicago truncatula 4.0v1	Medtr5g070020.1	Mt2R-MYB52	TSEILIKGQWTNEEDRKLIKLVKQYGERKWAQIAEKLEGRVGKQCRERWHNHLRPDIKKDSWSEEEEKILVATHAKIGNRWAEIAKKIPGRTENAIKNHWNATKRRQNS
Medicago truncatula 4.0v1	Medtr5g078140.1	Mt2R-MYB53	KVGLKKGPWTAEEDRKLINFILTNGQCCWRAVPKLAGLLRCGKSCRLRWTNYLRPDLKRGLLSEYEEKMVIDLHAQLGNRWSKIASHLPGRTDNEIKNHWNTHIKKKL
Medicago truncatula 4.0v1	Medtr5g078800.1	Mt2R-MYB54	RGLRKGAWTYEEDNLLKDCIQKYGEGKWHLVPKRAGLNRCRKSCRLRWVNYLNPAINRESFSHDEIDMILRLHKLLGNRWSLIAARLPGRTANDVKNYWHTNLRKKVI
Medicago truncatula 4.0v1	Medtr5g078860.1	Mt2R-MYB55	KARGLRKGAWTYEEDNLLKACIHKYGEGKWHLVPKRTGLNRCRKSCRLRWLNYLNPAINRESFAEDEIDMILRLHKLLGNKWSLIAARLPGRTANDVKNYWHTNLRKKVI
Medicago truncatula 4.0v1	Medtr5g078910.1	Mt2R-MYB56	RSLRKGAWTYEEDKLLKACIQKYGEGKWHLVSQRAGLNRCRKSCRLRWVNYLCPNIKRESFAEDEVDMILRLHKLLGNRWSLIAARLPGRTANDVKNFWHTHLRKKVV
Medicago truncatula 4.0v1	Medtr5g078930.1	Mt2R-MYB57	NSRIKRKRTWTSEEDKLLKAYINKYGEGKWHLIPKRAELFIPFAVCAGVLFVIGTSSLFLTGLNRCRKSCRLRWLNYLNPTINREIFSEDEADMILRLHNLLGNRWTLIAARLQGRTANDVKNYWNTHLKNKDV
Medicago truncatula 4.0v1	Medtr5g078950.1	Mt2R-MYB58	RAVKRGAWTYEEDKLLKACINKYGEGKWHFVPQRAGLSRCRKSCRLRWLNYLSPNIKRESFAEDEVDLILRLQKLLGNRWSLIAARLPGRTANDVKNYWHTNLRKKL
Medicago truncatula 4.0v1	Medtr5g079120.1	Mt2R-MYB59	NKRGLRKGAWTYEEDNLLKAYIHKYGEGKWHLIPKRTGLNRCRKSCRLRWVNYLNPNINRESITEDEADMIIRLHNLLGNRWSLIAARLPSRTANDVKNYWNTHLRKKVL
Medicago truncatula 4.0v1	Medtr5g079220.1	Mt2R-MYB60	TRGVRKGAWTYEEDKLLKACMQKYGEGKWHLVPQRAGLNRCRKSCRLRWLNYLNPTINRESFSEDEVDMILRLHKLLGNRWSLIAARLPGRTANDVKNYWHTHLRKKMV
Medicago truncatula 4.0v1	Medtr5g079290.1	Mt2R-MYB61	TRGVRKGAWTYEEDKLLKACIQKYGEGKWHLVPQRAGLNRCRKSCRLRWLNYLTPNIKRESFAEDEVDMMLRLHKLLGNRWSLIAARLPGRTANDVKNYWHTHLRKKM
Medicago truncatula 4.0v1	Medtr5g079670.1	Mt2R-MYB62	KENINKGAWSKQEDQKLIDYIQVHGEGCWGSIPKAAGLHRCGKSCRLRWLNYLRPDIKRGIFAQDEEDLIIKLHALLGNRWALIAGRLPGRTDNEVKNYWNSHIRRKL
Medicago truncatula 4.0v1	Medtr5g082910.1	Mt2R-MYB63	ENGDKVKGSWSKTEDETLVKLVKENGARNWSVISNEIPGRSGKSCRLRWCNQLSPTVQHLPFTPAEDSIIIEAHAVHGNKWATISRLLPGRTDNAIKNHWNSTLRRRRT
Medicago truncatula 4.0v1	Medtr5g088080.1	Mt2R-MYB64	STDTKKGTWSKEEDEILKAYVEKHGTRNWNEVSKNAGLIRCGKSCRLRWYNHLQPDVKKGPFSEEEKSKVFEFYIKYGEFKWSKLAHELPGRSDNDIKNFWNARKRKLEK
Medicago truncatula 4.0v1	Medtr5g088150.1	Mt2R-MYB65	STDTKKGTWSKEEDEILKAYVEKHGTRNWNEVSKNAGLIRCGKSCRLRWYNHLQPDVKKGPFSEEEKSKVFEFYIKYGEFKWSKLAHELPGRSDNDIKNFWNARKRKLEK
Medicago truncatula 4.0v1	Medtr5g088610.1	Mt2R-MYB66	DTKKGKWSKEEDEILKAYVEKHGTRNWDEVSKNTGLAHFGRSCRFRWYNNLRPGVKKGPFSKEEENKVFQLRKKFGGFKWSKIARELPGRTDNDIKNFWNARKRKHKK
Medicago truncatula 4.0v1	Medtr5g088640.1	Mt2R-MYB67	STDTKKGTWSKEEDEILKAYVEKHGTRNWNEVSKNAGLIRCGKSCRLRWYNHLQPDVKKGPFSEEEKSKVFEFYIKYGEFKWSKLAHELPGRSDNDIKNFWNARKRKLEK
Medicago truncatula 4.0v1	Medtr6g009430.1	Mt2R-MYB68	KTNVKRGLWTPEEDAKILAYVANHGIGNWTAVPKKAGLNRCGKSCRLRYTNYLRPDLKHDSFTPEEEELIITLHGAIGSRWSCIAKRLPGRTDNDVKNYWNTKLRKKL

Medicago truncatula 4.0v1	Medtr6g012180.1	Mt2R-MYB69	KEGVKKGPWTPEEDIILVSYIQEHGPGNWKAVPTNTGLLRCSKSCRLRWTNYLRPGIKRGNFTDQEEKMIIHLQALLGNRWAAIAAYLPQRTDNDIKNYWNTYLKKKL
Medicago truncatula 4.0v1	Medtr6g012690.1	Mt2R-MYB70	KVGLKKGPWTPEEDQKLLAYIDEHGHGSWRALPAKAGLQRCGKSCRLRWTNYLRPDIKRGKFSLQEEQTIIQLHALLGNRWSAIATHLAKRTDNEIKNYWNTHLKKRL
Medicago truncatula 4.0v1	Medtr6g031120.1	Mt2R-MYB71	NKMGLKKGPWTSEEDEVLVNFIKKNGGHGSWRSLPKLAGLLRCGKSCRLRWTNYLRPDIKRGPFTPEEEKLVIQLHAILGNRWASIASQLPGRTDNEIKNLWNTHLKKRL
Medicago truncatula 4.0v1	Medtr6g055910.1	Mt2R-MYB72	KSKYRKGLWSPEEDNKLSNYIMKHGHSCWSSVPIKAGLQRNGKSCRLRWINYLRPGLKRGMLSKHEEDKIMTLHQMLGNKWSQIAQQLPGRTDNEIKNYWHSYLKKKEV
Medicago truncatula 4.0v1	Medtr6g074860.1	Mt2R-MYB73	SRGHWRPSEDEKLRELVESYGPHNWNAIAENLRGRSGKSCRLRWFNQLDPRINRNPFTEEEEERLITSHRIHGNRWAVIARHFPGRTDNAVKNHWHVMMARIR
Medicago truncatula 4.0v1	Medtr7g010210.1	Mt2R-MYB74	KEGVKKGPWTPEEDIILVTYIQEHGPGNWRAVPTKTGLSRCSKSCRLRWTNYLRPGIKRGNFTEQEEKMIIHLQDLLGNRWAAIASYLPQRTDNDIKNYWNTHLKKKL
Medicago truncatula 4.0v1	Medtr7g011170.1	Mt2R-MYB75	KVGLKKGPWTSEEDQKLLSYIEEHGHGSWRSLPTKAGLERCGKSCRLRWTNYLRPDIKRGKFSVQEEQTIIQLHAFLGNRWSSIATRLPKRTDNEIKNYWNTHLKKRL
Medicago truncatula 4.0v1	Medtr7g017260.1	Mt2R-MYB76	NTIGVKKGAWTYEEDNLLKAYINKYGEGKWHLIPQRAGSGLNRCRKSCRLRWINYLKPNINRKSFSEDEVDMILRLHKLLGNRWSLIAGRLPGRTANSVKNYWNTHLLKKVV
Medicago truncatula 4.0v1	Medtr7g037130.1	Mt2R-MYB77	KDLNIIKGQWTTDEDRILIQLVDRFGLRNWSKIAKYMNGRIGKQCRERWNNHLCLDIKKEAWTEEEDKILVEAHKIVGNKWAEISRRLSGRTENSVKNHWNATKRCLKA
Medicago truncatula 4.0v1	Medtr7g037260.1	Mt2R-MYB78	KDLNIIKGQWTTDEDRILIQLVDRFGLRKWSKIAKYMNGRIGKQCRERWNNHLCPDIKKESWTEEEDKILIEAHKIVGNKWAEIARRLPGRTENSVKNHWNTTKRRQKA
Medicago truncatula 4.0v1	Medtr7g061550.1	Mt2R-MYB79	MSDMKDRQRWRAEEDALLRAYVKQYGPREWNLVSQRMNTPLNRDAKSCLERWKNYLKPGIKKGSLTEEEQRLVISLQATHGNKWKKIAAQVPGRTAKRLGKWWEVFKEKQQ<br>R
Medicago truncatula 4.0v1	Medtr7g076740.1	Mt2R-MYB80	KKGLKKGPWTAEEDEILANYIKKNGGHGSWRSLPKITGLLRCGKSCRLRWTNYLRPDIKRGPFTPEEEKLVIQLHAILGNRWAAIASQLPGRTDNEIKNFWNTHLKKRL
Medicago truncatula 4.0v1	Medtr7g086960.1	Mt2R-MYB81	VKVGYSKLCSRGHWRPIEDAKLKELVAEYGPQNWNLIAEHLEGRSGKSCRLRWFNQLDPRINKKTFSEEEEERLLSAHKMYGNKWAIIARLFPGRTDNAVKNHWHVIMARRH
Medicago truncatula 4.0v1	Medtr7g087130.1	Mt2R-MYB82	KVGIKKGPWTPEEDIILVSYIQEHGPGNWRTVPTNTGLSRCSKSCRLRWTNYLRPGIKRGNFTPHEEGMIIHLQALLGNKWAAIASYLPQRTDNDIKNYWNTHLKKKL
Medicago truncatula 4.0v1	Medtr7g087350.1	Mt2R-MYB83	KVGIKKGPWTPEEDIILVSYIQEHGPGNWRTVPTNTGLSRCSKSCRLRWTNYLRPGIKRGNFTPHEEGMIIHLQALLGNKWAAIASYLPQRTDNDIKNYWNTHLKKKL
Medicago truncatula 4.0v1	Medtr7g102110.1	Mt2R-MYB84	KANVKKGPWSPEEDAKLKEYIEKHGTGGNWITLPKKVGLTRCGKSCRLRWLNYLRPNIKHGEFSDSEDKIICTLFASIGSRWSIIASKLEGRTDNDVKNYWNTKLKKKI
Medicago truncatula 4.0v1	Medtr7g109320.1	Mt2R-MYB85	KVGLKKGRWTAEEDEILTQYVKANGEGSWRSLPKNAGLLRCGKSCRLRWINYLRADLKRGNISSEEESIIIKMHASFGNRWSLIASHLPGRTDNEIKNYWNSHLSRKV
Medicago truncatula 4.0v1	Medtr7g110830.1	Mt2R-MYB86	KQKLRKGLWSPEEDEKLLNHITKYGHGCWSSVPKQAGLQRCGKSCRLRWINYLRPDLKRGTFSQEEENLIIELHSVLGNRWSQIAAQLPGRTDNEIKNLWNSCLKKKL
Medicago truncatula 4.0v1	Medtr7g111290.1	Mt2R-MYB87	KQKLRKGLWSPEEDEKLLNYITKHGHGCWSSVPKLAGLQRCGKSCRLRWINYLRPDLKRGAFSEQEENSIIELHALLGNRWSQIAAQLPGRTDNEIKNLWNSSLKKKL
Medicago truncatula 4.0v1	Medtr8g006470.1	Mt2R-MYB88	EHVRKGPWKTEEDEVLLHHVKKYGPRDWSSIRSKGLLQRTGKSCRLRWVNKLRPNLKNGCKFTIEEERIVIELQEQFGNKWAKIASYLPGRTDNDVKNFWSSRQKRLA
Medicago truncatula 4.0v1	Medtr8g020490.1	Mt2R-MYB89	KEVNKGAWSREEDDILSKYVVIHGEGKWQKVAQNAGLKRCGKSCRQRWLNYLKPGIKRGHISTDEEDMIIRLHRLLGNRWSLIAKRLPGRTDNEIKNYWNTNLSKKL
Medicago truncatula 4.0v1	Medtr8g060940.1	Mt2R-MYB90	GGVRKGAWTYKEDELLKACINTYGEGKWNLVPQRSGLNRCRKSCRLRWLNYLSPNINRGRFSEDEEDLILRLHKLLGNRWSLIAGRLPGRTANDVKNYWHTNLAKKVV
Medicago truncatula 4.0v1	Medtr8g086920.1	Mt2R-MYB91	KLGIKKGPWTPEEDIILVSYIQQHGPGNWRSVPTNTGLMRCSKSCRLRWTNYLRPGIKRGNFTDHEEKMIVHLQALLGNRWAAIASYLPQRTDNDIKNYWNTHLKRKM
Medicago truncatula 4.0v1	Medtr8g095390.1	Mt2R-MYB92	KQHTNKGAWSKEEDERLINYIKQQGEGCWRSLPKAAGLARCGKSCRLRWINYLRPDLKRGNFTHEEDELIISLHAMVGNKWSQIAQKLPGRTDNEIKNYWNTHIKRKL
Medicago truncatula 4.0v1	Medtr7g061330.1	Mt3R-MYB1	PIRRAKGGWTAEEDETLRNAVAAFKGKHWKKIAEFFADRSEVQCLHRWQKVLNPELVKGPWTQEEDDKIVELVAKYGPTKWSLIAKSLPGRIGKQCRERWHNHLNPDIKKDAW<br>TLEEELALMNAHRIHGNKWAEIAKVLPGRTDNAIKNHWNSSLKKKL
Medicago truncatula 4.0v1	Medtr7g461410.1	Mt3R-MYB2	PIRRAKGGWTAEEDETLRNAVAAFKGKHWKKIAEFFADRSEVQCLHRWQKVLNPELVKGPWTQEEDDKIVELVAKYGPTKWSLIAKSLPGRIGKQCRERWHNHLNPDIKKDAW<br>TLEEELALMNAHRIHGNKWAEIAKVLPGRTDNAIKNHWNSSLKKKL
Medicago truncatula 4.0v1	Medtr1g026870.1	Mt3R-MYB3	TRRSTKGQWTPEEDETLRKAVQRFQGKNWKKIAECFKDRTDVQCLHRWQKVLNPELVKGPWSKEEDDVMIELVNKYGPKKWSTIAQHLPGRIGKQCRERWHNHLNPAINKEA<br>WTQEEELALIRAHQIYGNKWAELTKFLPGRTDNAIKNHWNSSVKKKL
Medicago truncatula 4.0v1	Medtr3g110028.1	Mt3R-MYB4	TRRSTKGQWTPEEDNILRKAVERFQGKNWKKIAECFKDRTDVQCLHRWQKVLNPELIKGPWSKEEDETIVDLVNKYGPKKWSTIAQHLPGRIGKQCRERWHNHLNPSINKEAWT<br>QEEELALIHAHQIYGNRWAELSKFLPGRTDNAIKNHWNSSVKKKL
Medicago truncatula 4.0v1	Medtr5g010650.1	Mt3R-MYB5	QISSKNGIWTEEEDNLLIEIVKMHDGSIWKSIGGCAAYFPGRTDIECFHRWQKVSNHDLVKRSWTKEEDEALIELVRKYGFKRWSFIAKSMPGRIGKQCRERWHNNLDPVIKKGA<br>WTEEEESILAHYYQIYGSKWSEIARVLPGRSDNAIKNHWNSSMKKKS

Supplementary Table S2. Summary of information of 73 eukaryote 2R-MYB subfamilies.

Subfamily	Unikont										Bikont																																									
																						Plantae																															
														Chromalveolata									Viridiplantae																														
	Ichthyosporea	Fung					Apusozoa		Amoebozoa		Excayata			Alveolata		Stramenopiles			Rhizaria	Haptophyta	Cryptophyta	Rhodophyta	Chlorophyta				Moss	Tracheophyte	Gymnosperm	basal magnoliophyta	Eudicots								Monocots		Intron patterns												
	Sa	Sc	Um	Nh	Am	Sp	Fa	Ttr	Dp	Dd	Tb	Lm	Ng	Th	Pf	Pr	Ps	Ak	Bn	Eh	Gt	Cm	Ol	Mp	Vc	Cr	Pp	Sm	Pa	Atr	Ac	Sl	St	Vv	At	Cs	Pt	Mt	Zm	Os	a	b	c	d	e	f	g	h	i	j	k	l	other
**S1**	.	.	.	.	.	.	.	.	.	.	.	.	.	.	.	.	.	.	.	.	.	.	.	.	.	.	.	.	1	1	3	5	5	3	5	3	4	7	12	7	46	2	2	4	.	.	.	.	.	.	.	.	.
**S2**	.	.	.	.	.	.	.	.	.	.	.	.	.	.	.	.	.	.	.	.	.	.	.	.	.	.	.	.	.	.	2	5	5	4	3	2	4	2	5	3	30	.	4	.	.	.	.	.	.	.	.	.	.
**S3**	.	.	.	.	.	.	.	.	.	.	.	.	.	.	.	.	.	.	.	.	.	.	.	.	.	.	.	.	.	.	.	1	2	1	4	1	2	.	.	.	11	.	.	.	.	.	.	.	.	.	.	.	.
**S4**	.	.	.	.	.	.	.	.	.	.	.	.	.	.	.	.	.	.	.	.	.	.	.	.	.	.	.	.	17	3	3	4	4	9	6	4	12	3	11	5	30	.	31	.	.	.	.	.	.	.	.	.	.
**S5**	.	.	.	.	.	.	.	.	.	.	.	.	.	.	.	.	.	.	.	.	.	.	.	.	.	.	.	.	.	3	1	1	.	7	1	1	3	.	2	2	19	.	.	.	.	.	.	.	.	.	.	.	.
**S6**	.	.	.	.	.	.	.	.	.	.	.	.	.	.	.	.	.	.	.	.	.	.	.	.	.	.	.	.	.	.	3	4	.	10	4	.	5	10	.	.	36												.
**S7**	.	.	.	.	.	.	.	.	.	.	.	.	.	.	.	.	.	.	.	.	.	.	.	.	.	.	.	.	.	.	1	3	3	2	3	3	3	3	5	2	27	.	1	.	.	.	.	.	.	.	.	.	.
**S8**	.	.	.	.	.	.	.	.	.	.	.	.	.	.	.	.	.	.	.	.	.	.	.	.	.	.	3	.	3	2	3	5	4	4	6	3	7	2	8	5	27	4	19	.	.	.	.	.	.	.	.	.	.
**S9**	.	.	.	.	.	.	.	.	.	.	.	.	.	.	.	.	.	.	.	.	.	.	.	.	.	.	.	.	2	1	1	5	5	3	2	3	4	3	4	3	32	.	1	.	.	.	.	.	.	.	.	.	.
**S10**	.	.	.	.	.	.	.	.	.	.	.	.	.	.	.	.	.	.	.	.	.	.	.	.	.	.	.	.	.	.	.	.	.	.	2	.	.	.	.	.	.	.	2	.	.	.	.	.	.	.	.	.	.
**S11**	.	.	.	.	.	.	.	.	.	.	.	.	.	.	.	.	.	.	.	.	.	.	.	.	.	.	.	.	.	1	1	4	4	3	3	3	6	3	2	1	30	.	.	.	.	.	.	.	.	.	.	.	.
**S12**	.	.	.	.	.	.	.	.	.	.	.	.	.	.	.	.	.	.	.	.	.	.	.	.	.	.	.	.	.	.	.	.	.	.	6	.	.	.	.	.	6	.	.	.	.	.	.	.	.	.	.	.	.
**S13**	.	.	.	.	.	.	.	.	.	.	.	.	.	.	.	.	.	.	.	.	.	.	.	.	.	.	.	.	.	1	1	.	.	2	4	2	4	3	3	2	21	.	.	.	.	.	.	.	.	.	.	.	.
**S14**	.	.	.	.	.	.	.	.	.	.	.	.	.	.	.	.	.	.	.	.	.	.	.	.	.	.	.	.	8	3	5	9	6	7	6	6	8	3	11	8	68	1	.	.	.	.	.	.	.	.	.	.	.
**S15**	.	.	.	.	.	.	.	.	.	.	.	.	.	.	.	.	.	.	.	.	.	.	.	.	.	.	.	.	.	.	2	.	.	3	3	1	3	.	.	.	12	.	.	.	.	.	.	.	.	.	.	.	.
**S16**	.	.	.	.	.	.	.	.	.	.	.	.	.	.	.	.	.	.	.	.	.	.	.	.	.	.	.	.	.	1	1	1	1	1	3	1	1	1	3	3	17	.	.	.	.	.	.	.	.	.	.	.	.
**S17**	.	.	.	.	.	.	.	.	.	.	.	.	.	.	.	.	.	.	.	.	.	.	.	.	.	.	.	.	.	1	2	3	3	2	3	2	5	2	4	3	29	.	.	.	.	.	.	.	.	.	.	.	.
**S18**	.	.	.	.	.	.	.	.	.	.	.	.	.	.	.	.	.	.	.	.	.	.	.	.	.	.	2	3	3	2	2	4	5	4	7	3	5	13	11	5	.	.	.	1	.	#	.	.	.	.	.	.	.
**S19**	.	.	.	.	.	.	.	.	.	.	.	.	.	.	.	.	.	.	.	.	.	.	.	.	.	.	.	.	.	.	1	1	1	1	3	1	1	.	1	2	11	2	.	.	.	.	.	.	.	.	.	.	.
**S20**	.	.	.	.	.	.	.	.	.	.	.	.	.	.	.	.	.	.	.	.	.	.	.	.	.	.	.	.	.	2	3	7	7	4	6	5	8	4	13	7	50	6	1	7	.	.	.	.	.	.	.	.	.
**S21**	.	.	.	.	.	.	.	.	.	.	.	.	.	.	.	.	.	.	.	.	.	.	.	.	.	.	8	1	3	4	4	3	4	6	8	4	13	4	8	4	.	.	.	.	61	.	.	.	.	.	.	.	.
**S22**	.	.	.	.	.	.	.	.	.	.	.	.	.	.	.	.	.	.	.	.	.	.	.	.	.	.	1	2	3	2	4	3	5	2	4	5	9	3	7	5	.	.	.	48	.	.	.	.	.	.	.	.	.
**S23**	.	.	.	.	.	.	.	.	.	.	.	.	.	.	.	.	.	.	.	.	.	.	.	.	.	.	2	.	.	1	1	2	2	2	3	2	1	.	1	1	.	.	.	.	.	.	.	#	.	.	.	.	.
**S24**	.	.	.	.	.	.	.	.	.	.	.	.	.	.	.	.	.	.	.	.	.	.	.	.	.	.	.	.	.	1	2	3	1	4	3	3	5	1	3	3	21	2	5	.	.	.	.	.	.	.	.	.	.
**S25**	.	.	.	.	.	.	.	.	.	.	.	.	.	.	.	.	.	.	.	.	.	.	.	.	.	.	.	.	.	2	2	4	3	3	7	3	6	4	4	5	.	.	.	.	.	.	.	.	.	41	.	.	.
**S26**	.	.	.	.	.	.	.	.	.	.	.	.	.	.	.	.	.	.	.	.	.	.	.	.	.	.	2	.	1	1	2	2	.	1	1	1	3	1	3	1	.	.	.	.	.	.	17	.	.	.	.	.	.
**S27**	.	.	.	.	.	.	.	.	.	.	.	.	.	.	.	.	.	.	.	.	.	.	.	.	.	.	.	1	1	1	1	1	.	2	1	1	3	1	1	1	.	.	.	13	.	.	.	.	.	.	.	.	.
**S28**	.	.	.	.	.	.	.	.	.	.	.	.	.	.	.	.	.	.	.	.	.	.	.	.	2	1	1	.	.	1	1	2	3	1	2	1	2	1	1	1	.	.	.	.	.	.	.	.	16	.	.	.	.
**S29**	1	1	1	1	1	1	1	1	1	1	1	1	1	1	1	.	1	1	1	1	1	.	1	1	1	1	1	4	1	1	1	1	1	1	1	1	2	1	1	1	.	.	.	.	.	.	.	.	.	.	.	13	.
**S30**	.	.	.	.	.	.	.	.	.	.	.	.	.	.	.	.	.	.	.	.	.	.	.	.	.	.	.	.	1	.	2	3	2	3	2	3	5	2	2	2	18	7	2	.	.	.	.	.	.	.	.	.	.
**S31**	.	.	.	.	.	.	.	.	.	.	.	.	.	.	.	.	.	.	.	.	.	.	.	.	.	.	.	.	2	1	1	1	.	1	1	1	2	1	1	1	9	1	.	.	.	.	.	.	.	.	.	.	.
**S32**	.	.	.	.	.	.	.	.	.	.	.	.	.	.	.	.	.	.	.	.	.	.	.	.	.	.	.	.	18	1	1	2	2	2	2	2	4	1	1	1	.	.	18	.	.	.	.	.	.	.	.	.	.
**S33**	.	.	.	.	.	.	.	.	.	.	.	.	.	.	.	.	.	.	.	.	.	.	.	.	.	.	.	.	.	1	1	1	1	2	1	1	3	1	1	1	13	.	.	.	.	.	.	.	.	.	.	.	.
**S34**	.	.	.	.	.	.	.	.	.	.	.	.	.	.	.	.	.	.	.	.	.	.	.	.	.	.	.	.	.	.	.	4	3	.	.	.	.	.	.	.	7	.	.	.	.	.	.	.	.	.	.	.	.
**S35**	.	.	.	.	.	.	.	.	.	.	.	.	.	.	.	.	.	.	.	.	.	.	.	.	.	.	.	.	.	.	1	2	2	1	.	1	2	1	.	.	10	.	.	.	.	.	.	.	.	.	.	.	.
**S36**	.	.	.	.	.	.	.	.	.	.	.	.	.	.	.	.	.	.	.	.	.	.	.	.	.	.	.	.	.	.	1	2	2	2	1	1	3	2	.	.	14	.	.	.	.	.	.	.	.	.	.	.	.
**S37**		.	.	.					.	.	.	.		.									.	.	.	.	.	.	.	.	.	1	1	2	1	.	3	.	.	.	7												.
**S38**	.	.	.	.	.	.	.	.	.	.	.	.	.	.	.	.	.	.	.	.	.	.	.	.	.	.	.	.	.	2	2	4	2	2	3	2	3	2	5	3	24	3	1	.	.	.	.	.	.	.	.	.	.
**S39**	.	.	.	.	.	.	.	.	.	.	.	.	.	.	.	.	.	.	.	.	.	.	.	.	.	.	.	.	.	.	.	.	.	.	.	.	.	.	4	3	6	1	.	.	.	.	.	.	.	.	.	.	.
**S40**	.	.	.	.	.	.	.	.	.	.	.	.	.	.	.	.	.	.	.	.	.	.	.	.	.	.	.	.	.	.	.	.	.	.	.	.	.	.	2	2	3	1	.	.	.	.	.	.	.	.	.	.	.
**S41**	.	.	.	.	.	.	.	.	.	.	.	.	.	.	.	.	.	.	.	.	.	.	.	.	.	.	.	.	.	.	.	.	.	.	.	.	.	.	3	2	5	.	.	.	.	.	.	.	.	.	.	.	.
**S42**	.	.	.	.	.	.	.	.	.	.	.	.	.	.	.	.	.	.	.	.	.	.	.	.	.	.	.	.	.	.	.	.	.	.	.	.	.	.	3	2	.	.	3	2	.	.	.	.	.	.	.	.	.
**S43**	.	.	.	.	.	.	.	.	.	.	.	.	.	.	.	.	.	.	.	.	.	.	.	.	.	.	.	.	.	.	3	11	8	3	.	3	9	2	.	.	38	1	.	.	.	.	.	.	.	.	.	.	.
**S44**	.	.	.	.	.	.	.	.	.	.	.	.	.	.	.	.	.	.	.	.	.	.	.	.	.	.	.	.	4	5	2	1	.	2	1	2	6	1	1	1	18	.	.	.	.	.	.	.	.	.	.	.	.
**S45**	.	.	.	.	.	.	.	.	.	.	.	.	.	.	.	.	.	.	.	.	.	.	.	.	.	.	.	.	.	.	1	.	.	3	.	3	5	1	.	.	14	.	.	.	.	.	.	.	.	.	.	.	.
**S46**	.	.	.	.	.	.	.	.	.	.	.	.	.	.	.	.	.	.	.	.	.	.	.	.	.	.	3	.	.	.	1	1	1	1	1	.	2	.	1	1	12	.	.	.	.	.	.	.	.	.	.	.	.
**S47**	.	.	.	.	.	.	.	.	.	.	.	.	.	.	.	.	.	.	.	.	.	.	.	.	.	.	.	.	4	1	.	.	.	1	.	1	5	.	.	.	7	.	.	.	.	.	.	.	.	.	.	.	.

**S48**	.	.	.	.	.	.	.	.	.	.	.	.	.	.	.	.	.	.	.	.	.	.	.	.	.	.	.	.	.	.	.	.	.	1	.	1	2	.	.	.	.	.	3	.	.	.	.	.	.	.	.	.	1
**S49**	.	.	.	.	.	.	.	.	.	.	.	.	.	.	.	.	.	.	.	.	.	.	.	.	.	.	8	.	.	.	.	.	.	.	.	.	.	.	.	.	6	.	2	.	.	.	.	.	.	.	.	.	.
**S50**	.	.	.	.	.	.	.	.	.	.	.	.	.	.	.	.	.	.	.	.	.	.	.	.	.	.	4	.	.	.	.	.	.	.	.	.	.	.	.	.	4	.	.	.	.	.	.	.	.	.	.	.	.
**S51**	.	.	.	.	.	.	.	.	.	.	.	.	.	.	.	.	.	.	.	.	.	.	.	.	.	.	5	.	.	.	.	.	.	.	.	.	.	.	.	.	3	.	2	.	.	.	.	.	.	.	.	.	.
**S52**	.	.	.	.	.	.	.	.	.	.	.	.	.	.	.	.	.	.	.	.	.	.	.	.	.	.	5	.	.	.	.	.	.	.	.	.	.	.	.	.	5	.	.	.	.	.	.	.	.	.	.	.	.
**S53**	.	.	.	.	.	.	.	.	.	.	.	.	.	.	.	.	.	.	.	.	.	.	.	.	.	.	2	.	2	.	.	.	.	.	.	.	.	.	.	.	2	.	.	.	.	.	.	.	.	.	.	.	.
**S54**	.	.	.	.	.	.	.	.	.	.	.	.	.	.	.	.	.	.	.	.	.	.	.	.	.	.	.	.	.	.	1	.	.	.	.	.	.	.	4	2	6	.	1	.	.	.	.	.	.	.	.	.	.
**S55**	.	.	.	.	.	.	.	.	.	.	.	.	.	.	.	.	.	.	.	.	.	.	.	.	.	.	.	.	14	.	.	.	.	.	.	.	.	.	.	.	.	.	.	.	.	.	.	.	.	.	.	.	.
**S56**	.	.	.	.	.	.	.	.	.	.	.	.	.	.	.	.	.	.	.	.	.	.	.	.	.	.	.	.	4	.	.	.	.	.	.	.	.	.	.	.	.	.	.	.	.	.	.	.	.	.	.	.	.
**S57**	.	.	.	.	.	.	.	.	.	.	.	.	.	.	.	.	.	.	.	.	.	.	.	.	.	.	.	.	4	.	.	.	.	.	.	.	.	.	.	.	.	.	.	.	.	.	.	.	.	.	.	.	.
**S58**	.	.	.	.	.	.	.	.	.	.	.	.	.	.	.	.	.	.	.	.	.	.	.	.	.	.	.	.	.	.	.	2	1	.	.	.	.	.	.	.	.	.	3	.	.	.	.	.	.	.	.	.	.
**S59**	.	.	.	.	.	.	.	.	.	.	.	.	.	.	.	.	.	.	.	.	.	.	.	.	.	.	.	.	.	.	.	2	2	.	.	.	.	.	.	.	4	.	.	.	.	.	.	.	.	.	.	.	.
**S60**	.	.	.	.	.	.	.	.	.	.	.	.	.	.	.	.	.	.	.	.	.	.	.	.	.	.	.	.	.	.	.	.	.	.	.	.	.	.	1	1	1	.	1	.	.	.	.	.	.	.	.	.	.
**S61**	.	.	.	.	.	.	.	.	.	.	.	.	.	.	.	.	.	.	.	.	.	.	.	.	.	.	.	.	1	1	.	.	.	.	.	.	.	.	1	.	.	.	.	.	.	.	.	.	.	.	2	.	.
**S62**	.	.	.	.	.	.	.	.	.	.	.	.	.	.	.	.	.	.	.	.	.	.	.	.	.	.	1	1	.	.	.	.	.	.	.	.	.	.	.	.	.	.	.	.	.	.	.	.	.	.	.	.	2
**S63**	.	.	.	.	.	.	.	.	.	.	.	.	.	.	.	.	.	.	.	.	.	.	.	.	.	.	.	.	4	.	.	.	.	.	.	.	.	.	.	.	.	.	.	.	.	.	.	.	.	.	.	.	.
**S64**	.	.	.	.	.	.	.	.	.	.	.	.	.	.	.	.	.	.	.	.	.	.	.	.	.	.	.	.	4	.	.	.	.	.	.	.	.	.	.	.	.	.	.	.	.	.	.	.	.	.	.	.	.
**S65**	.	.	.	.	.	.	.	.	.	.	.	.	.	.	.	.	.	.	.	.	.	.	.	.	.	.	.	.	9	.	.	.	.	.	.	.	.	.	.	.	.	.	.	.	.	.	.	.	.	.	.	.	.
**S66**	.	.	.	.	.	.	.	.	.	.	.	.	.	.	.	.	.	.	.	.	.	.	.	.	.	.	.	.	3	.	.	.	.	.	.	.	.	.	.	.	.	.	.	.	.	.	.	.	.	.	.	.	.
**S67**	.	.	.	.	.	.	.	.	.	.	.	.	.	.	.	.	.	.	.	.	.	.	.	.	.	.	.	.	7	.	.	.	.	.	.	.	.	.	.	.	.	.	.	.	.	.	.	.	.	.	.	.	.
**S68**	.	.	.	.	.	.	.	.	.	.	.	.	.	.	.	.	.	.	.	.	.	.	.	.	.	.	3	.	.	.	.	.	.	.	.	.	.	.	.	.	.	.	.	.	.	.	.	.	.	3	.	.	.
**S69**	.	.	.	.	.	.	.	.	.	.	.	.	.	.	.	.	.	.	.	.	.	.	.	.	.	.	.	.	.			.	.	.	.	.	.	.	1	2	.	.	.	3	.	.	.	.	.	.	.	.	.
**S70**	.	.	.	.	.	.	.	.	.	.	.	.	.	.	.	.	.	.	.	.	.	.	.	.	2	2	.	.	.	.	.	.	.	.	.	.	.	.	.	.	.	.	.	.	.	.	.	.	.	.	.	.	.
**S71**	.	.	.	.	.	.	.	.	.	.	.	.	.	.	.	.	.	.	.	.	.	.	.	.	2	4	.	.	.	.	.	.	.	.	.	.	.	.	.	.	.	.	.	.	.	.	.	.	.	.	.	.	.
**S72**	.	.	.	.	.	.	.	.	.	.	.	.	.	.	.	.	.	.	.	.	.	.	.	.	.	.	.	.	20	.	.	.	.	.	.	.	.	.	.	.	.	.	.	.	.	.	.	.	.	.	.	.	.
**S73**	.	.	.	.	.	.	.	.	.	.	.	.	.	.	.	.	.	.	.	.	.	.	.	.	.	.	.	.	.	.	.	.	.	1	.	.	2	.	.	.	3	.	.	.	.	.	.	.	.	.	.	.	.
**Orphan**	.	.	.	1	1	.	.	2	2	2	.	.	2	3	.	1	5	1	2	5	5	4	.	1	.	3	.	3	16	6	5	2	1	6	4	1	1	3	3	2													
**Total**	1	1	1	2	2	1	1	3	3	3	1	1	3	4	1	1	6	2	3	6	6	4	1	2	7	1<br>1	51	15	160	53	75	12<br>2	#<br>#	12<br>5	#<br>#	87	#<br>#	92	15<br>8	##	76<br>2	3<br>1	10<br>2	7<br>8	6<br>1	#	1<br>7	#	1<br>6	4<br>4	2	1<br>3	3

Supplementary Table S3. The distributions of land plant 2R-MYB genes in each intron pattern.

Intron pattern	Pp	Sm	Zm	Os	Ac	Sl	St	Vv	At	Csi	Mt	Pt
**a**	26	3	67	56	46	85	68	88	76	54	57	117
**b**	0	0	16	8	0	6	4	2	2	2	0	4
**c**	4	0	21	13	6	8	6	11	15	9	7	20
**d**	1	3	22	11	5	4	5	3	5	5	5	12
**e**	8	1	8	4	4	3	4	6	8	4	4	13
**f**	2	2	11	5	2	4	5	4	7	3	11	4
**g**	2	0	4	1	2	0	0	1	1	1	1	3
**h**	2	0	1	1	1	2	2	2	3	2	0	1
**i**	1	0	1	1	1	2	2	1	2	1	1	2
**j**	3	0	3	5	7	6	3	3	7	3	4	6
**k**	0	0	2	0	0	0	0	0	0	0	0	0
**l**	1	4	1	1	1	1	1	1	1	1	0	2
**other**	1	2	1	0	0	1	2	3	0	2	2	2
**Total**	51	15	158	106	75	122	102	125	127	87	92	186

Supplementary Table S4. The assessment of conserved motifs in each subfamily.

Motif	E-vlaue	Width	Sequence
1	1.50E-1922	15	[SQ][MR]GIDPVTH[KR]PLSEx
2	2.10E-112	21	SPxSPxSSSSExSSSSSSxxx
3	1.80E-179	21	S[SE][ED]xF[PA][EQ]ID[EDG][SN]FW[ST][ED][TPV][LF][SL][AM][DE]N
4	1.40E-183	15	DD[DG]M[DE]FW[LY][ND][LV][FL][MI][KQ][SA]G
5	3.40E-30	15	[IP][PE][IFL][DE][IS][DG]P[DE][FIL]WS[IM][IL]D[GS]
6	8.90E-81	23	[EG][EA][ED][EG]K[EQ][WK]WLE[YD]LENELGL[WE]GTx[DE]
7	9.50E-283	28	[TP]xSxD[KN][AS]P[TA]D[YI]FL[RK][IL]WNSE[VAI]G[EDK][SA]FRKE[NS]
8	1.40E-408	29	A[SLC][PA][LSA][TA]RH[MK]AQWE[SNT]ARLEAEARLS[RL][EL]SLL[MF]
9	6.20E-155	40	EDV[ML]AGSD[ST]SSSN[ED]LEDSS[DE][ST][AS]LQLLLDFP[GS][NG]NDMSFL[EG][GH]
10	5.90E-57	25	C[CIN]VKTPEVLSC[FQ][AP]D[MI]TM[GD]QTE[HD]K[AV][PA]
11	2.90E-34	20	[DN]NNN[NS][TI][GN][CS][MI][IQ][ST]EL[SR]TSF[QL]QQ
12	4.70E-50	74	PLS[SL]QILCN[ST]DARAPSAWEICDP[IV]YV[LS]PE[LF]SED[ILV]ISSS[YN][NK][MV]C[TR][SI]T[IKR]D[YD]Q[ST]TS[TS]DS[TI]LD[VM]LHSD[CS][IR][DV][SP][LA][EI][LS][EH][ID]D[HN]
13	8.00E-43	13	SR[KQR]I[HY][TS][FY]RR[KP][SY][TN][EA]
14	1.60E-44	21	KLPG[AG]xKR[RK]GGRTS[RG]S[AS]MKKN
15	4.40E-313	12	PDLNL[DE]L[SC]I[SG][LP]P
16	4.80E-234	15	[KQ]NxV[IF]K[PT][KR][PA]V[RK]F[TS]TA
17	2.40E-104	11	L[SR]KK[LV]xSxExK
18	2.00E-151	29	[NS]FN[VG][DN][ED]FFDFSNE[GD][PS][LC][NG]LEW[MV][SN][KR]FLE[LI]DE
19	2.10E-44	21	P[NT]DN[MT]L[ED]K[LVI][YF]EEY[LQ]QLL[KNS]EDD
20	1.70E-90	15	[NG][DE]D[TV]FSSFL[DN]SLIN[ED]
21	1.20E-97	21	[ND][HVI][EKV][EV][TE][TI][ND][ST]SKSW[CS][DQ]LL[LV][NE]D[SC][IM]
22	9.00E-64	21	R[HQ]SE[MI]VRPN[GR]C[NS][GN]YG[CS]S[ND][CH][AS][SF]
23	1.60E-46	21	T[EH]DDSER[IT]AFGMA[DN][VI]N[LF][VL]E[TA]E
24	1.60E-111	21	N[SC]T[SC]SM[SW][SN]L[AE]D[QE][QER]SPHSD[HY]FA
25	3.50E-118	25	L[AD]ESL[LF][DY]LNELSSPDCNLLSSPSD[CF]
26	7.40E-53	64	[KQ]I[AV]VNIE[TN]N[VI][TN]DAQSFDEQ[SL][RK]TLL[QE]ED[LS]TME[IK]CS[HR][PH]H[PN]NMTSL[MI]LEDEEL[IF]FK[QH]NGITSEGVL[GV]
27	3.80E-98	28	FE[DV]MCSDQSNMQRVNS[FL]T[VF][AP]DDQG[MI]EE[FL]
28	1.30E-42	15	TDFG[LS]EE[FL]Y[RGM][GE][PAL]ATL
29	2.60E-291	15	xDxLWD[NG]LW[NS][LM][DE]DIW
30	2.70E-146	41	KKAK[SLN][SP][TQS]D[NQ][AS][ED]KA[KR][NRT][RQ][LY]L[KR]RQQF[QH]QQQQQ[QL][QL]QQQQQQQQQQ
31	2.80E-80	17	[ST]P[SE][SN]SS[ST][SAT]SSSSSSSSD
32	4.00E-913	21	K[HQ]A[KR]QL[KN][CI]D[VA]NSKQF[KR]D[TA][MV]RY
33	6.00E-444	21	LWMPRL[LVI][EQ][RK]I[QE]A[AS][SA][AS]SSSxxx
34	1.70E-42	15	[DN]NRVHFERST[GR]S[YD]CL
35	1.10E-192	57	[SP][DE]LD[SGI][AE]EP[SL][TS]SFP[TGR][QK]N[TS]E[ED][IA][AE]VQ[TAI][NI][KT]T[PQ][YC]LQ[DE]DIS[SP]Q[VA]IPI[SH]E[GV]N[YD][EDG][YN][VG]LQ[GR]EI[QHR][HGS][GPS]
36	2.30E-35	21	N[DE]DGQP[GS][AE][AT][LS]Q[EQ][HD][LF][EK][GD][SL]D[NK][QL][FV]
37	1.70E-47	21	Y[NI]F[SP]V[GR]SL[AS][TNS][LD]L[SLY]S[SE][EF][FY][ACF][DA]D[GA]
38	1.30E-59	21	I[PLT]PS[SF]SYM[VIL]ASPEAFS[DE][SN]E[ST][YH]
39	1.10E-807	29	K[NV][AS][AS]NLSHMAQWESARLEAEARL[VA]RES[KS]L
40	1.40E-141	20	[PR]P[PR]CLD[VI]LKAWQ[GM]VW[TS][KS]Lx[ST]
41	3.50E-97	20	G[AG][DS]xDDLESPTSTL[SN]FSENA
42	2.30E-35	11	V[ND][SA]SP[SIT][DG]S[PS][MV]F
43	1.40E-42	20	[GA][NG][IAF][EIG][EA]GFT[GEN]LLL[GS][NG]SLDR[DN][LP]
44	3.00E-231	15	xT[ED][ED][ED]N[KD][SN]Y[WC][NS][NDS][IL]L[NK]
45	2.10E-279	29	M[EN]HH[PS][WLP][DE][ED][HN]A[LV][RK]LQA[ED]A[AT]QLAK[LI]Q[YL]LQ[NY]L
46	2.50E-213	21	RPRTD[IF][FL][AS][SA]LP[QH]L[IL]A[LA]ANL[RK]E
47	5.50E-199	21	[SA]PRLDLLDLSS[IL]L[NS]SSLYNSS
48	6.40E-145	17	Q[PA]L[VL]NP[ED][LIV]L[RK]LAT[ST]LLS
49	2.00E-142	21	ASVLSTPSSSPTPLNS[NS]S[TS][YT][IY
50	2.00E-48	11	IP[ED][IS]LD[VI][ND][DE]F[ML]
51	1.40E-735	32	[SP][KR]GQWERRLQTDI[HN][MT]A[KR][QK]AL[CR][ED]ALS[LP][DE]K[PS][SP][SP]S

52	5.30E-618	29	[PS]S[SQ][SA][ST][TS]YASS[TA][ED]NI[SA][RK]LL[EQ]GWM[RK][SN]SPKSS
53	6.00E-359	21	xQ[PV]P[LF][ST][LM][LI]E[KS]WL[LF][DE][ED][AG][GA][GA]QxE
54	6.30E-36	36	[GE]T[MIL][AG][QLN]S[SGT][GS][GAT][GINV]N[QS][ADIV][AS][EV]E[AE]A[RQ]RCFRD[NST][LI][VL][SN]KAVRECG[KR]
55	1.30E-38	37	N[VAL]N[HIMV][AT][SG][FVY][NH]SP[LF]A[GS][AT][PT][AT][GA][LMV]RL[HY]E[CY][EP]VP[ST]PRMVLW[DN][FM]QE
56	1.80E-219	33	T[TS]LA[PL]PQ[VG][AG]HLAEAALGCFKD[ED]MLHLLT[KR]KR[IP]D
57	2.20E-52	15	D[DAN]T[IV][EQ][KR]IK[LY][GS]LSRA[IM]
58	1.70E-64	26	SS[SA]ASSFV[DE][AT]ILD[RK][DE][SK]E[MI][FL][LS][DQ]FP[EP]LL
59	1.20E-515	25	FS[PA]E[FL][LM][AS]VM[QR]EMI[RA]KEVRNYM[SA][GS][LV][EG]
60	1.30E-249	21	[ES]SSSSEPDP[PL]TSLSLSLPGS[DG]
61	9.10E-133	15	M[SN]PGSPSGSD[VSL]SDSS
62	2.50E-216	41	SLLGPEFV[DE][YF]EE[PH]PPFSSHELA[AS][IL]AT[DE][IL][SN][NS][IV]AW[IL]KSGLE[NS]S
63	1.10E-182	15	Q[VL][FY]RPV[AP]RT[GS][AG][FV]S[PV]Y
64	9.40E-91	18	C[GS]E[PR][EQ]VPS[KR]CGHGCCGN[QP]
65	2.30E-466	13	RQ[KR]GIDP[AN]THKPL
66	2.00E-107	15	x[ED][ED][IL]KW[SP][ED]YL[HNQ][TG][PT][FL][LF]
67	1.30E-120	33	[PGS][SA][SF]S[SN][NS][ST][GSN]S[SRN][GNS][GS][SN]S[SMT][SG][FIT]E[LP]QS[NT][ST]S[FIL][FL]E[NS][SN][VAI]F[SP]W
68	7.00E-117	21	QL[LQ]QT[PSA]D[IM]YDKDLQR[MIL][AS][ALV][SA]F[GE]
69	1.60E-180	21	GEWDLE[EG]L[ML][KE][DN]VSS[FL]PFLDFQ
70	1.40E-41	15	[LMI]M[TS][ML][CY]MDSSSSS[ST]SS
71	4.10E-65	15	LPK[VL][LM]F[AS][ED]WL[SD][LMS][DS]YF
72	1.80E-421	15	AR[KR]YRE[QR]S[SR][AL]Y[RG][RK]RK
73	4.00E-398	15	xKKx[VP]PFIDFLGVGA
74	9.30E-210	21	PP[QR]S[ST][LI]L[QE][NDE]YI[KR]SL[NT]LxSxST
75	8.20E-157	21	[ED][VM]D[QS]LMGx[ES]V[KN]KEMDL[MI]EM[IV]S
76	1.20E-106	11	RRQ[FN]S[RK]R[KR]CRK
77	1.10E-318	14	RQRAGLP[LI]YPP[ED][VI]Q
78	6.30E-198	21	[CS]LSPRNSGLL[DE][AD]LL[HY]E[AS]Q[TA][LM]S
79	1.10E-144	29	N[PS][AVC]TPD[ST][FL][FI]D[DE][FL]P[TP]DMFD[HSY][IL]EP[LF]PS[PS]S[ES]W
80	3.30E-122	15	[AS]VKLELPSLQYSE[TS][SD]
81	1.90E-135	21	[PQ][IL][DEK][EF][GR]KY[DP][HS]I[LF][ED][TE][FI][AN]EK[LY][VL][KQD][EV]
82	4.20E-59	11	SCxWNN[ML]P[GP][IV]C
83	8.70E-78	37	Q[IL]PQP[QT][PL]DL[PV][FL][SL]PES[QH][ED]L[LV]ARL[DG]D[PS][YN][FL][LFM][DN][VM][FL]G[THP][AIQ][DE][AT][PS]
84	1.60E-64	29	S[SN][SN][ST][ST][PT][SV][RS][PST][PS]SPSV[TS]LSLSPSTVA[AP]AP[PS][AI]
85	5.70E-43	23	C[SQ][SL]SYL[ED]N[PS][ED][QV]I[KR]M[VM]PLP[DEP][LM][VM][NK][PS]
86	1.10E-712	87	[AV][AQ]HKKEA[AT]WRL[RKS]R[VL]E[LQ]QLESEK[AS][CR][RK]RREKMEEIE[AS]K[IMV][RK][AC]LREE[QE][KA]A[AFT]L[DE][RK][IV]E[AS][ED]YREQL[AS][GA]L[AS]KH[LAV][RK]L[TA]KRRDAE[AS]KE[QA]KL[AM][ED][QA]W[ATS]
87	3.30E-86	29	ENS[NTS]S[CY][IS]N[LS]NNKR[IAV][LI][FL]P[DN]G[FI][NS]T[DP]G[TA][APS]E[SAN]A
88	4.60E-108	29	[DHL][GRV]D[CD][GC][TP][TN][AN]NQ[QL][LP]R[HP]PFAVL[AV]QN[FLV][HN][NS][VL][NK][ES][LT]
89	5.40E-298	52	FLKKDDPK[IV]TALMQQAELLSSLA[LQ]KVN[AT][ED]NT[DN]QS[LM]ENAWKVLQDFLN[QKR][ST]KE[NS]
90	1.20E-270	44	[TC]EF[ST]SPL[QH]VTPLFRSLA[AD][GA]IP[ST]PKFSESER[NQ]FLLK[TV]LG[VM]ES[PT]SP
91	3.50E-2377	66	DDPR[KR]L[RK]PGEIDPNPE[ST]KPARPDPVDMDEDEKEMLSEARARLANT[RQ]GKKAKRKAREKQLEEARRLA
92	8.40E-396	21	[SA]LQKRREL[KR]AAGI[DE][VS]RQ[RK][KR][RK]K
93	8.60E-619	25	[IV]DYNAEIPFEK[KR]PP[PA]GF[YF]D[TV][SA][DE]E[ED]R
94	5.40E-529	41	FPTTIEELEG[KR]RRVDIE[AE]Q[LE]RK[QK]D[IVA][AK][RK][NQ]KIA[QE]RQDAPSA[IV][LM]
95	5.00E-573	29	PEAVRKRSKL[MV]LPAPQ[IV]SD[HA]ELE[EQ]I[AV]K[ML]G
96	1.30E-336	25	DAI[ML]MEA[EQ]NLA[RA]L[RT][EN][ST]QTPLLGG[ED]N
97	1.30E-326	21	LHPSDFSGVTP[KR]KR[EV][IV][QA]TPNP
98	2.70E-264	17	LR[AS]GLA[SG]LP[AQ]PKN[ED][YF][EQ]I
99	4.30E-287	28	EQA[DE]E[LM][IV][RK]KE[LM][LV]A[LM]LEHD[NA]AKYP[LV]x[DE]KS
100	8.50E-686	67	D[AE]CL[DN][DQ][LV][ML]Y[FL]P[ST][RQ][NQ][RA]Y[GET][RL][AS][SA][VL]A[SG][NK][KAM][DE][RK][LI][AE][AS]L[QK]NE[FL]EN[VN][RK]K[HK]M[ED]R[ED][AT][KR]KA[AL][KR]LE[KQ]K[LI]K[VIL]L[TL]GGYQ[TR]RA
101	3.10E-287	36	K[QE][LM][ED][TQ]A[GA][TI]ELE[CT]F[REK]ALH[EK]QE[QL]LA[AI][PS]xR[IL]EALQEEVQ
102	3.40E-75	15	QKE[RL]E[RK]ELQKRY[GK][AD]L

Supplementary Table S5. The query sequences used in the identifications of MYB proteins in each species.

Genome ID NO.	Gene Name	Subfamily	Protein sequence of the MYB domain
At3g28910	AtMYB030	S1	KGGVKKGPWTPEEDIILVTYIQEHGPGNWRAVPTNTGLLRCSKSCRLRWTNYLRPGIKRGNFTEHEEKMIVHLQALLGNRWAAIASYLPQRTDNDIKNYWNTHLKKKL
At3g23250	AtMYB015	S2	KMGLKRGPWTPEEDQILVSFILNHGHSNWRALPKQAGLLRCGKSCRLRWMNYLKPDIKRGNFTKEEEDAIISLHQILGNRWSAIAAKLPGRTDNEIKNVWHTHLKKRL
At3g12820	AtMYB010	S3	KSQVKRGPWSDEESERLRSFILKNGHQNWRSLPKLAGLMRCGKSCRLRWINYLRPGLKRGNFTKEEEDTIIHLHQAYGNKWSKIASNFPGRTDNEIKNVWNTHLKKRL
At4g38620	AtMYB004	S4	KAHTNKGAWTKEEDERLVAYIKAHGEGCWRSLPKAAGLLRCGKSCRLRWINYLRPDLKRGNFTEEEDELIIKLHSLLGNKWSLIAGRLPGRTDNEIKNYWNTHIRRKL
At5g35550	AtMYB123	S5	REELNRGAWTDHEDKILRDYITTHGEGKWSTLPNQAGLKRCGKSCRLRWKNYLRPGIKRGNISSDEEELIIRLHNLLGNRWSLIAGRLPGRTDNEIKNHWNSNLRKRL
At1g66380	AtMYB114	S6	SKGLRKGAWTAEEDSLLRQCIGKYGEGKWHQVPLRAGLNRCRKSCRLRWLNYLKPSIKRGKFSSDEVDLLLRLHKLLGNRWSLIAGRLPGRTANDVKNYWNTHLSKKH
At5g49330	AtMYB111	S7	KIGLKRGRWTAEEDEILTKYIQTNGEGSWRSLPKKAGLLRCGKSCRLRWINYLRRDLKRGNITSDEEEIIVKLHSLLGNRWSLIATHLPGRTDNEIKNYWNSHLSRKI
At1g66230	AtMYB020	S8	KVGLKKGPWTAEEDRKLINFILTNGQCCWRAVPKLSGLLRCGKSCRLRWTNYLRPDLKRGLLSDYEEKMVIDLHSQLGNRWSKIASHLPGRTDNEIKNHWNTHIKTKL
At5g15310	AtMYB016	S9	KLGLKKGPWTPEEDQKLLAYIEEHGHGSWRSLPEKAGLHRCGKSCRLRWTNYLRPDIKRGKFNLQEEQTIIQLHALLGNRWSAIATHLPKRTDNEIKNYWNTHLKKRL
At5g16770	AtMYB009	S10	ENGLKKGPWTQEEDDKLIDHIQKHGHGSWRALPKQAGLNRCGKSCRLRWTNYLRPDIKRGNFTEEEEQTIINLHSLLGNKWSSIAGNLPGRTDNEIKNYWNTHLRKKL
At4g05100	AtMYB074	S11	KNGLKKGPWTPEEDQKLIDYINIHGYGNWRTLPKNAGLQRCGKSCRLRWTNYLRPDIKRGRFSFEEEETIIQLHSIMGNKWSAIAARLPGRTDNEIKNYWNTHIRKRL
At5g61420	AtMYB028	S12	GEGLKKGAWTTEEDKKLISYIHDHGEGGWRDIPQKAGLKRCGKSCRLRWTNYLKPEIKRGEFSSEEEQIIIMLHASRGNKWSVIARHLPRRTDNEIKNYWNTHLKKRL
At1g57560	AtMYB050	S13	KQKLRKGLWSPEEDEKLLNYITKHGHGCWSSVPKLAGLERCGKSCRLRWINYLRPDLKRGAFSSEEQNLIVELHAVLGNRWSQIAARLPGRTDNEIKNLWNSCIKKKL
At2g36890	AtMYB038	S14	KANVKRGPWSPEEDAKLKDYIEKQGTGGNWIALPHKAGLRRCGKSCRLRWLNYLRPNIRHGDFTEEEDNIIYSLFASIGSRWSVIAAHLQGRTDNDIKNYWNTKLKKKL
At3g27920	AtMYB000	S15	NQEYKKGLWTVEEDNILMDYVLNHGTGQWNRIVRKTGLKRCGKSCRLRWMNYLSPNVNKGNFTEQEEDLIIRLHKLLGNRWSLIAKRVPGRTDNQVKNYWNTHLSKKL
At3g48920	AtMYB045	S16	KEKQRKGLWSPEEDEKLRSHVLKYGHGCWSTIPLQAGLQRNGKSCRLRWVNYLRPGLKKSLFTKQEETILLSLHSMLGNKWSQISKFLPGRTDNEIKNYWHSNLKKGV
At3g24310	AtMYB071	S17	EEGWRKGPWTAEEDRLLIDYVQLHGEGRWNSVARLAGLKRNGKSCRLRWVNYLRPDLKRGQITPHEETIILELHAKWGNRWSTIARSLPGRTDNEIKNYWRTHFKKKT
At5g06100	AtMYB033	S18	GHALKKGPWSSAEDDILIDYVNKHGEGNWNAVQKHTSLFRCGKSCRLRWANHLRPNLKKGAFSQEEEQLIVELHAKMGNRWARMAAHLPGRTDNEIKNYWNTRIKRRQ
At5g40350	AtMYB024	S19	DAEVRKGPWTMEEDLILINYIANHGEGVWNSLAKSAGLKRTGKSCRLRWLNYLRPDVRRGNITPEEQLTIMELHAKWGNRWSKIAKHLPGRTDNEIKNFWRTKIQKYI
At1g48000	AtMYB112	S20	EIEIRRGPWTVEEDMKLVSYISLHGEGRWNSLSRSAGLNRTGKSCRLRWLNYLRPDIRRGDISLQEQFIILELHSRWGNRWSKIAQHLPGRTDNEIKNYWRTRVQKHA
At4g33450	AtMYB069	S21	KPSCQRGHWRPVEDDNLRQLVEQYGPKNWNFIAQHLYGRSGKSCRLRWYNQLDPNITKKPFTEEEEERLLKAHRIQGNRWASIARLFPGRTDNAVKNHFHVIMARRK
At3g50060	AtMYB077	S22	MADRVKGPWSQEEDEQLRRMVEKYGPRNWSAISKSIPGRSGKSCRLRWCNQLSPEVEHRPFSPEEDETIVTARAQFGNKWATIARLLNGRTDNAVKNHWNSTLKRKC
At3g55730	AtMYB109	S23	IRSKVKGPWSTEEDAVLTKLVRKLGPRNWSLIARGIPGRSGKSCRLRWCNQLDPCLKRKPFSDEEDRMIISAHAVHGNKWAVIAKLLTGRTDNAIKNHWNSTLRRKY
At1g34670	AtMYB093	S24	ENGLKKGPWTPEEDQKLIDYIHKHGHGSWRALPKLADLNRCGKSCRLRWTNYLRPDIKRGKFSAEEEQTILHLHSILGNKWSAIATHLQGRTDNEIKNFWNTHLKKKL
At5g58850	AtMYB119	S25	SKNLIKGQWTAEEDRKLIRLVRQHGERKWAMISEKLEGRAGKQCRERWHNHLRPDIKKDGWSEEEERVLVESHMRIGNKWAEIAKLIPGRTENSIKNHWNATKRRQN
At5g52600	AtMYB082	orphan	KSYVKRGLWKPEEDMILKSYVETHGEGNWADISRRSGLKRGGKSCRLRWKNYLRPNIKRGSMSPQEQDLIIRMHKLLGNRWSLIAGRLPGRTDNEVKNYWNTHLNKKP
At3g13540	AtMYB005	orphan	KMGMKRGPWTVEEDEILVSFIKKEGEGRWRSLPKRAGLLRCGKSCRLRWMNYLRPSVKRGGITSDEEDLILRLHRLLGNRWSLIAGRIPGRTDNEIKNYWNTHLRKKL
At1g18710	AtMYB047	orphan	VDGMKKGEWTAEEDQKLGAYINEHGVCDWRSLPKRAGLQRCGKSCRLRWLNYLKPGIRRGKFTPQEEEEIIQLHAVLGNRWAAMAKKMQNRTDNDIKNHWNSCLKKRL
At3g61250	AtMYB017	orphan	KIGLKKGPWTPEEDEVLVAHIKKNGHGSWRTLPKLAGLLRCGKSCRLRWTNYLRPDIKRGPFTADEEKLVIQLHAILGNRWAAIAAQLPGRTDNEIKNLWNTHLKKRL
At4g17780	AtMYB039	orphan	DKGVKKGPWLPEEDDKLTAYINENGYGNWRSLPKLAGLNRCGKSCRLRWMNYLRPDIRRGKFSDGEESTIVRLHALLGNKWSKIAGHLPGRTDNEIKNYWNTHMRKKL
At3g28470	AtMYB035	orphan	KSNVKKGLWTEEEDAKILAYVAIHGVGNWSLIPKKAGLNRCGKSCRLRWTNYLRPDLKHDSFSTQEEELIIECHRAIGSRWSSIARKLPGRTDNDVKNHWNTKLKKKL
At5g56110	AtMYB080	orphan	KENVKRGQWTPEEDNKLASYIAQHGTRNWRLIPKNAGLQRCGKSCRLRWTNYLRPDLKHGQFSEAEEHIIVKFHSVLGNRWSLIAAQLPGRTDNDVKNYWNTKLKKKL
At3g53200	AtMYB027	orphan	EETLRRGPWLEEEDERLVKVISLLGERRWDSLAIVSGLKRSGKSCRLRWMNYLNPTLKRGPMSQEEERIIFQLHALWGNKWSKIARRLPGRTDNEIKNYWRTHYRKKQ
At3g12720	AtMYB067	orphan	KHKVKRGLWSPEEDEKLLRYITTHGHPSWSSVPKLAGLQRCGKSCRLRWINYLRPDLRRGSFNEEEEQIIIDVHRILGNKWAQIAKHLPGRTDNEVKNFWNSCIKKKL
At1g63910	AtMYB103	orphan	QQKVKRGLWSPEEDEKLIRYITTHGYGCWSEVPEKAGLQRCGKSCRLRWINYLRPDIRRGRFSPEEEKLIISLHGVVGNRWAHIASHLPGRTDNEIKNYWNSWIKKKI
At3g08500	AtMYB083	orphan	KPKLRKGLWSPDEDEKLIRYMLTNGQGCWSDIARNAGLLRCGKSCRLRWINYLRPDLKRGSFSPQEEDLIFHLHSILGNRWSQIATRLPGRTDNEIKNFWNSTLKKRL
At1g14350	AtMYB124	orphan	KKERHIVTWSQEEDVILREQITLHGTENWAIIASKFKDKSTRQCRRRWYTYLNSDFKRGGWSPEEDMLLCEAQRVFGNRWTEIAKVVSGRTDNAVKNRFTTLCKKRA
AT1G09770	AtCDC5	orphan	MRIMIKGGVWKNTEDEILKAAVMKYGKNQWARISSLLVRKSAKQCKARWYEWLDPSIKKTEWTREEDEKLLHLAKLLPTQWRTIAPIVGRTPSQCLERYEKLLDAAC
AT4G32730	At3R-MYB1	3R-MYB	ARRSTKGQWTPEEDEVLCKAVERFQGKNWKKIAECFKDRTDVQCLHRWQKVLNPELVKGPWSKEEDNTIIDLVEKYGPKKWSTISQHLPGRIGKQCRERWHNHLNPGINKNAWTQEEELTLIRAHQIYGNKWAELMKFLPGRSDNSIKNHWN[illegible]
AT4G00540	At3R-MYB2	4R-MYB	TRRSTKGGWTAEEDQILTNVVKKYQGRNWKRIAECLPGSEENRRNDVQCQHRWLKVLDPSLQKGAWKKEEDELLSELVKDYMENDRPPWSKISKELPGRIGKQCRERWHNHLNPTIIKSPWTREEELILVQAQRGNGNKWAEIAKLLPGRTEN[illegible]
AT3G09370	At3R-MYB3	5R-MYB	PIRRAKGGWTPEEDETLRQAVDTFKGKSWKNIAKSFPDRTEVQCLHRWQKVLNPDLIKGPWTHEEDEKIVELVEKYGPAKWSIIAQSLPGRIGKQCRERWHNHLNPDINKDAWTTEEEVALMNAHRSHGNKWAEIAKVLPGRTDNAIKNHWN[illegible]
AT5G11510	At3R-MYB4	6R-MYB	ARRSTRGQWTAEEDEILRKAVHSFKGKNWKKIAEYFKDRTDVQCLHRWQKVLNPELVKGPWTKEEDEMIVQLIEKYGPKKWSTIARFLPGRIGKQCRERWHNHLNPAINKEAWTQEEELLLIRAHQIYGNRWAELTKFLPGRSDNGIKNHWH[illegible]
AT5G02320	At3R-MYB5	7R-MYB	PMRRAKGGWTPEEDETLRRAVEKYKGKRWKKIAEFFPERTEVQCLHRWQKVLNPELVKGPWTQEEDDKIVELVKKYGPAKWSVIAKSLPGRIGKQCRERWHNHLNPGIRKDAWTVEEESALMNSHRMYGNKWAEIAKVLPGRTDNAIKNH[illegible]
